# Molecular profiling of stroma highlights stratifin as a novel biomarker of poor prognosis in pancreatic ductal adenocarcinoma

**DOI:** 10.1038/s41416-020-0863-1

**Published:** 2020-05-07

**Authors:** Fabien Robin, Gaëlle Angenard, Luis Cano, Laetitia Courtin-Tanguy, Elodie Gaignard, Zine-Eddine Khene, Damien Bergeat, Bruno Clément, Karim Boudjema, Cédric Coulouarn, Laurent Sulpice

**Affiliations:** Univ Rennes, Inserm, Inra, CHU Rennes, Service de Chirurgie Hépatobiliaire et Digestive, Institut NuMeCan (Nutrition Metabolisms and Cancer), UMR_S 1241, Rennes, France

**Keywords:** Prognostic markers, Cancer microenvironment

## Abstract

**Background:**

Pancreatic ductal adenocarcinoma (PDAC) is a deadly cancer worldwide, as a result of a late diagnosis and limited therapeutic options. Tumour microenvironment (or stroma) plays a key role in cancer onset and progression and constitutes an intrinsic histological hallmark of PDAC. Thus we hypothesised that relevant prognostic biomarkers and therapeutic targets can be identified in the stroma.

**Methods:**

Laser microdissection of the stroma from freshly frozen PDAC was combined to gene expression profiling. Protein expression of candidate biomarkers was evaluated by immunohistochemistry on tissue microarrays (*n* = 80 tumours) and by ELISA in plasma samples (*n* = 51 patients).

**Results:**

A signature made of 1256 genes that significantly discriminate the stroma from the non-tumour fibrous tissue was identified. Upregulated genes were associated with inflammation and metastasis processes and linked to NF-Kappa B and TGFβ pathways. TMA analysis validated an increased expression of SFN, ADAMTS12 and CXCL3 proteins in the stroma of PDAC. Stromal expression of SFN was further identified as an independent prognostic factor of overall (*p* = 0.003) and disease-free survival (DFS) (*p* = 0.034). SFN plasma expression was significantly associated with reduced DFS (*p* = 0.006).

**Conclusions:**

We demonstrated that gene expression changes within the stroma of PDAC correlate with tumour progression, and we identified Stratifin as a novel independent prognostic biomarker.

## Background

Pancreatic ductal adenocarcinoma (PDAC) is expected to become the second cause of cancer-related death by 2030 in developed countries.^1^ Despite recent progress in adjuvant therapeutics, the mortality to incidence ratio remains dramatically elevated. The lack of early detection and effective treatments accounts for the poor prognosis of PDAC. Thus the identification of accurate diagnostic and prognostic biomarkers and innovative therapeutic targets is a major challenge for the management of PDAC patients.

Desmoplastic reaction is a prominent pathological and histological feature of PDAC. Growing evidence demonstrates that the tumour microenvironment (or stroma) strongly influences tumour onset, progression and therapeutic response.^2^ The stroma is a complex and dynamic entity made of myofibroblasts and cancer-associated fibroblasts, as well as numerous types of immune cells, including macrophages, mast cells, lymphocytes and plasma cells. The stroma also contains extracellular matrix (ECM) components, such as collagen fibres and hyaluronic acids.^2^ The stroma is a dynamic system that co-evolves with tumour cells. Tumour–stroma crosstalk, which involves direct cell–cell communications and soluble factors, modulates key cancer-associated processes, including tumour growth and resistance to treatments. Accordingly, the stroma has been associated with the inefficacy of gemcitabine in clinical trials though a mechanism of drug scavenging by cancer-associated fibroblast.^3^ Thus specific targeting of the tumour microenvironment might be promising, although complex. Indeed, Ozdemir et al. demonstrated that a complete depletion of the stroma in PDAC enhances tumour aggressiveness,^4^ underlying a protective role of ECM and/or stromal cells. To date, stroma-oriented therapy targeting cellular and soluble factors in PDAC is not effective, particularly in the field of immunotherapies.^5^ However, targeting the acellular compartment of the stroma may represent a promising approach. Indeed, the high hydrostatic pressure of the stroma may account for blunting effective drug delivery within the tumour. Accordingly, a novel therapeutic strategy associating hyaluronidase and gemcitabine has been developed. Although Phase 1–2 studies showed promising results by improving disease-free survival (DFS) in a subgroup of patients with an excessive hyaluronic acid accumulation in the tumour,^6^ the Phase 3 HALO 301 study failed to meet the primary endpoint underlining the difficulty of developing treatments targeting the stroma. Supporting the importance of taking into account the stromal component in the management of PDAC patients, a new integrated stratification system including both tumour and stromal features was reported to efficiently select therapies and to predict patient outcomes.^7^

Given the critical role of the stroma in cancer and based on our previous studies highlighting clinically relevant transcriptomic alterations in the stroma of hepatobiliary tumours,^8,9^ we hypothesised that relevant prognostic biomarkers and therapeutic targets could be identified by characterising the alterations of the stroma in PDAC.

## Materials and methods

### Patients

Three cohorts of patients with primary PDAC were studied at a transcriptomic (*n* = 5), histological (*n* = 80) and plasmatic level (*n* = 50) (Supplementary Fig. 1, Supplementary Tables 1 and 2). Freshly frozen and formalin-fixed paraffin-embedded (FFPE) tissues were provided by the Biobank of the Rennes University Hospital (BB-0033-00056). Written informed consent was obtained from all patients. The study protocol fulfilled national laws and regulations and was approved by the local Ethics Committee. Histological and clinical features including those recorded upon follow-up examinations were obtained from hospital charts.

### Laser capture microdissection (LCM)

LCM was performed using the Arcturus Veritas Microdissection system (Applied Biosystems, Carlsbad, CA, USA), as previously described.^9^ From frozen tissues, serial sections of 10 µm were prepared using a Leica 3050S cryostat (Leica Microsystems, Wetzlar, Germany) and mounted onto a PEN membrane glass slide (Applied Biosystems). Tissue sections were dehydrated by successive immersions (30 s, twice) in 70%, 90% and 100% ethanol solutions. Enzymatic activity was locked by the immersion in a xylene solution (1 min, twice) before performing LCM.

### RNA extraction and gene expression profiling

Total RNA was purified using an Arcturus Picopure RNA Isolation Kit (Applied Biosystems). Genome-wide expression profiling was performed using human SurePrint Human Gene Expression V3 G3 8 × 60 K microarrays (Agilent Technologies, Santa Clara, CA, USA) as described.^10^ Fifty nanograms of total RNA was purified from LCM tissues and amplified with a low input QuickAmp Labelling Kit (Agilent Technologies). The amplification yield was 1.8 ± 0.7 µg complementary RNA (cRNA), and the specific activity was 5.8 ± 3.4 pmol Cy3 per µg cRNA. Gene expression data were analysed using Feature Extraction and GeneSpring softwares (Agilent Technologies) and further analysed using R-based ArrayTools. Briefly, microarray data were normalised using the quantile normalisation algorithm, and differentially expressed genes were identified by a two-sample univariate *t* test and a random variance model, as described.^11^ Clustering analysis was done using Cluster 3.0 and TreeView 1.6 with uncentered correlation and average linkage options.

### Data mining

Enrichment for specific biological functions or canonical pathways was evaluated using Enrichr.^12^ Gene set enrichment analysis was performed using the web-based tool developed by Broad Institute (www.broad.mit.edu/gsea/). Among the deregulated genes identified by the transcriptomic analysis, an exhaustive bibliography has been performed on MEDLINE to select the most relevant proteins in the field of cancer and never studied in pancreatic carcinoma. Data mining of The Cancer Genome Atlas (TCGA) data set (TCGA-PAAC, *n* = 178 cases) was also performed to validate the data (https://portal.gdc.cancer.gov/projects/TCGA-PAAD).

### Tissue microarray (TMA)

FFPE tissues were arrayed using a Minicore 3 tissue Arrayer (Excilone, Vicq, France). After haematoxylin–eosin staining, three representative areas of stroma from each PDAC tumour (T) and of fibrous tissue from periacinar areas in the surrounding non-tumour (NT) tissue were selected by an experienced pathologist (L.C.). Selected areas were punched with a cylinder of 1 mm diameter, and the samples were transferred into a recipient paraffin block. Thus each tissue block (NT and T) was represented by three independent spots in the TMA.

### Immunohistochemistry (IHC)

IHC experiments were performed using an automated Discovery XT immunostaining device (Ventana Medical System, Tucson, AZ, USA). TMA sections (4-µm thick) were evaluated for the expression of a disintegrin and metalloproteinase with thrombospondin motifs 12 (ADAMTS12), tumour necrosis factor (TNF) superfamily member 9 (TNFSF9), stratifin (SFN), integrin subunit beta 6 (ITGB6), CXC motif chemokine ligand 3 (CXCL3) and keratin 19 (KRT19) (Supplementary Table 3). Antigens were retrieved from deparaffinised and rehydrated tissues by incubating the slides for 48 min at 95 °C in CC1 Tris-based buffer (pH 8.0) (CXCL3, ADAMTS12 and KRT19) or in Ultra CC2 citrate buffer (pH 6.0) (SFN, ITGB6 and TNFSF9) (Ventana Medical System). Detection was performed using a Streptavidin–Biotin Peroxidase Kit (OmniMap, Biotinfree DAB Detection Systems, Ventana Medical System). TMA slides were analysed by an experienced pathologist (L.C.) in a blinded manner. Staining intensity in T stroma as well as in NT tissue was scored as follows: negative (0), mild (1), moderate (2), or strong (3). The staining analysis excluded tumour epithelial cells. Given that each T and NT sample was represented in triplicate, the sum of the three values was performed to obtain a score ranging from 0 to 9. This score was finally categorised into four groups to perform the statistical analysis: 0 (scores 0–1), 1 (scores 2–3), 2 (scores 4–7), and 3 (scores 8–9).

### Enzyme-linked immunosorbent assay (ELISA)

The concentration of SFN in plasma samples was measured using an ELISA kit according to the manufacturer’s instructions (CSBEL021135HU, Cusabio Technology, Houston, TX, USA). Evaluation of the ELISA absorbance values and calculation of the plasmatic concentration was performed using a 4 Parameter Logistic (4PL) nonlinear regression model.

### Statistical analysis

Differences in protein expression (NT versus T) were evaluated by chi-squared testing. Relationship between clinical and pathological parameters and protein expression was evaluated using chi-squared of Fisher’s exact test probability test. Univariate analysis was conducted between relevant variables with cumulative survivals using log-rank test. To estimate the statistical significance of protein expression on survival, a Cox proportional hazard ratio model was conducted by using variables with a *p* value <0.1 in the univariate analysis or variables known to impact prognosis.^13^ The most suitable model was selected using a stepwise regression. *p* < 0.05 was considered statistically significant in all comparisons. As regard to SFN plasmatic expression, a Kolmogorov–Smirnov and Shapiro–Wilk test was used to test for normal distribution of concentrations. Nonparametric data were compared using Mann–Whitney *U* test and for multiple comparisons Kruskal–Wallis test. The optimal threshold to dichotomise significance SFN plasmatic level was determined using the maximally selected log-rank test.^14^ All statistical analyses were performed with SPSS 24 (IBM) and GraphPad Prism 6.0.

## Results

### Study design

To identify relevant prognostic biomarkers in PDAC, an unsupervised gene expression analysis of the stroma was performed (Supplementary Fig. 1). The testing set included five cases of freshly frozen PDAC for which stroma analysis was conducted by combining LCM and genome-wide microarray profiling. To increase the robustness of the study, two independent validating sets of patients were used to perform a supervised analysis of protein candidates selected from the testing set. First, a set of 80 FFPE PDAC arrayed on a TMA and divided into 4 prognosis groups was used. Second, an independent cohort of plasmatic samples from 51 patients taken the day before surgery, matched to 20 samples from healthy controls, was analysed by ELISA. Clinical relevance of selected protein expression was analysed using clinical and follow-up data, including oncologic outcomes.

### Major gene expression changes in the stroma of PDAC

**Fig. 1:**
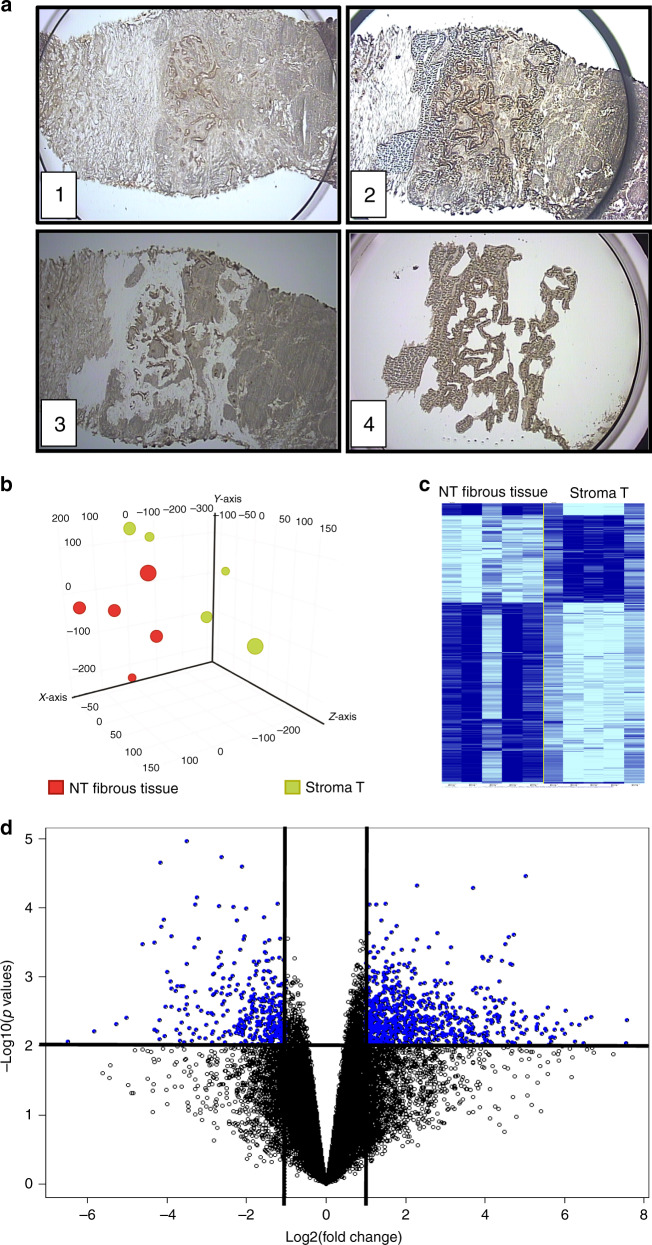
Gene expression profiling of microenvironment in PDAC. **a** Screenshots during 4 steps of LCM procedure: (1) Focusing the device on tumour area on fresh frozen tissue, a cap coated with an adhesive surface is positioned. (2) Cap in position on tissue after laser pulsed procedure. (3) Residual tissue on slide with laser dissection. (4) Selected microenvironment area on thermoplastic film of the cap. **b** Principal component analysis of global transcriptomic profiles of the five samples of stroma and five associated NT fibrous tissues. **c** Clustering analysis of genes differentially expressed between the T stroma and the surrounding NT fibrous tissue. **d** Volcano plot of 1256 non-redundant genes differentially expressed between the stroma and the surrounding NT fibrous tissue. Following microarray analysis of the 5 frozen PDAC from the testing set, genes were selected based on the significance of the differential gene expression in the T stroma versus the surrounding NT fibrous tissue (horizontal line; *p* < 0.01) and the level of induction or repression (vertical lines; fold change >2).

RNA was extracted after LCM of the stroma from freshly frozen resected PDAC specimens (Fig. 1a). For each tumour, microdissected fibrous tissue localised in periacinar areas of the NT pancreas was used as a reference. Following hybridisation on microarrays and processing of the gene expression dataset, 29 568 genes were retained for statistical analysis. Principal component analysis of global gene expression profiles highlighted two distinct groups corresponding to T and NT tissues, thus validating the LCM process (Fig. 1b). By using stringent statistical criteria (i.e. fold-change >2 and *p* < 0.01), 1256 genes were found to be differentially expressed between the T stroma and the NT fibrous tissue, including 358 upregulated and 858 downregulated genes in the stroma (Fig. 1c, d and Supplementary Table 4).

### Gene expression profiles of the stroma are associated with oncogenic pathways

**Fig. 2:**
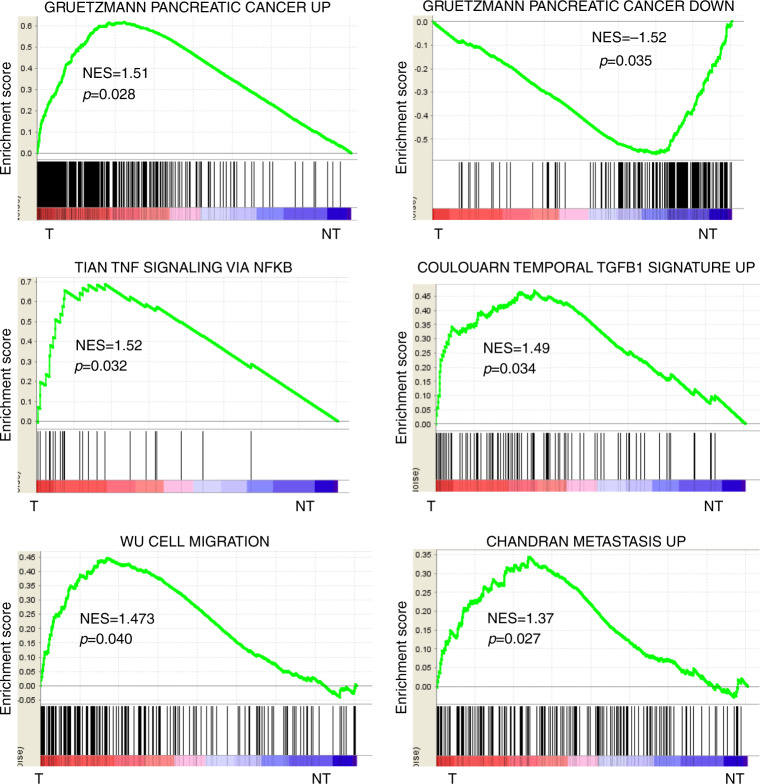
Functional analysis of the PDAC stroma signature. Gene set enrichment analysis (GSEA) using the gene expression profiles of PDAC stroma (left side, T) and adjacent NT fibrous tissue (right side, NT). As a validating step, the Gruetzmann signatures of genes upregulated and downregulated in pancreatic cancer are significantly enriched, respectively, in the stroma (T) and the non-fibrous tissue (NT). GSEA demonstrated a significant enrichment of gene signatures associated with TNF signalling via NFKB pathways, TGF-β pathway, cell migration and metastasis in the stroma.

Differentially expressed genes were categorised into functional modules. Upregulated genes in the stroma were related to cell cycle, cell migration and ECM remodelling, and included numerous soluble and transcription factors linked to cancer (e.g. ADAMTS12, CXCL3, FAP, ITGB4, LAMC2, LIF, MET, RUNX1, SFN, TNFSF9) (Supplementary Table 5). Unsupervised data mining by GSEA was also performed using curated gene sets (i.e. C2 collection). As a first validation, the analysis identified a significant enrichment of gene signatures previously shown to be upregulated and downregulated in PDAC, respectively, in the gene expression profiles of T stroma and NT fibrous tissue (Fig. 2). An enrichment of signatures associated with TNF/nuclear factor (NF)-Kappa B and transforming growth factor beta (TGFβ) signalling pathways, cell migration and metastasis, inflammation and hypoxia were also highlighted (Fig. 2 and Supplementary Fig. 2).

### Validation of transcriptomic profiles at a protein level

**Fig. 3:**
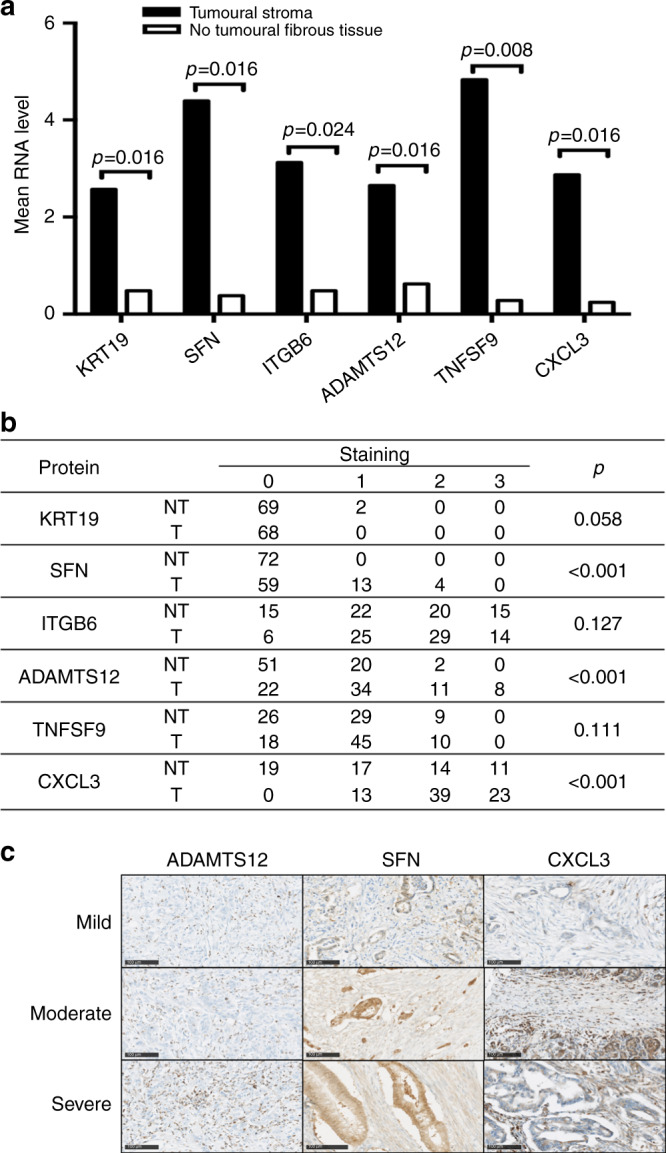
Validation of mRNA profiles at a protein level. **a** mRNA analysis of the selected genes demonstrated a significant increase in the expression of KRT19, SFN, ITGB6, ADAMTS12, TNFSF9 and CXCL3 in the stroma of PDAC as compared to the adjacent NT fibrous tissue. *p* Value was determined by using a two-tailed Student’s *t* test. **b** Immunohistological analysis of KRT19, SFN, ITGB6, ADAMTS12, TNFSF9 and CXCL3 protein expression in the stroma (T) and the surrounding NT fibrous tissue (NT) of an independent set of 80 patients with resected PDAC. Staining was scored as described in the “Materials and methods” section: negative (0), mild (1), moderate (2), or strong (3). The expression of SFN, ADAMTS12 and CXCL3 was significantly increased in the stroma of PDAC. **c** Representative strong immunostaining in stroma of PDAC of SFN, ADAMTS12 and CXCL3.

The expression of several differentially expressed genes was further evaluated at a protein level in an independent set of 80 PDAC arrayed on TMA (Supplementary Table 1). According to the enriched functional categories identified by the transcriptomic analysis, six candidate genes were selected (Fig. 3a). ADAMTS12 and SFN are associated with ECM remodelling, ITGB6 and KRT19 to the integrin pathway and TNFSF9 to the TNF pathway. CXCL3 was selected as a relevant soluble factor involved in inflammation acting as a potent chemoattractant for neutrophils. A statistically significant (*p* < 0.05) upregulation of SFN, ADAMTS12 and CXCL3 in the stroma of PDAC was confirmed at the protein level in the validating cohort (Fig. 3b, c).

### Clinical relevance of the overexpressed proteins

**Fig. 4:**
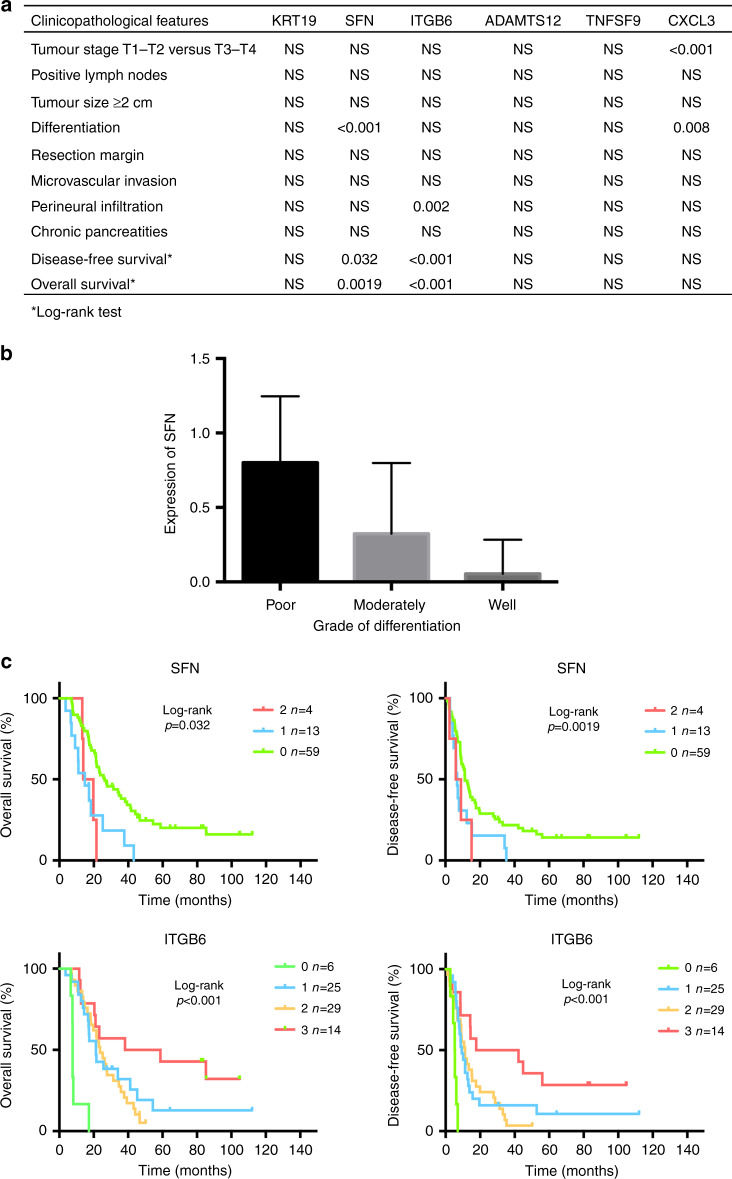
Stromal overexpression of the selected proteins correlates with pathological feature and prognosis in patients with PDAC. **a** Statistical analysis of protein expression in view of clinical and pathological features of PDAC patients. **b** Expression of SFN is associated with a poor differentiation. **c** Kaplan–Meier curves and log-rank analysis of overall survival (OS) (left panels) and disease-free survival (DFS) (right panels) according to the expression of SFN or ITGB6 in the stroma of resected PDAC. Staining was scored as negative (0), mild (1), moderate (2) or strong (3).

The immunostaining intensity of the selected proteins in the stroma of PDAC was analysed according to the clinical and pathological features of the FFPE set (Supplementary Table 1). Statistically significant associations were identified between SFN, ITGB6 and CXCL3 staining and clinical data (Fig. 4a). Expression of SFN was associated with differentiation (*p* < 0.001; Fig. 4b) and ITGB6 with perineural invasion (*p* < 0.001). CXCL3 was associated Tumour, Node, Metastasis stage and differentiation, which are associated with prognosis. No significant association was found between ADAMS12, TNFSF9 or KRT19 staining and the clinical variables tested (Fig. 4a). Importantly, SFN and ITGB6 expression was significantly correlated with patient overall survival (OS) and DFS supporting these proteins as candidate prognostic biomarkers (Fig. 4c).

### SFN expression in the stroma of PDAC is associated with a poor prognosis

From the clinical and pathological features of the FFPE validating set, a univariate analysis of risk factors influencing OS and/or DFS was performed. OS was influenced by positive lymph nodes (*p* = 0.030), quality of resection (*p* = 0.023), perineural infiltration (*p* = 0.013) and the staining of ITGB6 (*p* < 0.001) and SFN (*p* = 0.0019) in the stroma (Table 1A). DFS was significantly associated with positive lymph nodes (*p* = 0.047), a tumour size ≥2 cm (*p* = 0.043), perineural infiltration (*p* = 0.006) and the staining of ITGB6 (*p* < 0.001) and SFN (*p* = 0.032) (Table 1B). Based on these results, a multivariate analysis highlighted SFN in the stroma of PDAC as an independent risk factors for reduced OS (*p* = 0.003, hazard ratio (HR) = 2.145 95% confidence interval (CI) [1.327; 3.305]) and reduced DFS (*p* = 0.034, HR = 1.668, 95% CI [1.043; 2.546]). Conversely, a strong staining of ITGB6 was independently associated with an increased OS (*p* = 0.007, HR = 0.600, 95% CI [0.416; 0.867]) and DFS (*p* = 0.005, HR = 0.615, 95% CI [0.437; 0.861]) (Table 1). An analysis of the only epithelial expression of SFN excluded any association with OS and DFS (Supplementary Fig. 3). Interestingly, from this independent cohort of PDAC (178 patients), we observed that SFN expression from bulk tumours (i.e. without LCM of the stroma) was predictive of patient survival. High expression of SFN was associated with a statistically significant reduction of both OS and DFS (*p* < 0.05; Supplementary Fig. 4).

**Table 1:** Univariate and multivariate analysis of factors associated with overall (A) and disease-free (B) survival in the TMA validating set.

Clinicopathological features	Univariate (log-rank test)	Multivariate (Cox model)		
	*p* Value	Hazard ratio	[95% CI]	*p* Value
(A)				
Age	0.183			—
Gender	0.915			—
TNM score T1–T2 versus T3–T4	0.3			—
Positive lymph nodes	**0.03**	2.171	[1.197; 4.177]	**0.010**
Tumour size ≥2 cm	0.093			—
Differentiation	0.057			—
R1 resection	**0.023**	1.653	[0.764; 3.320]	0.192
Microvascular invasion	0.058			—
Perineural infiltration	**0.013**	1.256	[0.657; 2.571]	0.502
Chronic pancreatitis	0.87			—
Adjuvant chemotherapy	0.971			—
SFN staining	**0.0019**	2.145	[1.327; 3.305]	**0.003**
ITGB6 staining	**<0.001**	0.600	[0.416; 0.867]	**0.007**
(B)				
Age	0.867			—
Gender	0.783			—
TNM score T1–T2 versus T3–T4	0.491			—
Positive lymph nodes	**0.047**	1.792	[1.010; 3.296]	**0.046**
Tumour size ≥2 cm	**0.043**	1.522	[0.793; 3.176]	0.214
Differentiation	0.252			—
R1 resection	0.096			—
Microvascular invasion	0.066			—
Perineural infiltration	**0.006**	1.446	[0.768; 2.902]	0.261
Chronic pancreatitis	0.582			—
Adjuvant chemotherapy	0.39			—
SFN staining	**0.032**	1.668	[1.043; 2.546]	**0.034**
ITGB6 staining	**<0.001**	0.615	[0.437; 0.861]	**0.005**

Bold values indicate statistical significance *p* < 0.05.

### PDAC recurrence is associated with an elevated plasmatic level of SFN

According to the prognostic value of SFN in the stroma, its plasmatic expression was evaluated in a third independent set of 53 patients who underwent surgical resection for PDAC (Supplementary Table 2). From this validating cohort, two patients were excluded owing to an early postoperative death. SFN plasmatic levels were compared to 20 healthy controls. SFN plasmatic levels were higher in the PDAC set (0.81 versus 0.61 ng/ml), although the difference was not statistically significant (Supplementary Fig. 5). A threshold of 1.043 ng/ml has been determined to dichotomise high and low plasmatic expression of SFN. However, the analysis of clinical data identified plasmatic SFN level as an independent risk factor of PDAC recurrence in both univariate and multivariate analysis (*p* = 0.006, HR = 2.38, 95% CI [1.02; 5.531]; Table 2 and Supplementary Fig. 6).

**Table 2:** Univariate and multivariate analysis of factors associated with overall (A) and disease-free (B) survival in plasmatic validating set (log-rank test and Cox model).

Variables	Univariate	Multivariate		
	*p* Value	Hazard ratio	[95% CI]	*p* Value
(A)				
Age	0.127			—
Gender	0.247			—
TNM score T1–T2 versus T3–T4	0.162			—
Positive lymph nodes	0.815			—
Size ≥2 cm	0.934			—
Differentiation	0.715			—
Positive margin	0.800			—
Lymphovascular invasion	0.558			—
Perineural invasion	0.826			—
Chronic pancreatitis	0.115			—
Adjuvant chemotherapy	0.952			—
High SFN plasmatic level	0.308			—
(B)				
Age	0.161	0.955	[0.90; 1.003]	0.068
Gender	0.421			—
TNM score T1–T2 versus T3–T4	0.593			—
Positive lymph nodes	0.591			—
Size ≥2 cm	0.659			—
Differentiation	0.662			—
Positive margin	0.865			—
Lymphovascular invasion	0.105	1.94	[0.83; 4.51]	0.123
Perineural invasion	0.790			—
Chronic pancreatitis	0.508			—
Adjuvant chemotherapy	0.369			—
High SFN plasmatic level	**0.019**	2.380	[1.02; 5.531]	**0.006**

Bold values indicate statistical significance *p* < 0.05.

## Discussion

PDAC has become a major public health issue due to an increase in incidence and limited therapeutic progress. The present study focusses on the clinical relevance of gene expression in the stroma of PDAC. First, by combining LCM and gene expression profiling, we identified a signature specific of the stroma as compared to the adjacent NT fibrous tissue. Second, we validated the upregulation of SFN at the protein level in an independent cohort of 80 resected PDAC and highlighted its clinical relevance as an independent risk factor for OS and DFS. Finally, we demonstrated in a third independent cohort of 51 patients that a high plasma level of SFN was independently associated with recurrence.

An originality of the present work relies on the combination of LCM and genomic profiling to analyse the transcriptomic changes, which specifically occur in the stroma. LCM-based approach is relevant to provide quantitative and qualitative insights into cell biology from cells grown in their native microenvironment in complex tissues.^15^ However, data from LCM must be analysed cautiously in regard to the possible risk of contamination from adjacent epithelial cells. Thus, in our transcriptomic profiling of the stroma, KRT19 overexpression was certainly related to adjacent tumour epithelial cells. Indeed, IHC analysis highlighted a specific KRT19 staining only in tumour cells but not in the stroma. LCM does not provide a pure signal but an enrichment of stroma’s cells. To date, LCM has been validated in several cancers,^9,16^ including PDAC,^17,18^ but never specifically focusing on the stroma, as in our study. Early transcriptomic studies on PDAC, including those analysing precursor lesions (i.e. pancreatic intraepithelial neoplasia), highlighted genes involved in cell proliferation, migration and ECM remodelling.^18^ Here we specifically focussed on the stroma that is a prominent histological hallmark of PDAC representing from 50% to 90% of the whole tumour. It is suggested that the stroma dynamics contributes to the natural history of the disease and is related to its prognosis. By physicomechanics of high pressure inducing hypovascularity and vascular collapse, the stroma contributes to chemoresistance.^19,20^ Accordingly, our transcriptomic profiling highlighted specific gene expression signatures associated with hypoxia. The crosstalk between tumour epithelial cells and the stroma is also critical in tumour progression.^9,16,21–24^ In hepatocellular carcinoma (HCC), we recently characterised a bidirectional crosstalk between tumour cells and stellate cells. This crosstalk was associated with the secretion of pro-inflammatory and pro-angiogenic factors and a poor prognosis in HCC patients.^25^ Co-culturing pancreatic stellate cells and tumour epithelial cells would be relevant to identify genes and soluble factors involved in this crosstalk in PDAC.

Our study also provides new insights into potential therapeutic targets. Notably, a significant enrichment of TNFα signatures associated with NF-kappa B activation was highlighted. This pathway exhibits anti-apoptotic activities and is associated with tumour cell proliferation, metastasis and chemoresistance.^26^ To date, NF-Kappa B activation was only demonstrated in cell lines or mouse models of PDAC.^26–28^ Interestingly, promising results of combined therapies using NF-Kappa B inhibitors and chemotherapy agent were reported in biliary pancreatic cancers, with benefits in improving chemosensitivity and patient survival^29^. We also report an overexpression of CXCL3, both at the RNA and protein levels, associated with tumour stage and differentiation. Although alteration of numerous chemokines with pro- or anti-oncogenic effect were reported, CXCL3 and its CXCR2 receptor have been poorly investigated in PDAC.^30^ Further explorations would be interesting given that CXCL3–CXCR2 axis is associated with tumour cell proliferation and migration, notably in prostate and breast cancer.^31–33^

The most prominent result of the study was the significant association between the expression of SFN and ITGB6 and the prognosis of PDAC patients. Indeed, our multivariate analysis revealed that both SFN and ITGB6 were independent factors influencing OS and DFS. SFN, also known as 14-3-3-σ or stratifin, is a protein member of 14-3-3 family. Proteins from this family are associated with oncogenic pathways, including phosphoinositide-3 kinase/AKT or RTK/RAS signalling.^34,35^ SFN overexpression has been previously reported in lung and colon cancer.^35,36^ In PDAC, overexpression of SFN was first reported in a profiling study of whole tumours.^18^ The level of SFN expression is to be controlled by epigenetics mechanisms.^37^ SFN overexpression in cell lines was associated with resistance to γ-irradiation and anticancer drugs by causing resistance to treatment-induced apoptosis and G2/M arrest.^38^ In our study, overexpression of SFN in the stroma was correlated with worse prognosis, in agreement with previous reports of SFN expression in PDAC associated with lymph node metastasis. Based on our data demonstrating that epithelial SFN is not associated with survival, we can assume that a clinically relevant expression of SFN within the stroma can be captured from expression profiles derived from bulk tumours (as in the TCGA data set). In HCC, paracrine effect of tumour-derived SFN on metalloprotease expression by stromal cells was reported to contribute to tumour cell invasion.^39^ An increase in the plasmatic level of SFN was also reported in HCC.^40^ Although, this increase was not statistically significant in our study, we demonstrated that a high plasmatic level of SFN is an independent factor influencing PDAC recurrence after surgical resection. An external validation using a larger cohort of resected PDAC with a longer follow-up will be required to definitively validate SFN as a clinically relevant biomarker in PDAC, as compared with CA 19-9. The identification of robust prognostic biomarker in PDAC could provide guidance for the treatment schedule. To date, preoperative treatment (e.g. chemotherapy, radiochemotherapy) are only validated for borderline of locally advanced PDAC. The efficiency of this management in PDAC eligible for surgery was only evaluated in the Phase 3 trial PREOPANC, which assessed the interest of a neoadjuvant chemoradiotherapy to increase OS and DFS.^41^ Besides, a randomised controlled trial is actually ongoing (PANACHE, NCT02959879) to evaluate the benefit of FOLFIRINOX neoadjuvant chemotherapy for resectable PDAC. The identification of an aggressive phenotype of PDAC through SFN expression with IHC on biopsy or by blood test could be helpful in preoperative therapeutic management.

In conclusion, by using an unsupervised approach, we showed a clinically relevant association between transcriptomic changes in the stroma and the aggressiveness of PDAC, and we identified SFN as a novel promising candidate prognostic biomarker. This work supports the concept that targeting the microenvironment is a promising strategy in PDAC.

## Supplementary information

Supplementary Figures and Tables

## Data Availability

The data sets generated during and/or analysed during this study are available from the corresponding author on reasonable request.
