# Supplementary material for: Molecular profiling of stroma highlights stratifin as a novel biomarker of poor prognosis in pancreatic ductal adenocarcinoma
Source: Br J Cancer. 2020 May 7;123(1):72–80. doi: 10.1038/s41416-020-0863-1 (PMC7341840; doi:10.1038/s41416-020-0863-1)

**Supporting Table 1 : Clinicopathological features of TMA validating set**

| **Clinicopathological features** | **n=80** |
| --- | --- |
| Age, mean± SD | 65.5±11 |
| Gender , Male : Female | 41:39:00 |
| Tumor size, cm, range ± SD | 3.8 ± 4 |
| Range | janv-28 |
| Tumor size ≥ 2cm | 65 (81.3%) |
| TNM score |  |
| T1-T3 | 22 (27.5%) |
| T3-T4 | 58 (72.5) |
| Differentiation |  |
| Poor | 6 (7.5%) |
| Moderate | 37 (46.25%) |
| Well | 37 (46.25%) |
| Quality of resection |  |
| R0 | 68 (85%) |
| R1-R2 | 12 (15%) |
| Positive lymph nodes | 53 (66.3%) |
| Microvascular invasion | 27 (33.75%) |
| Perineural infiltration | 62 (77.5%) |
| Chronic pancreatitis | 25 (31.25%) |
| Adjuvant chemotherapy | 62 (77.5%) |
| Follow-up, month (mean ± SD) | 30.3 ± 25.8 |
| Range | 3.7 - 113 |
| Death | 65 (81.3%) |

**Supporting Table 2: Clinicopathological features of validating Set 2 (plasmatic samples) including 51 resected PDAC**

| Clinicopathological features | n=51 |
| --- | --- |
| Gender M : F | 35:16:00 |
| Age ± SD | 67.8 ± 9.6 |
| BMI± SD | 24.4 ± 3.9 |
| Surgical procedure |  |
| Pancreatoduodenectomy | 43 (84.3%) |
| Distal pancreatectomy | 8 (15.7%) |
| TNM score |  |
| 01-févr | 7 (13.7%) |
| 03-avr | 44 (86.3%) |
| Tumour size ≥ 2cm | 41 (80.4%) |
| Positive lymph node | 35 (68.6%) |
| Differentiation |  |
| Well | 31 (60.8%) |
| Moderately | 17 (33.3%) |
| Poor | 3 (5.9%) |
| Perineural invasion | 41 (80.4%) |
| Lymphovascular invasion | 32 (62.7%) |
| Chronic pancreatitis | 10 (24.5%) |
| Adjuvant chemotherapy | 46 (90.2%) |
| Median follow up, IQR | 17.2 [15] |
| Death | 12 (23.5%) |
| Serum SFN level (ng/mL), IQR | 0.55 [0.53] |
| SD : Standard deviation |  |
| IQR : Interquartile range |  |

**Supporting Table 3. List of primary antibodies used for IHC analysis**

| Antibody | Dilution | Protocol | Compagny | Reference |
| --- | --- | --- | --- | --- |
| Chemokine (C-X-C motif) ligand 3 (CXCL3) | 1/50 | pH8 at 95° | R&D System | MAB276-SP |
| Stratifin (SFN) | 1/200 | pH6 at 95° | Novus | NBP1-80610 |
| Integrin beta 6 (ITGB6) | 1/200 | pH6 at 95° | Novus | NBP1-86296 |
| A disintegrin and metalloproteinase with thrombospondin motifs 12 (ADAMTS12) | 1/100 | pH8 at 95° | Novus | NBP1-86296 |
| TNF superfamily member 9 (TNFSF9) | 1/100 | pH6 at 95° | R&D Systems | AF2295-SP |
| Cytokeratin 19 (KRT19) | 1/50 | pH8 at 95° | Dako | M0888 |
| Goat anti-MouseHRP | - | - | Ventana | 760-7060 |

**Supporting Table 4 : List of differentially expressed genes in the stroma of PDAC**

| **Parametric p-value** | **FDR** | **Permutation p-value** | **Geom mean intensity in Stroma NT** | **Geom mean intensity in Stroma T** | **Fold-change T/NT** | **Symbol** | **Name** | **Accession ID** | **MapLocation** |
| --- | --- | --- | --- | --- | --- | --- | --- | --- | --- |
| 0.0086227 | 0.142 | 0.047619 | 0.37 | 33.58 | 90.91 | [SLC6A14](http://www.ncbi.nlm.nih.gov/entrez/query.fcgi?cmd=search&db=gene&term=SLC6A14) | solute carrier family 6 (amino acid transporter), member 14 | [NM_007231](http://www.ncbi.nlm.nih.gov/entrez/query.fcgi?db=Nucleotide&term=NM_007231) | hs\|Xq23 |
| 0.0061912 | 0.137 | 0.015873 | 0.2 | 11.35 | 55.56 | [CEACAM5](http://www.ncbi.nlm.nih.gov/entrez/query.fcgi?cmd=search&db=gene&term=CEACAM5) | carcinoembryonic antigen-related cell adhesion molecule 5 | [NM_004363](http://www.ncbi.nlm.nih.gov/entrez/query.fcgi?db=Nucleotide&term=NM_004363) | hs\|19q13.2 |
| 0.0048869 | 0.137 | 0.015873 | 0.17 | 6.48 | 38.46 | [TRIM29](http://www.ncbi.nlm.nih.gov/entrez/query.fcgi?cmd=search&db=gene&term=TRIM29) | tripartite motif containing 29 | [NM_012101](http://www.ncbi.nlm.nih.gov/entrez/query.fcgi?db=Nucleotide&term=NM_012101) | hs\|11q23.3 |
| 0.0038922 | 0.137 | 0.0238095 | 0.13 | 4.39 | 32.26 | [GJB4](http://www.ncbi.nlm.nih.gov/entrez/query.fcgi?cmd=search&db=gene&term=GJB4) | gap junction protein, beta 4, 30.3kDa | [NM_153212](http://www.ncbi.nlm.nih.gov/entrez/query.fcgi?db=Nucleotide&term=NM_153212) | hs\|1p34.3 |
| 0.0003395 | 0.137 | 0.015873 | 0.27 | 6.65 | 24.39 | [TMPRSS4](http://www.ncbi.nlm.nih.gov/entrez/query.fcgi?cmd=search&db=gene&term=TMPRSS4) | transmembrane protease, serine 4 | [NM_001290094](http://www.ncbi.nlm.nih.gov/entrez/query.fcgi?db=Nucleotide&term=NM_001290094) | hs\|11q23.3 |
| 0.0058912 | 0.137 | 0.0238095 | 0.33 | 6.58 | 20 | [PITX1](http://www.ncbi.nlm.nih.gov/entrez/query.fcgi?cmd=search&db=gene&term=PITX1) | paired-like homeodomain 1 | [NM_002653](http://www.ncbi.nlm.nih.gov/entrez/query.fcgi?db=Nucleotide&term=NM_002653) | hs\|5q31.1 |
| 0.0003166 | 0.137 | 0.0079365 | 0.25 | 5.02 | 20 | [TRIM31](http://www.ncbi.nlm.nih.gov/entrez/query.fcgi?cmd=search&db=gene&term=TRIM31) | tripartite motif containing 31 | [NM_007028](http://www.ncbi.nlm.nih.gov/entrez/query.fcgi?db=Nucleotide&term=NM_007028) | hs\|6p22.1 |
| 0.006162 | 0.137 | 0.0238095 | 0.62 | 11.84 | 18.87 |  |  |  | hs\|Xq23 |
| 0.0042064 | 0.137 | 0.0238095 | 0.18 | 3.2 | 18.18 | [DLX5](http://www.ncbi.nlm.nih.gov/entrez/query.fcgi?cmd=search&db=gene&term=DLX5) | distal-less homeobox 5 | [NM_005221](http://www.ncbi.nlm.nih.gov/entrez/query.fcgi?db=Nucleotide&term=NM_005221) | hs\|7q21.3 |
| 0.0080623 | 0.141 | 0.047619 | 0.63 | 11.61 | 18.18 | [lnc-CDKN2A-1](http://www.ncbi.nlm.nih.gov/entrez/query.fcgi?cmd=search&db=gene&term=lnc-CDKN2A-1) | lnc-CDKN2A-1:1 | [BX648047](http://www.ncbi.nlm.nih.gov/entrez/query.fcgi?db=Nucleotide&term=BX648047) | hs\|9p21.3 |
| 2.19E-05 | 0.137 | 0.0079365 | 0.14 | 2.58 | 17.86 | [TINAG](http://www.ncbi.nlm.nih.gov/entrez/query.fcgi?cmd=search&db=gene&term=TINAG) | tubulointerstitial nephritis antigen | [NM_014464](http://www.ncbi.nlm.nih.gov/entrez/query.fcgi?db=Nucleotide&term=NM_014464) | hs\|6p12.1 |
| 0.0001876 | 0.137 | 0.0079365 | 0.34 | 6 | 17.54 | [GABRP](http://www.ncbi.nlm.nih.gov/entrez/query.fcgi?cmd=search&db=gene&term=GABRP) | gamma-aminobutyric acid (GABA) A receptor, pi | [NM_014211](http://www.ncbi.nlm.nih.gov/entrez/query.fcgi?db=Nucleotide&term=NM_014211) | hs\|5q35.1 |
| 0.000147 | 0.137 | 0.0079365 | 0.28 | 4.83 | 16.95 | [TNFSF9](http://www.ncbi.nlm.nih.gov/entrez/query.fcgi?cmd=search&db=gene&term=TNFSF9) | tumor necrosis factor (ligand) superfamily, member 9 | [NM_003811](http://www.ncbi.nlm.nih.gov/entrez/query.fcgi?db=Nucleotide&term=NM_003811) | hs\|19p13.3 |
| 0.0072172 | 0.138 | 0.031746 | 0.62 | 10.28 | 16.67 | [KLK6](http://www.ncbi.nlm.nih.gov/entrez/query.fcgi?cmd=search&db=gene&term=KLK6) | kallikrein-related peptidase 6 | [NM_001012964](http://www.ncbi.nlm.nih.gov/entrez/query.fcgi?db=Nucleotide&term=NM_001012964) | hs\|19q13.41 |
| 0.0027308 | 0.137 | 0.0238095 | 0.4 | 6.47 | 16.39 | [CTSE](http://www.ncbi.nlm.nih.gov/entrez/query.fcgi?cmd=search&db=gene&term=CTSE) | cathepsin E | [NM_001910](http://www.ncbi.nlm.nih.gov/entrez/query.fcgi?db=Nucleotide&term=NM_001910) | hs\|1q32.1 |
| 0.0008537 | 0.137 | 0.0238095 | 0.13 | 2.04 | 16.13 | [lnc-FANK1-3](http://www.ncbi.nlm.nih.gov/entrez/query.fcgi?cmd=search&db=gene&term=lnc-FANK1-3) | lnc-FANK1-3:1 |  | hs\|10q26.2 |
| 0.001162 | 0.137 | 0.015873 | 0.22 | 3.36 | 15.15 | [GJB3](http://www.ncbi.nlm.nih.gov/entrez/query.fcgi?cmd=search&db=gene&term=GJB3) | gap junction protein, beta 3, 31kDa | [NM_024009](http://www.ncbi.nlm.nih.gov/entrez/query.fcgi?db=Nucleotide&term=NM_024009) | hs\|1p34.3 |
| 0.002718 | 0.137 | 0.0238095 | 0.21 | 3.09 | 14.93 | [LOC102723721](http://www.ncbi.nlm.nih.gov/entrez/query.fcgi?cmd=search&db=gene&term=LOC102723721) | uncharacterized LOC102723721 | [XR_424100](http://www.ncbi.nlm.nih.gov/entrez/query.fcgi?db=Nucleotide&term=XR_424100) | hs\|10p14 |
| 0.0002591 | 0.137 | 0.0079365 | 0.28 | 4.16 | 14.71 | [CEACAM3](http://www.ncbi.nlm.nih.gov/entrez/query.fcgi?cmd=search&db=gene&term=CEACAM3) | carcinoembryonic antigen-related cell adhesion molecule 3 | [NM_001277163](http://www.ncbi.nlm.nih.gov/entrez/query.fcgi?db=Nucleotide&term=NM_001277163) | hs\|19q13.2 |
| 0.0014648 | 0.137 | 0.0079365 | 0.18 | 2.61 | 14.71 | [CEACAM6](http://www.ncbi.nlm.nih.gov/entrez/query.fcgi?cmd=search&db=gene&term=CEACAM6) | carcinoembryonic antigen-related cell adhesion molecule 6 (non-specific cross reacting antigen) | [NM_002483](http://www.ncbi.nlm.nih.gov/entrez/query.fcgi?db=Nucleotide&term=NM_002483) | hs\|19q13.2 |
| 0.001602 | 0.137 | 0.0079365 | 0.33 | 4.52 | 13.7 | [CCL20](http://www.ncbi.nlm.nih.gov/entrez/query.fcgi?cmd=search&db=gene&term=CCL20) | chemokine (C-C motif) ligand 20 | [NM_004591](http://www.ncbi.nlm.nih.gov/entrez/query.fcgi?db=Nucleotide&term=NM_004591) | hs\|2q36.3 |
| 0.0058955 | 0.137 | 0.0238095 | 0.48 | 6.47 | 13.51 | [KRT16P2](http://www.ncbi.nlm.nih.gov/entrez/query.fcgi?cmd=search&db=gene&term=KRT16P2) | keratin 16 pseudogene 2 | [NR_029392](http://www.ncbi.nlm.nih.gov/entrez/query.fcgi?db=Nucleotide&term=NR_029392) | hs\|17p11.2 |
| 0.0013628 | 0.137 | 0.0079365 | 0.21 | 2.79 | 13.33 | [CXCL10](http://www.ncbi.nlm.nih.gov/entrez/query.fcgi?cmd=search&db=gene&term=CXCL10) | chemokine (C-X-C motif) ligand 10 | [NM_001565](http://www.ncbi.nlm.nih.gov/entrez/query.fcgi?db=Nucleotide&term=NM_001565) | hs\|4q21.1 |
| 0.0030396 | 0.137 | 0.015873 | 0.22 | 2.82 | 12.99 | [STRA6](http://www.ncbi.nlm.nih.gov/entrez/query.fcgi?cmd=search&db=gene&term=STRA6) | stimulated by retinoic acid 6 | [NM_001199042](http://www.ncbi.nlm.nih.gov/entrez/query.fcgi?db=Nucleotide&term=NM_001199042) | hs\|15q24.1 |
| 0.0067031 | 0.137 | 0.0238095 | 0.44 | 5.59 | 12.82 | [GJB2](http://www.ncbi.nlm.nih.gov/entrez/query.fcgi?cmd=search&db=gene&term=GJB2) | gap junction protein, beta 2, 26kDa | [NM_004004](http://www.ncbi.nlm.nih.gov/entrez/query.fcgi?db=Nucleotide&term=NM_004004) | hs\|13q12.11 |
| 0.0084993 | 0.141 | 0.0238095 | 0.4 | 5.09 | 12.66 | [FGFBP1](http://www.ncbi.nlm.nih.gov/entrez/query.fcgi?cmd=search&db=gene&term=FGFBP1) | fibroblast growth factor binding protein 1 | [NM_005130](http://www.ncbi.nlm.nih.gov/entrez/query.fcgi?db=Nucleotide&term=NM_005130) | hs\|4p15.32 |
| 0.0013593 | 0.137 | 0.015873 | 0.19 | 2.38 | 12.66 | [SHISA2](http://www.ncbi.nlm.nih.gov/entrez/query.fcgi?cmd=search&db=gene&term=SHISA2) | shisa family member 2 | [NM_001007538](http://www.ncbi.nlm.nih.gov/entrez/query.fcgi?db=Nucleotide&term=NM_001007538) | hs\|13q12.13 |
| 0.004569 | 0.137 | 0.015873 | 0.29 | 3.66 | 12.5 | [GPRC5A](http://www.ncbi.nlm.nih.gov/entrez/query.fcgi?cmd=search&db=gene&term=GPRC5A) | G protein-coupled receptor, class C, group 5, member A | [NM_003979](http://www.ncbi.nlm.nih.gov/entrez/query.fcgi?db=Nucleotide&term=NM_003979) | hs\|12p13.1 |
| 0.0099291 | 0.144 | 0.0396825 | 0.42 | 5.12 | 12.2 | [lnc-PRICKLE2-3](http://www.ncbi.nlm.nih.gov/entrez/query.fcgi?cmd=search&db=gene&term=lnc-PRICKLE2-3) | lnc-PRICKLE2-3:1 |  | hs\|3p14.1 |
| 0.0013806 | 0.137 | 0.015873 | 0.27 | 3.23 | 12.05 | [ANKRD30BP2](http://www.ncbi.nlm.nih.gov/entrez/query.fcgi?cmd=search&db=gene&term=ANKRD30BP2) | ankyrin repeat domain 30B pseudogene 2 | [NR_026916](http://www.ncbi.nlm.nih.gov/entrez/query.fcgi?db=Nucleotide&term=NR_026916) | hs\|21q11.2 |
| 0.0088986 | 0.142 | 0.015873 | 0.24 | 2.87 | 11.9 | [CXCL3](http://www.ncbi.nlm.nih.gov/entrez/query.fcgi?cmd=search&db=gene&term=CXCL3) | chemokine (C-X-C motif) ligand 3 | [NM_002090](http://www.ncbi.nlm.nih.gov/entrez/query.fcgi?db=Nucleotide&term=NM_002090) | hs\|4q13.3 |
| 0.0029411 | 0.137 | 0.015873 | 0.38 | 4.39 | 11.63 | [SFN](http://www.ncbi.nlm.nih.gov/entrez/query.fcgi?cmd=search&db=gene&term=SFN) | stratifin | [NM_006142](http://www.ncbi.nlm.nih.gov/entrez/query.fcgi?db=Nucleotide&term=NM_006142) | hs\|1p36.11 |
| 0.0006574 | 0.137 | 0.0079365 | 0.38 | 4.27 | 11.36 | [C2orf54](http://www.ncbi.nlm.nih.gov/entrez/query.fcgi?cmd=search&db=gene&term=C2orf54) | chromosome 2 open reading frame 54 | [NM_024861](http://www.ncbi.nlm.nih.gov/entrez/query.fcgi?db=Nucleotide&term=NM_024861) | hs\|2q37.3 |
| 0.0019709 | 0.137 | 0.0079365 | 0.3 | 3.38 | 11.36 | [CEACAM3](http://www.ncbi.nlm.nih.gov/entrez/query.fcgi?cmd=search&db=gene&term=CEACAM3) | carcinoembryonic antigen-related cell adhesion molecule 3 | [NM_001815](http://www.ncbi.nlm.nih.gov/entrez/query.fcgi?db=Nucleotide&term=NM_001815) | hs\|19q13.2 |
| 1.08E-05 | 0.137 | 0.0079365 | 0.28 | 3.13 | 11.36 | [HOXA10](http://www.ncbi.nlm.nih.gov/entrez/query.fcgi?cmd=search&db=gene&term=HOXA10) | homeobox A10 | [NM_018951](http://www.ncbi.nlm.nih.gov/entrez/query.fcgi?db=Nucleotide&term=NM_018951) | hs\|7p15.2 |
| 0.0025298 | 0.137 | 0.015873 | 0.32 | 3.66 | 11.36 | [LAMC2](http://www.ncbi.nlm.nih.gov/entrez/query.fcgi?cmd=search&db=gene&term=LAMC2) | laminin, gamma 2 | [NM_018891](http://www.ncbi.nlm.nih.gov/entrez/query.fcgi?db=Nucleotide&term=NM_018891) | hs\|1q25.3 |
| 0.003555 | 0.137 | 0.015873 | 0.19 | 2.16 | 11.11 | [GCNT3](http://www.ncbi.nlm.nih.gov/entrez/query.fcgi?cmd=search&db=gene&term=GCNT3) | glucosaminyl (N-acetyl) transferase 3, mucin type | [NM_004751](http://www.ncbi.nlm.nih.gov/entrez/query.fcgi?db=Nucleotide&term=NM_004751) | hs\|15q22.2 |
| 0.0055785 | 0.137 | 0.0238095 | 0.48 | 5.25 | 10.87 | [LAMC2](http://www.ncbi.nlm.nih.gov/entrez/query.fcgi?cmd=search&db=gene&term=LAMC2) | laminin, gamma 2 | [NM_005562](http://www.ncbi.nlm.nih.gov/entrez/query.fcgi?db=Nucleotide&term=NM_005562) | hs\|1q25.3 |
| 0.008331 | 0.141 | 0.047619 | 0.52 | 5.48 | 10.42 | [DQX1](http://www.ncbi.nlm.nih.gov/entrez/query.fcgi?cmd=search&db=gene&term=DQX1) | DEAQ box RNA-dependent ATPase 1 | [NM_133637](http://www.ncbi.nlm.nih.gov/entrez/query.fcgi?db=Nucleotide&term=NM_133637) | hs\|2p13.1 |
| 0.0066611 | 0.137 | 0.0238095 | 0.3 | 2.97 | 10.1 | [MUC13](http://www.ncbi.nlm.nih.gov/entrez/query.fcgi?cmd=search&db=gene&term=MUC13) | mucin 13, cell surface associated | [NM_033049](http://www.ncbi.nlm.nih.gov/entrez/query.fcgi?db=Nucleotide&term=NM_033049) | hs\|3q21.2 |
| 0.0016361 | 0.137 | 0.0079365 | 0.47 | 4.61 | 10 | [HRASLS2](http://www.ncbi.nlm.nih.gov/entrez/query.fcgi?cmd=search&db=gene&term=HRASLS2) | HRAS-like suppressor 2 | [NM_017878](http://www.ncbi.nlm.nih.gov/entrez/query.fcgi?db=Nucleotide&term=NM_017878) | hs\|11q12.3 |
| 0.0059068 | 0.137 | 0.0079365 | 0.28 | 2.72 | 10 | [IHH](http://www.ncbi.nlm.nih.gov/entrez/query.fcgi?cmd=search&db=gene&term=IHH) | indian hedgehog | [NM_002181](http://www.ncbi.nlm.nih.gov/entrez/query.fcgi?db=Nucleotide&term=NM_002181) | hs\|2q35 |
| 0.0003768 | 0.137 | 0.0079365 | 0.4 | 3.99 | 10 | [LAMB3](http://www.ncbi.nlm.nih.gov/entrez/query.fcgi?cmd=search&db=gene&term=LAMB3) | laminin, beta 3 | [NM_001017402](http://www.ncbi.nlm.nih.gov/entrez/query.fcgi?db=Nucleotide&term=NM_001017402) | hs\|1q32.2 |
| 9.08E-05 | 0.137 | 0.0079365 | 0.23 | 2.22 | 10 | [TRIM15](http://www.ncbi.nlm.nih.gov/entrez/query.fcgi?cmd=search&db=gene&term=TRIM15) | tripartite motif containing 15 | [NM_033229](http://www.ncbi.nlm.nih.gov/entrez/query.fcgi?db=Nucleotide&term=NM_033229) | hs\|6p22.1 |
| 0.007524 | 0.139 | 0.015873 | 0.28 | 2.6 | 9.09 | [C6orf222](http://www.ncbi.nlm.nih.gov/entrez/query.fcgi?cmd=search&db=gene&term=C6orf222) | chromosome 6 open reading frame 222 | [NM_001010903](http://www.ncbi.nlm.nih.gov/entrez/query.fcgi?db=Nucleotide&term=NM_001010903) | hs\|6p21.31 |
| 0.0030976 | 0.137 | 0.0079365 | 0.51 | 4.49 | 9.09 | [SCEL](http://www.ncbi.nlm.nih.gov/entrez/query.fcgi?cmd=search&db=gene&term=SCEL) | sciellin | [NM_144777](http://www.ncbi.nlm.nih.gov/entrez/query.fcgi?db=Nucleotide&term=NM_144777) | hs\|13q22.3 |
| 7.08E-05 | 0.137 | 0.0079365 | 0.38 | 3.66 | 9.09 | [SIM2](http://www.ncbi.nlm.nih.gov/entrez/query.fcgi?cmd=search&db=gene&term=SIM2) | single-minded family bHLH transcription factor 2 | [NM_005069](http://www.ncbi.nlm.nih.gov/entrez/query.fcgi?db=Nucleotide&term=NM_005069) | hs\|21q22.13 |
| 0.0002817 | 0.137 | 0.0079365 | 0.34 | 3.1 | 9.09 | [TMEM45B](http://www.ncbi.nlm.nih.gov/entrez/query.fcgi?cmd=search&db=gene&term=TMEM45B) | transmembrane protein 45B | [NM_138788](http://www.ncbi.nlm.nih.gov/entrez/query.fcgi?db=Nucleotide&term=NM_138788) | hs\|11q24.3 |
| 0.0026809 | 0.137 | 0.0079365 | 0.45 | 4.16 | 9.09 | [UBD](http://www.ncbi.nlm.nih.gov/entrez/query.fcgi?cmd=search&db=gene&term=UBD) | ubiquitin D | [NM_006398](http://www.ncbi.nlm.nih.gov/entrez/query.fcgi?db=Nucleotide&term=NM_006398) | hs\|6p22.1 |
| 0.0077045 | 0.14 | 0.031746 | 0.28 | 2.29 | 8.33 | [ANLN](http://www.ncbi.nlm.nih.gov/entrez/query.fcgi?cmd=search&db=gene&term=ANLN) | anillin, actin binding protein | [NM_018685](http://www.ncbi.nlm.nih.gov/entrez/query.fcgi?db=Nucleotide&term=NM_018685) | hs\|7p14.2 |
| 0.0049569 | 0.137 | 0.0238095 | 0.47 | 3.8 | 8.33 | [APOC2](http://www.ncbi.nlm.nih.gov/entrez/query.fcgi?cmd=search&db=gene&term=APOC2) | apolipoprotein C-II | [NM_000483](http://www.ncbi.nlm.nih.gov/entrez/query.fcgi?db=Nucleotide&term=NM_000483) | hs\|19q13.32 |
| 0.0015219 | 0.137 | 0.0079365 | 0.33 | 2.48 | 7.69 | [CXCL1](http://www.ncbi.nlm.nih.gov/entrez/query.fcgi?cmd=search&db=gene&term=CXCL1) | chemokine (C-X-C motif) ligand 1 (melanoma growth stimulating activity, alpha) | [NM_001511](http://www.ncbi.nlm.nih.gov/entrez/query.fcgi?db=Nucleotide&term=NM_001511) | hs\|4q13.3 |
| 0.0033146 | 0.137 | 0.015873 | 0.26 | 2.08 | 7.69 | [lnc-NFYB-1](http://www.ncbi.nlm.nih.gov/entrez/query.fcgi?cmd=search&db=gene&term=lnc-NFYB-1) | lnc-NFYB-1:1 |  | hs\|12q23.3 |
| 0.0033416 | 0.137 | 0.0079365 | 0.38 | 2.85 | 7.69 | [ZG16B](http://www.ncbi.nlm.nih.gov/entrez/query.fcgi?cmd=search&db=gene&term=ZG16B) | zymogen granule protein 16B | [NM_145252](http://www.ncbi.nlm.nih.gov/entrez/query.fcgi?db=Nucleotide&term=NM_145252) | hs\|16p13.3 |
| 0.0008838 | 0.137 | 0.0079365 | 0.55 | 3.9 | 7.14 | [C4BPB](http://www.ncbi.nlm.nih.gov/entrez/query.fcgi?cmd=search&db=gene&term=C4BPB) | complement component 4 binding protein, beta | [NM_000716](http://www.ncbi.nlm.nih.gov/entrez/query.fcgi?db=Nucleotide&term=NM_000716) | hs\|1q32.2 |
| 0.0035726 | 0.137 | 0.015873 | 0.29 | 2.05 | 7.14 | [EGR4](http://www.ncbi.nlm.nih.gov/entrez/query.fcgi?cmd=search&db=gene&term=EGR4) | early growth response 4 | [NM_001965](http://www.ncbi.nlm.nih.gov/entrez/query.fcgi?db=Nucleotide&term=NM_001965) | hs\|2p13.1 |
| 0.008839 | 0.142 | 0.031746 | 0.48 | 3.49 | 7.14 | [KIF26B](http://www.ncbi.nlm.nih.gov/entrez/query.fcgi?cmd=search&db=gene&term=KIF26B) | kinesin family member 26B | [NM_018012](http://www.ncbi.nlm.nih.gov/entrez/query.fcgi?db=Nucleotide&term=NM_018012) | hs\|1q44 |
| 0.0066963 | 0.137 | 0.015873 | 0.36 | 2.6 | 7.14 | [OLR1](http://www.ncbi.nlm.nih.gov/entrez/query.fcgi?cmd=search&db=gene&term=OLR1) | oxidized low density lipoprotein (lectin-like) receptor 1 | [NM_002543](http://www.ncbi.nlm.nih.gov/entrez/query.fcgi?db=Nucleotide&term=NM_002543) | hs\|12p13.2 |
| 0.0009126 | 0.137 | 0.0079365 | 0.49 | 3.5 | 7.14 | [SH3D21](http://www.ncbi.nlm.nih.gov/entrez/query.fcgi?cmd=search&db=gene&term=SH3D21) | SH3 domain containing 21 | [NM_001162530](http://www.ncbi.nlm.nih.gov/entrez/query.fcgi?db=Nucleotide&term=NM_001162530) | hs\|1p34.3 |
| 0.001847 | 0.137 | 0.0079365 | 0.57 | 4.12 | 7.14 | [XDH](http://www.ncbi.nlm.nih.gov/entrez/query.fcgi?cmd=search&db=gene&term=XDH) | xanthine dehydrogenase | [NM_000379](http://www.ncbi.nlm.nih.gov/entrez/query.fcgi?db=Nucleotide&term=NM_000379) | hs\|2p23.1 |
| 0.0017779 | 0.137 | 0.0238095 | 0.52 | 3.64 | 7.14 | [XLOC_l2_014209](http://www.ncbi.nlm.nih.gov/entrez/query.fcgi?cmd=search&db=gene&term=XLOC_l2_014209) |  |  | hs\|8q12.1 |
| 0.0060854 | 0.137 | 0.015873 | 0.35 | 2.48 | 7.14 |  |  |  | hs\|2q35 |
| 0.0016618 | 0.137 | 0.0079365 | 0.31 | 2.13 | 6.67 | [CEACAM7](http://www.ncbi.nlm.nih.gov/entrez/query.fcgi?cmd=search&db=gene&term=CEACAM7) | carcinoembryonic antigen-related cell adhesion molecule 7 | [NM_006890](http://www.ncbi.nlm.nih.gov/entrez/query.fcgi?db=Nucleotide&term=NM_006890) | hs\|19q13.2 |
| 9.48E-05 | 0.137 | 0.0079365 | 0.48 | 3.12 | 6.67 | [ITGB6](http://www.ncbi.nlm.nih.gov/entrez/query.fcgi?cmd=search&db=gene&term=ITGB6) | integrin, beta 6 | [NM_000888](http://www.ncbi.nlm.nih.gov/entrez/query.fcgi?db=Nucleotide&term=NM_000888) | hs\|2q24.2 |
| 0.0078491 | 0.14 | 0.0238095 | 0.26 | 1.7 | 6.67 | [LAMP5](http://www.ncbi.nlm.nih.gov/entrez/query.fcgi?cmd=search&db=gene&term=LAMP5) | lysosomal-associated membrane protein family, member 5 | [NM_012261](http://www.ncbi.nlm.nih.gov/entrez/query.fcgi?db=Nucleotide&term=NM_012261) | hs\|20p12.2 |
| 0.0046434 | 0.137 | 0.031746 | 0.26 | 1.78 | 6.67 | [LOC101927650](http://www.ncbi.nlm.nih.gov/entrez/query.fcgi?cmd=search&db=gene&term=LOC101927650) | uncharacterized LOC101927650 | [NR_110918](http://www.ncbi.nlm.nih.gov/entrez/query.fcgi?db=Nucleotide&term=NR_110918) | hs\|16q21 |
| 0.0045894 | 0.137 | 0.015873 | 0.36 | 2.42 | 6.67 | [MIR31HG](http://www.ncbi.nlm.nih.gov/entrez/query.fcgi?cmd=search&db=gene&term=MIR31HG) | MIR31 host gene (non-protein coding) | [NR_027054](http://www.ncbi.nlm.nih.gov/entrez/query.fcgi?db=Nucleotide&term=NR_027054) | hs\|9p21.3 |
| 0.0005252 | 0.137 | 0.0079365 | 0.54 | 3.54 | 6.67 | [TSPAN1](http://www.ncbi.nlm.nih.gov/entrez/query.fcgi?cmd=search&db=gene&term=TSPAN1) | tetraspanin 1 | [NM_005727](http://www.ncbi.nlm.nih.gov/entrez/query.fcgi?db=Nucleotide&term=NM_005727) | hs\|1p34.1 |
| 0.0025094 | 0.137 | 0.0079365 | 0.61 | 3.7 | 6.25 | [AIM1L](http://www.ncbi.nlm.nih.gov/entrez/query.fcgi?cmd=search&db=gene&term=AIM1L) | absent in melanoma 1-like | [NM_001039775](http://www.ncbi.nlm.nih.gov/entrez/query.fcgi?db=Nucleotide&term=NM_001039775) | hs\|1p36.11 |
| 0.0007219 | 0.137 | 0.0079365 | 0.41 | 2.65 | 6.25 | [AOC1](http://www.ncbi.nlm.nih.gov/entrez/query.fcgi?cmd=search&db=gene&term=AOC1) | amine oxidase, copper containing 1 | [NM_001091](http://www.ncbi.nlm.nih.gov/entrez/query.fcgi?db=Nucleotide&term=NM_001091) | hs\|7q36.1 |
| 0.0017403 | 0.137 | 0.0079365 | 0.42 | 2.57 | 6.25 | [CXCL10](http://www.ncbi.nlm.nih.gov/entrez/query.fcgi?cmd=search&db=gene&term=CXCL10) | chemokine (C-X-C motif) ligand 10 | [NM_001565](http://www.ncbi.nlm.nih.gov/entrez/query.fcgi?db=Nucleotide&term=NM_001565) | hs\|4q21.1 |
| 0.0004342 | 0.137 | 0.0079365 | 0.44 | 2.75 | 6.25 | [GALNT5](http://www.ncbi.nlm.nih.gov/entrez/query.fcgi?cmd=search&db=gene&term=GALNT5) | polypeptide N-acetylgalactosaminyltransferase 5 | [NM_014568](http://www.ncbi.nlm.nih.gov/entrez/query.fcgi?db=Nucleotide&term=NM_014568) | hs\|2q24.1 |
| 0.0006637 | 0.137 | 0.0079365 | 0.43 | 2.66 | 6.25 | [KRT19P2](http://www.ncbi.nlm.nih.gov/entrez/query.fcgi?cmd=search&db=gene&term=KRT19P2) | keratin 19 pseudogene 2 | [NR_036685](http://www.ncbi.nlm.nih.gov/entrez/query.fcgi?db=Nucleotide&term=NR_036685) | hs\|12q22 |
| 0.0014464 | 0.137 | 0.0079365 | 0.34 | 2.07 | 6.25 | [lnc-NCOA3-2](http://www.ncbi.nlm.nih.gov/entrez/query.fcgi?cmd=search&db=gene&term=lnc-NCOA3-2) | lnc-NCOA3-2:1 |  | hs\|20q13.13 |
| 1.86E-05 | 0.137 | 0.0079365 | 0.36 | 2.19 | 6.25 | [S100A14](http://www.ncbi.nlm.nih.gov/entrez/query.fcgi?cmd=search&db=gene&term=S100A14) | S100 calcium binding protein A14 | [NM_020672](http://www.ncbi.nlm.nih.gov/entrez/query.fcgi?db=Nucleotide&term=NM_020672) | hs\|1q21.3 |
| 0.0063109 | 0.137 | 0.0238095 | 0.35 | 2.03 | 5.88 | [CYP4F2](http://www.ncbi.nlm.nih.gov/entrez/query.fcgi?cmd=search&db=gene&term=CYP4F2) | cytochrome P450, family 4, subfamily F, polypeptide 2 | [NM_001082](http://www.ncbi.nlm.nih.gov/entrez/query.fcgi?db=Nucleotide&term=NM_001082) | hs\|19p13.12 |
| 0.0010461 | 0.137 | 0.0079365 | 0.53 | 3.19 | 5.88 | [IL1RN](http://www.ncbi.nlm.nih.gov/entrez/query.fcgi?cmd=search&db=gene&term=IL1RN) | interleukin 1 receptor antagonist | [NM_173843](http://www.ncbi.nlm.nih.gov/entrez/query.fcgi?db=Nucleotide&term=NM_173843) | hs\|2q13 |
| 0.0072168 | 0.138 | 0.015873 | 0.37 | 2.2 | 5.88 | [NKX3-2](http://www.ncbi.nlm.nih.gov/entrez/query.fcgi?cmd=search&db=gene&term=NKX3-2) | NK3 homeobox 2 | [NM_001189](http://www.ncbi.nlm.nih.gov/entrez/query.fcgi?db=Nucleotide&term=NM_001189) | hs\|4p15.33 |
| 0.0036066 | 0.137 | 0.015873 | 0.39 | 2.33 | 5.88 | [RHBDL2](http://www.ncbi.nlm.nih.gov/entrez/query.fcgi?cmd=search&db=gene&term=RHBDL2) | rhomboid, veinlet-like 2 (Drosophila) | [NM_017821](http://www.ncbi.nlm.nih.gov/entrez/query.fcgi?db=Nucleotide&term=NM_017821) | hs\|1p34.3 |
| 0.0087833 | 0.142 | 0.0238095 | 0.47 | 2.78 | 5.88 | [TNFAIP6](http://www.ncbi.nlm.nih.gov/entrez/query.fcgi?cmd=search&db=gene&term=TNFAIP6) | tumor necrosis factor, alpha-induced protein 6 | [NM_007115](http://www.ncbi.nlm.nih.gov/entrez/query.fcgi?db=Nucleotide&term=NM_007115) | hs\|2q23.3 |
| 0.0022474 | 0.137 | 0.0079365 | 0.31 | 1.87 | 5.88 | [TPRG1](http://www.ncbi.nlm.nih.gov/entrez/query.fcgi?cmd=search&db=gene&term=TPRG1) | tumor protein p63 regulated 1 | [NM_198485](http://www.ncbi.nlm.nih.gov/entrez/query.fcgi?db=Nucleotide&term=NM_198485) | hs\|3q28 |
| 0.0031168 | 0.137 | 0.015873 | 0.35 | 1.94 | 5.56 | [INHBA](http://www.ncbi.nlm.nih.gov/entrez/query.fcgi?cmd=search&db=gene&term=INHBA) | inhibin, beta A | [NM_002192](http://www.ncbi.nlm.nih.gov/entrez/query.fcgi?db=Nucleotide&term=NM_002192) | hs\|7p14.1 |
| 0.0018267 | 0.137 | 0.0079365 | 0.45 | 2.51 | 5.56 | [KCNN4](http://www.ncbi.nlm.nih.gov/entrez/query.fcgi?cmd=search&db=gene&term=KCNN4) | potassium channel, calcium activated intermediate/small conductance subfamily N alpha, member 4 | [NM_002250](http://www.ncbi.nlm.nih.gov/entrez/query.fcgi?db=Nucleotide&term=NM_002250) | hs\|19q13.31 |
| 0.0060902 | 0.137 | 0.0238095 | 0.48 | 2.6 | 5.56 | [NTM](http://www.ncbi.nlm.nih.gov/entrez/query.fcgi?cmd=search&db=gene&term=NTM) | neurotrimin | [NM_001144058](http://www.ncbi.nlm.nih.gov/entrez/query.fcgi?db=Nucleotide&term=NM_001144058) | hs\|11q25 |
| 0.0013779 | 0.137 | 0.0079365 | 0.54 | 2.9 | 5.56 | [RHBDL2](http://www.ncbi.nlm.nih.gov/entrez/query.fcgi?cmd=search&db=gene&term=RHBDL2) | rhomboid, veinlet-like 2 (Drosophila) | [NM_017821](http://www.ncbi.nlm.nih.gov/entrez/query.fcgi?db=Nucleotide&term=NM_017821) | hs\|1p34.3 |
| 0.0010635 | 0.137 | 0.0079365 | 0.48 | 2.57 | 5.26 | [KRT19](http://www.ncbi.nlm.nih.gov/entrez/query.fcgi?cmd=search&db=gene&term=KRT19) | keratin 19, type I | [NM_002276](http://www.ncbi.nlm.nih.gov/entrez/query.fcgi?db=Nucleotide&term=NM_002276) | hs\|17q21.2 |
| 0.0068363 | 0.137 | 0.0238095 | 0.43 | 2.23 | 5.26 | [MMP1](http://www.ncbi.nlm.nih.gov/entrez/query.fcgi?cmd=search&db=gene&term=MMP1) | matrix metallopeptidase 1 (interstitial collagenase) | [NM_002421](http://www.ncbi.nlm.nih.gov/entrez/query.fcgi?db=Nucleotide&term=NM_002421) | hs\|11q22.2 |
| 0.0017659 | 0.137 | 0.015873 | 0.55 | 2.93 | 5.26 | [RHBDL2](http://www.ncbi.nlm.nih.gov/entrez/query.fcgi?cmd=search&db=gene&term=RHBDL2) | rhomboid, veinlet-like 2 (Drosophila) | [NM_017821](http://www.ncbi.nlm.nih.gov/entrez/query.fcgi?db=Nucleotide&term=NM_017821) | hs\|1p34.3 |
| 0.0009723 | 0.137 | 0.0079365 | 0.36 | 1.86 | 5 | [FUT2](http://www.ncbi.nlm.nih.gov/entrez/query.fcgi?cmd=search&db=gene&term=FUT2) | fucosyltransferase 2 (secretor status included) | [NM_000511](http://www.ncbi.nlm.nih.gov/entrez/query.fcgi?db=Nucleotide&term=NM_000511) | hs\|19q13.33 |
| 0.0052443 | 0.137 | 0.0238095 | 0.39 | 1.96 | 5 | [HOPX](http://www.ncbi.nlm.nih.gov/entrez/query.fcgi?cmd=search&db=gene&term=HOPX) | HOP homeobox | [NM_139211](http://www.ncbi.nlm.nih.gov/entrez/query.fcgi?db=Nucleotide&term=NM_139211) | hs\|4q12 |
| 9.63E-05 | 0.137 | 0.0079365 | 0.34 | 1.71 | 5 | [LRP8](http://www.ncbi.nlm.nih.gov/entrez/query.fcgi?cmd=search&db=gene&term=LRP8) | low density lipoprotein receptor-related protein 8, apolipoprotein e receptor | [NM_033300](http://www.ncbi.nlm.nih.gov/entrez/query.fcgi?db=Nucleotide&term=NM_033300) | hs\|1p32.3 |
| 0.0096684 | 0.144 | 0.015873 | 0.72 | 3.55 | 5 | [SH3D21](http://www.ncbi.nlm.nih.gov/entrez/query.fcgi?cmd=search&db=gene&term=SH3D21) | SH3 domain containing 21 | [AK056459](http://www.ncbi.nlm.nih.gov/entrez/query.fcgi?db=Nucleotide&term=AK056459) | hs\|1p34.3 |
| 0.000627 | 0.137 | 0.0079365 | 0.38 | 1.9 | 5 | [TMC5](http://www.ncbi.nlm.nih.gov/entrez/query.fcgi?cmd=search&db=gene&term=TMC5) | transmembrane channel-like 5 | [NM_024780](http://www.ncbi.nlm.nih.gov/entrez/query.fcgi?db=Nucleotide&term=NM_024780) | hs\|16p12.3 |
| 0.0015572 | 0.137 | 0.015873 | 0.47 | 2.24 | 4.76 | [C19orf33](http://www.ncbi.nlm.nih.gov/entrez/query.fcgi?cmd=search&db=gene&term=C19orf33) | chromosome 19 open reading frame 33 | [NM_033520](http://www.ncbi.nlm.nih.gov/entrez/query.fcgi?db=Nucleotide&term=NM_033520) | hs\|19q13.2 |
| 0.0001535 | 0.137 | 0.0079365 | 0.58 | 2.75 | 4.76 | [CEACAM1](http://www.ncbi.nlm.nih.gov/entrez/query.fcgi?cmd=search&db=gene&term=CEACAM1) | carcinoembryonic antigen-related cell adhesion molecule 1 (biliary glycoprotein) | [NM_001712](http://www.ncbi.nlm.nih.gov/entrez/query.fcgi?db=Nucleotide&term=NM_001712) | hs\|19q13.2 |
| 0.0065306 | 0.137 | 0.031746 | 0.45 | 2.15 | 4.76 | [GDA](http://www.ncbi.nlm.nih.gov/entrez/query.fcgi?cmd=search&db=gene&term=GDA) | guanine deaminase | [NM_004293](http://www.ncbi.nlm.nih.gov/entrez/query.fcgi?db=Nucleotide&term=NM_004293) | hs\|9q21.13 |
| 0.005481 | 0.137 | 0.015873 | 0.55 | 2.58 | 4.76 | [LEF1-AS1](http://www.ncbi.nlm.nih.gov/entrez/query.fcgi?cmd=search&db=gene&term=LEF1-AS1) | LEF1 antisense RNA 1 | [NR_029373](http://www.ncbi.nlm.nih.gov/entrez/query.fcgi?db=Nucleotide&term=NR_029373) | hs\|4q25 |
| 0.0013053 | 0.137 | 0.0079365 | 0.62 | 2.92 | 4.76 | [PTK6](http://www.ncbi.nlm.nih.gov/entrez/query.fcgi?cmd=search&db=gene&term=PTK6) | protein tyrosine kinase 6 | [NM_005975](http://www.ncbi.nlm.nih.gov/entrez/query.fcgi?db=Nucleotide&term=NM_005975) | hs\|20q13.33 |
| 0.0060311 | 0.137 | 0.0079365 | 0.41 | 1.9 | 4.76 | [TMEM92](http://www.ncbi.nlm.nih.gov/entrez/query.fcgi?cmd=search&db=gene&term=TMEM92) | transmembrane protein 92 | [NM_153229](http://www.ncbi.nlm.nih.gov/entrez/query.fcgi?db=Nucleotide&term=NM_153229) | hs\|17q21.33 |
| 0.0059038 | 0.137 | 0.0238095 | 0.43 | 1.9 | 4.55 | [FAP](http://www.ncbi.nlm.nih.gov/entrez/query.fcgi?cmd=search&db=gene&term=FAP) | fibroblast activation protein, alpha | [NM_004460](http://www.ncbi.nlm.nih.gov/entrez/query.fcgi?db=Nucleotide&term=NM_004460) | hs\|2q24.2 |
| 0.0046442 | 0.137 | 0.0238095 | 0.5 | 2.24 | 4.55 | [FOXC2-AS1](http://www.ncbi.nlm.nih.gov/entrez/query.fcgi?cmd=search&db=gene&term=FOXC2-AS1) | FOXC2 antisense RNA 1 | [NR_125795](http://www.ncbi.nlm.nih.gov/entrez/query.fcgi?db=Nucleotide&term=NR_125795) | hs\|16q24.1 |
| 0.0050031 | 0.137 | 0.031746 | 0.43 | 2 | 4.55 | [GPX2](http://www.ncbi.nlm.nih.gov/entrez/query.fcgi?cmd=search&db=gene&term=GPX2) | glutathione peroxidase 2 (gastrointestinal) | [NM_002083](http://www.ncbi.nlm.nih.gov/entrez/query.fcgi?db=Nucleotide&term=NM_002083) | hs\|14q23.3 |
| 0.0041146 | 0.137 | 0.015873 | 0.63 | 2.81 | 4.55 | [HN1](http://www.ncbi.nlm.nih.gov/entrez/query.fcgi?cmd=search&db=gene&term=HN1) | hematological and neurological expressed 1 | [NM_001002033](http://www.ncbi.nlm.nih.gov/entrez/query.fcgi?db=Nucleotide&term=NM_001002033) | hs\|17q25.1 |
| 0.0031834 | 0.137 | 0.0079365 | 0.5 | 2.25 | 4.55 | [SGPP2](http://www.ncbi.nlm.nih.gov/entrez/query.fcgi?cmd=search&db=gene&term=SGPP2) | sphingosine-1-phosphate phosphatase 2 | [XM_005246297](http://www.ncbi.nlm.nih.gov/entrez/query.fcgi?db=Nucleotide&term=XM_005246297) | hs\|2q36.1 |
| 0.0067288 | 0.137 | 0.047619 | 0.82 | 3.72 | 4.55 | [SMCO2](http://www.ncbi.nlm.nih.gov/entrez/query.fcgi?cmd=search&db=gene&term=SMCO2) | single-pass membrane protein with coiled-coil domains 2 | [NM_001145010](http://www.ncbi.nlm.nih.gov/entrez/query.fcgi?db=Nucleotide&term=NM_001145010) | hs\|12p11.23 |
| 0.0094104 | 0.143 | 0.0238095 | 0.62 | 2.65 | 4.35 | [ADAMTS12](http://www.ncbi.nlm.nih.gov/entrez/query.fcgi?cmd=search&db=gene&term=ADAMTS12) | ADAM metallopeptidase with thrombospondin type 1 motif, 12 | [NM_030955](http://www.ncbi.nlm.nih.gov/entrez/query.fcgi?db=Nucleotide&term=NM_030955) | hs\|5p13.3 |
| 0.0069625 | 0.138 | 0.0396825 | 0.54 | 2.33 | 4.35 | [ATP2C2](http://www.ncbi.nlm.nih.gov/entrez/query.fcgi?cmd=search&db=gene&term=ATP2C2) | ATPase, Ca++ transporting, type 2C, member 2 | [NM_014861](http://www.ncbi.nlm.nih.gov/entrez/query.fcgi?db=Nucleotide&term=NM_014861) | hs\|16q24.1 |
| 0.0004023 | 0.137 | 0.0079365 | 0.53 | 2.36 | 4.35 | [BIK](http://www.ncbi.nlm.nih.gov/entrez/query.fcgi?cmd=search&db=gene&term=BIK) | BCL2-interacting killer (apoptosis-inducing) | [NM_001197](http://www.ncbi.nlm.nih.gov/entrez/query.fcgi?db=Nucleotide&term=NM_001197) | hs\|22q13.2 |
| 0.0020179 | 0.137 | 0.0079365 | 0.55 | 2.38 | 4.35 | [CXCL1](http://www.ncbi.nlm.nih.gov/entrez/query.fcgi?cmd=search&db=gene&term=CXCL1) | chemokine (C-X-C motif) ligand 1 (melanoma growth stimulating activity, alpha) | [NM_001511](http://www.ncbi.nlm.nih.gov/entrez/query.fcgi?db=Nucleotide&term=NM_001511) | hs\|4q13.3 |
| 0.0018244 | 0.137 | 0.015873 | 0.58 | 2.53 | 4.35 | [FOXL1](http://www.ncbi.nlm.nih.gov/entrez/query.fcgi?cmd=search&db=gene&term=FOXL1) | forkhead box L1 | [NM_005250](http://www.ncbi.nlm.nih.gov/entrez/query.fcgi?db=Nucleotide&term=NM_005250) | hs\|16q24.1 |
| 0.0043287 | 0.137 | 0.015873 | 0.62 | 2.67 | 4.35 | [HN1](http://www.ncbi.nlm.nih.gov/entrez/query.fcgi?cmd=search&db=gene&term=HN1) | hematological and neurological expressed 1 | [NM_001002032](http://www.ncbi.nlm.nih.gov/entrez/query.fcgi?db=Nucleotide&term=NM_001002032) | hs\|17q25.1 |
| 0.0092441 | 0.143 | 0.015873 | 0.54 | 2.38 | 4.35 | [IL21R](http://www.ncbi.nlm.nih.gov/entrez/query.fcgi?cmd=search&db=gene&term=IL21R) | interleukin 21 receptor | [NM_181078](http://www.ncbi.nlm.nih.gov/entrez/query.fcgi?db=Nucleotide&term=NM_181078) | hs\|16p12.1 |
| 2.55E-05 | 0.137 | 0.0079365 | 0.49 | 2.14 | 4.35 | [RND1](http://www.ncbi.nlm.nih.gov/entrez/query.fcgi?cmd=search&db=gene&term=RND1) | Rho family GTPase 1 | [NM_014470](http://www.ncbi.nlm.nih.gov/entrez/query.fcgi?db=Nucleotide&term=NM_014470) | hs\|12q13.12 |
| 0.0053092 | 0.137 | 0.031746 | 0.4 | 1.75 | 4.35 | [RUNX1](http://www.ncbi.nlm.nih.gov/entrez/query.fcgi?cmd=search&db=gene&term=RUNX1) | runt-related transcription factor 1 | [NM_001122607](http://www.ncbi.nlm.nih.gov/entrez/query.fcgi?db=Nucleotide&term=NM_001122607) | hs\|21q22.12 |
| 0.0064209 | 0.137 | 0.0238095 | 0.72 | 3.09 | 4.35 |  |  |  | hs\|2q31.1 |
| 0.0062263 | 0.137 | 0.0396825 | 0.33 | 1.39 | 4.17 | [ANTXR1](http://www.ncbi.nlm.nih.gov/entrez/query.fcgi?cmd=search&db=gene&term=ANTXR1) | anthrax toxin receptor 1 | [NM_053034](http://www.ncbi.nlm.nih.gov/entrez/query.fcgi?db=Nucleotide&term=NM_053034) | hs\|2p13.3 |
| 0.0045473 | 0.137 | 0.0238095 | 0.55 | 2.28 | 4.17 | [BAIAP2L2](http://www.ncbi.nlm.nih.gov/entrez/query.fcgi?cmd=search&db=gene&term=BAIAP2L2) | BAI1-associated protein 2-like 2 | [NM_025045](http://www.ncbi.nlm.nih.gov/entrez/query.fcgi?db=Nucleotide&term=NM_025045) | hs\|22q13.1 |
| 0.0017065 | 0.137 | 0.015873 | 0.59 | 2.51 | 4.17 | [BIRC3](http://www.ncbi.nlm.nih.gov/entrez/query.fcgi?cmd=search&db=gene&term=BIRC3) | baculoviral IAP repeat containing 3 | [NM_001165](http://www.ncbi.nlm.nih.gov/entrez/query.fcgi?db=Nucleotide&term=NM_001165) | hs\|11q22.2 |
| 0.0053736 | 0.137 | 0.0238095 | 0.4 | 1.67 | 4.17 | [FXYD3](http://www.ncbi.nlm.nih.gov/entrez/query.fcgi?cmd=search&db=gene&term=FXYD3) | FXYD domain containing ion transport regulator 3 | [NM_001136007](http://www.ncbi.nlm.nih.gov/entrez/query.fcgi?db=Nucleotide&term=NM_001136007) | hs\|19q13.12 |
| 0.0002595 | 0.137 | 0.0079365 | 0.6 | 2.47 | 4.17 | [KCNK1](http://www.ncbi.nlm.nih.gov/entrez/query.fcgi?cmd=search&db=gene&term=KCNK1) | potassium channel, two pore domain subfamily K, member 1 | [NM_002245](http://www.ncbi.nlm.nih.gov/entrez/query.fcgi?db=Nucleotide&term=NM_002245) | hs\|1q42.2 |
| 0.0029321 | 0.137 | 0.031746 | 0.71 | 2.98 | 4.17 | [lnc-ARID1B-1](http://www.ncbi.nlm.nih.gov/entrez/query.fcgi?cmd=search&db=gene&term=lnc-ARID1B-1) | lnc-ARID1B-1:1 |  | hs\|6q25.3 |
| 0.0055551 | 0.137 | 0.0079365 | 0.52 | 2.16 | 4.17 | [lnc-NDUFV1-1](http://www.ncbi.nlm.nih.gov/entrez/query.fcgi?cmd=search&db=gene&term=lnc-NDUFV1-1) | lnc-NDUFV1-1:1 | [DA667009](http://www.ncbi.nlm.nih.gov/entrez/query.fcgi?db=Nucleotide&term=DA667009) | hs\|11q13.2 |
| 0.0083468 | 0.141 | 0.031746 | 0.42 | 1.74 | 4.17 | [RUNX1](http://www.ncbi.nlm.nih.gov/entrez/query.fcgi?cmd=search&db=gene&term=RUNX1) | runt-related transcription factor 1 | [NM_001001890](http://www.ncbi.nlm.nih.gov/entrez/query.fcgi?db=Nucleotide&term=NM_001001890) | hs\|21q22.12 |
| 0.0002876 | 0.137 | 0.0079365 | 0.48 | 2.01 | 4.17 | [SGPP2](http://www.ncbi.nlm.nih.gov/entrez/query.fcgi?cmd=search&db=gene&term=SGPP2) | sphingosine-1-phosphate phosphatase 2 | [NM_152386](http://www.ncbi.nlm.nih.gov/entrez/query.fcgi?db=Nucleotide&term=NM_152386) | hs\|2q36.1 |
| 0.0051645 | 0.137 | 0.0238095 | 0.65 | 2.71 | 4.17 | [SLC16A3](http://www.ncbi.nlm.nih.gov/entrez/query.fcgi?cmd=search&db=gene&term=SLC16A3) | solute carrier family 16 (monocarboxylate transporter), member 3 | [NM_001042422](http://www.ncbi.nlm.nih.gov/entrez/query.fcgi?db=Nucleotide&term=NM_001042422) | hs\|17q25.3 |
| 0.0041644 | 0.137 | 0.015873 | 0.63 | 2.56 | 4 | [CAPG](http://www.ncbi.nlm.nih.gov/entrez/query.fcgi?cmd=search&db=gene&term=CAPG) | capping protein (actin filament), gelsolin-like | [NM_001747](http://www.ncbi.nlm.nih.gov/entrez/query.fcgi?db=Nucleotide&term=NM_001747) | hs\|2p11.2 |
| 0.0001037 | 0.137 | 0.0079365 | 0.49 | 1.97 | 4 | [FOXQ1](http://www.ncbi.nlm.nih.gov/entrez/query.fcgi?cmd=search&db=gene&term=FOXQ1) | forkhead box Q1 | [NM_033260](http://www.ncbi.nlm.nih.gov/entrez/query.fcgi?db=Nucleotide&term=NM_033260) | hs\|6p25.3 |
| 0.0088882 | 0.142 | 0.031746 | 0.72 | 2.9 | 4 | [LAMA3](http://www.ncbi.nlm.nih.gov/entrez/query.fcgi?cmd=search&db=gene&term=LAMA3) | laminin, alpha 3 | [NM_198129](http://www.ncbi.nlm.nih.gov/entrez/query.fcgi?db=Nucleotide&term=NM_198129) | hs\|18q11.2 |
| 0.0052229 | 0.137 | 0.0238095 | 0.54 | 2.19 | 4 | [PLAUR](http://www.ncbi.nlm.nih.gov/entrez/query.fcgi?cmd=search&db=gene&term=PLAUR) | plasminogen activator, urokinase receptor | [NM_001005377](http://www.ncbi.nlm.nih.gov/entrez/query.fcgi?db=Nucleotide&term=NM_001005377) | hs\|19q13.31 |
| 0.0036609 | 0.137 | 0.0238095 | 0.49 | 1.94 | 4 |  |  |  | hs\|5q22.2 |
| 0.0073659 | 0.139 | 0.015873 | 0.7 | 2.65 | 3.85 | [CSAG1](http://www.ncbi.nlm.nih.gov/entrez/query.fcgi?cmd=search&db=gene&term=CSAG1) | chondrosarcoma associated gene 1 | [NM_153478](http://www.ncbi.nlm.nih.gov/entrez/query.fcgi?db=Nucleotide&term=NM_153478) | hs\|Xq28 |
| 0.0099833 | 0.144 | 0.031746 | 0.6 | 2.34 | 3.85 | [FXYD3](http://www.ncbi.nlm.nih.gov/entrez/query.fcgi?cmd=search&db=gene&term=FXYD3) | FXYD domain containing ion transport regulator 3 | [NM_001136007](http://www.ncbi.nlm.nih.gov/entrez/query.fcgi?db=Nucleotide&term=NM_001136007) | hs\|19q13.12 |
| 0.0012788 | 0.137 | 0.015873 | 0.41 | 1.59 | 3.85 | [LZTS1](http://www.ncbi.nlm.nih.gov/entrez/query.fcgi?cmd=search&db=gene&term=LZTS1) | leucine zipper, putative tumor suppressor 1 | [NM_021020](http://www.ncbi.nlm.nih.gov/entrez/query.fcgi?db=Nucleotide&term=NM_021020) | hs\|8p21.3 |
| 0.0010504 | 0.137 | 0.015873 | 0.85 | 3.24 | 3.85 | [P2RY2](http://www.ncbi.nlm.nih.gov/entrez/query.fcgi?cmd=search&db=gene&term=P2RY2) | purinergic receptor P2Y, G-protein coupled, 2 | [NM_176072](http://www.ncbi.nlm.nih.gov/entrez/query.fcgi?db=Nucleotide&term=NM_176072) | hs\|11q13.4 |
| 0.0041865 | 0.137 | 0.015873 | 0.66 | 2.52 | 3.85 | [PLEK2](http://www.ncbi.nlm.nih.gov/entrez/query.fcgi?cmd=search&db=gene&term=PLEK2) | pleckstrin 2 | [NM_016445](http://www.ncbi.nlm.nih.gov/entrez/query.fcgi?db=Nucleotide&term=NM_016445) | hs\|14q23.3 |
| 0.0097985 | 0.144 | 0.031746 | 0.53 | 2 | 3.85 | [RASL11B](http://www.ncbi.nlm.nih.gov/entrez/query.fcgi?cmd=search&db=gene&term=RASL11B) | RAS-like, family 11, member B | [NM_023940](http://www.ncbi.nlm.nih.gov/entrez/query.fcgi?db=Nucleotide&term=NM_023940) | hs\|4q12 |
| 0.0039612 | 0.137 | 0.015873 | 0.39 | 1.51 | 3.85 | [RUNX1](http://www.ncbi.nlm.nih.gov/entrez/query.fcgi?cmd=search&db=gene&term=RUNX1) | runt-related transcription factor 1 | [NM_001001890](http://www.ncbi.nlm.nih.gov/entrez/query.fcgi?db=Nucleotide&term=NM_001001890) | hs\|21q22.12 |
| 0.0073296 | 0.139 | 0.015873 | 0.47 | 1.78 | 3.85 |  |  | [XM_005263390](http://www.ncbi.nlm.nih.gov/entrez/query.fcgi?db=Nucleotide&term=XM_005263390) | hs\|4q21.22 |
| 0.0057216 | 0.137 | 0.015873 | 0.58 | 2.16 | 3.7 | [CAPN8](http://www.ncbi.nlm.nih.gov/entrez/query.fcgi?cmd=search&db=gene&term=CAPN8) | calpain 8 |  | hs\|1q41 |
| 0.001906 | 0.137 | 0.015873 | 0.59 | 2.19 | 3.7 | [CLDN23](http://www.ncbi.nlm.nih.gov/entrez/query.fcgi?cmd=search&db=gene&term=CLDN23) | claudin 23 | [NM_194284](http://www.ncbi.nlm.nih.gov/entrez/query.fcgi?db=Nucleotide&term=NM_194284) | hs\|8p23.1 |
| 0.0021775 | 0.137 | 0.0238095 | 0.56 | 2.09 | 3.7 | [EPS8L3](http://www.ncbi.nlm.nih.gov/entrez/query.fcgi?cmd=search&db=gene&term=EPS8L3) | EPS8-like 3 | [NM_139053](http://www.ncbi.nlm.nih.gov/entrez/query.fcgi?db=Nucleotide&term=NM_139053) | hs\|1p13.3 |
| 0.0070066 | 0.138 | 0.0238095 | 0.48 | 1.79 | 3.7 | [FLJ32255](http://www.ncbi.nlm.nih.gov/entrez/query.fcgi?cmd=search&db=gene&term=FLJ32255) | uncharacterized LOC643977 | [NR_104643](http://www.ncbi.nlm.nih.gov/entrez/query.fcgi?db=Nucleotide&term=NR_104643) | hs\|5p12 |
| 0.002255 | 0.137 | 0.0079365 | 0.52 | 1.95 | 3.7 | [MDFI](http://www.ncbi.nlm.nih.gov/entrez/query.fcgi?cmd=search&db=gene&term=MDFI) | MyoD family inhibitor | [NM_001300804](http://www.ncbi.nlm.nih.gov/entrez/query.fcgi?db=Nucleotide&term=NM_001300804) | hs\|6p21.1 |
| 0.0034496 | 0.137 | 0.015873 | 0.61 | 2.24 | 3.7 | [MMP14](http://www.ncbi.nlm.nih.gov/entrez/query.fcgi?cmd=search&db=gene&term=MMP14) | matrix metallopeptidase 14 (membrane-inserted) | [NM_004995](http://www.ncbi.nlm.nih.gov/entrez/query.fcgi?db=Nucleotide&term=NM_004995) | hs\|14q11.2 |
| 0.0045674 | 0.137 | 0.0079365 | 0.44 | 1.68 | 3.7 | [MMP7](http://www.ncbi.nlm.nih.gov/entrez/query.fcgi?cmd=search&db=gene&term=MMP7) | matrix metallopeptidase 7 (matrilysin, uterine) | [NM_002423](http://www.ncbi.nlm.nih.gov/entrez/query.fcgi?db=Nucleotide&term=NM_002423) | hs\|11q22.2 |
| 0.0028711 | 0.137 | 0.0079365 | 0.67 | 2.43 | 3.7 | [PMAIP1](http://www.ncbi.nlm.nih.gov/entrez/query.fcgi?cmd=search&db=gene&term=PMAIP1) | phorbol-12-myristate-13-acetate-induced protein 1 | [NM_021127](http://www.ncbi.nlm.nih.gov/entrez/query.fcgi?db=Nucleotide&term=NM_021127) | hs\|18q21.32 |
| 0.0095232 | 0.143 | 0.031746 | 0.66 | 2.44 | 3.7 | [TMEM154](http://www.ncbi.nlm.nih.gov/entrez/query.fcgi?cmd=search&db=gene&term=TMEM154) | transmembrane protein 154 | [NM_152680](http://www.ncbi.nlm.nih.gov/entrez/query.fcgi?db=Nucleotide&term=NM_152680) | hs\|4q31.3 |
| 0.0018987 | 0.137 | 0.0079365 | 0.79 | 2.81 | 3.57 | [ABHD17C](http://www.ncbi.nlm.nih.gov/entrez/query.fcgi?cmd=search&db=gene&term=ABHD17C) | abhydrolase domain containing 17C | [NM_021214](http://www.ncbi.nlm.nih.gov/entrez/query.fcgi?db=Nucleotide&term=NM_021214) | hs\|15q25.1 |
| 0.0094359 | 0.143 | 0.0238095 | 0.6 | 2.16 | 3.57 | [ASPHD2](http://www.ncbi.nlm.nih.gov/entrez/query.fcgi?cmd=search&db=gene&term=ASPHD2) | aspartate beta-hydroxylase domain containing 2 | [NM_020437](http://www.ncbi.nlm.nih.gov/entrez/query.fcgi?db=Nucleotide&term=NM_020437) | hs\|22q12.1 |
| 0.0032866 | 0.137 | 0.0238095 | 0.54 | 1.95 | 3.57 | [FOXS1](http://www.ncbi.nlm.nih.gov/entrez/query.fcgi?cmd=search&db=gene&term=FOXS1) | forkhead box S1 | [NM_004118](http://www.ncbi.nlm.nih.gov/entrez/query.fcgi?db=Nucleotide&term=NM_004118) | hs\|20q11.21 |
| 0.0026393 | 0.137 | 0.0238095 | 0.71 | 2.57 | 3.57 | [PAQR4](http://www.ncbi.nlm.nih.gov/entrez/query.fcgi?cmd=search&db=gene&term=PAQR4) | progestin and adipoQ receptor family member IV | [NM_152341](http://www.ncbi.nlm.nih.gov/entrez/query.fcgi?db=Nucleotide&term=NM_152341) | hs\|16p13.3 |
| 0.0039723 | 0.137 | 0.015873 | 0.73 | 2.62 | 3.57 | [PHLDA2](http://www.ncbi.nlm.nih.gov/entrez/query.fcgi?cmd=search&db=gene&term=PHLDA2) | pleckstrin homology-like domain, family A, member 2 | [NM_003311](http://www.ncbi.nlm.nih.gov/entrez/query.fcgi?db=Nucleotide&term=NM_003311) | hs\|11p15.4 |
| 0.0020053 | 0.137 | 0.015873 | 0.39 | 1.4 | 3.57 | [PRR5L](http://www.ncbi.nlm.nih.gov/entrez/query.fcgi?cmd=search&db=gene&term=PRR5L) | proline rich 5 like | [NM_024841](http://www.ncbi.nlm.nih.gov/entrez/query.fcgi?db=Nucleotide&term=NM_024841) | hs\|11p12 |
| 0.0024292 | 0.137 | 0.015873 | 0.61 | 2.2 | 3.57 | [STYK1](http://www.ncbi.nlm.nih.gov/entrez/query.fcgi?cmd=search&db=gene&term=STYK1) | serine/threonine/tyrosine kinase 1 | [NM_018423](http://www.ncbi.nlm.nih.gov/entrez/query.fcgi?db=Nucleotide&term=NM_018423) | hs\|12p13.2 |
| 0.0015296 | 0.137 | 0.0079365 | 0.55 | 1.95 | 3.57 | [USP30-AS1](http://www.ncbi.nlm.nih.gov/entrez/query.fcgi?cmd=search&db=gene&term=USP30-AS1) | USP30 antisense RNA 1 | [NR_038996](http://www.ncbi.nlm.nih.gov/entrez/query.fcgi?db=Nucleotide&term=NR_038996) | hs\|12q24.11 |
| 0.0079002 | 0.14 | 0.0079365 | 0.44 | 1.56 | 3.57 | [XLOC_l2_014579](http://www.ncbi.nlm.nih.gov/entrez/query.fcgi?cmd=search&db=gene&term=XLOC_l2_014579) |  |  | hs\|8q21.3 |
| 0.0006005 | 0.137 | 0.0079365 | 0.72 | 2.53 | 3.57 |  |  |  | hs\|2p16.1 |
| 0.0021538 | 0.137 | 0.0079365 | 0.56 | 1.93 | 3.45 | [C1orf106](http://www.ncbi.nlm.nih.gov/entrez/query.fcgi?cmd=search&db=gene&term=C1orf106) | chromosome 1 open reading frame 106 | [NM_018265](http://www.ncbi.nlm.nih.gov/entrez/query.fcgi?db=Nucleotide&term=NM_018265) | hs\|1q32.1 |
| 0.0009422 | 0.137 | 0.0079365 | 0.61 | 2.07 | 3.45 | [CEACAM1](http://www.ncbi.nlm.nih.gov/entrez/query.fcgi?cmd=search&db=gene&term=CEACAM1) | carcinoembryonic antigen-related cell adhesion molecule 1 (biliary glycoprotein) | [NM_001184816](http://www.ncbi.nlm.nih.gov/entrez/query.fcgi?db=Nucleotide&term=NM_001184816) | hs\|19q13.2 |
| 0.0066322 | 0.137 | 0.015873 | 0.66 | 2.25 | 3.45 | [LOC100507420](http://www.ncbi.nlm.nih.gov/entrez/query.fcgi?cmd=search&db=gene&term=LOC100507420) | uncharacterized LOC100507420 | [NR_121620](http://www.ncbi.nlm.nih.gov/entrez/query.fcgi?db=Nucleotide&term=NR_121620) | hs\|8p11.23 |
| 0.0074732 | 0.139 | 0.0238095 | 0.71 | 2.47 | 3.45 | [SLC22A18AS](http://www.ncbi.nlm.nih.gov/entrez/query.fcgi?cmd=search&db=gene&term=SLC22A18AS) | solute carrier family 22 (organic cation transporter), member 18 antisense | [NM_007105](http://www.ncbi.nlm.nih.gov/entrez/query.fcgi?db=Nucleotide&term=NM_007105) | hs\|11p15.4 |
| 0.0008198 | 0.137 | 0.0079365 | 0.68 | 2.29 | 3.45 | [SPATC1](http://www.ncbi.nlm.nih.gov/entrez/query.fcgi?cmd=search&db=gene&term=SPATC1) | spermatogenesis and centriole associated 1 | [NM_198572](http://www.ncbi.nlm.nih.gov/entrez/query.fcgi?db=Nucleotide&term=NM_198572) | hs\|8q24.3 |
| 0.0017603 | 0.137 | 0.015873 | 0.66 | 2.3 | 3.45 | [TGM2](http://www.ncbi.nlm.nih.gov/entrez/query.fcgi?cmd=search&db=gene&term=TGM2) | transglutaminase 2 | [NM_198951](http://www.ncbi.nlm.nih.gov/entrez/query.fcgi?db=Nucleotide&term=NM_198951) | hs\|20q11.23 |
| 0.0029049 | 0.137 | 0.0238095 | 0.57 | 1.99 | 3.45 | [TMPRSS3](http://www.ncbi.nlm.nih.gov/entrez/query.fcgi?cmd=search&db=gene&term=TMPRSS3) | transmembrane protease, serine 3 | [NM_032405](http://www.ncbi.nlm.nih.gov/entrez/query.fcgi?db=Nucleotide&term=NM_032405) | hs\|21q22.3 |
| 0.007829 | 0.14 | 0.015873 | 0.59 | 2.02 | 3.45 | [TRIM59](http://www.ncbi.nlm.nih.gov/entrez/query.fcgi?cmd=search&db=gene&term=TRIM59) | tripartite motif containing 59 | [NM_173084](http://www.ncbi.nlm.nih.gov/entrez/query.fcgi?db=Nucleotide&term=NM_173084) | hs\|3q25.33 |
| 0.0050734 | 0.137 | 0.0238095 | 0.62 | 2.06 | 3.33 | [CAPG](http://www.ncbi.nlm.nih.gov/entrez/query.fcgi?cmd=search&db=gene&term=CAPG) | capping protein (actin filament), gelsolin-like | [NM_001256140](http://www.ncbi.nlm.nih.gov/entrez/query.fcgi?db=Nucleotide&term=NM_001256140) | hs\|2p11.2 |
| 0.0037713 | 0.137 | 0.015873 | 0.69 | 2.29 | 3.33 | [CLTB](http://www.ncbi.nlm.nih.gov/entrez/query.fcgi?cmd=search&db=gene&term=CLTB) | clathrin, light chain B | [NM_007097](http://www.ncbi.nlm.nih.gov/entrez/query.fcgi?db=Nucleotide&term=NM_007097) | hs\|5q35.2 |
| 0.0059197 | 0.137 | 0.0079365 | 0.77 | 2.58 | 3.33 | [EPS8L1](http://www.ncbi.nlm.nih.gov/entrez/query.fcgi?cmd=search&db=gene&term=EPS8L1) | EPS8-like 1 | [NM_133180](http://www.ncbi.nlm.nih.gov/entrez/query.fcgi?db=Nucleotide&term=NM_133180) | hs\|19q13.42 |
| 0.0094134 | 0.143 | 0.0238095 | 0.73 | 2.41 | 3.33 | [FHAD1](http://www.ncbi.nlm.nih.gov/entrez/query.fcgi?cmd=search&db=gene&term=FHAD1) | forkhead-associated (FHA) phosphopeptide binding domain 1 | [NM_052929](http://www.ncbi.nlm.nih.gov/entrez/query.fcgi?db=Nucleotide&term=NM_052929) | hs\|1p36.21 |
| 0.0030042 | 0.137 | 0.015873 | 0.61 | 2.03 | 3.33 | [PROC](http://www.ncbi.nlm.nih.gov/entrez/query.fcgi?cmd=search&db=gene&term=PROC) | protein C (inactivator of coagulation factors Va and VIIIa) | [NM_000312](http://www.ncbi.nlm.nih.gov/entrez/query.fcgi?db=Nucleotide&term=NM_000312) | hs\|2q14.3 |
| 0.0006657 | 0.137 | 0.0079365 | 0.58 | 1.95 | 3.33 | [SLC45A3](http://www.ncbi.nlm.nih.gov/entrez/query.fcgi?cmd=search&db=gene&term=SLC45A3) | solute carrier family 45, member 3 | [NM_033102](http://www.ncbi.nlm.nih.gov/entrez/query.fcgi?db=Nucleotide&term=NM_033102) | hs\|1q32.1 |
| 0.0045246 | 0.137 | 0.015873 | 0.66 | 2.19 | 3.33 | [UNC5B-AS1](http://www.ncbi.nlm.nih.gov/entrez/query.fcgi?cmd=search&db=gene&term=UNC5B-AS1) | UNC5B antisense RNA 1 | [NR_038453](http://www.ncbi.nlm.nih.gov/entrez/query.fcgi?db=Nucleotide&term=NR_038453) | hs\|10q22.1 |
| 0.0031431 | 0.137 | 0.0079365 | 0.43 | 1.45 | 3.33 |  |  |  | hs\|16p12.1 |
| 0.0046843 | 0.137 | 0.015873 | 0.63 | 2.05 | 3.23 | [ANO1](http://www.ncbi.nlm.nih.gov/entrez/query.fcgi?cmd=search&db=gene&term=ANO1) | anoctamin 1, calcium activated chloride channel | [NM_018043](http://www.ncbi.nlm.nih.gov/entrez/query.fcgi?db=Nucleotide&term=NM_018043) | hs\|11q13.3 |
| 0.007308 | 0.139 | 0.031746 | 0.51 | 1.64 | 3.23 | [B3GNT3](http://www.ncbi.nlm.nih.gov/entrez/query.fcgi?cmd=search&db=gene&term=B3GNT3) | UDP-GlcNAc:betaGal beta-1,3-N-acetylglucosaminyltransferase 3 | [NM_014256](http://www.ncbi.nlm.nih.gov/entrez/query.fcgi?db=Nucleotide&term=NM_014256) | hs\|19p13.11 |
| 0.0005978 | 0.137 | 0.0079365 | 0.5 | 1.61 | 3.23 | [FUT3](http://www.ncbi.nlm.nih.gov/entrez/query.fcgi?cmd=search&db=gene&term=FUT3) | fucosyltransferase 3 (galactoside 3(4)-L-fucosyltransferase, Lewis blood group) | [NM_000149](http://www.ncbi.nlm.nih.gov/entrez/query.fcgi?db=Nucleotide&term=NM_000149) | hs\|19p13.3 |
| 0.0021033 | 0.137 | 0.0079365 | 0.51 | 1.65 | 3.23 | [HOXB5](http://www.ncbi.nlm.nih.gov/entrez/query.fcgi?cmd=search&db=gene&term=HOXB5) | homeobox B5 | [NM_002147](http://www.ncbi.nlm.nih.gov/entrez/query.fcgi?db=Nucleotide&term=NM_002147) | hs\|17q21.32 |
| 0.0050021 | 0.137 | 0.015873 | 0.56 | 1.84 | 3.23 | [SERPINA1](http://www.ncbi.nlm.nih.gov/entrez/query.fcgi?cmd=search&db=gene&term=SERPINA1) | serpin peptidase inhibitor, clade A (alpha-1 antiproteinase, antitrypsin), member 1 | [NM_001002236](http://www.ncbi.nlm.nih.gov/entrez/query.fcgi?db=Nucleotide&term=NM_001002236) | hs\|14q32.13 |
| 0.0044252 | 0.137 | 0.015873 | 0.6 | 1.93 | 3.23 | [SP140](http://www.ncbi.nlm.nih.gov/entrez/query.fcgi?cmd=search&db=gene&term=SP140) | SP140 nuclear body protein | [NM_001005176](http://www.ncbi.nlm.nih.gov/entrez/query.fcgi?db=Nucleotide&term=NM_001005176) | hs\|2q37.1 |
| 0.0051377 | 0.137 | 0.0238095 | 0.61 | 1.91 | 3.13 | [BICD1](http://www.ncbi.nlm.nih.gov/entrez/query.fcgi?cmd=search&db=gene&term=BICD1) | bicaudal D homolog 1 (Drosophila) | [NM_001714](http://www.ncbi.nlm.nih.gov/entrez/query.fcgi?db=Nucleotide&term=NM_001714) | hs\|12p11.21 |
| 0.0044525 | 0.137 | 0.015873 | 0.69 | 2.16 | 3.13 | [CKMT1A](http://www.ncbi.nlm.nih.gov/entrez/query.fcgi?cmd=search&db=gene&term=CKMT1A) | creatine kinase, mitochondrial 1A | [NM_001015001](http://www.ncbi.nlm.nih.gov/entrez/query.fcgi?db=Nucleotide&term=NM_001015001) | hs\|15q15.3 |
| 0.0080993 | 0.141 | 0.031746 | 0.78 | 2.44 | 3.13 | [FRMD5](http://www.ncbi.nlm.nih.gov/entrez/query.fcgi?cmd=search&db=gene&term=FRMD5) | FERM domain containing 5 | [NM_001286491](http://www.ncbi.nlm.nih.gov/entrez/query.fcgi?db=Nucleotide&term=NM_001286491) | hs\|15q15.3 |
| 0.0026129 | 0.137 | 0.015873 | 0.62 | 1.93 | 3.13 | [HOXB6](http://www.ncbi.nlm.nih.gov/entrez/query.fcgi?cmd=search&db=gene&term=HOXB6) | homeobox B6 | [NM_018952](http://www.ncbi.nlm.nih.gov/entrez/query.fcgi?db=Nucleotide&term=NM_018952) | hs\|17q21.32 |
| 0.0098992 | 0.144 | 0.031746 | 0.5 | 1.57 | 3.13 | [HTRA3](http://www.ncbi.nlm.nih.gov/entrez/query.fcgi?cmd=search&db=gene&term=HTRA3) | HtrA serine peptidase 3 | [NM_053044](http://www.ncbi.nlm.nih.gov/entrez/query.fcgi?db=Nucleotide&term=NM_053044) | hs\|4p16.1 |
| 0.0022454 | 0.137 | 0.015873 | 0.53 | 1.65 | 3.13 | [MLPH](http://www.ncbi.nlm.nih.gov/entrez/query.fcgi?cmd=search&db=gene&term=MLPH) | melanophilin | [NM_024101](http://www.ncbi.nlm.nih.gov/entrez/query.fcgi?db=Nucleotide&term=NM_024101) | hs\|2q37.3 |
| 0.0049769 | 0.137 | 0.0238095 | 0.55 | 1.71 | 3.13 | [PLEKHS1](http://www.ncbi.nlm.nih.gov/entrez/query.fcgi?cmd=search&db=gene&term=PLEKHS1) | pleckstrin homology domain containing, family S member 1 | [NM_182601](http://www.ncbi.nlm.nih.gov/entrez/query.fcgi?db=Nucleotide&term=NM_182601) | hs\|10q25.3 |
| 0.0045002 | 0.137 | 0.0238095 | 0.65 | 2.01 | 3.13 | [S100A11](http://www.ncbi.nlm.nih.gov/entrez/query.fcgi?cmd=search&db=gene&term=S100A11) | S100 calcium binding protein A11 | [NM_005620](http://www.ncbi.nlm.nih.gov/entrez/query.fcgi?db=Nucleotide&term=NM_005620) | hs\|1q21.3 |
| 0.0030334 | 0.137 | 0.0238095 | 0.8 | 2.43 | 3.03 | [ITGA2](http://www.ncbi.nlm.nih.gov/entrez/query.fcgi?cmd=search&db=gene&term=ITGA2) | integrin, alpha 2 (CD49B, alpha 2 subunit of VLA-2 receptor) | [NM_002203](http://www.ncbi.nlm.nih.gov/entrez/query.fcgi?db=Nucleotide&term=NM_002203) | hs\|5q11.2 |
| 0.0022189 | 0.137 | 0.0079365 | 0.73 | 2.2 | 3.03 | [ITGA3](http://www.ncbi.nlm.nih.gov/entrez/query.fcgi?cmd=search&db=gene&term=ITGA3) | integrin, alpha 3 (antigen CD49C, alpha 3 subunit of VLA-3 receptor) | [NM_002204](http://www.ncbi.nlm.nih.gov/entrez/query.fcgi?db=Nucleotide&term=NM_002204) | hs\|17q21.33 |
| 0.0081943 | 0.141 | 0.0238095 | 0.57 | 1.74 | 3.03 | [KYNU](http://www.ncbi.nlm.nih.gov/entrez/query.fcgi?cmd=search&db=gene&term=KYNU) | kynureninase | [NM_001032998](http://www.ncbi.nlm.nih.gov/entrez/query.fcgi?db=Nucleotide&term=NM_001032998) | hs\|2q22.2 |
| 0.0032812 | 0.137 | 0.015873 | 0.53 | 1.61 | 3.03 | [lnc-CBX2-1](http://www.ncbi.nlm.nih.gov/entrez/query.fcgi?cmd=search&db=gene&term=lnc-CBX2-1) | lnc-CBX2-1:1 |  | hs\|17q25.3 |
| 0.0087379 | 0.142 | 0.015873 | 0.79 | 2.38 | 3.03 | [LRRC66](http://www.ncbi.nlm.nih.gov/entrez/query.fcgi?cmd=search&db=gene&term=LRRC66) | leucine rich repeat containing 66 | [NM_001024611](http://www.ncbi.nlm.nih.gov/entrez/query.fcgi?db=Nucleotide&term=NM_001024611) | hs\|4q12 |
| 0.00402 | 0.137 | 0.015873 | 0.59 | 1.77 | 3.03 | [TSPAN8](http://www.ncbi.nlm.nih.gov/entrez/query.fcgi?cmd=search&db=gene&term=TSPAN8) | tetraspanin 8 | [NM_004616](http://www.ncbi.nlm.nih.gov/entrez/query.fcgi?db=Nucleotide&term=NM_004616) | hs\|12q21.1 |
| 0.0048422 | 0.137 | 0.015873 | 0.63 | 1.85 | 2.94 | [ARL4C](http://www.ncbi.nlm.nih.gov/entrez/query.fcgi?cmd=search&db=gene&term=ARL4C) | ADP-ribosylation factor-like 4C | [NM_001282431](http://www.ncbi.nlm.nih.gov/entrez/query.fcgi?db=Nucleotide&term=NM_001282431) | hs\|2q37.1 |
| 0.0022604 | 0.137 | 0.015873 | 0.66 | 1.92 | 2.94 | [CDCA7](http://www.ncbi.nlm.nih.gov/entrez/query.fcgi?cmd=search&db=gene&term=CDCA7) | cell division cycle associated 7 | [NM_031942](http://www.ncbi.nlm.nih.gov/entrez/query.fcgi?db=Nucleotide&term=NM_031942) | hs\|2q31.1 |
| 0.0010796 | 0.137 | 0.0079365 | 0.58 | 1.71 | 2.94 | [CKLF](http://www.ncbi.nlm.nih.gov/entrez/query.fcgi?cmd=search&db=gene&term=CKLF) | chemokine-like factor | [NM_016951](http://www.ncbi.nlm.nih.gov/entrez/query.fcgi?db=Nucleotide&term=NM_016951) | hs\|16q21 |
| 0.0027223 | 0.137 | 0.015873 | 0.53 | 1.56 | 2.94 | [CORO2A](http://www.ncbi.nlm.nih.gov/entrez/query.fcgi?cmd=search&db=gene&term=CORO2A) | coronin, actin binding protein, 2A | [NM_003389](http://www.ncbi.nlm.nih.gov/entrez/query.fcgi?db=Nucleotide&term=NM_003389) | hs\|9q22.33 |
| 0.0022717 | 0.137 | 0.0079365 | 0.76 | 2.24 | 2.94 | [HOXC-AS2](http://www.ncbi.nlm.nih.gov/entrez/query.fcgi?cmd=search&db=gene&term=HOXC-AS2) | HOXC cluster antisense RNA 2 | [NR_047505](http://www.ncbi.nlm.nih.gov/entrez/query.fcgi?db=Nucleotide&term=NR_047505) | hs\|12q13.13 |
| 0.000137 | 0.137 | 0.0079365 | 0.66 | 1.95 | 2.94 | [lnc-RP11-582J16.5.1-3](http://www.ncbi.nlm.nih.gov/entrez/query.fcgi?cmd=search&db=gene&term=lnc-RP11-582J16.5.1-3) | lnc-RP11-582J16.5.1-3:1 |  | hs\|8p21.3 |
| 0.0020086 | 0.137 | 0.0079365 | 0.69 | 2.05 | 2.94 | [LOC100996573](http://www.ncbi.nlm.nih.gov/entrez/query.fcgi?cmd=search&db=gene&term=LOC100996573) | uncharacterized LOC100996573 | [XM_003846565](http://www.ncbi.nlm.nih.gov/entrez/query.fcgi?db=Nucleotide&term=XM_003846565) | hs\|12q24.33 |
| 0.0010589 | 0.137 | 0.015873 | 0.5 | 1.49 | 2.94 | [P3H2](http://www.ncbi.nlm.nih.gov/entrez/query.fcgi?cmd=search&db=gene&term=P3H2) | prolyl 3-hydroxylase 2 | [NM_018192](http://www.ncbi.nlm.nih.gov/entrez/query.fcgi?db=Nucleotide&term=NM_018192) | hs\|3q28 |
| 0.0008165 | 0.137 | 0.0079365 | 0.54 | 1.59 | 2.94 | [SGCD](http://www.ncbi.nlm.nih.gov/entrez/query.fcgi?cmd=search&db=gene&term=SGCD) | sarcoglycan, delta (35kDa dystrophin-associated glycoprotein) | [NM_172244](http://www.ncbi.nlm.nih.gov/entrez/query.fcgi?db=Nucleotide&term=NM_172244) | hs\|5q33.3 |
| 0.0067976 | 0.137 | 0.015873 | 0.66 | 1.93 | 2.94 | [TTC24](http://www.ncbi.nlm.nih.gov/entrez/query.fcgi?cmd=search&db=gene&term=TTC24) | tetratricopeptide repeat domain 24 | [NM_001105669](http://www.ncbi.nlm.nih.gov/entrez/query.fcgi?db=Nucleotide&term=NM_001105669) | hs\|1q23.1 |
| 0.0003186 | 0.137 | 0.0079365 | 0.59 | 1.72 | 2.86 | [ACSL5](http://www.ncbi.nlm.nih.gov/entrez/query.fcgi?cmd=search&db=gene&term=ACSL5) | acyl-CoA synthetase long-chain family member 5 | [NM_203380](http://www.ncbi.nlm.nih.gov/entrez/query.fcgi?db=Nucleotide&term=NM_203380) | hs\|10q25.2 |
| 0.0058474 | 0.137 | 0.015873 | 0.67 | 1.92 | 2.86 | [CLDN4](http://www.ncbi.nlm.nih.gov/entrez/query.fcgi?cmd=search&db=gene&term=CLDN4) | claudin 4 | [NM_001305](http://www.ncbi.nlm.nih.gov/entrez/query.fcgi?db=Nucleotide&term=NM_001305) | hs\|7q11.23 |
| 0.0062175 | 0.137 | 0.015873 | 0.56 | 1.61 | 2.86 | [ETV7](http://www.ncbi.nlm.nih.gov/entrez/query.fcgi?cmd=search&db=gene&term=ETV7) | ets variant 7 | [NM_016135](http://www.ncbi.nlm.nih.gov/entrez/query.fcgi?db=Nucleotide&term=NM_016135) | hs\|6p21.31 |
| 0.0004546 | 0.137 | 0.0079365 | 0.54 | 1.55 | 2.86 | [IER5L](http://www.ncbi.nlm.nih.gov/entrez/query.fcgi?cmd=search&db=gene&term=IER5L) | immediate early response 5-like | [NM_203434](http://www.ncbi.nlm.nih.gov/entrez/query.fcgi?db=Nucleotide&term=NM_203434) | hs\|9q34.11 |
| 0.0005843 | 0.137 | 0.0079365 | 0.61 | 1.74 | 2.86 | [LIF](http://www.ncbi.nlm.nih.gov/entrez/query.fcgi?cmd=search&db=gene&term=LIF) | leukemia inhibitory factor | [NM_002309](http://www.ncbi.nlm.nih.gov/entrez/query.fcgi?db=Nucleotide&term=NM_002309) | hs\|22q12.2 |
| 0.0032187 | 0.137 | 0.015873 | 0.62 | 1.8 | 2.86 | [LINC00673](http://www.ncbi.nlm.nih.gov/entrez/query.fcgi?cmd=search&db=gene&term=LINC00673) | long intergenic non-protein coding RNA 673 | [NR_036488](http://www.ncbi.nlm.nih.gov/entrez/query.fcgi?db=Nucleotide&term=NR_036488) | hs\|17q24.3 |
| 0.0004348 | 0.137 | 0.0079365 | 0.6 | 1.74 | 2.86 | [MTMR11](http://www.ncbi.nlm.nih.gov/entrez/query.fcgi?cmd=search&db=gene&term=MTMR11) | myotubularin related protein 11 | [NM_181873](http://www.ncbi.nlm.nih.gov/entrez/query.fcgi?db=Nucleotide&term=NM_181873) | hs\|1q21.2 |
| 0.0084497 | 0.141 | 0.015873 | 0.78 | 2.26 | 2.86 | [RASAL1](http://www.ncbi.nlm.nih.gov/entrez/query.fcgi?cmd=search&db=gene&term=RASAL1) | RAS protein activator like 1 (GAP1 like) | [NM_004658](http://www.ncbi.nlm.nih.gov/entrez/query.fcgi?db=Nucleotide&term=NM_004658) | hs\|12q24.13 |
| 0.0083507 | 0.141 | 0.031746 | 0.6 | 1.72 | 2.86 | [RASGRP1](http://www.ncbi.nlm.nih.gov/entrez/query.fcgi?cmd=search&db=gene&term=RASGRP1) | RAS guanyl releasing protein 1 (calcium and DAG-regulated) | [NM_005739](http://www.ncbi.nlm.nih.gov/entrez/query.fcgi?db=Nucleotide&term=NM_005739) | hs\|15q14 |
| 0.0069019 | 0.138 | 0.015873 | 0.48 | 1.36 | 2.86 | [RHOH](http://www.ncbi.nlm.nih.gov/entrez/query.fcgi?cmd=search&db=gene&term=RHOH) | ras homolog family member H | [NM_004310](http://www.ncbi.nlm.nih.gov/entrez/query.fcgi?db=Nucleotide&term=NM_004310) | hs\|4p14 |
| 0.0070498 | 0.138 | 0.015873 | 0.62 | 1.78 | 2.86 | [TBC1D2](http://www.ncbi.nlm.nih.gov/entrez/query.fcgi?cmd=search&db=gene&term=TBC1D2) | TBC1 domain family, member 2 | [NM_001267571](http://www.ncbi.nlm.nih.gov/entrez/query.fcgi?db=Nucleotide&term=NM_001267571) | hs\|9q22.33 |
| 0.0099302 | 0.144 | 0.031746 | 0.61 | 1.75 | 2.86 | [TPM4](http://www.ncbi.nlm.nih.gov/entrez/query.fcgi?cmd=search&db=gene&term=TPM4) | tropomyosin 4 | [NM_003290](http://www.ncbi.nlm.nih.gov/entrez/query.fcgi?db=Nucleotide&term=NM_003290) | hs\|19p13.12 |
| 0.0012667 | 0.137 | 0.0079365 | 0.75 | 2.13 | 2.86 | [UNC5B](http://www.ncbi.nlm.nih.gov/entrez/query.fcgi?cmd=search&db=gene&term=UNC5B) | unc-5 homolog B (C. elegans) | [NM_170744](http://www.ncbi.nlm.nih.gov/entrez/query.fcgi?db=Nucleotide&term=NM_170744) | hs\|10q22.1 |
| 0.0019755 | 0.137 | 0.0079365 | 0.68 | 1.88 | 2.78 | [BTBD16](http://www.ncbi.nlm.nih.gov/entrez/query.fcgi?cmd=search&db=gene&term=BTBD16) | BTB (POZ) domain containing 16 | [NM_144587](http://www.ncbi.nlm.nih.gov/entrez/query.fcgi?db=Nucleotide&term=NM_144587) | hs\|10q26.13 |
| 0.0031409 | 0.137 | 0.0079365 | 0.59 | 1.64 | 2.78 | [CASC15](http://www.ncbi.nlm.nih.gov/entrez/query.fcgi?cmd=search&db=gene&term=CASC15) | cancer susceptibility candidate 15 (non-protein coding) | [NR_015410](http://www.ncbi.nlm.nih.gov/entrez/query.fcgi?db=Nucleotide&term=NR_015410) | hs\|6p22.3 |
| 0.003988 | 0.137 | 0.015873 | 0.61 | 1.69 | 2.78 | [CHST1](http://www.ncbi.nlm.nih.gov/entrez/query.fcgi?cmd=search&db=gene&term=CHST1) | carbohydrate (keratan sulfate Gal-6) sulfotransferase 1 | [NM_003654](http://www.ncbi.nlm.nih.gov/entrez/query.fcgi?db=Nucleotide&term=NM_003654) | hs\|11p11.2 |
| 0.0046722 | 0.137 | 0.015873 | 0.48 | 1.31 | 2.78 | [CXCR4](http://www.ncbi.nlm.nih.gov/entrez/query.fcgi?cmd=search&db=gene&term=CXCR4) | chemokine (C-X-C motif) receptor 4 | [NM_001008540](http://www.ncbi.nlm.nih.gov/entrez/query.fcgi?db=Nucleotide&term=NM_001008540) | hs\|2q22.1 |
| 0.001439 | 0.137 | 0.0079365 | 0.72 | 1.97 | 2.78 | [FERMT1](http://www.ncbi.nlm.nih.gov/entrez/query.fcgi?cmd=search&db=gene&term=FERMT1) | fermitin family member 1 | [NM_017671](http://www.ncbi.nlm.nih.gov/entrez/query.fcgi?db=Nucleotide&term=NM_017671) | hs\|20p12.3 |
| 0.0052289 | 0.137 | 0.0079365 | 0.65 | 1.8 | 2.78 | [GK](http://www.ncbi.nlm.nih.gov/entrez/query.fcgi?cmd=search&db=gene&term=GK) | glycerol kinase | [NM_001205019](http://www.ncbi.nlm.nih.gov/entrez/query.fcgi?db=Nucleotide&term=NM_001205019) | hs\|Xp21.2 |
| 0.0005861 | 0.137 | 0.0079365 | 0.59 | 1.61 | 2.78 | [LINC01132](http://www.ncbi.nlm.nih.gov/entrez/query.fcgi?cmd=search&db=gene&term=LINC01132) | long intergenic non-protein coding RNA 1132 | [NR_038856](http://www.ncbi.nlm.nih.gov/entrez/query.fcgi?db=Nucleotide&term=NR_038856) | hs\|1q42.3 |
| 0.0018225 | 0.137 | 0.0079365 | 0.59 | 1.66 | 2.78 | [LOC101927975](http://www.ncbi.nlm.nih.gov/entrez/query.fcgi?cmd=search&db=gene&term=LOC101927975) | uncharacterized LOC101927975 | [XR_246372](http://www.ncbi.nlm.nih.gov/entrez/query.fcgi?db=Nucleotide&term=XR_246372) | hs\|1p22.2 |
| 0.0037734 | 0.137 | 0.015873 | 0.58 | 1.57 | 2.7 | [AKAP5](http://www.ncbi.nlm.nih.gov/entrez/query.fcgi?cmd=search&db=gene&term=AKAP5) | A kinase (PRKA) anchor protein 5 | [NM_004857](http://www.ncbi.nlm.nih.gov/entrez/query.fcgi?db=Nucleotide&term=NM_004857) | hs\|14q23.3 |
| 0.0002926 | 0.137 | 0.0079365 | 0.74 | 2.01 | 2.7 | [AP1S3](http://www.ncbi.nlm.nih.gov/entrez/query.fcgi?cmd=search&db=gene&term=AP1S3) | adaptor-related protein complex 1, sigma 3 subunit | [NM_001039569](http://www.ncbi.nlm.nih.gov/entrez/query.fcgi?db=Nucleotide&term=NM_001039569) | hs\|2q36.1 |
| 0.0049187 | 0.137 | 0.0238095 | 0.68 | 1.81 | 2.7 | [ARL9](http://www.ncbi.nlm.nih.gov/entrez/query.fcgi?cmd=search&db=gene&term=ARL9) | ADP-ribosylation factor-like 9 | [NM_206919](http://www.ncbi.nlm.nih.gov/entrez/query.fcgi?db=Nucleotide&term=NM_206919) | hs\|4q12 |
| 0.0075218 | 0.139 | 0.0238095 | 0.57 | 1.57 | 2.7 | [C1QTNF5](http://www.ncbi.nlm.nih.gov/entrez/query.fcgi?cmd=search&db=gene&term=C1QTNF5) | C1q and tumor necrosis factor related protein 5 | [NM_015645](http://www.ncbi.nlm.nih.gov/entrez/query.fcgi?db=Nucleotide&term=NM_015645) | hs\|11q23.3 |
| 0.0074348 | 0.139 | 0.0079365 | 0.74 | 2.01 | 2.7 | [DSC2](http://www.ncbi.nlm.nih.gov/entrez/query.fcgi?cmd=search&db=gene&term=DSC2) | desmocollin 2 | [AF085831](http://www.ncbi.nlm.nih.gov/entrez/query.fcgi?db=Nucleotide&term=AF085831) | hs\|18q12.1 |
| 0.0023338 | 0.137 | 0.015873 | 0.59 | 1.58 | 2.7 | [FAM81A](http://www.ncbi.nlm.nih.gov/entrez/query.fcgi?cmd=search&db=gene&term=FAM81A) | family with sequence similarity 81, member A | [NM_152450](http://www.ncbi.nlm.nih.gov/entrez/query.fcgi?db=Nucleotide&term=NM_152450) | hs\|15q22.2 |
| 0.000828 | 0.137 | 0.0079365 | 0.52 | 1.43 | 2.7 | [FUT6](http://www.ncbi.nlm.nih.gov/entrez/query.fcgi?cmd=search&db=gene&term=FUT6) | fucosyltransferase 6 (alpha (1,3) fucosyltransferase) | [NM_000150](http://www.ncbi.nlm.nih.gov/entrez/query.fcgi?db=Nucleotide&term=NM_000150) | hs\|19p13.3 |
| 0.0040919 | 0.137 | 0.015873 | 0.62 | 1.68 | 2.7 | [GALNT4](http://www.ncbi.nlm.nih.gov/entrez/query.fcgi?cmd=search&db=gene&term=GALNT4) | polypeptide N-acetylgalactosaminyltransferase 4 | [NM_003774](http://www.ncbi.nlm.nih.gov/entrez/query.fcgi?db=Nucleotide&term=NM_003774) | hs\|12q21.33 |
| 0.0043876 | 0.137 | 0.0238095 | 0.68 | 1.81 | 2.7 | [KIAA1217](http://www.ncbi.nlm.nih.gov/entrez/query.fcgi?cmd=search&db=gene&term=KIAA1217) | KIAA1217 | [NM_019590](http://www.ncbi.nlm.nih.gov/entrez/query.fcgi?db=Nucleotide&term=NM_019590) | hs\|10p12.1 |
| 0.0029898 | 0.137 | 0.015873 | 0.62 | 1.66 | 2.7 | [MGAT3](http://www.ncbi.nlm.nih.gov/entrez/query.fcgi?cmd=search&db=gene&term=MGAT3) | mannosyl (beta-1,4-)-glycoprotein beta-1,4-N-acetylglucosaminyltransferase | [NM_002409](http://www.ncbi.nlm.nih.gov/entrez/query.fcgi?db=Nucleotide&term=NM_002409) | hs\|22q13.1 |
| 0.0027483 | 0.137 | 0.015873 | 0.61 | 1.62 | 2.7 | [MYO1E](http://www.ncbi.nlm.nih.gov/entrez/query.fcgi?cmd=search&db=gene&term=MYO1E) | myosin IE | [NM_004998](http://www.ncbi.nlm.nih.gov/entrez/query.fcgi?db=Nucleotide&term=NM_004998) | hs\|15q22.2 |
| 0.0041579 | 0.137 | 0.0238095 | 0.61 | 1.64 | 2.7 | [POF1B](http://www.ncbi.nlm.nih.gov/entrez/query.fcgi?cmd=search&db=gene&term=POF1B) | premature ovarian failure, 1B | [NM_024921](http://www.ncbi.nlm.nih.gov/entrez/query.fcgi?db=Nucleotide&term=NM_024921) | hs\|Xq21.1 |
| 0.0029246 | 0.137 | 0.0079365 | 0.51 | 1.36 | 2.7 | [RARRES1](http://www.ncbi.nlm.nih.gov/entrez/query.fcgi?cmd=search&db=gene&term=RARRES1) | retinoic acid receptor responder (tazarotene induced) 1 | [NM_206963](http://www.ncbi.nlm.nih.gov/entrez/query.fcgi?db=Nucleotide&term=NM_206963) | hs\|3q25.32 |
| 0.0008571 | 0.137 | 0.0079365 | 0.68 | 1.83 | 2.7 | [TNFRSF21](http://www.ncbi.nlm.nih.gov/entrez/query.fcgi?cmd=search&db=gene&term=TNFRSF21) | tumor necrosis factor receptor superfamily, member 21 | [NM_014452](http://www.ncbi.nlm.nih.gov/entrez/query.fcgi?db=Nucleotide&term=NM_014452) | hs\|6p12.3 |
| 0.0056266 | 0.137 | 0.0238095 | 0.71 | 1.91 | 2.7 | [TUBA1C](http://www.ncbi.nlm.nih.gov/entrez/query.fcgi?cmd=search&db=gene&term=TUBA1C) | tubulin, alpha 1c | [NM_032704](http://www.ncbi.nlm.nih.gov/entrez/query.fcgi?db=Nucleotide&term=NM_032704) | hs\|12q13.12 |
| 0.0084799 | 0.141 | 0.015873 | 0.62 | 1.67 | 2.7 |  |  |  | hs\|2q13 |
| 0.0089309 | 0.142 | 0.0238095 | 0.54 | 1.41 | 2.63 | [ANTXR1](http://www.ncbi.nlm.nih.gov/entrez/query.fcgi?cmd=search&db=gene&term=ANTXR1) | anthrax toxin receptor 1 | [NM_032208](http://www.ncbi.nlm.nih.gov/entrez/query.fcgi?db=Nucleotide&term=NM_032208) | hs\|2p13.3 |
| 0.0030691 | 0.137 | 0.0238095 | 0.46 | 1.22 | 2.63 | [EDNRA](http://www.ncbi.nlm.nih.gov/entrez/query.fcgi?cmd=search&db=gene&term=EDNRA) | endothelin receptor type A | [NM_001957](http://www.ncbi.nlm.nih.gov/entrez/query.fcgi?db=Nucleotide&term=NM_001957) | hs\|4q31.22 |
| 0.0037178 | 0.137 | 0.0079365 | 0.69 | 1.85 | 2.63 | [HKDC1](http://www.ncbi.nlm.nih.gov/entrez/query.fcgi?cmd=search&db=gene&term=HKDC1) | hexokinase domain containing 1 | [NM_025130](http://www.ncbi.nlm.nih.gov/entrez/query.fcgi?db=Nucleotide&term=NM_025130) | hs\|10q22.1 |
| 0.0091284 | 0.143 | 0.031746 | 0.61 | 1.61 | 2.63 | [IL10RB-AS1](http://www.ncbi.nlm.nih.gov/entrez/query.fcgi?cmd=search&db=gene&term=IL10RB-AS1) | IL10RB antisense RNA 1 (head to head) | [NR_038974](http://www.ncbi.nlm.nih.gov/entrez/query.fcgi?db=Nucleotide&term=NR_038974) | hs\|21q22.11 |
| 0.0046845 | 0.137 | 0.015873 | 0.57 | 1.5 | 2.63 | [NREP](http://www.ncbi.nlm.nih.gov/entrez/query.fcgi?cmd=search&db=gene&term=NREP) | neuronal regeneration related protein | [NM_004772](http://www.ncbi.nlm.nih.gov/entrez/query.fcgi?db=Nucleotide&term=NM_004772) | hs\|5q22.1 |
| 0.0081028 | 0.141 | 0.0079365 | 0.73 | 1.91 | 2.63 | [SDC4](http://www.ncbi.nlm.nih.gov/entrez/query.fcgi?cmd=search&db=gene&term=SDC4) | syndecan 4 | [NM_002999](http://www.ncbi.nlm.nih.gov/entrez/query.fcgi?db=Nucleotide&term=NM_002999) | hs\|20q13.12 |
| 0.0021848 | 0.137 | 0.0079365 | 0.77 | 2.04 | 2.63 | [SLC2A1](http://www.ncbi.nlm.nih.gov/entrez/query.fcgi?cmd=search&db=gene&term=SLC2A1) | solute carrier family 2 (facilitated glucose transporter), member 1 | [NM_006516](http://www.ncbi.nlm.nih.gov/entrez/query.fcgi?db=Nucleotide&term=NM_006516) | hs\|1p34.2 |
| 0.003239 | 0.137 | 0.0079365 | 0.64 | 1.68 | 2.63 | [SLC35E3](http://www.ncbi.nlm.nih.gov/entrez/query.fcgi?cmd=search&db=gene&term=SLC35E3) | solute carrier family 35, member E3 | [AF119871](http://www.ncbi.nlm.nih.gov/entrez/query.fcgi?db=Nucleotide&term=AF119871) | hs\|12q15 |
| 0.0043261 | 0.137 | 0.0079365 | 0.59 | 1.54 | 2.63 | [TSPAN15](http://www.ncbi.nlm.nih.gov/entrez/query.fcgi?cmd=search&db=gene&term=TSPAN15) | tetraspanin 15 | [NM_012339](http://www.ncbi.nlm.nih.gov/entrez/query.fcgi?db=Nucleotide&term=NM_012339) | hs\|10q22.1 |
| 0.0063476 | 0.137 | 0.031746 | 0.73 | 1.93 | 2.63 |  |  |  | hs\|2p13.1 |
| 0.0084833 | 0.141 | 0.015873 | 0.65 | 1.65 | 2.56 | [CMTM3](http://www.ncbi.nlm.nih.gov/entrez/query.fcgi?cmd=search&db=gene&term=CMTM3) | CKLF-like MARVEL transmembrane domain containing 3 | [NM_144601](http://www.ncbi.nlm.nih.gov/entrez/query.fcgi?db=Nucleotide&term=NM_144601) | hs\|16q21 |
| 0.0081991 | 0.141 | 0.0238095 | 0.67 | 1.74 | 2.56 | [ECM1](http://www.ncbi.nlm.nih.gov/entrez/query.fcgi?cmd=search&db=gene&term=ECM1) | extracellular matrix protein 1 | [NM_004425](http://www.ncbi.nlm.nih.gov/entrez/query.fcgi?db=Nucleotide&term=NM_004425) | hs\|1q21.3 |
| 0.0017488 | 0.137 | 0.015873 | 0.71 | 1.81 | 2.56 | [EPHA2](http://www.ncbi.nlm.nih.gov/entrez/query.fcgi?cmd=search&db=gene&term=EPHA2) | EPH receptor A2 | [NM_004431](http://www.ncbi.nlm.nih.gov/entrez/query.fcgi?db=Nucleotide&term=NM_004431) | hs\|1p36.13 |
| 0.005958 | 0.137 | 0.015873 | 0.58 | 1.49 | 2.56 | [GBP3](http://www.ncbi.nlm.nih.gov/entrez/query.fcgi?cmd=search&db=gene&term=GBP3) | guanylate binding protein 3 | [NM_018284](http://www.ncbi.nlm.nih.gov/entrez/query.fcgi?db=Nucleotide&term=NM_018284) | hs\|1p22.2 |
| 0.0099619 | 0.144 | 0.031746 | 0.71 | 1.84 | 2.56 | [HELLS](http://www.ncbi.nlm.nih.gov/entrez/query.fcgi?cmd=search&db=gene&term=HELLS) | helicase, lymphoid-specific | [NM_018063](http://www.ncbi.nlm.nih.gov/entrez/query.fcgi?db=Nucleotide&term=NM_018063) | hs\|10q23.33 |
| 0.0073424 | 0.139 | 0.0238095 | 0.65 | 1.65 | 2.56 | [HHIPL1](http://www.ncbi.nlm.nih.gov/entrez/query.fcgi?cmd=search&db=gene&term=HHIPL1) | HHIP-like 1 | [NM_001127258](http://www.ncbi.nlm.nih.gov/entrez/query.fcgi?db=Nucleotide&term=NM_001127258) | hs\|14q32.2 |
| 0.00904 | 0.143 | 0.031746 | 0.77 | 1.97 | 2.56 | [HMGA1](http://www.ncbi.nlm.nih.gov/entrez/query.fcgi?cmd=search&db=gene&term=HMGA1) | high mobility group AT-hook 1 | [NM_145901](http://www.ncbi.nlm.nih.gov/entrez/query.fcgi?db=Nucleotide&term=NM_145901) | hs\|6p21.31 |
| 0.007283 | 0.139 | 0.031746 | 0.64 | 1.65 | 2.56 | [LOC101928304](http://www.ncbi.nlm.nih.gov/entrez/query.fcgi?cmd=search&db=gene&term=LOC101928304) | uncharacterized LOC101928304 | [NR_125855](http://www.ncbi.nlm.nih.gov/entrez/query.fcgi?db=Nucleotide&term=NR_125855) | hs\|6q23.2 |
| 0.0080303 | 0.141 | 0.0238095 | 0.63 | 1.61 | 2.56 | [POF1B](http://www.ncbi.nlm.nih.gov/entrez/query.fcgi?cmd=search&db=gene&term=POF1B) | premature ovarian failure, 1B | [NM_024921](http://www.ncbi.nlm.nih.gov/entrez/query.fcgi?db=Nucleotide&term=NM_024921) | hs\|Xq21.1 |
| 0.0081829 | 0.141 | 0.0238095 | 0.67 | 1.71 | 2.56 | [S100A11](http://www.ncbi.nlm.nih.gov/entrez/query.fcgi?cmd=search&db=gene&term=S100A11) | S100 calcium binding protein A11 | [NM_005620](http://www.ncbi.nlm.nih.gov/entrez/query.fcgi?db=Nucleotide&term=NM_005620) | hs\|1q21.3 |
| 0.0010697 | 0.137 | 0.0079365 | 0.69 | 1.77 | 2.56 | [S100A6](http://www.ncbi.nlm.nih.gov/entrez/query.fcgi?cmd=search&db=gene&term=S100A6) | S100 calcium binding protein A6 | [NM_014624](http://www.ncbi.nlm.nih.gov/entrez/query.fcgi?db=Nucleotide&term=NM_014624) | hs\|1q21.3 |
| 0.0028634 | 0.137 | 0.0079365 | 0.55 | 1.41 | 2.56 | [SEMA4F](http://www.ncbi.nlm.nih.gov/entrez/query.fcgi?cmd=search&db=gene&term=SEMA4F) | sema domain, immunoglobulin domain (Ig), transmembrane domain (TM) and short cytoplasmic domain, (semaphorin) 4F | [NM_004263](http://www.ncbi.nlm.nih.gov/entrez/query.fcgi?db=Nucleotide&term=NM_004263) | hs\|2p13.1 |
| 0.0076156 | 0.139 | 0.031746 | 0.54 | 1.39 | 2.56 | [SLC52A3](http://www.ncbi.nlm.nih.gov/entrez/query.fcgi?cmd=search&db=gene&term=SLC52A3) | solute carrier family 52 (riboflavin transporter), member 3 | [NM_033409](http://www.ncbi.nlm.nih.gov/entrez/query.fcgi?db=Nucleotide&term=NM_033409) | hs\|20p13 |
| 0.0022756 | 0.137 | 0.0079365 | 0.71 | 1.83 | 2.56 | [SQLE](http://www.ncbi.nlm.nih.gov/entrez/query.fcgi?cmd=search&db=gene&term=SQLE) | squalene epoxidase | [NM_003129](http://www.ncbi.nlm.nih.gov/entrez/query.fcgi?db=Nucleotide&term=NM_003129) | hs\|8q24.13 |
| 0.0040047 | 0.137 | 0.0079365 | 0.59 | 1.52 | 2.56 | [ZSWIM4](http://www.ncbi.nlm.nih.gov/entrez/query.fcgi?cmd=search&db=gene&term=ZSWIM4) | zinc finger, SWIM-type containing 4 | [NM_023072](http://www.ncbi.nlm.nih.gov/entrez/query.fcgi?db=Nucleotide&term=NM_023072) | hs\|19p13.13 |
| 0.008367 | 0.141 | 0.015873 | 0.61 | 1.56 | 2.56 |  |  |  | hs\|7p22.1 |
| 0.0025226 | 0.137 | 0.0238095 | 0.87 | 2.2 | 2.5 | [AP1S3](http://www.ncbi.nlm.nih.gov/entrez/query.fcgi?cmd=search&db=gene&term=AP1S3) | adaptor-related protein complex 1, sigma 3 subunit | [NM_001039569](http://www.ncbi.nlm.nih.gov/entrez/query.fcgi?db=Nucleotide&term=NM_001039569) | hs\|2q36.1 |
| 0.0057783 | 0.137 | 0.0238095 | 0.68 | 1.69 | 2.5 | [ASPHD2](http://www.ncbi.nlm.nih.gov/entrez/query.fcgi?cmd=search&db=gene&term=ASPHD2) | aspartate beta-hydroxylase domain containing 2 | [NM_020437](http://www.ncbi.nlm.nih.gov/entrez/query.fcgi?db=Nucleotide&term=NM_020437) | hs\|22q12.1 |
| 0.0011974 | 0.137 | 0.015873 | 0.74 | 1.85 | 2.5 | [CA13](http://www.ncbi.nlm.nih.gov/entrez/query.fcgi?cmd=search&db=gene&term=CA13) | carbonic anhydrase XIII | [NM_198584](http://www.ncbi.nlm.nih.gov/entrez/query.fcgi?db=Nucleotide&term=NM_198584) | hs\|8q21.2 |
| 0.0020145 | 0.137 | 0.015873 | 0.65 | 1.62 | 2.5 | [CDCP1](http://www.ncbi.nlm.nih.gov/entrez/query.fcgi?cmd=search&db=gene&term=CDCP1) | CUB domain containing protein 1 | [NM_022842](http://www.ncbi.nlm.nih.gov/entrez/query.fcgi?db=Nucleotide&term=NM_022842) | hs\|3p21.31 |
| 0.002301 | 0.137 | 0.015873 | 0.8 | 2.01 | 2.5 | [FGD6](http://www.ncbi.nlm.nih.gov/entrez/query.fcgi?cmd=search&db=gene&term=FGD6) | FYVE, RhoGEF and PH domain containing 6 | [NM_018351](http://www.ncbi.nlm.nih.gov/entrez/query.fcgi?db=Nucleotide&term=NM_018351) | hs\|12q22 |
| 0.0038874 | 0.137 | 0.015873 | 0.54 | 1.34 | 2.5 | [GPR132](http://www.ncbi.nlm.nih.gov/entrez/query.fcgi?cmd=search&db=gene&term=GPR132) | G protein-coupled receptor 132 | [NM_013345](http://www.ncbi.nlm.nih.gov/entrez/query.fcgi?db=Nucleotide&term=NM_013345) | hs\|14q32.33 |
| 0.0072525 | 0.139 | 0.015873 | 0.86 | 2.18 | 2.5 | [lnc-GPR137C-1](http://www.ncbi.nlm.nih.gov/entrez/query.fcgi?cmd=search&db=gene&term=lnc-GPR137C-1) | lnc-GPR137C-1:1 |  | hs\|14q22.1 |
| 0.0052486 | 0.137 | 0.0079365 | 0.67 | 1.66 | 2.5 | [MCU](http://www.ncbi.nlm.nih.gov/entrez/query.fcgi?cmd=search&db=gene&term=MCU) | mitochondrial calcium uniporter | [NM_138357](http://www.ncbi.nlm.nih.gov/entrez/query.fcgi?db=Nucleotide&term=NM_138357) | hs\|10q22.1 |
| 0.000538 | 0.137 | 0.0079365 | 0.7 | 1.76 | 2.5 | [MET](http://www.ncbi.nlm.nih.gov/entrez/query.fcgi?cmd=search&db=gene&term=MET) | MET proto-oncogene, receptor tyrosine kinase | [NM_000245](http://www.ncbi.nlm.nih.gov/entrez/query.fcgi?db=Nucleotide&term=NM_000245) | hs\|7q31.2 |
| 0.007023 | 0.138 | 0.015873 | 0.69 | 1.7 | 2.5 | [POF1B](http://www.ncbi.nlm.nih.gov/entrez/query.fcgi?cmd=search&db=gene&term=POF1B) | premature ovarian failure, 1B | [NM_024921](http://www.ncbi.nlm.nih.gov/entrez/query.fcgi?db=Nucleotide&term=NM_024921) | hs\|Xq21.1 |
| 0.0038136 | 0.137 | 0.0079365 | 0.62 | 1.55 | 2.5 | [RTP4](http://www.ncbi.nlm.nih.gov/entrez/query.fcgi?cmd=search&db=gene&term=RTP4) | receptor (chemosensory) transporter protein 4 | [NM_022147](http://www.ncbi.nlm.nih.gov/entrez/query.fcgi?db=Nucleotide&term=NM_022147) | hs\|3q27.3 |
| 0.0040252 | 0.137 | 0.0238095 | 0.79 | 1.96 | 2.5 | [S100A16](http://www.ncbi.nlm.nih.gov/entrez/query.fcgi?cmd=search&db=gene&term=S100A16) | S100 calcium binding protein A16 | [NM_080388](http://www.ncbi.nlm.nih.gov/entrez/query.fcgi?db=Nucleotide&term=NM_080388) | hs\|1q21.3 |
| 0.0055792 | 0.137 | 0.0238095 | 0.53 | 1.35 | 2.5 | [SLC38A6](http://www.ncbi.nlm.nih.gov/entrez/query.fcgi?cmd=search&db=gene&term=SLC38A6) | solute carrier family 38, member 6 | [NM_153811](http://www.ncbi.nlm.nih.gov/entrez/query.fcgi?db=Nucleotide&term=NM_153811) | hs\|14q23.1 |
| 0.004605 | 0.137 | 0.0079365 | 0.68 | 1.7 | 2.5 | [STIL](http://www.ncbi.nlm.nih.gov/entrez/query.fcgi?cmd=search&db=gene&term=STIL) | SCL/TAL1 interrupting locus | [NM_001282936](http://www.ncbi.nlm.nih.gov/entrez/query.fcgi?db=Nucleotide&term=NM_001282936) | hs\|1p33 |
| 0.0031085 | 0.137 | 0.0079365 | 0.67 | 1.62 | 2.44 | [CKLF](http://www.ncbi.nlm.nih.gov/entrez/query.fcgi?cmd=search&db=gene&term=CKLF) | chemokine-like factor | [NM_001040138](http://www.ncbi.nlm.nih.gov/entrez/query.fcgi?db=Nucleotide&term=NM_001040138) | hs\|16q21 |
| 0.0051303 | 0.137 | 0.0238095 | 0.55 | 1.36 | 2.44 | [GBP2](http://www.ncbi.nlm.nih.gov/entrez/query.fcgi?cmd=search&db=gene&term=GBP2) | guanylate binding protein 2, interferon-inducible | [NM_004120](http://www.ncbi.nlm.nih.gov/entrez/query.fcgi?db=Nucleotide&term=NM_004120) | hs\|1p22.2 |
| 0.0051019 | 0.137 | 0.0238095 | 0.7 | 1.7 | 2.44 | [lnc-CLEC2D-7](http://www.ncbi.nlm.nih.gov/entrez/query.fcgi?cmd=search&db=gene&term=lnc-CLEC2D-7) | lnc-CLEC2D-7:1 |  | hs\|12p13.31 |
| 0.0073112 | 0.139 | 0.0079365 | 0.66 | 1.6 | 2.44 | [LOC102723465](http://www.ncbi.nlm.nih.gov/entrez/query.fcgi?cmd=search&db=gene&term=LOC102723465) | uncharacterized LOC102723465 | [XR_425055](http://www.ncbi.nlm.nih.gov/entrez/query.fcgi?db=Nucleotide&term=XR_425055) | hs\|1q32.1 |
| 0.0055642 | 0.137 | 0.0238095 | 0.58 | 1.42 | 2.44 | [LXN](http://www.ncbi.nlm.nih.gov/entrez/query.fcgi?cmd=search&db=gene&term=LXN) | latexin | [NM_020169](http://www.ncbi.nlm.nih.gov/entrez/query.fcgi?db=Nucleotide&term=NM_020169) | hs\|3q25.32 |
| 0.0010233 | 0.137 | 0.0079365 | 0.64 | 1.55 | 2.44 | [TNFAIP3](http://www.ncbi.nlm.nih.gov/entrez/query.fcgi?cmd=search&db=gene&term=TNFAIP3) | tumor necrosis factor, alpha-induced protein 3 | [NM_006290](http://www.ncbi.nlm.nih.gov/entrez/query.fcgi?db=Nucleotide&term=NM_006290) | hs\|6q23.3 |
| 0.0052676 | 0.137 | 0.0238095 | 0.73 | 1.74 | 2.38 | [ANXA2](http://www.ncbi.nlm.nih.gov/entrez/query.fcgi?cmd=search&db=gene&term=ANXA2) | annexin A2 | [NM_001002857](http://www.ncbi.nlm.nih.gov/entrez/query.fcgi?db=Nucleotide&term=NM_001002857) | hs\|15q22.2 |
| 0.0067551 | 0.137 | 0.031746 | 0.68 | 1.6 | 2.38 | [AZIN2](http://www.ncbi.nlm.nih.gov/entrez/query.fcgi?cmd=search&db=gene&term=AZIN2) | antizyme inhibitor 2 | [NM_052998](http://www.ncbi.nlm.nih.gov/entrez/query.fcgi?db=Nucleotide&term=NM_052998) | hs\|1p35.1 |
| 0.006526 | 0.137 | 0.015873 | 0.71 | 1.68 | 2.38 | [ETV1](http://www.ncbi.nlm.nih.gov/entrez/query.fcgi?cmd=search&db=gene&term=ETV1) | ets variant 1 | [NM_004956](http://www.ncbi.nlm.nih.gov/entrez/query.fcgi?db=Nucleotide&term=NM_004956) | hs\|7p21.2 |
| 0.0037217 | 0.137 | 0.0079365 | 0.8 | 1.93 | 2.38 | [ITGB4](http://www.ncbi.nlm.nih.gov/entrez/query.fcgi?cmd=search&db=gene&term=ITGB4) | integrin, beta 4 | [NM_000213](http://www.ncbi.nlm.nih.gov/entrez/query.fcgi?db=Nucleotide&term=NM_000213) | hs\|17q25.1 |
| 0.0030794 | 0.137 | 0.0079365 | 0.62 | 1.47 | 2.38 | [lnc-CDIPT-1](http://www.ncbi.nlm.nih.gov/entrez/query.fcgi?cmd=search&db=gene&term=lnc-CDIPT-1) | lnc-CDIPT-1:7 | [AK097472](http://www.ncbi.nlm.nih.gov/entrez/query.fcgi?db=Nucleotide&term=AK097472) | hs\|16p11.2 |
| 0.0074666 | 0.139 | 0.031746 | 0.7 | 1.65 | 2.38 | [LRFN4](http://www.ncbi.nlm.nih.gov/entrez/query.fcgi?cmd=search&db=gene&term=LRFN4) | leucine rich repeat and fibronectin type III domain containing 4 | [NM_024036](http://www.ncbi.nlm.nih.gov/entrez/query.fcgi?db=Nucleotide&term=NM_024036) | hs\|11q13.2 |
| 0.0026659 | 0.137 | 0.0079365 | 0.72 | 1.72 | 2.38 | [NCEH1](http://www.ncbi.nlm.nih.gov/entrez/query.fcgi?cmd=search&db=gene&term=NCEH1) | neutral cholesterol ester hydrolase 1 | [NM_020792](http://www.ncbi.nlm.nih.gov/entrez/query.fcgi?db=Nucleotide&term=NM_020792) | hs\|3q26.31 |
| 0.0063127 | 0.137 | 0.015873 | 0.66 | 1.57 | 2.38 | [OR7C2](http://www.ncbi.nlm.nih.gov/entrez/query.fcgi?cmd=search&db=gene&term=OR7C2) | olfactory receptor, family 7, subfamily C, member 2 | [NM_012377](http://www.ncbi.nlm.nih.gov/entrez/query.fcgi?db=Nucleotide&term=NM_012377) | hs\|19p13.12 |
| 0.0051855 | 0.137 | 0.015873 | 0.75 | 1.8 | 2.38 | [VASP](http://www.ncbi.nlm.nih.gov/entrez/query.fcgi?cmd=search&db=gene&term=VASP) | vasodilator-stimulated phosphoprotein | [NM_003370](http://www.ncbi.nlm.nih.gov/entrez/query.fcgi?db=Nucleotide&term=NM_003370) | hs\|19q13.32 |
| 0.005616 | 0.137 | 0.015873 | 0.71 | 1.64 | 2.33 | [ABCC3](http://www.ncbi.nlm.nih.gov/entrez/query.fcgi?cmd=search&db=gene&term=ABCC3) | ATP-binding cassette, sub-family C (CFTR/MRP), member 3 | [NM_001144070](http://www.ncbi.nlm.nih.gov/entrez/query.fcgi?db=Nucleotide&term=NM_001144070) | hs\|17q21.33 |
| 0.0018072 | 0.137 | 0.0079365 | 0.61 | 1.41 | 2.33 | [AFAP1](http://www.ncbi.nlm.nih.gov/entrez/query.fcgi?cmd=search&db=gene&term=AFAP1) | actin filament associated protein 1 | [NM_001134647](http://www.ncbi.nlm.nih.gov/entrez/query.fcgi?db=Nucleotide&term=NM_001134647) | hs\|4p16.1 |
| 8.66E-05 | 0.137 | 0.0079365 | 0.63 | 1.47 | 2.33 | [CLDN16](http://www.ncbi.nlm.nih.gov/entrez/query.fcgi?cmd=search&db=gene&term=CLDN16) | claudin 16 | [NM_006580](http://www.ncbi.nlm.nih.gov/entrez/query.fcgi?db=Nucleotide&term=NM_006580) | hs\|3q28 |
| 0.0034929 | 0.137 | 0.015873 | 0.68 | 1.58 | 2.33 | [IER3](http://www.ncbi.nlm.nih.gov/entrez/query.fcgi?cmd=search&db=gene&term=IER3) | immediate early response 3 | [NM_003897](http://www.ncbi.nlm.nih.gov/entrez/query.fcgi?db=Nucleotide&term=NM_003897) | hs\|6p21.33 |
| 0.0061068 | 0.137 | 0.015873 | 0.74 | 1.72 | 2.33 | [PKM](http://www.ncbi.nlm.nih.gov/entrez/query.fcgi?cmd=search&db=gene&term=PKM) | pyruvate kinase, muscle | [NM_182470](http://www.ncbi.nlm.nih.gov/entrez/query.fcgi?db=Nucleotide&term=NM_182470) | hs\|15q23 |
| 0.0064829 | 0.137 | 0.015873 | 0.72 | 1.68 | 2.33 | [TBXAS1](http://www.ncbi.nlm.nih.gov/entrez/query.fcgi?cmd=search&db=gene&term=TBXAS1) | thromboxane A synthase 1 (platelet) | [NM_001166254](http://www.ncbi.nlm.nih.gov/entrez/query.fcgi?db=Nucleotide&term=NM_001166254) | hs\|7q34 |
| 0.001933 | 0.137 | 0.0079365 | 0.66 | 1.55 | 2.33 | [TIAM2](http://www.ncbi.nlm.nih.gov/entrez/query.fcgi?cmd=search&db=gene&term=TIAM2) | T-cell lymphoma invasion and metastasis 2 | [NM_012454](http://www.ncbi.nlm.nih.gov/entrez/query.fcgi?db=Nucleotide&term=NM_012454) | hs\|6q25.3 |
| 0.0071273 | 0.138 | 0.015873 | 0.75 | 1.7 | 2.27 | [BCL2A1](http://www.ncbi.nlm.nih.gov/entrez/query.fcgi?cmd=search&db=gene&term=BCL2A1) | BCL2-related protein A1 | [NM_004049](http://www.ncbi.nlm.nih.gov/entrez/query.fcgi?db=Nucleotide&term=NM_004049) | hs\|15q25.1 |
| 0.005489 | 0.137 | 0.0238095 | 0.63 | 1.43 | 2.27 | [CHN1](http://www.ncbi.nlm.nih.gov/entrez/query.fcgi?cmd=search&db=gene&term=CHN1) | chimerin 1 | [NM_001822](http://www.ncbi.nlm.nih.gov/entrez/query.fcgi?db=Nucleotide&term=NM_001822) | hs\|2q31.1 |
| 0.0057313 | 0.137 | 0.015873 | 0.78 | 1.78 | 2.27 | [EFNB2](http://www.ncbi.nlm.nih.gov/entrez/query.fcgi?cmd=search&db=gene&term=EFNB2) | ephrin-B2 | [NM_004093](http://www.ncbi.nlm.nih.gov/entrez/query.fcgi?db=Nucleotide&term=NM_004093) | hs\|13q33.3 |
| 0.0034737 | 0.137 | 0.015873 | 0.62 | 1.42 | 2.27 | [GCHFR](http://www.ncbi.nlm.nih.gov/entrez/query.fcgi?cmd=search&db=gene&term=GCHFR) | GTP cyclohydrolase I feedback regulator | [NM_005258](http://www.ncbi.nlm.nih.gov/entrez/query.fcgi?db=Nucleotide&term=NM_005258) | hs\|15q15.1 |
| 0.0038696 | 0.137 | 0.0079365 | 0.59 | 1.35 | 2.27 | [HOXA3](http://www.ncbi.nlm.nih.gov/entrez/query.fcgi?cmd=search&db=gene&term=HOXA3) | homeobox A3 | [NM_153631](http://www.ncbi.nlm.nih.gov/entrez/query.fcgi?db=Nucleotide&term=NM_153631) | hs\|7p15.2 |
| 0.0026635 | 0.137 | 0.0238095 | 0.78 | 1.76 | 2.27 | [IGF2BP2](http://www.ncbi.nlm.nih.gov/entrez/query.fcgi?cmd=search&db=gene&term=IGF2BP2) | insulin-like growth factor 2 mRNA binding protein 2 | [NM_006548](http://www.ncbi.nlm.nih.gov/entrez/query.fcgi?db=Nucleotide&term=NM_006548) | hs\|3q27.2 |
| 0.0092754 | 0.143 | 0.031746 | 0.81 | 1.83 | 2.27 | [KCNE3](http://www.ncbi.nlm.nih.gov/entrez/query.fcgi?cmd=search&db=gene&term=KCNE3) | potassium channel, voltage gated subfamily E regulatory beta subunit 3 | [NM_005472](http://www.ncbi.nlm.nih.gov/entrez/query.fcgi?db=Nucleotide&term=NM_005472) | hs\|11q13.4 |
| 0.0062398 | 0.137 | 0.0238095 | 0.63 | 1.44 | 2.27 | [LAD1](http://www.ncbi.nlm.nih.gov/entrez/query.fcgi?cmd=search&db=gene&term=LAD1) | ladinin 1 | [NM_005558](http://www.ncbi.nlm.nih.gov/entrez/query.fcgi?db=Nucleotide&term=NM_005558) | hs\|1q32.1 |
| 0.0005309 | 0.137 | 0.0079365 | 0.72 | 1.62 | 2.27 | [LBH](http://www.ncbi.nlm.nih.gov/entrez/query.fcgi?cmd=search&db=gene&term=LBH) | limb bud and heart development | [NM_030915](http://www.ncbi.nlm.nih.gov/entrez/query.fcgi?db=Nucleotide&term=NM_030915) | hs\|2p23.1 |
| 0.0025456 | 0.137 | 0.015873 | 0.78 | 1.76 | 2.27 | [LIMK1](http://www.ncbi.nlm.nih.gov/entrez/query.fcgi?cmd=search&db=gene&term=LIMK1) | LIM domain kinase 1 | [NM_002314](http://www.ncbi.nlm.nih.gov/entrez/query.fcgi?db=Nucleotide&term=NM_002314) | hs\|7q11.23 |
| 0.0094741 | 0.143 | 0.0238095 | 0.77 | 1.77 | 2.27 | [LOC101927070](http://www.ncbi.nlm.nih.gov/entrez/query.fcgi?cmd=search&db=gene&term=LOC101927070) | uncharacterized LOC101927070 | [NR_126337](http://www.ncbi.nlm.nih.gov/entrez/query.fcgi?db=Nucleotide&term=NR_126337) | hs\|2q11.2 |
| 0.0035281 | 0.137 | 0.015873 | 0.69 | 1.57 | 2.27 | [PML](http://www.ncbi.nlm.nih.gov/entrez/query.fcgi?cmd=search&db=gene&term=PML) | promyelocytic leukemia | [NM_033238](http://www.ncbi.nlm.nih.gov/entrez/query.fcgi?db=Nucleotide&term=NM_033238) | hs\|15q24.1 |
| 0.0093457 | 0.143 | 0.0238095 | 0.65 | 1.47 | 2.27 | [RRN3P2](http://www.ncbi.nlm.nih.gov/entrez/query.fcgi?cmd=search&db=gene&term=RRN3P2) | RNA polymerase I transcription factor homolog (S. cerevisiae) pseudogene 2 | [NR_003369](http://www.ncbi.nlm.nih.gov/entrez/query.fcgi?db=Nucleotide&term=NR_003369) | hs\|16p11.2 |
| 0.0050929 | 0.137 | 0.0238095 | 0.61 | 1.37 | 2.27 | [SGK223](http://www.ncbi.nlm.nih.gov/entrez/query.fcgi?cmd=search&db=gene&term=SGK223) | homolog of rat pragma of Rnd2 | [NM_001080826](http://www.ncbi.nlm.nih.gov/entrez/query.fcgi?db=Nucleotide&term=NM_001080826) | hs\|8p23.1 |
| 0.001329 | 0.137 | 0.0079365 | 0.67 | 1.52 | 2.27 | [TNFRSF10A](http://www.ncbi.nlm.nih.gov/entrez/query.fcgi?cmd=search&db=gene&term=TNFRSF10A) | tumor necrosis factor receptor superfamily, member 10a | [NM_003844](http://www.ncbi.nlm.nih.gov/entrez/query.fcgi?db=Nucleotide&term=NM_003844) | hs\|8p21.3 |
| 0.0044955 | 0.137 | 0.0238095 | 0.56 | 1.27 | 2.27 |  |  |  | hs\|8q22.3 |
| 0.004002 | 0.137 | 0.015873 | 0.7 | 1.55 | 2.22 | [ABRACL](http://www.ncbi.nlm.nih.gov/entrez/query.fcgi?cmd=search&db=gene&term=ABRACL) | ABRA C-terminal like | [NM_021243](http://www.ncbi.nlm.nih.gov/entrez/query.fcgi?db=Nucleotide&term=NM_021243) | hs\|6q24.1 |
| 0.0078752 | 0.14 | 0.0238095 | 0.72 | 1.59 | 2.22 | [APOL2](http://www.ncbi.nlm.nih.gov/entrez/query.fcgi?cmd=search&db=gene&term=APOL2) | apolipoprotein L, 2 | [NM_145637](http://www.ncbi.nlm.nih.gov/entrez/query.fcgi?db=Nucleotide&term=NM_145637) | hs\|22q12.3 |
| 0.0018067 | 0.137 | 0.0079365 | 0.74 | 1.65 | 2.22 | [CYB5R2](http://www.ncbi.nlm.nih.gov/entrez/query.fcgi?cmd=search&db=gene&term=CYB5R2) | cytochrome b5 reductase 2 | [NM_016229](http://www.ncbi.nlm.nih.gov/entrez/query.fcgi?db=Nucleotide&term=NM_016229) | hs\|11p15.4 |
| 0.0025697 | 0.137 | 0.0079365 | 0.61 | 1.35 | 2.22 | [DUSP5](http://www.ncbi.nlm.nih.gov/entrez/query.fcgi?cmd=search&db=gene&term=DUSP5) | dual specificity phosphatase 5 | [NM_004419](http://www.ncbi.nlm.nih.gov/entrez/query.fcgi?db=Nucleotide&term=NM_004419) | hs\|10q25.2 |
| 0.0078852 | 0.14 | 0.0238095 | 0.69 | 1.54 | 2.22 | [EFNB1](http://www.ncbi.nlm.nih.gov/entrez/query.fcgi?cmd=search&db=gene&term=EFNB1) | ephrin-B1 | [NM_004429](http://www.ncbi.nlm.nih.gov/entrez/query.fcgi?db=Nucleotide&term=NM_004429) | hs\|Xq13.1 |
| 0.0023278 | 0.137 | 0.015873 | 0.64 | 1.43 | 2.22 | [HENMT1](http://www.ncbi.nlm.nih.gov/entrez/query.fcgi?cmd=search&db=gene&term=HENMT1) | HEN1 methyltransferase homolog 1 (Arabidopsis) | [NM_144584](http://www.ncbi.nlm.nih.gov/entrez/query.fcgi?db=Nucleotide&term=NM_144584) | hs\|1p13.3 |
| 0.0092653 | 0.143 | 0.0238095 | 0.7 | 1.56 | 2.22 | [PARPBP](http://www.ncbi.nlm.nih.gov/entrez/query.fcgi?cmd=search&db=gene&term=PARPBP) | PARP1 binding protein | [NM_017915](http://www.ncbi.nlm.nih.gov/entrez/query.fcgi?db=Nucleotide&term=NM_017915) | hs\|12q23.2 |
| 0.0050467 | 0.137 | 0.031746 | 0.82 | 1.84 | 2.22 | [PGM2L1](http://www.ncbi.nlm.nih.gov/entrez/query.fcgi?cmd=search&db=gene&term=PGM2L1) | phosphoglucomutase 2-like 1 | [NM_173582](http://www.ncbi.nlm.nih.gov/entrez/query.fcgi?db=Nucleotide&term=NM_173582) | hs\|11q13.4 |
| 0.0025331 | 0.137 | 0.0079365 | 0.69 | 1.53 | 2.22 | [PMEPA1](http://www.ncbi.nlm.nih.gov/entrez/query.fcgi?cmd=search&db=gene&term=PMEPA1) | prostate transmembrane protein, androgen induced 1 | [NM_020182](http://www.ncbi.nlm.nih.gov/entrez/query.fcgi?db=Nucleotide&term=NM_020182) | hs\|20q13.31 |
| 0.0002778 | 0.137 | 0.0079365 | 0.66 | 1.45 | 2.22 | [RASGEF1B](http://www.ncbi.nlm.nih.gov/entrez/query.fcgi?cmd=search&db=gene&term=RASGEF1B) | RasGEF domain family, member 1B | [NM_152545](http://www.ncbi.nlm.nih.gov/entrez/query.fcgi?db=Nucleotide&term=NM_152545) | hs\|4q21.21 |
| 0.0039949 | 0.137 | 0.0079365 | 0.67 | 1.48 | 2.22 | [SLC7A7](http://www.ncbi.nlm.nih.gov/entrez/query.fcgi?cmd=search&db=gene&term=SLC7A7) | solute carrier family 7 (amino acid transporter light chain, y+L system), member 7 | [NM_001126106](http://www.ncbi.nlm.nih.gov/entrez/query.fcgi?db=Nucleotide&term=NM_001126106) | hs\|14q11.2 |
| 0.0047858 | 0.137 | 0.015873 | 0.65 | 1.42 | 2.22 | [STARD4](http://www.ncbi.nlm.nih.gov/entrez/query.fcgi?cmd=search&db=gene&term=STARD4) | StAR-related lipid transfer (START) domain containing 4 | [NM_139164](http://www.ncbi.nlm.nih.gov/entrez/query.fcgi?db=Nucleotide&term=NM_139164) | hs\|5q22.1 |
| 0.0068934 | 0.138 | 0.015873 | 0.66 | 1.46 | 2.22 | [STX6](http://www.ncbi.nlm.nih.gov/entrez/query.fcgi?cmd=search&db=gene&term=STX6) | syntaxin 6 | [NM_005819](http://www.ncbi.nlm.nih.gov/entrez/query.fcgi?db=Nucleotide&term=NM_005819) | hs\|1q25.3 |
| 0.003478 | 0.137 | 0.015873 | 0.73 | 1.63 | 2.22 | [TSKU](http://www.ncbi.nlm.nih.gov/entrez/query.fcgi?cmd=search&db=gene&term=TSKU) | tsukushi, small leucine rich proteoglycan | [NM_015516](http://www.ncbi.nlm.nih.gov/entrez/query.fcgi?db=Nucleotide&term=NM_015516) | hs\|11q13.5 |
| 0.008448 | 0.141 | 0.031746 | 0.72 | 1.57 | 2.17 | [AKR1C3](http://www.ncbi.nlm.nih.gov/entrez/query.fcgi?cmd=search&db=gene&term=AKR1C3) | aldo-keto reductase family 1, member C3 | [NM_003739](http://www.ncbi.nlm.nih.gov/entrez/query.fcgi?db=Nucleotide&term=NM_003739) | hs\|10p15.1 |
| 0.0093827 | 0.143 | 0.031746 | 0.78 | 1.71 | 2.17 | [ANXA2P3](http://www.ncbi.nlm.nih.gov/entrez/query.fcgi?cmd=search&db=gene&term=ANXA2P3) | annexin A2 pseudogene 3 | [NR_001446](http://www.ncbi.nlm.nih.gov/entrez/query.fcgi?db=Nucleotide&term=NR_001446) | hs\|10q21.3 |
| 0.0013165 | 0.137 | 0.0079365 | 0.68 | 1.5 | 2.17 | [C11orf80](http://www.ncbi.nlm.nih.gov/entrez/query.fcgi?cmd=search&db=gene&term=C11orf80) | chromosome 11 open reading frame 80 | [NM_024650](http://www.ncbi.nlm.nih.gov/entrez/query.fcgi?db=Nucleotide&term=NM_024650) | hs\|11q13.2 |
| 0.0046033 | 0.137 | 0.015873 | 0.77 | 1.66 | 2.17 | [CDR2L](http://www.ncbi.nlm.nih.gov/entrez/query.fcgi?cmd=search&db=gene&term=CDR2L) | cerebellar degeneration-related protein 2-like | [NM_014603](http://www.ncbi.nlm.nih.gov/entrez/query.fcgi?db=Nucleotide&term=NM_014603) | hs\|17q25.1 |
| 0.0052724 | 0.137 | 0.0079365 | 0.79 | 1.7 | 2.17 | [CXCL16](http://www.ncbi.nlm.nih.gov/entrez/query.fcgi?cmd=search&db=gene&term=CXCL16) | chemokine (C-X-C motif) ligand 16 | [NM_001100812](http://www.ncbi.nlm.nih.gov/entrez/query.fcgi?db=Nucleotide&term=NM_001100812) | hs\|17p13.2 |
| 0.0065529 | 0.137 | 0.0238095 | 0.6 | 1.31 | 2.17 | [DNM3OS](http://www.ncbi.nlm.nih.gov/entrez/query.fcgi?cmd=search&db=gene&term=DNM3OS) | DNM3 opposite strand/antisense RNA | [NR_038397](http://www.ncbi.nlm.nih.gov/entrez/query.fcgi?db=Nucleotide&term=NR_038397) | hs\|1q24.3 |
| 0.0092377 | 0.143 | 0.015873 | 0.8 | 1.75 | 2.17 | [FAT1](http://www.ncbi.nlm.nih.gov/entrez/query.fcgi?cmd=search&db=gene&term=FAT1) | FAT atypical cadherin 1 | [NM_005245](http://www.ncbi.nlm.nih.gov/entrez/query.fcgi?db=Nucleotide&term=NM_005245) | hs\|4q35.2 |
| 0.001249 | 0.137 | 0.0079365 | 0.83 | 1.8 | 2.17 | [LETM2](http://www.ncbi.nlm.nih.gov/entrez/query.fcgi?cmd=search&db=gene&term=LETM2) | leucine zipper-EF-hand containing transmembrane protein 2 | [NM_144652](http://www.ncbi.nlm.nih.gov/entrez/query.fcgi?db=Nucleotide&term=NM_144652) | hs\|8p11.23 |
| 0.0050117 | 0.137 | 0.0238095 | 0.73 | 1.59 | 2.17 | [PML](http://www.ncbi.nlm.nih.gov/entrez/query.fcgi?cmd=search&db=gene&term=PML) | promyelocytic leukemia | [NM_033247](http://www.ncbi.nlm.nih.gov/entrez/query.fcgi?db=Nucleotide&term=NM_033247) | hs\|15q24.1 |
| 0.0096755 | 0.144 | 0.031746 | 0.59 | 1.29 | 2.17 | [RGS1](http://www.ncbi.nlm.nih.gov/entrez/query.fcgi?cmd=search&db=gene&term=RGS1) | regulator of G-protein signaling 1 | [NM_002922](http://www.ncbi.nlm.nih.gov/entrez/query.fcgi?db=Nucleotide&term=NM_002922) | hs\|1q31.2 |
| 0.0058408 | 0.137 | 0.015873 | 0.7 | 1.53 | 2.17 | [RHPN2](http://www.ncbi.nlm.nih.gov/entrez/query.fcgi?cmd=search&db=gene&term=RHPN2) | rhophilin, Rho GTPase binding protein 2 | [NM_033103](http://www.ncbi.nlm.nih.gov/entrez/query.fcgi?db=Nucleotide&term=NM_033103) | hs\|19q13.11 |
| 0.0046373 | 0.137 | 0.015873 | 0.63 | 1.37 | 2.17 | [SPON2](http://www.ncbi.nlm.nih.gov/entrez/query.fcgi?cmd=search&db=gene&term=SPON2) | spondin 2, extracellular matrix protein | [NM_012445](http://www.ncbi.nlm.nih.gov/entrez/query.fcgi?db=Nucleotide&term=NM_012445) | hs\|4p16.3 |
| 0.002339 | 0.137 | 0.0079365 | 0.64 | 1.4 | 2.17 | [STEAP1](http://www.ncbi.nlm.nih.gov/entrez/query.fcgi?cmd=search&db=gene&term=STEAP1) | six transmembrane epithelial antigen of the prostate 1 | [NM_012449](http://www.ncbi.nlm.nih.gov/entrez/query.fcgi?db=Nucleotide&term=NM_012449) | hs\|7q21.13 |
| 0.0069756 | 0.138 | 0.0238095 | 0.7 | 1.54 | 2.17 | [XLOC_l2_007456](http://www.ncbi.nlm.nih.gov/entrez/query.fcgi?cmd=search&db=gene&term=XLOC_l2_007456) |  |  | hs\|2q11.2 |
| 0.0090724 | 0.143 | 0.031746 | 0.75 | 1.59 | 2.13 | [ANXA2](http://www.ncbi.nlm.nih.gov/entrez/query.fcgi?cmd=search&db=gene&term=ANXA2) | annexin A2 | [NM_001002857](http://www.ncbi.nlm.nih.gov/entrez/query.fcgi?db=Nucleotide&term=NM_001002857) | hs\|15q22.2 |
| 0.0046312 | 0.137 | 0.0079365 | 0.67 | 1.44 | 2.13 | [BPNT1](http://www.ncbi.nlm.nih.gov/entrez/query.fcgi?cmd=search&db=gene&term=BPNT1) | 3'(2'), 5'-bisphosphate nucleotidase 1 | [NM_006085](http://www.ncbi.nlm.nih.gov/entrez/query.fcgi?db=Nucleotide&term=NM_006085) | hs\|1q41 |
| 0.0030611 | 0.137 | 0.0079365 | 0.68 | 1.44 | 2.13 | [C8orf31](http://www.ncbi.nlm.nih.gov/entrez/query.fcgi?cmd=search&db=gene&term=C8orf31) | chromosome 8 open reading frame 31 | [NM_173687](http://www.ncbi.nlm.nih.gov/entrez/query.fcgi?db=Nucleotide&term=NM_173687) | hs\|8q24.3 |
| 0.0056891 | 0.137 | 0.0079365 | 0.72 | 1.53 | 2.13 | [DSC2](http://www.ncbi.nlm.nih.gov/entrez/query.fcgi?cmd=search&db=gene&term=DSC2) | desmocollin 2 | [NM_024422](http://www.ncbi.nlm.nih.gov/entrez/query.fcgi?db=Nucleotide&term=NM_024422) | hs\|18q12.1 |
| 0.0050463 | 0.137 | 0.015873 | 0.74 | 1.56 | 2.13 | [EFNA4](http://www.ncbi.nlm.nih.gov/entrez/query.fcgi?cmd=search&db=gene&term=EFNA4) | ephrin-A4 | [NM_182690](http://www.ncbi.nlm.nih.gov/entrez/query.fcgi?db=Nucleotide&term=NM_182690) | hs\|1q22 |
| 0.0096789 | 0.144 | 0.0238095 | 0.64 | 1.38 | 2.13 | [GBP3](http://www.ncbi.nlm.nih.gov/entrez/query.fcgi?cmd=search&db=gene&term=GBP3) | guanylate binding protein 3 | [NM_018284](http://www.ncbi.nlm.nih.gov/entrez/query.fcgi?db=Nucleotide&term=NM_018284) | hs\|1p22.2 |
| 0.0092969 | 0.143 | 0.0238095 | 0.67 | 1.42 | 2.13 | [HEPH](http://www.ncbi.nlm.nih.gov/entrez/query.fcgi?cmd=search&db=gene&term=HEPH) | hephaestin | [NM_014799](http://www.ncbi.nlm.nih.gov/entrez/query.fcgi?db=Nucleotide&term=NM_014799) | hs\|Xq12 |
| 0.0028424 | 0.137 | 0.015873 | 0.66 | 1.42 | 2.13 | [IER3](http://www.ncbi.nlm.nih.gov/entrez/query.fcgi?cmd=search&db=gene&term=IER3) | immediate early response 3 | [NM_003897](http://www.ncbi.nlm.nih.gov/entrez/query.fcgi?db=Nucleotide&term=NM_003897) | hs\|6p21.33 |
| 0.0067228 | 0.137 | 0.0238095 | 0.72 | 1.52 | 2.13 | [IL4R](http://www.ncbi.nlm.nih.gov/entrez/query.fcgi?cmd=search&db=gene&term=IL4R) | interleukin 4 receptor | [NM_000418](http://www.ncbi.nlm.nih.gov/entrez/query.fcgi?db=Nucleotide&term=NM_000418) | hs\|16p12.1 |
| 0.0096531 | 0.144 | 0.0079365 | 0.76 | 1.6 | 2.13 | [NFKBIE](http://www.ncbi.nlm.nih.gov/entrez/query.fcgi?cmd=search&db=gene&term=NFKBIE) | nuclear factor of kappa light polypeptide gene enhancer in B-cells inhibitor, epsilon | [NM_004556](http://www.ncbi.nlm.nih.gov/entrez/query.fcgi?db=Nucleotide&term=NM_004556) | hs\|6p21.1 |
| 0.0030694 | 0.137 | 0.0079365 | 0.71 | 1.53 | 2.13 | [OCIAD2](http://www.ncbi.nlm.nih.gov/entrez/query.fcgi?cmd=search&db=gene&term=OCIAD2) | OCIA domain containing 2 | [NM_001014446](http://www.ncbi.nlm.nih.gov/entrez/query.fcgi?db=Nucleotide&term=NM_001014446) | hs\|4p11 |
| 0.0018754 | 0.137 | 0.015873 | 0.71 | 1.52 | 2.13 | [PLXDC1](http://www.ncbi.nlm.nih.gov/entrez/query.fcgi?cmd=search&db=gene&term=PLXDC1) | plexin domain containing 1 | [NM_020405](http://www.ncbi.nlm.nih.gov/entrez/query.fcgi?db=Nucleotide&term=NM_020405) | hs\|17q12 |
| 0.006312 | 0.137 | 0.0396825 | 0.85 | 1.81 | 2.13 | [S100A2](http://www.ncbi.nlm.nih.gov/entrez/query.fcgi?cmd=search&db=gene&term=S100A2) | S100 calcium binding protein A2 | [NM_005978](http://www.ncbi.nlm.nih.gov/entrez/query.fcgi?db=Nucleotide&term=NM_005978) | hs\|1q21.3 |
| 0.0088626 | 0.142 | 0.015873 | 0.71 | 1.53 | 2.13 | [SAMD9L](http://www.ncbi.nlm.nih.gov/entrez/query.fcgi?cmd=search&db=gene&term=SAMD9L) | sterile alpha motif domain containing 9-like | [XM_005250193](http://www.ncbi.nlm.nih.gov/entrez/query.fcgi?db=Nucleotide&term=XM_005250193) | hs\|7q21.2 |
| 0.0086459 | 0.142 | 0.0238095 | 0.71 | 1.51 | 2.13 | [TRAF1](http://www.ncbi.nlm.nih.gov/entrez/query.fcgi?cmd=search&db=gene&term=TRAF1) | TNF receptor-associated factor 1 | [NM_005658](http://www.ncbi.nlm.nih.gov/entrez/query.fcgi?db=Nucleotide&term=NM_005658) | hs\|9q33.2 |
| 0.009164 | 0.143 | 0.031746 | 0.61 | 1.3 | 2.13 | [TRANK1](http://www.ncbi.nlm.nih.gov/entrez/query.fcgi?cmd=search&db=gene&term=TRANK1) | tetratricopeptide repeat and ankyrin repeat containing 1 | [NM_014831](http://www.ncbi.nlm.nih.gov/entrez/query.fcgi?db=Nucleotide&term=NM_014831) | hs\|3p22.2 |
| 0.0078479 | 0.14 | 0.0238095 | 0.79 | 1.69 | 2.13 | [XRCC4](http://www.ncbi.nlm.nih.gov/entrez/query.fcgi?cmd=search&db=gene&term=XRCC4) | X-ray repair complementing defective repair in Chinese hamster cells 4 | [NM_022550](http://www.ncbi.nlm.nih.gov/entrez/query.fcgi?db=Nucleotide&term=NM_022550) | hs\|5q14.2 |
| 0.0089531 | 0.142 | 0.015873 | 0.75 | 1.58 | 2.08 | [AK1](http://www.ncbi.nlm.nih.gov/entrez/query.fcgi?cmd=search&db=gene&term=AK1) | adenylate kinase 1 | [NM_000476](http://www.ncbi.nlm.nih.gov/entrez/query.fcgi?db=Nucleotide&term=NM_000476) | hs\|9q34.11 |
| 0.005473 | 0.137 | 0.0079365 | 0.59 | 1.24 | 2.08 | [CCDC74B](http://www.ncbi.nlm.nih.gov/entrez/query.fcgi?cmd=search&db=gene&term=CCDC74B) | coiled-coil domain containing 74B | [NM_207310](http://www.ncbi.nlm.nih.gov/entrez/query.fcgi?db=Nucleotide&term=NM_207310) | hs\|2q21.1 |
| 0.0071134 | 0.138 | 0.015873 | 0.62 | 1.3 | 2.08 | [CRYM](http://www.ncbi.nlm.nih.gov/entrez/query.fcgi?cmd=search&db=gene&term=CRYM) | crystallin, mu | [NM_001888](http://www.ncbi.nlm.nih.gov/entrez/query.fcgi?db=Nucleotide&term=NM_001888) | hs\|16p12.2 |
| 0.0026301 | 0.137 | 0.015873 | 0.7 | 1.45 | 2.08 | [EDARADD](http://www.ncbi.nlm.nih.gov/entrez/query.fcgi?cmd=search&db=gene&term=EDARADD) | EDAR-associated death domain | [NM_080738](http://www.ncbi.nlm.nih.gov/entrez/query.fcgi?db=Nucleotide&term=NM_080738) | hs\|1q43 |
| 0.0016674 | 0.137 | 0.0079365 | 0.74 | 1.53 | 2.08 | [ITPR3](http://www.ncbi.nlm.nih.gov/entrez/query.fcgi?cmd=search&db=gene&term=ITPR3) | inositol 1,4,5-trisphosphate receptor, type 3 | [NM_002224](http://www.ncbi.nlm.nih.gov/entrez/query.fcgi?db=Nucleotide&term=NM_002224) | hs\|6p21.31 |
| 0.0059776 | 0.137 | 0.031746 | 0.82 | 1.7 | 2.08 | [LINC00920](http://www.ncbi.nlm.nih.gov/entrez/query.fcgi?cmd=search&db=gene&term=LINC00920) | long intergenic non-protein coding RNA 920 |  | hs\|16q21 |
| 0.0083674 | 0.141 | 0.0238095 | 0.78 | 1.63 | 2.08 | [LOC102724094](http://www.ncbi.nlm.nih.gov/entrez/query.fcgi?cmd=search&db=gene&term=LOC102724094) | uncharacterized LOC102724094 | [NR_120519](http://www.ncbi.nlm.nih.gov/entrez/query.fcgi?db=Nucleotide&term=NR_120519) | hs\|7q22.1 |
| 0.0005744 | 0.137 | 0.0079365 | 0.74 | 1.55 | 2.08 | [LPCAT4](http://www.ncbi.nlm.nih.gov/entrez/query.fcgi?cmd=search&db=gene&term=LPCAT4) | lysophosphatidylcholine acyltransferase 4 | [NM_153613](http://www.ncbi.nlm.nih.gov/entrez/query.fcgi?db=Nucleotide&term=NM_153613) | hs\|15q14 |
| 0.0057907 | 0.137 | 0.0238095 | 0.7 | 1.47 | 2.08 | [SERTAD4](http://www.ncbi.nlm.nih.gov/entrez/query.fcgi?cmd=search&db=gene&term=SERTAD4) | SERTA domain containing 4 | [NM_019605](http://www.ncbi.nlm.nih.gov/entrez/query.fcgi?db=Nucleotide&term=NM_019605) | hs\|1q32.2 |
| 0.0076434 | 0.139 | 0.0238095 | 0.81 | 1.69 | 2.08 | [SLC37A1](http://www.ncbi.nlm.nih.gov/entrez/query.fcgi?cmd=search&db=gene&term=SLC37A1) | solute carrier family 37 (glucose-6-phosphate transporter), member 1 | [NM_018964](http://www.ncbi.nlm.nih.gov/entrez/query.fcgi?db=Nucleotide&term=NM_018964) | hs\|21q22.3 |
| 0.0029528 | 0.137 | 0.015873 | 0.75 | 1.56 | 2.08 | [TGFBR1](http://www.ncbi.nlm.nih.gov/entrez/query.fcgi?cmd=search&db=gene&term=TGFBR1) | transforming growth factor, beta receptor 1 | [NM_004612](http://www.ncbi.nlm.nih.gov/entrez/query.fcgi?db=Nucleotide&term=NM_004612) | hs\|9q22.33 |
| 0.0087778 | 0.142 | 0.0238095 | 0.7 | 1.44 | 2.08 | [TNFSF13B](http://www.ncbi.nlm.nih.gov/entrez/query.fcgi?cmd=search&db=gene&term=TNFSF13B) | tumor necrosis factor (ligand) superfamily, member 13b | [NM_006573](http://www.ncbi.nlm.nih.gov/entrez/query.fcgi?db=Nucleotide&term=NM_006573) | hs\|13q33.3 |
| 0.0058055 | 0.137 | 0.0238095 | 0.77 | 1.55 | 2.04 | [ADAM10](http://www.ncbi.nlm.nih.gov/entrez/query.fcgi?cmd=search&db=gene&term=ADAM10) | ADAM metallopeptidase domain 10 | [NM_001110](http://www.ncbi.nlm.nih.gov/entrez/query.fcgi?db=Nucleotide&term=NM_001110) | hs\|15q21.3 |
| 0.004566 | 0.137 | 0.015873 | 0.79 | 1.63 | 2.04 | [BAK1](http://www.ncbi.nlm.nih.gov/entrez/query.fcgi?cmd=search&db=gene&term=BAK1) | BCL2-antagonist/killer 1 | [NM_001188](http://www.ncbi.nlm.nih.gov/entrez/query.fcgi?db=Nucleotide&term=NM_001188) | hs\|6p21.31 |
| 0.0084246 | 0.141 | 0.031746 | 0.76 | 1.54 | 2.04 | [C1GALT1](http://www.ncbi.nlm.nih.gov/entrez/query.fcgi?cmd=search&db=gene&term=C1GALT1) | core 1 synthase, glycoprotein-N-acetylgalactosamine 3-beta-galactosyltransferase 1 | [NM_020156](http://www.ncbi.nlm.nih.gov/entrez/query.fcgi?db=Nucleotide&term=NM_020156) | hs\|7p22.1 |
| 0.0022298 | 0.137 | 0.015873 | 0.61 | 1.26 | 2.04 | [GAB3](http://www.ncbi.nlm.nih.gov/entrez/query.fcgi?cmd=search&db=gene&term=GAB3) | GRB2-associated binding protein 3 | [NM_001081573](http://www.ncbi.nlm.nih.gov/entrez/query.fcgi?db=Nucleotide&term=NM_001081573) | hs\|Xq28 |
| 0.0038392 | 0.137 | 0.0079365 | 0.74 | 1.51 | 2.04 | [ID1](http://www.ncbi.nlm.nih.gov/entrez/query.fcgi?cmd=search&db=gene&term=ID1) | inhibitor of DNA binding 1, dominant negative helix-loop-helix protein | [NM_002165](http://www.ncbi.nlm.nih.gov/entrez/query.fcgi?db=Nucleotide&term=NM_002165) | hs\|20q11.21 |
| 0.0061345 | 0.137 | 0.0238095 | 0.77 | 1.56 | 2.04 | [JUP](http://www.ncbi.nlm.nih.gov/entrez/query.fcgi?cmd=search&db=gene&term=JUP) | junction plakoglobin | [NM_002230](http://www.ncbi.nlm.nih.gov/entrez/query.fcgi?db=Nucleotide&term=NM_002230) | hs\|17q21.2 |
| 0.0086832 | 0.142 | 0.031746 | 0.67 | 1.38 | 2.04 | [LOC101928837](http://www.ncbi.nlm.nih.gov/entrez/query.fcgi?cmd=search&db=gene&term=LOC101928837) | uncharacterized LOC101928837 | [NR_120561](http://www.ncbi.nlm.nih.gov/entrez/query.fcgi?db=Nucleotide&term=NR_120561) | hs\|11q13.5 |
| 0.0086181 | 0.142 | 0.0238095 | 0.75 | 1.54 | 2.04 | [NMI](http://www.ncbi.nlm.nih.gov/entrez/query.fcgi?cmd=search&db=gene&term=NMI) | N-myc (and STAT) interactor | [NM_004688](http://www.ncbi.nlm.nih.gov/entrez/query.fcgi?db=Nucleotide&term=NM_004688) | hs\|2q23.3 |
| 0.0029773 | 0.137 | 0.0079365 | 0.8 | 1.64 | 2.04 | [PCDH1](http://www.ncbi.nlm.nih.gov/entrez/query.fcgi?cmd=search&db=gene&term=PCDH1) | protocadherin 1 | [NM_002587](http://www.ncbi.nlm.nih.gov/entrez/query.fcgi?db=Nucleotide&term=NM_002587) | hs\|5q31.3 |
| 0.0048225 | 0.137 | 0.015873 | 0.58 | 1.19 | 2.04 | [PDGFRB](http://www.ncbi.nlm.nih.gov/entrez/query.fcgi?cmd=search&db=gene&term=PDGFRB) | platelet-derived growth factor receptor, beta polypeptide | [NM_002609](http://www.ncbi.nlm.nih.gov/entrez/query.fcgi?db=Nucleotide&term=NM_002609) | hs\|5q32 |
| 0.0093226 | 0.143 | 0.0238095 | 0.64 | 1.3 | 2.04 | [PLEKHG1](http://www.ncbi.nlm.nih.gov/entrez/query.fcgi?cmd=search&db=gene&term=PLEKHG1) | pleckstrin homology domain containing, family G (with RhoGef domain) member 1 | [NM_001029884](http://www.ncbi.nlm.nih.gov/entrez/query.fcgi?db=Nucleotide&term=NM_001029884) | hs\|6q25.1 |
| 0.0063895 | 0.137 | 0.015873 | 0.74 | 1.51 | 2.04 | [SOX4](http://www.ncbi.nlm.nih.gov/entrez/query.fcgi?cmd=search&db=gene&term=SOX4) | SRY (sex determining region Y)-box 4 | [NM_003107](http://www.ncbi.nlm.nih.gov/entrez/query.fcgi?db=Nucleotide&term=NM_003107) | hs\|6p22.3 |
| 0.007526 | 0.139 | 0.015873 | 0.7 | 1.43 | 2.04 | [SYTL2](http://www.ncbi.nlm.nih.gov/entrez/query.fcgi?cmd=search&db=gene&term=SYTL2) | synaptotagmin-like 2 | [NM_032943](http://www.ncbi.nlm.nih.gov/entrez/query.fcgi?db=Nucleotide&term=NM_032943) | hs\|11q14.1 |
| 0.0031159 | 0.137 | 0.015873 | 0.7 | 1.43 | 2.04 | [TMEM2](http://www.ncbi.nlm.nih.gov/entrez/query.fcgi?cmd=search&db=gene&term=TMEM2) | transmembrane protein 2 | [NM_013390](http://www.ncbi.nlm.nih.gov/entrez/query.fcgi?db=Nucleotide&term=NM_013390) | hs\|9q21.13 |
| 0.0065234 | 0.137 | 0.0079365 | 0.82 | 1.67 | 2.04 |  |  |  | hs\|12q21.2 |
| 0.0014615 | 0.137 | 0.0079365 | 0.85 | 1.7 | 2 | [ADAM9](http://www.ncbi.nlm.nih.gov/entrez/query.fcgi?cmd=search&db=gene&term=ADAM9) | ADAM metallopeptidase domain 9 | [NM_003816](http://www.ncbi.nlm.nih.gov/entrez/query.fcgi?db=Nucleotide&term=NM_003816) | hs\|8p11.22 |
| 0.001146 | 0.137 | 0.0079365 | 0.77 | 1.56 | 2 | [B3GNT5](http://www.ncbi.nlm.nih.gov/entrez/query.fcgi?cmd=search&db=gene&term=B3GNT5) | UDP-GlcNAc:betaGal beta-1,3-N-acetylglucosaminyltransferase 5 | [NM_032047](http://www.ncbi.nlm.nih.gov/entrez/query.fcgi?db=Nucleotide&term=NM_032047) | hs\|3q27.1 |
| 0.006725 | 0.137 | 0.015873 | 0.64 | 1.28 | 2 | [CD58](http://www.ncbi.nlm.nih.gov/entrez/query.fcgi?cmd=search&db=gene&term=CD58) | CD58 molecule | [NM_001779](http://www.ncbi.nlm.nih.gov/entrez/query.fcgi?db=Nucleotide&term=NM_001779) | hs\|1p13.1 |
| 0.0046566 | 0.137 | 0.0079365 | 0.79 | 1.59 | 2 | [LINC00857](http://www.ncbi.nlm.nih.gov/entrez/query.fcgi?cmd=search&db=gene&term=LINC00857) | long intergenic non-protein coding RNA 857 | [NR_038464](http://www.ncbi.nlm.nih.gov/entrez/query.fcgi?db=Nucleotide&term=NR_038464) | hs\|10q22.3 |
| 0.0066441 | 0.137 | 0.0079365 | 0.65 | 1.31 | 2 | [lnc-EPHA1-1](http://www.ncbi.nlm.nih.gov/entrez/query.fcgi?cmd=search&db=gene&term=lnc-EPHA1-1) | lnc-EPHA1-1:1 | [XM_006710194](http://www.ncbi.nlm.nih.gov/entrez/query.fcgi?db=Nucleotide&term=XM_006710194) | hs\|7q34 |
| 0.0080427 | 0.141 | 0.0238095 | 0.82 | 1.64 | 2 | [MICALL2](http://www.ncbi.nlm.nih.gov/entrez/query.fcgi?cmd=search&db=gene&term=MICALL2) | MICAL-like 2 | [NM_182924](http://www.ncbi.nlm.nih.gov/entrez/query.fcgi?db=Nucleotide&term=NM_182924) | hs\|7p22.3 |
| 0.0052914 | 0.137 | 0.015873 | 0.8 | 1.62 | 2 | [YWHAZ](http://www.ncbi.nlm.nih.gov/entrez/query.fcgi?cmd=search&db=gene&term=YWHAZ) | tyrosine 3-monooxygenase/tryptophan 5-monooxygenase activation protein, zeta | [NM_145690](http://www.ncbi.nlm.nih.gov/entrez/query.fcgi?db=Nucleotide&term=NM_145690) | hs\|8q22.3 |
| 0.0096777 | 0.144 | 0.015873 | 0.74 | 1.49 | 2 | [ZFP64](http://www.ncbi.nlm.nih.gov/entrez/query.fcgi?cmd=search&db=gene&term=ZFP64) | ZFP64 zinc finger protein | [NM_199427](http://www.ncbi.nlm.nih.gov/entrez/query.fcgi?db=Nucleotide&term=NM_199427) | hs\|20q13.2 |
| 0.0087247 | 0.142 | 0.0238095 | 1.61 | 0.8 | 0.5 | [lnc-AC016251.1-8](http://www.ncbi.nlm.nih.gov/entrez/query.fcgi?cmd=search&db=gene&term=lnc-AC016251.1-8) | lnc-AC016251.1-8:3 |  | hs\|15q26.2 |
| 0.0031976 | 0.137 | 0.0079365 | 1.63 | 0.82 | 0.5 | [PITX3](http://www.ncbi.nlm.nih.gov/entrez/query.fcgi?cmd=search&db=gene&term=PITX3) | paired-like homeodomain 3 | [NM_005029](http://www.ncbi.nlm.nih.gov/entrez/query.fcgi?db=Nucleotide&term=NM_005029) | hs\|10q24.32 |
| 0.0014638 | 0.137 | 0.0079365 | 1.53 | 0.77 | 0.5 |  |  |  | hs\|11q13.3 |
| 0.0048365 | 0.137 | 0.0238095 | 1.54 | 0.77 | 0.5 |  |  | [AY956761](http://www.ncbi.nlm.nih.gov/entrez/query.fcgi?db=Nucleotide&term=AY956761) | hs\|3q27.1 |
| 0.0036561 | 0.137 | 0.015873 | 1.62 | 0.8 | 0.5 | [lnc-CRIPAK-1](http://www.ncbi.nlm.nih.gov/entrez/query.fcgi?cmd=search&db=gene&term=lnc-CRIPAK-1) | lnc-CRIPAK-1:1 | [XM_006713937](http://www.ncbi.nlm.nih.gov/entrez/query.fcgi?db=Nucleotide&term=XM_006713937) | hs\|4p16.3 |
| 0.0057876 | 0.137 | 0.0238095 | 1.68 | 0.83 | 0.5 | [lnc-IFFO1-1](http://www.ncbi.nlm.nih.gov/entrez/query.fcgi?cmd=search&db=gene&term=lnc-IFFO1-1) | lnc-IFFO1-1:1 |  | hs\|12p13.31 |
| 0.0015254 | 0.137 | 0.0079365 | 1.48 | 0.74 | 0.5 | [lnc-NLGN2-1](http://www.ncbi.nlm.nih.gov/entrez/query.fcgi?cmd=search&db=gene&term=lnc-NLGN2-1) | lnc-NLGN2-1:2 |  | hs\|17p13.1 |
| 0.005569 | 0.137 | 0.015873 | 1.63 | 0.81 | 0.5 | [lnc-PIK3R1-3](http://www.ncbi.nlm.nih.gov/entrez/query.fcgi?cmd=search&db=gene&term=lnc-PIK3R1-3) | lnc-PIK3R1-3:1 |  | hs\|5q13.1 |
| 0.0068182 | 0.137 | 0.015873 | 1.34 | 0.66 | 0.5 | [CACHD1](http://www.ncbi.nlm.nih.gov/entrez/query.fcgi?cmd=search&db=gene&term=CACHD1) | cache domain containing 1 | [NM_020925](http://www.ncbi.nlm.nih.gov/entrez/query.fcgi?db=Nucleotide&term=NM_020925) | hs\|1p31.3 |
| 0.0054017 | 0.137 | 0.0079365 | 1.23 | 0.61 | 0.5 | [LINC01094](http://www.ncbi.nlm.nih.gov/entrez/query.fcgi?cmd=search&db=gene&term=LINC01094) | long intergenic non-protein coding RNA 1094 |  | hs\|4q21.21 |
| 0.0046262 | 0.137 | 0.0238095 | 1.74 | 0.86 | 0.5 | [lnc-TUSC5-2](http://www.ncbi.nlm.nih.gov/entrez/query.fcgi?cmd=search&db=gene&term=lnc-TUSC5-2) | lnc-TUSC5-2:1 |  | hs\|17p13.3 |
| 0.002357 | 0.137 | 0.0079365 | 1.74 | 0.86 | 0.5 | [QPRT](http://www.ncbi.nlm.nih.gov/entrez/query.fcgi?cmd=search&db=gene&term=QPRT) | quinolinate phosphoribosyltransferase | [NM_014298](http://www.ncbi.nlm.nih.gov/entrez/query.fcgi?db=Nucleotide&term=NM_014298) | hs\|16p11.2 |
| 0.0006295 | 0.137 | 0.0079365 | 1.42 | 0.7 | 0.5 |  |  | [XR_108869](http://www.ncbi.nlm.nih.gov/entrez/query.fcgi?db=Nucleotide&term=XR_108869) | hs\|8q22.1 |
| 0.0043589 | 0.137 | 0.015873 | 1.7 | 0.84 | 0.5 |  |  |  | hs\|15q24.1 |
| 0.0080734 | 0.141 | 0.0079365 | 1.68 | 0.83 | 0.5 |  |  | [XM_006718601](http://www.ncbi.nlm.nih.gov/entrez/query.fcgi?db=Nucleotide&term=XM_006718601) | hs\|11q13.3 |
| 0.0015965 | 0.137 | 0.0079365 | 1.34 | 0.66 | 0.49 | [GDF7](http://www.ncbi.nlm.nih.gov/entrez/query.fcgi?cmd=search&db=gene&term=GDF7) | growth differentiation factor 7 | [NM_182828](http://www.ncbi.nlm.nih.gov/entrez/query.fcgi?db=Nucleotide&term=NM_182828) | hs\|2p24.1 |
| 0.0012085 | 0.137 | 0.0079365 | 1.72 | 0.85 | 0.49 | [LINC00969](http://www.ncbi.nlm.nih.gov/entrez/query.fcgi?cmd=search&db=gene&term=LINC00969) | long intergenic non-protein coding RNA 969 | [NR_122105](http://www.ncbi.nlm.nih.gov/entrez/query.fcgi?db=Nucleotide&term=NR_122105) | hs\|3q29 |
| 0.0079998 | 0.141 | 0.015873 | 1.76 | 0.87 | 0.49 | [SBK2](http://www.ncbi.nlm.nih.gov/entrez/query.fcgi?cmd=search&db=gene&term=SBK2) | SH3 domain binding kinase family, member 2 | [NM_001101401](http://www.ncbi.nlm.nih.gov/entrez/query.fcgi?db=Nucleotide&term=NM_001101401) | hs\|19q13.42 |
| 0.0044014 | 0.137 | 0.015873 | 1.26 | 0.62 | 0.49 | [WEE2-AS1](http://www.ncbi.nlm.nih.gov/entrez/query.fcgi?cmd=search&db=gene&term=WEE2-AS1) | WEE2 antisense RNA 1 | [NR_015392](http://www.ncbi.nlm.nih.gov/entrez/query.fcgi?db=Nucleotide&term=NR_015392) | hs\|7q34 |
| 0.001799 | 0.137 | 0.0079365 | 1.56 | 0.77 | 0.49 | [XLOC_l2_010854](http://www.ncbi.nlm.nih.gov/entrez/query.fcgi?cmd=search&db=gene&term=XLOC_l2_010854) |  |  | hs\|4q28.3 |
| 0.0047044 | 0.137 | 0.0079365 | 1.77 | 0.87 | 0.49 | [ADAM32](http://www.ncbi.nlm.nih.gov/entrez/query.fcgi?cmd=search&db=gene&term=ADAM32) | ADAM metallopeptidase domain 32 | [NM_145004](http://www.ncbi.nlm.nih.gov/entrez/query.fcgi?db=Nucleotide&term=NM_145004) | hs\|8p11.22 |
| 0.0094353 | 0.143 | 0.015873 | 1.35 | 0.66 | 0.49 | [ATOH8](http://www.ncbi.nlm.nih.gov/entrez/query.fcgi?cmd=search&db=gene&term=ATOH8) | atonal homolog 8 (Drosophila) | [NM_032827](http://www.ncbi.nlm.nih.gov/entrez/query.fcgi?db=Nucleotide&term=NM_032827) | hs\|2p11.2 |
| 0.0015693 | 0.137 | 0.0079365 | 1.38 | 0.68 | 0.49 | [BMP1](http://www.ncbi.nlm.nih.gov/entrez/query.fcgi?cmd=search&db=gene&term=BMP1) | bone morphogenetic protein 1 | [AK291620](http://www.ncbi.nlm.nih.gov/entrez/query.fcgi?db=Nucleotide&term=AK291620) | hs\|8p21.3 |
| 0.007798 | 0.14 | 0.0238095 | 1.77 | 0.87 | 0.49 | [lnc-ESCO1-2](http://www.ncbi.nlm.nih.gov/entrez/query.fcgi?cmd=search&db=gene&term=lnc-ESCO1-2) | lnc-ESCO1-2:1 | [BX099285](http://www.ncbi.nlm.nih.gov/entrez/query.fcgi?db=Nucleotide&term=BX099285) | hs\|18q11.1 |
| 0.0037678 | 0.137 | 0.0079365 | 1.76 | 0.86 | 0.49 | [lnc-NEO1-1](http://www.ncbi.nlm.nih.gov/entrez/query.fcgi?cmd=search&db=gene&term=lnc-NEO1-1) | lnc-NEO1-1:2 |  | hs\|15q24.1 |
| 0.0031946 | 0.137 | 0.0079365 | 1.33 | 0.65 | 0.49 | [PIFO](http://www.ncbi.nlm.nih.gov/entrez/query.fcgi?cmd=search&db=gene&term=PIFO) | primary cilia formation | [NM_181643](http://www.ncbi.nlm.nih.gov/entrez/query.fcgi?db=Nucleotide&term=NM_181643) | hs\|1p13.2 |
| 0.0039912 | 0.137 | 0.015873 | 1.59 | 0.78 | 0.49 |  |  | [XM_006710560](http://www.ncbi.nlm.nih.gov/entrez/query.fcgi?db=Nucleotide&term=XM_006710560) | hs\|1p36.11 |
| 0.0054368 | 0.137 | 0.015873 | 1.73 | 0.85 | 0.49 |  |  |  | hs\|19p13.11 |
| 0.0092736 | 0.143 | 0.0396825 | 1.71 | 0.84 | 0.49 |  |  |  | hs\|14q32.32 |
| 0.0035681 | 0.137 | 0.015873 | 1.34 | 0.65 | 0.49 | [ALDH6A1](http://www.ncbi.nlm.nih.gov/entrez/query.fcgi?cmd=search&db=gene&term=ALDH6A1) | aldehyde dehydrogenase 6 family, member A1 | [NM_005589](http://www.ncbi.nlm.nih.gov/entrez/query.fcgi?db=Nucleotide&term=NM_005589) | hs\|14q24.3 |
| 0.0022792 | 0.137 | 0.015873 | 1.62 | 0.79 | 0.49 | [CFAP74](http://www.ncbi.nlm.nih.gov/entrez/query.fcgi?cmd=search&db=gene&term=CFAP74) | cilia and flagella associated protein 74 | [XM_006710997](http://www.ncbi.nlm.nih.gov/entrez/query.fcgi?db=Nucleotide&term=XM_006710997) | hs\|1p36.33 |
| 0.0044859 | 0.137 | 0.015873 | 1.79 | 0.88 | 0.49 | [LOC100134868](http://www.ncbi.nlm.nih.gov/entrez/query.fcgi?cmd=search&db=gene&term=LOC100134868) | uncharacterized LOC100134868 | [NR_004846](http://www.ncbi.nlm.nih.gov/entrez/query.fcgi?db=Nucleotide&term=NR_004846) | hs\|20p11.1 |
| 0.0004596 | 0.137 | 0.0079365 | 1.55 | 0.76 | 0.49 | [XLOC_l2_014182](http://www.ncbi.nlm.nih.gov/entrez/query.fcgi?cmd=search&db=gene&term=XLOC_l2_014182) |  |  | hs\|8p11.22 |
| 0.001225 | 0.137 | 0.0079365 | 1.84 | 0.9 | 0.49 |  |  | [DB027098](http://www.ncbi.nlm.nih.gov/entrez/query.fcgi?db=Nucleotide&term=DB027098) | hs\|17q21.31 |
| 0.0071606 | 0.138 | 0.015873 | 1.62 | 0.79 | 0.49 | [CALML5](http://www.ncbi.nlm.nih.gov/entrez/query.fcgi?cmd=search&db=gene&term=CALML5) | calmodulin-like 5 | [NM_017422](http://www.ncbi.nlm.nih.gov/entrez/query.fcgi?db=Nucleotide&term=NM_017422) | hs\|10p15.1 |
| 0.0087536 | 0.142 | 0.0238095 | 1.61 | 0.78 | 0.49 | [lnc-TUBGCP3-6](http://www.ncbi.nlm.nih.gov/entrez/query.fcgi?cmd=search&db=gene&term=lnc-TUBGCP3-6) | lnc-TUBGCP3-6:3 |  | hs\|13q34 |
| 0.0021741 | 0.137 | 0.0079365 | 1.67 | 0.81 | 0.49 | [MIOX](http://www.ncbi.nlm.nih.gov/entrez/query.fcgi?cmd=search&db=gene&term=MIOX) | myo-inositol oxygenase | [NM_017584](http://www.ncbi.nlm.nih.gov/entrez/query.fcgi?db=Nucleotide&term=NM_017584) | hs\|22q13.33 |
| 0.0098117 | 0.144 | 0.0238095 | 1.46 | 0.7 | 0.49 | [PHACTR4](http://www.ncbi.nlm.nih.gov/entrez/query.fcgi?cmd=search&db=gene&term=PHACTR4) | phosphatase and actin regulator 4 | [NM_001048183](http://www.ncbi.nlm.nih.gov/entrez/query.fcgi?db=Nucleotide&term=NM_001048183) | hs\|1p35.3 |
| 0.0066493 | 0.137 | 0.015873 | 1.49 | 0.72 | 0.49 | [TEX13B](http://www.ncbi.nlm.nih.gov/entrez/query.fcgi?cmd=search&db=gene&term=TEX13B) | testis expressed 13B | [NM_031273](http://www.ncbi.nlm.nih.gov/entrez/query.fcgi?db=Nucleotide&term=NM_031273) | hs\|Xq22.3 |
| 0.0033126 | 0.137 | 0.0079365 | 1.27 | 0.62 | 0.49 | [TNXB](http://www.ncbi.nlm.nih.gov/entrez/query.fcgi?cmd=search&db=gene&term=TNXB) | tenascin XB | [NM_019105](http://www.ncbi.nlm.nih.gov/entrez/query.fcgi?db=Nucleotide&term=NM_019105) | hs\|6p21.33 |
| 0.0007097 | 0.137 | 0.0079365 | 1.29 | 0.63 | 0.49 | [VGLL3](http://www.ncbi.nlm.nih.gov/entrez/query.fcgi?cmd=search&db=gene&term=VGLL3) | vestigial-like family member 3 | [NM_016206](http://www.ncbi.nlm.nih.gov/entrez/query.fcgi?db=Nucleotide&term=NM_016206) | hs\|3p12.1 |
| 0.0044313 | 0.137 | 0.0079365 | 1.76 | 0.86 | 0.49 |  |  |  | hs\|11p11.2 |
| 0.0038664 | 0.137 | 0.031746 | 1.75 | 0.84 | 0.48 | [EMX1](http://www.ncbi.nlm.nih.gov/entrez/query.fcgi?cmd=search&db=gene&term=EMX1) | empty spiracles homeobox 1 | [BC037242](http://www.ncbi.nlm.nih.gov/entrez/query.fcgi?db=Nucleotide&term=BC037242) | hs\|2p13.2 |
| 0.0052086 | 0.137 | 0.015873 | 1.54 | 0.74 | 0.48 | [lnc-FGF8-1](http://www.ncbi.nlm.nih.gov/entrez/query.fcgi?cmd=search&db=gene&term=lnc-FGF8-1) | lnc-FGF8-1:2 |  | hs\|10q24.32 |
| 0.0089456 | 0.142 | 0.031746 | 1.76 | 0.85 | 0.48 | [LOC401052](http://www.ncbi.nlm.nih.gov/entrez/query.fcgi?cmd=search&db=gene&term=LOC401052) | uncharacterized LOC401052 | [NM_001008737](http://www.ncbi.nlm.nih.gov/entrez/query.fcgi?db=Nucleotide&term=NM_001008737) | hs\|3p25.3 |
| 0.0097498 | 0.144 | 0.015873 | 1.78 | 0.86 | 0.48 |  |  | [DA338882](http://www.ncbi.nlm.nih.gov/entrez/query.fcgi?db=Nucleotide&term=DA338882) | hs\|14q32.12 |
| 0.0052495 | 0.137 | 0.031746 | 1.71 | 0.82 | 0.48 | [lnc-XIAP-1](http://www.ncbi.nlm.nih.gov/entrez/query.fcgi?cmd=search&db=gene&term=lnc-XIAP-1) | lnc-XIAP-1:1 |  | hs\|Xq25 |
| 0.0014653 | 0.137 | 0.0079365 | 1.53 | 0.74 | 0.48 | [ACAT1](http://www.ncbi.nlm.nih.gov/entrez/query.fcgi?cmd=search&db=gene&term=ACAT1) | acetyl-CoA acetyltransferase 1 | [NM_000019](http://www.ncbi.nlm.nih.gov/entrez/query.fcgi?db=Nucleotide&term=NM_000019) | hs\|11q22.3 |
| 0.0051303 | 0.137 | 0.0238095 | 1.87 | 0.9 | 0.48 | [DUX4](http://www.ncbi.nlm.nih.gov/entrez/query.fcgi?cmd=search&db=gene&term=DUX4) | double homeobox 4 | [NM_001293798](http://www.ncbi.nlm.nih.gov/entrez/query.fcgi?db=Nucleotide&term=NM_001293798) |  |
| 0.0090314 | 0.143 | 0.015873 | 1.4 | 0.67 | 0.48 | [EEF2](http://www.ncbi.nlm.nih.gov/entrez/query.fcgi?cmd=search&db=gene&term=EEF2) | eukaryotic translation elongation factor 2 | [NM_001961](http://www.ncbi.nlm.nih.gov/entrez/query.fcgi?db=Nucleotide&term=NM_001961) | hs\|19p13.3 |
| 0.0010256 | 0.137 | 0.0079365 | 1.67 | 0.79 | 0.48 | [lnc-OPN4-2](http://www.ncbi.nlm.nih.gov/entrez/query.fcgi?cmd=search&db=gene&term=lnc-OPN4-2) | lnc-OPN4-2:1 | [AK090628](http://www.ncbi.nlm.nih.gov/entrez/query.fcgi?db=Nucleotide&term=AK090628) | hs\|10q23.1 |
| 0.0011436 | 0.137 | 0.0079365 | 1.27 | 0.61 | 0.48 |  |  |  | hs\|6q24.2 |
| 0.0083115 | 0.141 | 0.015873 | 1.44 | 0.69 | 0.48 |  |  |  | hs\|4q35.2 |
| 0.0002327 | 0.137 | 0.0079365 | 1.4 | 0.67 | 0.48 | [EDA](http://www.ncbi.nlm.nih.gov/entrez/query.fcgi?cmd=search&db=gene&term=EDA) | ectodysplasin A | [NM_001005610](http://www.ncbi.nlm.nih.gov/entrez/query.fcgi?db=Nucleotide&term=NM_001005610) | hs\|Xq13.1 |
| 0.0040028 | 0.137 | 0.015873 | 1.62 | 0.77 | 0.48 | [FAM71E2](http://www.ncbi.nlm.nih.gov/entrez/query.fcgi?cmd=search&db=gene&term=FAM71E2) | family with sequence similarity 71, member E2 | [NM_001145402](http://www.ncbi.nlm.nih.gov/entrez/query.fcgi?db=Nucleotide&term=NM_001145402) | hs\|19q13.42 |
| 0.0007173 | 0.137 | 0.0079365 | 1.69 | 0.81 | 0.48 | [LINC00106](http://www.ncbi.nlm.nih.gov/entrez/query.fcgi?cmd=search&db=gene&term=LINC00106) | long intergenic non-protein coding RNA 106 | [BQ009527](http://www.ncbi.nlm.nih.gov/entrez/query.fcgi?db=Nucleotide&term=BQ009527) | hs\|Xp22.33 |
| 0.002363 | 0.137 | 0.015873 | 1.68 | 0.8 | 0.48 | [lnc-C6orf221-2](http://www.ncbi.nlm.nih.gov/entrez/query.fcgi?cmd=search&db=gene&term=lnc-C6orf221-2) | lnc-C6orf221-2:3 | [AL832252](http://www.ncbi.nlm.nih.gov/entrez/query.fcgi?db=Nucleotide&term=AL832252) | hs\|6q13 |
| 0.0082058 | 0.141 | 0.0238095 | 1.36 | 0.65 | 0.48 | [lnc-POFUT2-3](http://www.ncbi.nlm.nih.gov/entrez/query.fcgi?cmd=search&db=gene&term=lnc-POFUT2-3) | lnc-POFUT2-3:1 |  | hs\|21q22.3 |
| 0.0035075 | 0.137 | 0.015873 | 1.6 | 0.76 | 0.48 | [lnc-SH2D7-5](http://www.ncbi.nlm.nih.gov/entrez/query.fcgi?cmd=search&db=gene&term=lnc-SH2D7-5) | lnc-SH2D7-5:2 |  | hs\|15q24.3 |
| 0.0014299 | 0.137 | 0.0079365 | 1.56 | 0.74 | 0.48 |  |  | [TCONS_l2_00011128](http://www.ncbi.nlm.nih.gov/entrez/query.fcgi?db=Nucleotide&term=TCONS_l2_00011128) | hs\|17p11.2 |
| 0.0022088 | 0.137 | 0.015873 | 1.21 | 0.57 | 0.47 | [HRASLS](http://www.ncbi.nlm.nih.gov/entrez/query.fcgi?cmd=search&db=gene&term=HRASLS) | HRAS-like suppressor | [NM_020386](http://www.ncbi.nlm.nih.gov/entrez/query.fcgi?db=Nucleotide&term=NM_020386) | hs\|3q29 |
| 0.0009924 | 0.137 | 0.0079365 | 1.72 | 0.81 | 0.47 | [lnc-RP11-503N18.3.1-3](http://www.ncbi.nlm.nih.gov/entrez/query.fcgi?cmd=search&db=gene&term=lnc-RP11-503N18.3.1-3) | lnc-RP11-503N18.3.1-3:1 | [DB125444](http://www.ncbi.nlm.nih.gov/entrez/query.fcgi?db=Nucleotide&term=DB125444) | hs\|4p16.3 |
| 0.0018588 | 0.137 | 0.0079365 | 1.45 | 0.69 | 0.47 | [PRDM11](http://www.ncbi.nlm.nih.gov/entrez/query.fcgi?cmd=search&db=gene&term=PRDM11) | PR domain containing 11 | [NM_001256696](http://www.ncbi.nlm.nih.gov/entrez/query.fcgi?db=Nucleotide&term=NM_001256696) | hs\|11p11.2 |
| 0.0055771 | 0.137 | 0.0238095 | 1.47 | 0.7 | 0.47 | [CHID1](http://www.ncbi.nlm.nih.gov/entrez/query.fcgi?cmd=search&db=gene&term=CHID1) | chitinase domain containing 1 | [NM_023947](http://www.ncbi.nlm.nih.gov/entrez/query.fcgi?db=Nucleotide&term=NM_023947) | hs\|11p15.5 |
| 0.004954 | 0.137 | 0.0079365 | 1.65 | 0.77 | 0.47 | [FIGNL2](http://www.ncbi.nlm.nih.gov/entrez/query.fcgi?cmd=search&db=gene&term=FIGNL2) | fidgetin-like 2 | [NM_001013690](http://www.ncbi.nlm.nih.gov/entrez/query.fcgi?db=Nucleotide&term=NM_001013690) | hs\|12q13.13 |
| 0.0023075 | 0.137 | 0.0079365 | 1.32 | 0.62 | 0.47 | [LINC00173](http://www.ncbi.nlm.nih.gov/entrez/query.fcgi?cmd=search&db=gene&term=LINC00173) | long intergenic non-protein coding RNA 173 | [NR_027345](http://www.ncbi.nlm.nih.gov/entrez/query.fcgi?db=Nucleotide&term=NR_027345) | hs\|12q24.22 |
| 0.0038164 | 0.137 | 0.015873 | 1.78 | 0.84 | 0.47 | [lnc-FABP3-1](http://www.ncbi.nlm.nih.gov/entrez/query.fcgi?cmd=search&db=gene&term=lnc-FABP3-1) | lnc-FABP3-1:1 |  | hs\|1p35.2 |
| 0.0025652 | 0.137 | 0.015873 | 1.61 | 0.75 | 0.47 | [LOC403323](http://www.ncbi.nlm.nih.gov/entrez/query.fcgi?cmd=search&db=gene&term=LOC403323) | uncharacterized LOC403323 | [NR_122077](http://www.ncbi.nlm.nih.gov/entrez/query.fcgi?db=Nucleotide&term=NR_122077) | hs\|9q13 |
| 0.0021881 | 0.137 | 0.015873 | 1.7 | 0.8 | 0.47 | [PLAC4](http://www.ncbi.nlm.nih.gov/entrez/query.fcgi?cmd=search&db=gene&term=PLAC4) | placenta-specific 4 | [NM_182832](http://www.ncbi.nlm.nih.gov/entrez/query.fcgi?db=Nucleotide&term=NM_182832) | hs\|21q22.2 |
| 0.0022525 | 0.137 | 0.015873 | 1.8 | 0.84 | 0.47 | [PROSER3](http://www.ncbi.nlm.nih.gov/entrez/query.fcgi?cmd=search&db=gene&term=PROSER3) | proline and serine rich 3 | [NM_001039887](http://www.ncbi.nlm.nih.gov/entrez/query.fcgi?db=Nucleotide&term=NM_001039887) | hs\|19q13.12 |
| 0.007931 | 0.14 | 0.015873 | 1.86 | 0.87 | 0.47 | [RBMS2](http://www.ncbi.nlm.nih.gov/entrez/query.fcgi?cmd=search&db=gene&term=RBMS2) | RNA binding motif, single stranded interacting protein 2 | [NM_002898](http://www.ncbi.nlm.nih.gov/entrez/query.fcgi?db=Nucleotide&term=NM_002898) | hs\|12q13.3 |
| 0.0048683 | 0.137 | 0.031746 | 1.75 | 0.82 | 0.47 |  |  | [XR_109251](http://www.ncbi.nlm.nih.gov/entrez/query.fcgi?db=Nucleotide&term=XR_109251) | hs\|16p13.3 |
| 0.0087958 | 0.142 | 0.031746 | 1.85 | 0.87 | 0.47 |  |  | [BG190865](http://www.ncbi.nlm.nih.gov/entrez/query.fcgi?db=Nucleotide&term=BG190865) | hs\|8q22.2 |
| 0.003877 | 0.137 | 0.0079365 | 1.38 | 0.64 | 0.47 | [ACAT1](http://www.ncbi.nlm.nih.gov/entrez/query.fcgi?cmd=search&db=gene&term=ACAT1) | acetyl-CoA acetyltransferase 1 | [NM_000019](http://www.ncbi.nlm.nih.gov/entrez/query.fcgi?db=Nucleotide&term=NM_000019) | hs\|11q22.3 |
| 0.0046816 | 0.137 | 0.015873 | 1.61 | 0.75 | 0.47 | [lnc-C17orf97-6](http://www.ncbi.nlm.nih.gov/entrez/query.fcgi?cmd=search&db=gene&term=lnc-C17orf97-6) | lnc-C17orf97-6:1 | [DB058128](http://www.ncbi.nlm.nih.gov/entrez/query.fcgi?db=Nucleotide&term=DB058128) | hs\|17p13.3 |
| 0.0037418 | 0.137 | 0.0238095 | 1.85 | 0.87 | 0.47 | [MOG](http://www.ncbi.nlm.nih.gov/entrez/query.fcgi?cmd=search&db=gene&term=MOG) | myelin oligodendrocyte glycoprotein | [NM_001008229](http://www.ncbi.nlm.nih.gov/entrez/query.fcgi?db=Nucleotide&term=NM_001008229) | hs\|6p22.1 |
| 0.0045877 | 0.137 | 0.031746 | 1.77 | 0.83 | 0.47 | [NPPA](http://www.ncbi.nlm.nih.gov/entrez/query.fcgi?cmd=search&db=gene&term=NPPA) | natriuretic peptide A |  | hs\|1p36.22 |
| 0.004384 | 0.137 | 0.0238095 | 1.84 | 0.86 | 0.47 | [RUVBL1-AS1](http://www.ncbi.nlm.nih.gov/entrez/query.fcgi?cmd=search&db=gene&term=RUVBL1-AS1) | RUVBL1 antisense RNA 1 | [NR_046645](http://www.ncbi.nlm.nih.gov/entrez/query.fcgi?db=Nucleotide&term=NR_046645) | hs\|3q21.3 |
| 0.0025908 | 0.137 | 0.015873 | 1.48 | 0.69 | 0.47 | [TMEM184A](http://www.ncbi.nlm.nih.gov/entrez/query.fcgi?cmd=search&db=gene&term=TMEM184A) | transmembrane protein 184A | [NM_001097620](http://www.ncbi.nlm.nih.gov/entrez/query.fcgi?db=Nucleotide&term=NM_001097620) | hs\|7p22.3 |
| 8.88E-05 | 0.137 | 0.0079365 | 1.6 | 0.75 | 0.47 | [XKR5](http://www.ncbi.nlm.nih.gov/entrez/query.fcgi?cmd=search&db=gene&term=XKR5) | XK, Kell blood group complex subunit-related family, member 5 | [NM_207411](http://www.ncbi.nlm.nih.gov/entrez/query.fcgi?db=Nucleotide&term=NM_207411) | hs\|8p23.1 |
| 0.006975 | 0.138 | 0.031746 | 1.75 | 0.82 | 0.47 |  |  |  | hs\|3p13 |
| 0.0075881 | 0.139 | 0.015873 | 1.48 | 0.69 | 0.47 | [ADRA2B](http://www.ncbi.nlm.nih.gov/entrez/query.fcgi?cmd=search&db=gene&term=ADRA2B) | adrenoceptor alpha 2B | [NM_000682](http://www.ncbi.nlm.nih.gov/entrez/query.fcgi?db=Nucleotide&term=NM_000682) | hs\|2q11.1 |
| 0.0093606 | 0.143 | 0.031746 | 1.64 | 0.76 | 0.47 | [ENKD1](http://www.ncbi.nlm.nih.gov/entrez/query.fcgi?cmd=search&db=gene&term=ENKD1) | enkurin domain containing 1 | [NM_032140](http://www.ncbi.nlm.nih.gov/entrez/query.fcgi?db=Nucleotide&term=NM_032140) | hs\|16q22.1 |
| 0.0091998 | 0.143 | 0.031746 | 1.79 | 0.83 | 0.47 | [MOS](http://www.ncbi.nlm.nih.gov/entrez/query.fcgi?cmd=search&db=gene&term=MOS) | v-mos Moloney murine sarcoma viral oncogene homolog | [NM_005372](http://www.ncbi.nlm.nih.gov/entrez/query.fcgi?db=Nucleotide&term=NM_005372) | hs\|8q12.1 |
| 0.00883 | 0.142 | 0.031746 | 1.7 | 0.79 | 0.47 |  |  | [BM473780](http://www.ncbi.nlm.nih.gov/entrez/query.fcgi?db=Nucleotide&term=BM473780) | hs\|9q21.11 |
| 0.0010174 | 0.137 | 0.0079365 | 1.43 | 0.66 | 0.46 | [CHN2](http://www.ncbi.nlm.nih.gov/entrez/query.fcgi?cmd=search&db=gene&term=CHN2) | chimerin 2 | [NM_001293070](http://www.ncbi.nlm.nih.gov/entrez/query.fcgi?db=Nucleotide&term=NM_001293070) | hs\|7p14.3 |
| 0.0033595 | 0.137 | 0.015873 | 1.72 | 0.8 | 0.46 | [KRTAP5-11](http://www.ncbi.nlm.nih.gov/entrez/query.fcgi?cmd=search&db=gene&term=KRTAP5-11) | keratin associated protein 5-11 |  | hs\|11q13.4 |
| 0.0004362 | 0.137 | 0.0079365 | 1.55 | 0.72 | 0.46 | [lnc-DHX15-1](http://www.ncbi.nlm.nih.gov/entrez/query.fcgi?cmd=search&db=gene&term=lnc-DHX15-1) | lnc-DHX15-1:1 |  | hs\|4p15.2 |
| 0.0016609 | 0.137 | 0.0079365 | 1.82 | 0.84 | 0.46 | [PCOLCE-AS1](http://www.ncbi.nlm.nih.gov/entrez/query.fcgi?cmd=search&db=gene&term=PCOLCE-AS1) | PCOLCE antisense RNA 1 | [NR_038910](http://www.ncbi.nlm.nih.gov/entrez/query.fcgi?db=Nucleotide&term=NR_038910) | hs\|7q22.1 |
| 0.0035808 | 0.137 | 0.015873 | 1.7 | 0.79 | 0.46 | [TFE3](http://www.ncbi.nlm.nih.gov/entrez/query.fcgi?cmd=search&db=gene&term=TFE3) | transcription factor binding to IGHM enhancer 3 | [NM_006521](http://www.ncbi.nlm.nih.gov/entrez/query.fcgi?db=Nucleotide&term=NM_006521) | hs\|Xp11.23 |
| 0.0054306 | 0.137 | 0.015873 | 1.13 | 0.52 | 0.46 | [MAST4](http://www.ncbi.nlm.nih.gov/entrez/query.fcgi?cmd=search&db=gene&term=MAST4) | microtubule associated serine/threonine kinase family member 4 | [NM_001290226](http://www.ncbi.nlm.nih.gov/entrez/query.fcgi?db=Nucleotide&term=NM_001290226) | hs\|5q12.3 |
| 0.0025579 | 0.137 | 0.0079365 | 1.35 | 0.63 | 0.46 | [PROB1](http://www.ncbi.nlm.nih.gov/entrez/query.fcgi?cmd=search&db=gene&term=PROB1) | proline-rich basic protein 1 | [NM_001161546](http://www.ncbi.nlm.nih.gov/entrez/query.fcgi?db=Nucleotide&term=NM_001161546) | hs\|5q31.2 |
| 0.0038022 | 0.137 | 0.015873 | 1.65 | 0.76 | 0.46 | [CNTNAP1](http://www.ncbi.nlm.nih.gov/entrez/query.fcgi?cmd=search&db=gene&term=CNTNAP1) | contactin associated protein 1 | [NM_003632](http://www.ncbi.nlm.nih.gov/entrez/query.fcgi?db=Nucleotide&term=NM_003632) | hs\|17q21.2 |
| 0.0038314 | 0.137 | 0.0238095 | 1.36 | 0.62 | 0.46 | [ADIRF-AS1](http://www.ncbi.nlm.nih.gov/entrez/query.fcgi?cmd=search&db=gene&term=ADIRF-AS1) | ADIRF antisense RNA 1 | [BC017676](http://www.ncbi.nlm.nih.gov/entrez/query.fcgi?db=Nucleotide&term=BC017676) | hs\|10q23.2 |
| 0.0083488 | 0.141 | 0.015873 | 1.78 | 0.81 | 0.46 | [lnc-C17orf75-2](http://www.ncbi.nlm.nih.gov/entrez/query.fcgi?cmd=search&db=gene&term=lnc-C17orf75-2) | lnc-C17orf75-2:1 |  | hs\|17q11.2 |
| 0.0098426 | 0.144 | 0.0238095 | 1.33 | 0.61 | 0.46 | [MAST1](http://www.ncbi.nlm.nih.gov/entrez/query.fcgi?cmd=search&db=gene&term=MAST1) | microtubule associated serine/threonine kinase 1 | [NM_014975](http://www.ncbi.nlm.nih.gov/entrez/query.fcgi?db=Nucleotide&term=NM_014975) | hs\|19p13.2 |
| 0.005316 | 0.137 | 0.031746 | 1.2 | 0.55 | 0.46 | [MT1B](http://www.ncbi.nlm.nih.gov/entrez/query.fcgi?cmd=search&db=gene&term=MT1B) | metallothionein 1B | [NM_005947](http://www.ncbi.nlm.nih.gov/entrez/query.fcgi?db=Nucleotide&term=NM_005947) | hs\|16q12.2 |
| 0.009689 | 0.144 | 0.031746 | 1.2 | 0.55 | 0.46 | [MT1IP](http://www.ncbi.nlm.nih.gov/entrez/query.fcgi?cmd=search&db=gene&term=MT1IP) | metallothionein 1I, pseudogene | [NR_003669](http://www.ncbi.nlm.nih.gov/entrez/query.fcgi?db=Nucleotide&term=NR_003669) | hs\|16q13 |
| 0.0061916 | 0.137 | 0.031746 | 1.84 | 0.84 | 0.46 | [SYTL1](http://www.ncbi.nlm.nih.gov/entrez/query.fcgi?cmd=search&db=gene&term=SYTL1) | synaptotagmin-like 1 | [NM_032872](http://www.ncbi.nlm.nih.gov/entrez/query.fcgi?db=Nucleotide&term=NM_032872) | hs\|1p36.11 |
| 0.007638 | 0.139 | 0.0238095 | 1.79 | 0.82 | 0.46 |  |  | [BC010535](http://www.ncbi.nlm.nih.gov/entrez/query.fcgi?db=Nucleotide&term=BC010535) | hs\|7q11.23 |
| 0.0030872 | 0.137 | 0.0079365 | 1.88 | 0.85 | 0.45 | [LOC101930506](http://www.ncbi.nlm.nih.gov/entrez/query.fcgi?cmd=search&db=gene&term=LOC101930506) | uncharacterized LOC101930506 | [XR_249340](http://www.ncbi.nlm.nih.gov/entrez/query.fcgi?db=Nucleotide&term=XR_249340) | hs\|2q37.3 |
| 0.0063203 | 0.137 | 0.015873 | 1.83 | 0.83 | 0.45 | [ZBTB20-AS4](http://www.ncbi.nlm.nih.gov/entrez/query.fcgi?cmd=search&db=gene&term=ZBTB20-AS4) | ZBTB20 antisense RNA 4 | [NR_046877](http://www.ncbi.nlm.nih.gov/entrez/query.fcgi?db=Nucleotide&term=NR_046877) | hs\|3q13.31 |
| 0.0041436 | 0.137 | 0.031746 | 1.87 | 0.85 | 0.45 | [LOC101927856](http://www.ncbi.nlm.nih.gov/entrez/query.fcgi?cmd=search&db=gene&term=LOC101927856) | uncharacterized LOC101927856 | [XR_245773](http://www.ncbi.nlm.nih.gov/entrez/query.fcgi?db=Nucleotide&term=XR_245773) | hs\|14q23.3 |
| 0.0027198 | 0.137 | 0.0238095 | 1.92 | 0.87 | 0.45 | [DUX4](http://www.ncbi.nlm.nih.gov/entrez/query.fcgi?cmd=search&db=gene&term=DUX4) | double homeobox 4 | [NM_001293798](http://www.ncbi.nlm.nih.gov/entrez/query.fcgi?db=Nucleotide&term=NM_001293798) |  |
| 0.0037842 | 0.137 | 0.015873 | 1.77 | 0.8 | 0.45 | [lnc-NES-1](http://www.ncbi.nlm.nih.gov/entrez/query.fcgi?cmd=search&db=gene&term=lnc-NES-1) | lnc-NES-1:1 |  | hs\|1q23.1 |
| 0.0074296 | 0.139 | 0.0079365 | 1.62 | 0.73 | 0.45 | [LOC100132356](http://www.ncbi.nlm.nih.gov/entrez/query.fcgi?cmd=search&db=gene&term=LOC100132356) | uncharacterized LOC100132356 | [NR_034127](http://www.ncbi.nlm.nih.gov/entrez/query.fcgi?db=Nucleotide&term=NR_034127) | hs\|5p12 |
| 0.0035456 | 0.137 | 0.0079365 | 1.57 | 0.71 | 0.45 | [RCL1](http://www.ncbi.nlm.nih.gov/entrez/query.fcgi?cmd=search&db=gene&term=RCL1) | RNA terminal phosphate cyclase-like 1 |  | hs\|9p24.1 |
| 0.0030857 | 0.137 | 0.031746 | 1.83 | 0.82 | 0.45 | [BPIFB2](http://www.ncbi.nlm.nih.gov/entrez/query.fcgi?cmd=search&db=gene&term=BPIFB2) | BPI fold containing family B, member 2 | [NM_025227](http://www.ncbi.nlm.nih.gov/entrez/query.fcgi?db=Nucleotide&term=NM_025227) | hs\|20q11.21 |
| 0.0085048 | 0.141 | 0.015873 | 1.37 | 0.62 | 0.45 | [GADD45G](http://www.ncbi.nlm.nih.gov/entrez/query.fcgi?cmd=search&db=gene&term=GADD45G) | growth arrest and DNA-damage-inducible, gamma | [NM_006705](http://www.ncbi.nlm.nih.gov/entrez/query.fcgi?db=Nucleotide&term=NM_006705) | hs\|9q22.2 |
| 0.0092318 | 0.143 | 0.031746 | 1.95 | 0.87 | 0.45 | [lnc-APOA4-1](http://www.ncbi.nlm.nih.gov/entrez/query.fcgi?cmd=search&db=gene&term=lnc-APOA4-1) | lnc-APOA4-1:1 |  | hs\|11q23.3 |
| 0.0057527 | 0.137 | 0.031746 | 1.91 | 0.86 | 0.45 | [MAPK8IP1](http://www.ncbi.nlm.nih.gov/entrez/query.fcgi?cmd=search&db=gene&term=MAPK8IP1) | mitogen-activated protein kinase 8 interacting protein 1 | [NM_005456](http://www.ncbi.nlm.nih.gov/entrez/query.fcgi?db=Nucleotide&term=NM_005456) | hs\|11p11.2 |
| 0.004494 | 0.137 | 0.0079365 | 1.3 | 0.58 | 0.45 | [PRO1804](http://www.ncbi.nlm.nih.gov/entrez/query.fcgi?cmd=search&db=gene&term=PRO1804) | uncharacterized LOC100133319 | [AF132201](http://www.ncbi.nlm.nih.gov/entrez/query.fcgi?db=Nucleotide&term=AF132201) | hs\|17q24.3 |
| 0.0056259 | 0.137 | 0.031746 | 1.83 | 0.82 | 0.45 | [SEC11C](http://www.ncbi.nlm.nih.gov/entrez/query.fcgi?cmd=search&db=gene&term=SEC11C) | SEC11 homolog C (S. cerevisiae) | [NM_033280](http://www.ncbi.nlm.nih.gov/entrez/query.fcgi?db=Nucleotide&term=NM_033280) | hs\|18q21.32 |
| 0.0067167 | 0.137 | 0.031746 | 1.86 | 0.83 | 0.45 | [SOWAHA](http://www.ncbi.nlm.nih.gov/entrez/query.fcgi?cmd=search&db=gene&term=SOWAHA) | sosondowah ankyrin repeat domain family member A | [NM_175873](http://www.ncbi.nlm.nih.gov/entrez/query.fcgi?db=Nucleotide&term=NM_175873) | hs\|5q31.1 |
| 0.0049597 | 0.137 | 0.0238095 | 1.58 | 0.71 | 0.45 | [SYT15](http://www.ncbi.nlm.nih.gov/entrez/query.fcgi?cmd=search&db=gene&term=SYT15) | synaptotagmin XV | [NM_181519](http://www.ncbi.nlm.nih.gov/entrez/query.fcgi?db=Nucleotide&term=NM_181519) | hs\|10q11.22 |
| 0.0025226 | 0.137 | 0.0079365 | 1.36 | 0.61 | 0.45 |  |  |  | hs\|17q24.2 |
| 0.0027272 | 0.137 | 0.015873 | 1.79 | 0.8 | 0.45 |  |  | [XR_241053](http://www.ncbi.nlm.nih.gov/entrez/query.fcgi?db=Nucleotide&term=XR_241053) | hs\|1p36.33 |
| 0.0052494 | 0.137 | 0.015873 | 1.58 | 0.71 | 0.45 |  |  |  | hs\|19q13.41 |
| 0.0048146 | 0.137 | 0.015873 | 1.53 | 0.69 | 0.45 | [ACAT1](http://www.ncbi.nlm.nih.gov/entrez/query.fcgi?cmd=search&db=gene&term=ACAT1) | acetyl-CoA acetyltransferase 1 | [NM_000019](http://www.ncbi.nlm.nih.gov/entrez/query.fcgi?db=Nucleotide&term=NM_000019) | hs\|11q22.3 |
| 0.0044004 | 0.137 | 0.015873 | 1.7 | 0.76 | 0.45 | [UMOD](http://www.ncbi.nlm.nih.gov/entrez/query.fcgi?cmd=search&db=gene&term=UMOD) | uromodulin | [NM_003361](http://www.ncbi.nlm.nih.gov/entrez/query.fcgi?db=Nucleotide&term=NM_003361) | hs\|16p12.3 |
| 0.0088787 | 0.142 | 0.015873 | 1.51 | 0.67 | 0.44 | [ADHFE1](http://www.ncbi.nlm.nih.gov/entrez/query.fcgi?cmd=search&db=gene&term=ADHFE1) | alcohol dehydrogenase, iron containing, 1 | [NM_144650](http://www.ncbi.nlm.nih.gov/entrez/query.fcgi?db=Nucleotide&term=NM_144650) | hs\|8q13.1 |
| 0.0030204 | 0.137 | 0.0079365 | 1.88 | 0.84 | 0.44 | [DPYSL4](http://www.ncbi.nlm.nih.gov/entrez/query.fcgi?cmd=search&db=gene&term=DPYSL4) | dihydropyrimidinase-like 4 | [NM_006426](http://www.ncbi.nlm.nih.gov/entrez/query.fcgi?db=Nucleotide&term=NM_006426) | hs\|10q26.3 |
| 0.0030647 | 0.137 | 0.0079365 | 1.76 | 0.78 | 0.44 | [NRXN3](http://www.ncbi.nlm.nih.gov/entrez/query.fcgi?cmd=search&db=gene&term=NRXN3) | neurexin 3 | [NM_004796](http://www.ncbi.nlm.nih.gov/entrez/query.fcgi?db=Nucleotide&term=NM_004796) | hs\|14q31.1 |
| 0.0022032 | 0.137 | 0.0079365 | 1.45 | 0.64 | 0.44 | [PLTP](http://www.ncbi.nlm.nih.gov/entrez/query.fcgi?cmd=search&db=gene&term=PLTP) | phospholipid transfer protein | [NM_006227](http://www.ncbi.nlm.nih.gov/entrez/query.fcgi?db=Nucleotide&term=NM_006227) | hs\|20q13.12 |
| 0.0025165 | 0.137 | 0.0079365 | 1.94 | 0.86 | 0.44 |  |  | [BQ278507](http://www.ncbi.nlm.nih.gov/entrez/query.fcgi?db=Nucleotide&term=BQ278507) |  |
| 0.0045335 | 0.137 | 0.0238095 | 1.61 | 0.72 | 0.44 |  |  |  | hs\|10p15.3 |
| 0.0076929 | 0.14 | 0.015873 | 1.72 | 0.76 | 0.44 | [LOC101928659](http://www.ncbi.nlm.nih.gov/entrez/query.fcgi?cmd=search&db=gene&term=LOC101928659) | uncharacterized LOC101928659 | [XR_243494](http://www.ncbi.nlm.nih.gov/entrez/query.fcgi?db=Nucleotide&term=XR_243494) | hs\|16q24.2 |
| 0.0075766 | 0.139 | 0.0396825 | 1.27 | 0.56 | 0.44 | [MT1M](http://www.ncbi.nlm.nih.gov/entrez/query.fcgi?cmd=search&db=gene&term=MT1M) | metallothionein 1M | [NM_176870](http://www.ncbi.nlm.nih.gov/entrez/query.fcgi?db=Nucleotide&term=NM_176870) | hs\|16q12.2 |
| 0.0031286 | 0.137 | 0.0238095 | 1.47 | 0.65 | 0.44 | [NBPF6](http://www.ncbi.nlm.nih.gov/entrez/query.fcgi?cmd=search&db=gene&term=NBPF6) | neuroblastoma breakpoint family, member 6 | [NM_001143988](http://www.ncbi.nlm.nih.gov/entrez/query.fcgi?db=Nucleotide&term=NM_001143988) | hs\|1p13.3 |
| 0.0008358 | 0.137 | 0.0079365 | 1.76 | 0.78 | 0.44 | [TPP1](http://www.ncbi.nlm.nih.gov/entrez/query.fcgi?cmd=search&db=gene&term=TPP1) | tripeptidyl peptidase I | [NM_000391](http://www.ncbi.nlm.nih.gov/entrez/query.fcgi?db=Nucleotide&term=NM_000391) | hs\|11p15.4 |
| 0.0040195 | 0.137 | 0.031746 | 1.94 | 0.86 | 0.44 | [XKR4](http://www.ncbi.nlm.nih.gov/entrez/query.fcgi?cmd=search&db=gene&term=XKR4) | XK, Kell blood group complex subunit-related family, member 4 | [BM685801](http://www.ncbi.nlm.nih.gov/entrez/query.fcgi?db=Nucleotide&term=BM685801) | hs\|8q12.1 |
| 0.0023805 | 0.137 | 0.015873 | 1.81 | 0.8 | 0.44 |  |  |  | hs\|11p15.2 |
| 0.0057018 | 0.137 | 0.0238095 | 1.83 | 0.81 | 0.44 |  |  |  | hs\|17q25.3 |
| 0.0030896 | 0.137 | 0.015873 | 1.75 | 0.77 | 0.44 | [XLOC_l2_001529](http://www.ncbi.nlm.nih.gov/entrez/query.fcgi?cmd=search&db=gene&term=XLOC_l2_001529) |  |  | hs\|10p11.23 |
| 0.0041467 | 0.137 | 0.0079365 | 1.2 | 0.52 | 0.44 | [ACER1](http://www.ncbi.nlm.nih.gov/entrez/query.fcgi?cmd=search&db=gene&term=ACER1) | alkaline ceramidase 1 | [NM_133492](http://www.ncbi.nlm.nih.gov/entrez/query.fcgi?db=Nucleotide&term=NM_133492) | hs\|19p13.3 |
| 0.0026422 | 0.137 | 0.015873 | 1.84 | 0.81 | 0.44 | [DUX4](http://www.ncbi.nlm.nih.gov/entrez/query.fcgi?cmd=search&db=gene&term=DUX4) | double homeobox 4 | [NM_001293798](http://www.ncbi.nlm.nih.gov/entrez/query.fcgi?db=Nucleotide&term=NM_001293798) |  |
| 0.0089009 | 0.142 | 0.015873 | 1.59 | 0.7 | 0.44 | [FAT3](http://www.ncbi.nlm.nih.gov/entrez/query.fcgi?cmd=search&db=gene&term=FAT3) | FAT atypical cadherin 3 | [NM_001008781](http://www.ncbi.nlm.nih.gov/entrez/query.fcgi?db=Nucleotide&term=NM_001008781) | hs\|11q14.3 |
| 0.0093464 | 0.143 | 0.031746 | 1.59 | 0.7 | 0.44 | [IPO9-AS1](http://www.ncbi.nlm.nih.gov/entrez/query.fcgi?cmd=search&db=gene&term=IPO9-AS1) | IPO9 antisense RNA 1 | [NR_046696](http://www.ncbi.nlm.nih.gov/entrez/query.fcgi?db=Nucleotide&term=NR_046696) | hs\|1q32.1 |
| 0.0087777 | 0.142 | 0.0238095 | 1.4 | 0.62 | 0.44 | [lnc-BAI1-1](http://www.ncbi.nlm.nih.gov/entrez/query.fcgi?cmd=search&db=gene&term=lnc-BAI1-1) | lnc-BAI1-1:1 | [BC039302](http://www.ncbi.nlm.nih.gov/entrez/query.fcgi?db=Nucleotide&term=BC039302) | hs\|8q24.3 |
| 0.0008189 | 0.137 | 0.0079365 | 1.75 | 0.77 | 0.44 | [lnc-CDH4-1](http://www.ncbi.nlm.nih.gov/entrez/query.fcgi?cmd=search&db=gene&term=lnc-CDH4-1) | lnc-CDH4-1:1 |  | hs\|20q13.33 |
| 0.0062559 | 0.137 | 0.0238095 | 1.72 | 0.75 | 0.44 | [lnc-KCNT1-1](http://www.ncbi.nlm.nih.gov/entrez/query.fcgi?cmd=search&db=gene&term=lnc-KCNT1-1) | lnc-KCNT1-1:1 |  | hs\|9q34.3 |
| 0.0003624 | 0.137 | 0.0079365 | 1.47 | 0.64 | 0.44 | [RBPMS2](http://www.ncbi.nlm.nih.gov/entrez/query.fcgi?cmd=search&db=gene&term=RBPMS2) | RNA binding protein with multiple splicing 2 | [NM_194272](http://www.ncbi.nlm.nih.gov/entrez/query.fcgi?db=Nucleotide&term=NM_194272) | hs\|15q22.31 |
| 0.0095994 | 0.144 | 0.0079365 | 1.51 | 0.66 | 0.44 | [SEMA3G](http://www.ncbi.nlm.nih.gov/entrez/query.fcgi?cmd=search&db=gene&term=SEMA3G) | sema domain, immunoglobulin domain (Ig), short basic domain, secreted, (semaphorin) 3G | [NM_020163](http://www.ncbi.nlm.nih.gov/entrez/query.fcgi?db=Nucleotide&term=NM_020163) | hs\|3p21.1 |
| 0.0075005 | 0.139 | 0.0238095 | 1.28 | 0.56 | 0.43 | [lnc-ALB-1](http://www.ncbi.nlm.nih.gov/entrez/query.fcgi?cmd=search&db=gene&term=lnc-ALB-1) | lnc-ALB-1:1 |  | hs\|4q13.3 |
| 0.0005314 | 0.137 | 0.015873 | 1.62 | 0.7 | 0.43 | [lnc-PIPOX-1](http://www.ncbi.nlm.nih.gov/entrez/query.fcgi?cmd=search&db=gene&term=lnc-PIPOX-1) | lnc-PIPOX-1:1 |  | hs\|17q11.2 |
| 0.0025116 | 0.137 | 0.0079365 | 1.7 | 0.74 | 0.43 | [LOC102723862](http://www.ncbi.nlm.nih.gov/entrez/query.fcgi?cmd=search&db=gene&term=LOC102723862) | uncharacterized LOC102723862 | [XR_428989](http://www.ncbi.nlm.nih.gov/entrez/query.fcgi?db=Nucleotide&term=XR_428989) | hs\|11q22.3 |
| 0.0040612 | 0.137 | 0.0079365 | 1.25 | 0.54 | 0.43 | [TNXB](http://www.ncbi.nlm.nih.gov/entrez/query.fcgi?cmd=search&db=gene&term=TNXB) | tenascin XB | [NM_019105](http://www.ncbi.nlm.nih.gov/entrez/query.fcgi?db=Nucleotide&term=NM_019105) | hs\|6p21.33 |
| 0.0055026 | 0.137 | 0.0396825 | 2 | 0.87 | 0.43 |  |  | [BM973477](http://www.ncbi.nlm.nih.gov/entrez/query.fcgi?db=Nucleotide&term=BM973477) | hs\|17p13.2 |
| 0.0011617 | 0.137 | 0.015873 | 1.72 | 0.74 | 0.43 | [LOC100133286](http://www.ncbi.nlm.nih.gov/entrez/query.fcgi?cmd=search&db=gene&term=LOC100133286) | uncharacterized LOC100133286 | [NR_040084](http://www.ncbi.nlm.nih.gov/entrez/query.fcgi?db=Nucleotide&term=NR_040084) | hs\|21q22.12 |
| 0.0052933 | 0.137 | 0.031746 | 1.29 | 0.56 | 0.43 | [MT1E](http://www.ncbi.nlm.nih.gov/entrez/query.fcgi?cmd=search&db=gene&term=MT1E) | metallothionein 1E | [NM_175617](http://www.ncbi.nlm.nih.gov/entrez/query.fcgi?db=Nucleotide&term=NM_175617) | hs\|16q12.2 |
| 0.0027423 | 0.137 | 0.015873 | 1.86 | 0.8 | 0.43 | [lnc-ID3-4](http://www.ncbi.nlm.nih.gov/entrez/query.fcgi?cmd=search&db=gene&term=lnc-ID3-4) | lnc-ID3-4:1 |  | hs\|1p36.11 |
| 0.0095241 | 0.143 | 0.0079365 | 1.5 | 0.65 | 0.43 |  |  |  | hs\|14q24.3 |
| 0.008922 | 0.142 | 0.0238095 | 1.9 | 0.82 | 0.43 | [FAM131C](http://www.ncbi.nlm.nih.gov/entrez/query.fcgi?cmd=search&db=gene&term=FAM131C) | family with sequence similarity 131, member C | [NM_182623](http://www.ncbi.nlm.nih.gov/entrez/query.fcgi?db=Nucleotide&term=NM_182623) | hs\|1p36.13 |
| 0.0005296 | 0.137 | 0.0079365 | 1.76 | 0.75 | 0.43 | [IPO5P1](http://www.ncbi.nlm.nih.gov/entrez/query.fcgi?cmd=search&db=gene&term=IPO5P1) | importin 5 pseudogene 1 | [NR_103742](http://www.ncbi.nlm.nih.gov/entrez/query.fcgi?db=Nucleotide&term=NR_103742) | hs\|19p12 |
| 0.0093446 | 0.143 | 0.0396825 | 1.41 | 0.6 | 0.43 | [lnc-RP11-17M16.1.1-7](http://www.ncbi.nlm.nih.gov/entrez/query.fcgi?cmd=search&db=gene&term=lnc-RP11-17M16.1.1-7) | lnc-RP11-17M16.1.1-7:3 |  | hs\|18q23 |
| 0.0021027 | 0.137 | 0.0079365 | 1.94 | 0.83 | 0.43 | [lnc-ZSCAN10-3](http://www.ncbi.nlm.nih.gov/entrez/query.fcgi?cmd=search&db=gene&term=lnc-ZSCAN10-3) | lnc-ZSCAN10-3:6 | [DB087096](http://www.ncbi.nlm.nih.gov/entrez/query.fcgi?db=Nucleotide&term=DB087096) | hs\|16p13.3 |
| 0.002892 | 0.137 | 0.015873 | 1.49 | 0.64 | 0.43 | [MPV17L](http://www.ncbi.nlm.nih.gov/entrez/query.fcgi?cmd=search&db=gene&term=MPV17L) | MPV17 mitochondrial membrane protein-like | [AF075063](http://www.ncbi.nlm.nih.gov/entrez/query.fcgi?db=Nucleotide&term=AF075063) | hs\|16p13.11 |
| 0.0042829 | 0.137 | 0.031746 | 1.22 | 0.52 | 0.43 | [MT1A](http://www.ncbi.nlm.nih.gov/entrez/query.fcgi?cmd=search&db=gene&term=MT1A) | metallothionein 1A | [NM_005946](http://www.ncbi.nlm.nih.gov/entrez/query.fcgi?db=Nucleotide&term=NM_005946) | hs\|16q12.2 |
| 0.0057152 | 0.137 | 0.0238095 | 2.06 | 0.88 | 0.43 | [NOMO1](http://www.ncbi.nlm.nih.gov/entrez/query.fcgi?cmd=search&db=gene&term=NOMO1) | NODAL modulator 1 | [NM_014287](http://www.ncbi.nlm.nih.gov/entrez/query.fcgi?db=Nucleotide&term=NM_014287) | hs\|16p13.11 |
| 0.0008572 | 0.137 | 0.0079365 | 1.8 | 0.77 | 0.43 | [XLOC_l2_004817](http://www.ncbi.nlm.nih.gov/entrez/query.fcgi?cmd=search&db=gene&term=XLOC_l2_004817) |  |  | hs\|15q26.3 |
| 0.0010038 | 0.137 | 0.0079365 | 1.95 | 0.84 | 0.43 |  |  |  | hs\|12q24.31 |
| 0.0085839 | 0.142 | 0.031746 | 1.8 | 0.77 | 0.43 |  |  |  | hs\|1q25.3 |
| 0.0036715 | 0.137 | 0.0238095 | 1.62 | 0.69 | 0.43 | [DISC1-IT1](http://www.ncbi.nlm.nih.gov/entrez/query.fcgi?cmd=search&db=gene&term=DISC1-IT1) | DISC1 intronic transcript 1 (non-protein coding) | [NR_126441](http://www.ncbi.nlm.nih.gov/entrez/query.fcgi?db=Nucleotide&term=NR_126441) | hs\|1q42.2 |
| 0.0010015 | 0.137 | 0.0079365 | 1.84 | 0.78 | 0.43 | [FLJ36777](http://www.ncbi.nlm.nih.gov/entrez/query.fcgi?cmd=search&db=gene&term=FLJ36777) | uncharacterized LOC730971 | [AK094096](http://www.ncbi.nlm.nih.gov/entrez/query.fcgi?db=Nucleotide&term=AK094096) | hs\|4p16.1 |
| 0.0010411 | 0.137 | 0.015873 | 1.9 | 0.81 | 0.43 | [TMC3-AS1](http://www.ncbi.nlm.nih.gov/entrez/query.fcgi?cmd=search&db=gene&term=TMC3-AS1) | TMC3 antisense RNA 1 | [NR_120365](http://www.ncbi.nlm.nih.gov/entrez/query.fcgi?db=Nucleotide&term=NR_120365) | hs\|15q25.1 |
| 0.0056758 | 0.137 | 0.0238095 | 1.36 | 0.58 | 0.42 | [LINC01354](http://www.ncbi.nlm.nih.gov/entrez/query.fcgi?cmd=search&db=gene&term=LINC01354) | long intergenic non-protein coding RNA 1354 |  | hs\|1q42.2 |
| 0.0030103 | 0.137 | 0.015873 | 1.65 | 0.7 | 0.42 | [LOC101928565](http://www.ncbi.nlm.nih.gov/entrez/query.fcgi?cmd=search&db=gene&term=LOC101928565) | uncharacterized LOC101928565 |  | hs\|1q24.2 |
| 0.0021564 | 0.137 | 0.015873 | 1.71 | 0.73 | 0.42 | [PON3](http://www.ncbi.nlm.nih.gov/entrez/query.fcgi?cmd=search&db=gene&term=PON3) | paraoxonase 3 | [NM_000940](http://www.ncbi.nlm.nih.gov/entrez/query.fcgi?db=Nucleotide&term=NM_000940) | hs\|7q21.3 |
| 0.0061073 | 0.137 | 0.0238095 | 1.44 | 0.61 | 0.42 | [GSTA4](http://www.ncbi.nlm.nih.gov/entrez/query.fcgi?cmd=search&db=gene&term=GSTA4) | glutathione S-transferase alpha 4 | [NM_001512](http://www.ncbi.nlm.nih.gov/entrez/query.fcgi?db=Nucleotide&term=NM_001512) | hs\|6p12.2 |
| 0.0022679 | 0.137 | 0.015873 | 1.95 | 0.82 | 0.42 | [RLTPR](http://www.ncbi.nlm.nih.gov/entrez/query.fcgi?cmd=search&db=gene&term=RLTPR) | RGD motif, leucine rich repeats, tropomodulin domain and proline-rich containing | [NM_001013838](http://www.ncbi.nlm.nih.gov/entrez/query.fcgi?db=Nucleotide&term=NM_001013838) | hs\|16q22.1 |
| 0.0092545 | 0.143 | 0.0079365 | 1.3 | 0.54 | 0.42 | [TNXB](http://www.ncbi.nlm.nih.gov/entrez/query.fcgi?cmd=search&db=gene&term=TNXB) | tenascin XB | [NM_019105](http://www.ncbi.nlm.nih.gov/entrez/query.fcgi?db=Nucleotide&term=NM_019105) | hs\|6p21.33 |
| 0.0053553 | 0.137 | 0.015873 | 1.91 | 0.8 | 0.42 | [ACR](http://www.ncbi.nlm.nih.gov/entrez/query.fcgi?cmd=search&db=gene&term=ACR) | acrosin | [NM_001097](http://www.ncbi.nlm.nih.gov/entrez/query.fcgi?db=Nucleotide&term=NM_001097) | hs\|22q13.33 |
| 0.0085239 | 0.142 | 0.031746 | 1.68 | 0.7 | 0.42 | [C20orf141](http://www.ncbi.nlm.nih.gov/entrez/query.fcgi?cmd=search&db=gene&term=C20orf141) | chromosome 20 open reading frame 141 | [NM_080739](http://www.ncbi.nlm.nih.gov/entrez/query.fcgi?db=Nucleotide&term=NM_080739) | hs\|20p13 |
| 0.0055283 | 0.137 | 0.0079365 | 1.46 | 0.61 | 0.42 | [GADD45G](http://www.ncbi.nlm.nih.gov/entrez/query.fcgi?cmd=search&db=gene&term=GADD45G) | growth arrest and DNA-damage-inducible, gamma | [NM_006705](http://www.ncbi.nlm.nih.gov/entrez/query.fcgi?db=Nucleotide&term=NM_006705) | hs\|9q22.2 |
| 0.0050261 | 0.137 | 0.031746 | 1.98 | 0.83 | 0.42 | [KRTAP2-1](http://www.ncbi.nlm.nih.gov/entrez/query.fcgi?cmd=search&db=gene&term=KRTAP2-1) | keratin associated protein 2-1 | [NM_001123387](http://www.ncbi.nlm.nih.gov/entrez/query.fcgi?db=Nucleotide&term=NM_001123387) | hs\|17q21.2 |
| 0.0019382 | 0.137 | 0.0238095 | 2.01 | 0.83 | 0.41 | [lnc-CCDC8-1](http://www.ncbi.nlm.nih.gov/entrez/query.fcgi?cmd=search&db=gene&term=lnc-CCDC8-1) | lnc-CCDC8-1:1 | [AK123720](http://www.ncbi.nlm.nih.gov/entrez/query.fcgi?db=Nucleotide&term=AK123720) | hs\|19q13.32 |
| 8.98E-05 | 0.137 | 0.0079365 | 1.54 | 0.64 | 0.41 | [lnc-SRR-2](http://www.ncbi.nlm.nih.gov/entrez/query.fcgi?cmd=search&db=gene&term=lnc-SRR-2) | lnc-SRR-2:1 |  | hs\|17p13.3 |
| 0.0051693 | 0.137 | 0.015873 | 1.77 | 0.74 | 0.41 | [lnc-SSTR4-2](http://www.ncbi.nlm.nih.gov/entrez/query.fcgi?cmd=search&db=gene&term=lnc-SSTR4-2) | lnc-SSTR4-2:4 |  | hs\|20p11.21 |
| 0.0078797 | 0.14 | 0.015873 | 1.49 | 0.62 | 0.41 | [UCKL1-AS1](http://www.ncbi.nlm.nih.gov/entrez/query.fcgi?cmd=search&db=gene&term=UCKL1-AS1) | UCKL1 antisense RNA 1 | [NR_027287](http://www.ncbi.nlm.nih.gov/entrez/query.fcgi?db=Nucleotide&term=NR_027287) | hs\|20q13.33 |
| 0.0035526 | 0.137 | 0.0079365 | 1.56 | 0.65 | 0.41 | [VSTM2A](http://www.ncbi.nlm.nih.gov/entrez/query.fcgi?cmd=search&db=gene&term=VSTM2A) | V-set and transmembrane domain containing 2A | [NM_001301009](http://www.ncbi.nlm.nih.gov/entrez/query.fcgi?db=Nucleotide&term=NM_001301009) | hs\|7p11.2 |
| 0.0094743 | 0.143 | 0.031746 | 1.73 | 0.72 | 0.41 |  |  |  | hs\|3q12.1 |
| 0.0042437 | 0.137 | 0.031746 | 1.96 | 0.81 | 0.41 | [lnc-DTNBP1-2](http://www.ncbi.nlm.nih.gov/entrez/query.fcgi?cmd=search&db=gene&term=lnc-DTNBP1-2) | lnc-DTNBP1-2:2 |  | hs\|6p22.3 |
| 0.0089113 | 0.142 | 0.031746 | 1.68 | 0.69 | 0.41 | [LOC646513](http://www.ncbi.nlm.nih.gov/entrez/query.fcgi?cmd=search&db=gene&term=LOC646513) | VLGN1945 | [XR_159371](http://www.ncbi.nlm.nih.gov/entrez/query.fcgi?db=Nucleotide&term=XR_159371) | hs\|22q12.2 |
| 0.0095991 | 0.144 | 0.031746 | 1.92 | 0.79 | 0.41 | [SYNGR3](http://www.ncbi.nlm.nih.gov/entrez/query.fcgi?cmd=search&db=gene&term=SYNGR3) | synaptogyrin 3 | [NM_004209](http://www.ncbi.nlm.nih.gov/entrez/query.fcgi?db=Nucleotide&term=NM_004209) | hs\|16p13.3 |
| 0.0012579 | 0.137 | 0.0079365 | 2.09 | 0.86 | 0.41 | [TNR](http://www.ncbi.nlm.nih.gov/entrez/query.fcgi?cmd=search&db=gene&term=TNR) | tenascin R | [NM_003285](http://www.ncbi.nlm.nih.gov/entrez/query.fcgi?db=Nucleotide&term=NM_003285) | hs\|1q25.1 |
| 0.0052077 | 0.137 | 0.0079365 | 1.35 | 0.56 | 0.41 | [ANKRD20A2](http://www.ncbi.nlm.nih.gov/entrez/query.fcgi?cmd=search&db=gene&term=ANKRD20A2) | ankyrin repeat domain 20 family, member A2 | [NM_001012421](http://www.ncbi.nlm.nih.gov/entrez/query.fcgi?db=Nucleotide&term=NM_001012421) | hs\|9p12 |
| 0.0021033 | 0.137 | 0.0238095 | 1.83 | 0.75 | 0.41 |  |  |  | hs\|9q22.32 |
| 0.0039319 | 0.137 | 0.015873 | 2.07 | 0.85 | 0.41 |  |  |  | hs\|19q13.2 |
| 0.0084152 | 0.141 | 0.0238095 | 1.39 | 0.57 | 0.41 | [ACACB](http://www.ncbi.nlm.nih.gov/entrez/query.fcgi?cmd=search&db=gene&term=ACACB) | acetyl-CoA carboxylase beta | [NM_001093](http://www.ncbi.nlm.nih.gov/entrez/query.fcgi?db=Nucleotide&term=NM_001093) | hs\|12q24.11 |
| 0.005028 | 0.137 | 0.0238095 | 2.04 | 0.84 | 0.41 | [CHST13](http://www.ncbi.nlm.nih.gov/entrez/query.fcgi?cmd=search&db=gene&term=CHST13) | carbohydrate (chondroitin 4) sulfotransferase 13 | [NM_152889](http://www.ncbi.nlm.nih.gov/entrez/query.fcgi?db=Nucleotide&term=NM_152889) | hs\|3q21.3 |
| 0.0085986 | 0.142 | 0.0079365 | 1.39 | 0.57 | 0.41 | [FOXP2](http://www.ncbi.nlm.nih.gov/entrez/query.fcgi?cmd=search&db=gene&term=FOXP2) | forkhead box P2 | [NM_148900](http://www.ncbi.nlm.nih.gov/entrez/query.fcgi?db=Nucleotide&term=NM_148900) | hs\|7q31.1 |
| 0.0061166 | 0.137 | 0.0396825 | 2.08 | 0.85 | 0.41 | [TMEM221](http://www.ncbi.nlm.nih.gov/entrez/query.fcgi?cmd=search&db=gene&term=TMEM221) | transmembrane protein 221 |  | hs\|19p13.11 |
| 0.0054682 | 0.137 | 0.0238095 | 1.85 | 0.76 | 0.41 | [UPK3B](http://www.ncbi.nlm.nih.gov/entrez/query.fcgi?cmd=search&db=gene&term=UPK3B) | uroplakin 3B | [NM_030570](http://www.ncbi.nlm.nih.gov/entrez/query.fcgi?db=Nucleotide&term=NM_030570) | hs\|7q11.23 |
| 0.00482 | 0.137 | 0.015873 | 2.1 | 0.86 | 0.41 |  |  |  | hs\|5p15.33 |
| 0.0034432 | 0.137 | 0.0238095 | 1.92 | 0.79 | 0.41 | [lnc-LAMA1-5](http://www.ncbi.nlm.nih.gov/entrez/query.fcgi?cmd=search&db=gene&term=lnc-LAMA1-5) | lnc-LAMA1-5:2 |  | hs\|18p11.23 |
| 0.0071014 | 0.138 | 0.015873 | 1.98 | 0.81 | 0.41 | [SPEM1](http://www.ncbi.nlm.nih.gov/entrez/query.fcgi?cmd=search&db=gene&term=SPEM1) | spermatid maturation 1 | [AK097400](http://www.ncbi.nlm.nih.gov/entrez/query.fcgi?db=Nucleotide&term=AK097400) | hs\|17p13.1 |
| 0.0020622 | 0.137 | 0.0079365 | 1.66 | 0.68 | 0.41 | [TMEM145](http://www.ncbi.nlm.nih.gov/entrez/query.fcgi?cmd=search&db=gene&term=TMEM145) | transmembrane protein 145 | [NM_173633](http://www.ncbi.nlm.nih.gov/entrez/query.fcgi?db=Nucleotide&term=NM_173633) | hs\|19q13.2 |
| 0.006948 | 0.138 | 0.0238095 | 1.56 | 0.64 | 0.41 |  |  | [BC017738](http://www.ncbi.nlm.nih.gov/entrez/query.fcgi?db=Nucleotide&term=BC017738) | hs\|1p36.33 |
| 0.0008669 | 0.137 | 0.0079365 | 1.56 | 0.63 | 0.41 | [LOC101930053](http://www.ncbi.nlm.nih.gov/entrez/query.fcgi?cmd=search&db=gene&term=LOC101930053) | uncharacterized LOC101930053 | [XR_428440](http://www.ncbi.nlm.nih.gov/entrez/query.fcgi?db=Nucleotide&term=XR_428440) | hs\|9p24.2 |
| 0.0023139 | 0.137 | 0.015873 | 1.83 | 0.74 | 0.41 | [SSX3](http://www.ncbi.nlm.nih.gov/entrez/query.fcgi?cmd=search&db=gene&term=SSX3) | synovial sarcoma, X breakpoint 3 | [NM_021014](http://www.ncbi.nlm.nih.gov/entrez/query.fcgi?db=Nucleotide&term=NM_021014) | hs\|Xp11.23 |
| 0.0095865 | 0.144 | 0.015873 | 1.77 | 0.72 | 0.41 |  |  |  | hs\|16p11.2 |
| 0.0041687 | 0.137 | 0.015873 | 1.6 | 0.65 | 0.4 | [lnc-TAF12-3](http://www.ncbi.nlm.nih.gov/entrez/query.fcgi?cmd=search&db=gene&term=lnc-TAF12-3) | lnc-TAF12-3:2 |  | hs\|1p35.3 |
| 0.0004324 | 0.137 | 0.0079365 | 1.59 | 0.64 | 0.4 | [TDRD1](http://www.ncbi.nlm.nih.gov/entrez/query.fcgi?cmd=search&db=gene&term=TDRD1) | tudor domain containing 1 | [NM_198795](http://www.ncbi.nlm.nih.gov/entrez/query.fcgi?db=Nucleotide&term=NM_198795) | hs\|10q25.3 |
| 0.0059218 | 0.137 | 0.0079365 | 1.9 | 0.77 | 0.4 | [WDFY4](http://www.ncbi.nlm.nih.gov/entrez/query.fcgi?cmd=search&db=gene&term=WDFY4) | WDFY family member 4 | [NM_020945](http://www.ncbi.nlm.nih.gov/entrez/query.fcgi?db=Nucleotide&term=NM_020945) | hs\|10q11.23 |
| 0.0020187 | 0.137 | 0.015873 | 1.77 | 0.72 | 0.4 |  |  |  | hs\|10q23.1 |
| 0.0062944 | 0.137 | 0.0238095 | 1.31 | 0.53 | 0.4 | [DMGDH](http://www.ncbi.nlm.nih.gov/entrez/query.fcgi?cmd=search&db=gene&term=DMGDH) | dimethylglycine dehydrogenase | [NM_013391](http://www.ncbi.nlm.nih.gov/entrez/query.fcgi?db=Nucleotide&term=NM_013391) | hs\|5q14.1 |
| 0.0023593 | 0.137 | 0.0079365 | 1.44 | 0.58 | 0.4 | [FOXP2](http://www.ncbi.nlm.nih.gov/entrez/query.fcgi?cmd=search&db=gene&term=FOXP2) | forkhead box P2 | [NM_014491](http://www.ncbi.nlm.nih.gov/entrez/query.fcgi?db=Nucleotide&term=NM_014491) | hs\|7q31.1 |
| 0.0096438 | 0.144 | 0.0238095 | 1.56 | 0.63 | 0.4 | [HSPB6](http://www.ncbi.nlm.nih.gov/entrez/query.fcgi?cmd=search&db=gene&term=HSPB6) | heat shock protein, alpha-crystallin-related, B6 | [NM_144617](http://www.ncbi.nlm.nih.gov/entrez/query.fcgi?db=Nucleotide&term=NM_144617) | hs\|19q13.12 |
| 0.0059091 | 0.137 | 0.031746 | 1.98 | 0.8 | 0.4 | [LINC00423](http://www.ncbi.nlm.nih.gov/entrez/query.fcgi?cmd=search&db=gene&term=LINC00423) | long intergenic non-protein coding RNA 423 |  | hs\|13q13.1 |
| 0.0079272 | 0.14 | 0.031746 | 1.94 | 0.78 | 0.4 |  |  |  | hs\|1p33 |
| 0.0021353 | 0.137 | 0.0079365 | 1.66 | 0.67 | 0.4 | [LOC388692](http://www.ncbi.nlm.nih.gov/entrez/query.fcgi?cmd=search&db=gene&term=LOC388692) | uncharacterized LOC388692 | [NR_111933](http://www.ncbi.nlm.nih.gov/entrez/query.fcgi?db=Nucleotide&term=NR_111933) | hs\|1q21.2 |
| 0.0030729 | 0.137 | 0.0238095 | 2.01 | 0.81 | 0.4 | [MOGAT3](http://www.ncbi.nlm.nih.gov/entrez/query.fcgi?cmd=search&db=gene&term=MOGAT3) | monoacylglycerol O-acyltransferase 3 | [NM_178176](http://www.ncbi.nlm.nih.gov/entrez/query.fcgi?db=Nucleotide&term=NM_178176) | hs\|7q22.1 |
| 0.0042173 | 0.137 | 0.0079365 | 2.02 | 0.81 | 0.4 | [ARHGAP4](http://www.ncbi.nlm.nih.gov/entrez/query.fcgi?cmd=search&db=gene&term=ARHGAP4) | Rho GTPase activating protein 4 | [NM_001164741](http://www.ncbi.nlm.nih.gov/entrez/query.fcgi?db=Nucleotide&term=NM_001164741) | hs\|Xq28 |
| 0.0066006 | 0.137 | 0.015873 | 1.73 | 0.69 | 0.4 | [lnc-AC118344.1-1](http://www.ncbi.nlm.nih.gov/entrez/query.fcgi?cmd=search&db=gene&term=lnc-AC118344.1-1) | lnc-AC118344.1-1:1 | [AY358191](http://www.ncbi.nlm.nih.gov/entrez/query.fcgi?db=Nucleotide&term=AY358191) | hs\|19q13.2 |
| 0.0051892 | 0.137 | 0.031746 | 2.22 | 0.88 | 0.4 | [LINC01210](http://www.ncbi.nlm.nih.gov/entrez/query.fcgi?cmd=search&db=gene&term=LINC01210) | long intergenic non-protein coding RNA 1210 |  | hs\|3q22.3 |
| 0.0059264 | 0.137 | 0.0238095 | 1.97 | 0.78 | 0.4 | [LOC151475](http://www.ncbi.nlm.nih.gov/entrez/query.fcgi?cmd=search&db=gene&term=LOC151475) | uncharacterized LOC151475 | [NR_040038](http://www.ncbi.nlm.nih.gov/entrez/query.fcgi?db=Nucleotide&term=NR_040038) | hs\|2q37.1 |
| 0.0050732 | 0.137 | 0.015873 | 1.71 | 0.68 | 0.4 | [TNNT3](http://www.ncbi.nlm.nih.gov/entrez/query.fcgi?cmd=search&db=gene&term=TNNT3) | troponin T type 3 (skeletal, fast) | [NM_001042780](http://www.ncbi.nlm.nih.gov/entrez/query.fcgi?db=Nucleotide&term=NM_001042780) | hs\|11p15.5 |
| 0.0084646 | 0.141 | 0.015873 | 2.09 | 0.83 | 0.4 |  |  |  | hs\|1p32.2 |
| 0.0052355 | 0.137 | 0.0238095 | 1.82 | 0.72 | 0.4 | [LGI3](http://www.ncbi.nlm.nih.gov/entrez/query.fcgi?cmd=search&db=gene&term=LGI3) | leucine-rich repeat LGI family, member 3 | [NM_139278](http://www.ncbi.nlm.nih.gov/entrez/query.fcgi?db=Nucleotide&term=NM_139278) | hs\|8p21.3 |
| 0.0043262 | 0.137 | 0.0238095 | 1.98 | 0.79 | 0.4 | [lnc-MIB2-1](http://www.ncbi.nlm.nih.gov/entrez/query.fcgi?cmd=search&db=gene&term=lnc-MIB2-1) | lnc-MIB2-1:2 |  | hs\|1p36.33 |
| 0.0044138 | 0.137 | 0.0238095 | 1.75 | 0.7 | 0.4 | [LOC100507564](http://www.ncbi.nlm.nih.gov/entrez/query.fcgi?cmd=search&db=gene&term=LOC100507564) | uncharacterized LOC100507564 | [NR_038953](http://www.ncbi.nlm.nih.gov/entrez/query.fcgi?db=Nucleotide&term=NR_038953) | hs\|1p32.3 |
| 0.0018199 | 0.137 | 0.0079365 | 1.39 | 0.55 | 0.4 | [CCDC69](http://www.ncbi.nlm.nih.gov/entrez/query.fcgi?cmd=search&db=gene&term=CCDC69) | coiled-coil domain containing 69 | [NM_015621](http://www.ncbi.nlm.nih.gov/entrez/query.fcgi?db=Nucleotide&term=NM_015621) | hs\|5q33.1 |
| 0.0045602 | 0.137 | 0.0238095 | 1.56 | 0.62 | 0.4 | [lnc-KATNAL2-4](http://www.ncbi.nlm.nih.gov/entrez/query.fcgi?cmd=search&db=gene&term=lnc-KATNAL2-4) | lnc-KATNAL2-4:1 |  | hs\|18q21.1 |
| 0.0017568 | 0.137 | 0.0079365 | 1.75 | 0.69 | 0.4 | [TNK2-AS1](http://www.ncbi.nlm.nih.gov/entrez/query.fcgi?cmd=search&db=gene&term=TNK2-AS1) | TNK2 antisense RNA 1 | [AK094115](http://www.ncbi.nlm.nih.gov/entrez/query.fcgi?db=Nucleotide&term=AK094115) | hs\|3q29 |
| 0.0028113 | 0.137 | 0.0238095 | 1.21 | 0.47 | 0.39 | [ADAM33](http://www.ncbi.nlm.nih.gov/entrez/query.fcgi?cmd=search&db=gene&term=ADAM33) | ADAM metallopeptidase domain 33 | [NM_153202](http://www.ncbi.nlm.nih.gov/entrez/query.fcgi?db=Nucleotide&term=NM_153202) | hs\|20p13 |
| 0.0028091 | 0.137 | 0.015873 | 1.72 | 0.67 | 0.39 | [lnc-KIAA1147-3](http://www.ncbi.nlm.nih.gov/entrez/query.fcgi?cmd=search&db=gene&term=lnc-KIAA1147-3) | lnc-KIAA1147-3:7 |  | hs\|7q34 |
| 0.0098723 | 0.144 | 0.031746 | 1.49 | 0.59 | 0.39 | [RUNDC3A](http://www.ncbi.nlm.nih.gov/entrez/query.fcgi?cmd=search&db=gene&term=RUNDC3A) | RUN domain containing 3A | [NM_006695](http://www.ncbi.nlm.nih.gov/entrez/query.fcgi?db=Nucleotide&term=NM_006695) | hs\|17q21.31 |
| 0.0026445 | 0.137 | 0.015873 | 2.22 | 0.87 | 0.39 | [SPRR2D](http://www.ncbi.nlm.nih.gov/entrez/query.fcgi?cmd=search&db=gene&term=SPRR2D) | small proline-rich protein 2D | [NM_006945](http://www.ncbi.nlm.nih.gov/entrez/query.fcgi?db=Nucleotide&term=NM_006945) | hs\|1q21.3 |
| 0.0028896 | 0.137 | 0.0238095 | 2 | 0.79 | 0.39 | [ZSCAN12P1](http://www.ncbi.nlm.nih.gov/entrez/query.fcgi?cmd=search&db=gene&term=ZSCAN12P1) | zinc finger and SCAN domain containing 12 pseudogene 1 | [NR_024063](http://www.ncbi.nlm.nih.gov/entrez/query.fcgi?db=Nucleotide&term=NR_024063) | hs\|6p22.1 |
| 0.0056916 | 0.137 | 0.031746 | 2.18 | 0.86 | 0.39 |  |  |  | hs\|15q13.1 |
| 0.0018237 | 0.137 | 0.015873 | 1.53 | 0.6 | 0.39 | [CDNF](http://www.ncbi.nlm.nih.gov/entrez/query.fcgi?cmd=search&db=gene&term=CDNF) | cerebral dopamine neurotrophic factor | [NM_001029954](http://www.ncbi.nlm.nih.gov/entrez/query.fcgi?db=Nucleotide&term=NM_001029954) | hs\|10p13 |
| 0.0062578 | 0.137 | 0.031746 | 2.1 | 0.82 | 0.39 | [DGKD](http://www.ncbi.nlm.nih.gov/entrez/query.fcgi?cmd=search&db=gene&term=DGKD) | diacylglycerol kinase, delta 130kDa | [NM_152879](http://www.ncbi.nlm.nih.gov/entrez/query.fcgi?db=Nucleotide&term=NM_152879) | hs\|2q37.1 |
| 0.0094714 | 0.143 | 0.0238095 | 2 | 0.79 | 0.39 | [HOTAIR](http://www.ncbi.nlm.nih.gov/entrez/query.fcgi?cmd=search&db=gene&term=HOTAIR) | HOX transcript antisense RNA | [NR_047517](http://www.ncbi.nlm.nih.gov/entrez/query.fcgi?db=Nucleotide&term=NR_047517) | hs\|12q13.13 |
| 0.0043435 | 0.137 | 0.015873 | 2.16 | 0.85 | 0.39 |  |  | [AF190162](http://www.ncbi.nlm.nih.gov/entrez/query.fcgi?db=Nucleotide&term=AF190162) | hs\|Xq26.3 |
| 0.0029579 | 0.137 | 0.015873 | 1.89 | 0.74 | 0.39 | [FAM129A](http://www.ncbi.nlm.nih.gov/entrez/query.fcgi?cmd=search&db=gene&term=FAM129A) | family with sequence similarity 129, member A | [NM_052966](http://www.ncbi.nlm.nih.gov/entrez/query.fcgi?db=Nucleotide&term=NM_052966) | hs\|1q25.3 |
| 0.0045608 | 0.137 | 0.031746 | 1.29 | 0.5 | 0.39 | [MT1HL1](http://www.ncbi.nlm.nih.gov/entrez/query.fcgi?cmd=search&db=gene&term=MT1HL1) | metallothionein 1H-like 1 | [NM_001276687](http://www.ncbi.nlm.nih.gov/entrez/query.fcgi?db=Nucleotide&term=NM_001276687) | hs\|1q43 |
| 0.0045978 | 0.137 | 0.0079365 | 1.78 | 0.69 | 0.39 | [FAM95B1](http://www.ncbi.nlm.nih.gov/entrez/query.fcgi?cmd=search&db=gene&term=FAM95B1) | family with sequence similarity 95, member B1 | [NR_026759](http://www.ncbi.nlm.nih.gov/entrez/query.fcgi?db=Nucleotide&term=NR_026759) | hs\|9p12 |
| 0.0028626 | 0.137 | 0.015873 | 2.02 | 0.78 | 0.39 | [LOC101928760](http://www.ncbi.nlm.nih.gov/entrez/query.fcgi?cmd=search&db=gene&term=LOC101928760) | uncharacterized LOC101928760 | [XR_245260](http://www.ncbi.nlm.nih.gov/entrez/query.fcgi?db=Nucleotide&term=XR_245260) | hs\|4q12 |
| 0.0035721 | 0.137 | 0.0238095 | 1.93 | 0.74 | 0.39 | [LOC101928324](http://www.ncbi.nlm.nih.gov/entrez/query.fcgi?cmd=search&db=gene&term=LOC101928324) | uncharacterized LOC101928324 | [NR_125953](http://www.ncbi.nlm.nih.gov/entrez/query.fcgi?db=Nucleotide&term=NR_125953) | hs\|1p36.11 |
| 0.00586 | 0.137 | 0.0079365 | 2.2 | 0.85 | 0.39 | [MKNK1](http://www.ncbi.nlm.nih.gov/entrez/query.fcgi?cmd=search&db=gene&term=MKNK1) | MAP kinase interacting serine/threonine kinase 1 | [NM_003684](http://www.ncbi.nlm.nih.gov/entrez/query.fcgi?db=Nucleotide&term=NM_003684) | hs\|1p33 |
| 0.0051231 | 0.137 | 0.0238095 | 1.33 | 0.51 | 0.39 | [TMEM56](http://www.ncbi.nlm.nih.gov/entrez/query.fcgi?cmd=search&db=gene&term=TMEM56) | transmembrane protein 56 | [NM_152487](http://www.ncbi.nlm.nih.gov/entrez/query.fcgi?db=Nucleotide&term=NM_152487) | hs\|1p21.3 |
| 0.003187 | 0.137 | 0.0238095 | 1.96 | 0.75 | 0.38 | [IL27](http://www.ncbi.nlm.nih.gov/entrez/query.fcgi?cmd=search&db=gene&term=IL27) | interleukin 27 | [NM_145659](http://www.ncbi.nlm.nih.gov/entrez/query.fcgi?db=Nucleotide&term=NM_145659) | hs\|16p11.2 |
| 0.0025735 | 0.137 | 0.015873 | 1.68 | 0.64 | 0.38 | [LINC01352](http://www.ncbi.nlm.nih.gov/entrez/query.fcgi?cmd=search&db=gene&term=LINC01352) | long intergenic non-protein coding RNA 1352 | [NR_110797](http://www.ncbi.nlm.nih.gov/entrez/query.fcgi?db=Nucleotide&term=NR_110797) | hs\|1q41 |
| 0.0035617 | 0.137 | 0.0238095 | 2.05 | 0.78 | 0.38 | [lnc-TRPM6-4](http://www.ncbi.nlm.nih.gov/entrez/query.fcgi?cmd=search&db=gene&term=lnc-TRPM6-4) | lnc-TRPM6-4:1 |  | hs\|9q21.13 |
| 0.0092906 | 0.143 | 0.0396825 | 2.17 | 0.82 | 0.38 | [FAAHP1](http://www.ncbi.nlm.nih.gov/entrez/query.fcgi?cmd=search&db=gene&term=FAAHP1) | fatty acid amide hydrolase pseudogene 1 | [NR_045483](http://www.ncbi.nlm.nih.gov/entrez/query.fcgi?db=Nucleotide&term=NR_045483) | hs\|1p33 |
| 0.0044192 | 0.137 | 0.031746 | 2.13 | 0.81 | 0.38 | [FGFRL1](http://www.ncbi.nlm.nih.gov/entrez/query.fcgi?cmd=search&db=gene&term=FGFRL1) | fibroblast growth factor receptor-like 1 | [NM_001004356](http://www.ncbi.nlm.nih.gov/entrez/query.fcgi?db=Nucleotide&term=NM_001004356) | hs\|4p16.3 |
| 0.0078482 | 0.14 | 0.031746 | 2.38 | 0.9 | 0.38 | [ABCC10](http://www.ncbi.nlm.nih.gov/entrez/query.fcgi?cmd=search&db=gene&term=ABCC10) | ATP-binding cassette, sub-family C (CFTR/MRP), member 10 | [NM_033450](http://www.ncbi.nlm.nih.gov/entrez/query.fcgi?db=Nucleotide&term=NM_033450) | hs\|6p21.1 |
| 0.0001511 | 0.137 | 0.0079365 | 1.88 | 0.71 | 0.38 | [lnc-ALOX12B-1](http://www.ncbi.nlm.nih.gov/entrez/query.fcgi?cmd=search&db=gene&term=lnc-ALOX12B-1) | lnc-ALOX12B-1:1 |  | hs\|17p13.1 |
| 0.0082487 | 0.141 | 0.031746 | 2.16 | 0.82 | 0.38 | [PGP](http://www.ncbi.nlm.nih.gov/entrez/query.fcgi?cmd=search&db=gene&term=PGP) | phosphoglycolate phosphatase | [NM_001042371](http://www.ncbi.nlm.nih.gov/entrez/query.fcgi?db=Nucleotide&term=NM_001042371) | hs\|16p13.3 |
| 0.0086893 | 0.142 | 0.031746 | 1.87 | 0.71 | 0.38 | [STAC2](http://www.ncbi.nlm.nih.gov/entrez/query.fcgi?cmd=search&db=gene&term=STAC2) | SH3 and cysteine rich domain 2 | [NM_198993](http://www.ncbi.nlm.nih.gov/entrez/query.fcgi?db=Nucleotide&term=NM_198993) | hs\|17q12 |
| 0.002852 | 0.137 | 0.015873 | 2.03 | 0.76 | 0.38 | [CECR3](http://www.ncbi.nlm.nih.gov/entrez/query.fcgi?cmd=search&db=gene&term=CECR3) | cat eye syndrome chromosome region, candidate 3 (non-protein coding) | [NR_038398](http://www.ncbi.nlm.nih.gov/entrez/query.fcgi?db=Nucleotide&term=NR_038398) | hs\|22q11.1 |
| 0.0098684 | 0.144 | 0.0238095 | 1.22 | 0.46 | 0.38 | [CTTNBP2](http://www.ncbi.nlm.nih.gov/entrez/query.fcgi?cmd=search&db=gene&term=CTTNBP2) | cortactin binding protein 2 | [NM_033427](http://www.ncbi.nlm.nih.gov/entrez/query.fcgi?db=Nucleotide&term=NM_033427) | hs\|7q31.2 |
| 0.007477 | 0.139 | 0.0238095 | 1.87 | 0.71 | 0.38 | [JAG2](http://www.ncbi.nlm.nih.gov/entrez/query.fcgi?cmd=search&db=gene&term=JAG2) | jagged 2 | [NM_145159](http://www.ncbi.nlm.nih.gov/entrez/query.fcgi?db=Nucleotide&term=NM_145159) | hs\|14q32.33 |
| 0.0052102 | 0.137 | 0.0079365 | 1.82 | 0.69 | 0.38 | [MUSTN1](http://www.ncbi.nlm.nih.gov/entrez/query.fcgi?cmd=search&db=gene&term=MUSTN1) | musculoskeletal, embryonic nuclear protein 1 | [NM_205853](http://www.ncbi.nlm.nih.gov/entrez/query.fcgi?db=Nucleotide&term=NM_205853) | hs\|3p21.1 |
| 0.0090321 | 0.143 | 0.0238095 | 1.71 | 0.65 | 0.38 | [MYZAP](http://www.ncbi.nlm.nih.gov/entrez/query.fcgi?cmd=search&db=gene&term=MYZAP) | myocardial zonula adherens protein | [NM_001018100](http://www.ncbi.nlm.nih.gov/entrez/query.fcgi?db=Nucleotide&term=NM_001018100) | hs\|15q21.3 |
| 0.0022271 | 0.137 | 0.015873 | 1.93 | 0.72 | 0.38 | [lnc-MFAP4-4](http://www.ncbi.nlm.nih.gov/entrez/query.fcgi?cmd=search&db=gene&term=lnc-MFAP4-4) | lnc-MFAP4-4:1 |  | hs\|17p11.2 |
| 0.0035274 | 0.137 | 0.0079365 | 1.5 | 0.56 | 0.38 | [XLOC_l2_002729](http://www.ncbi.nlm.nih.gov/entrez/query.fcgi?cmd=search&db=gene&term=XLOC_l2_002729) |  |  | hs\|11q14.1 |
| 0.0076141 | 0.139 | 0.031746 | 2.2 | 0.83 | 0.38 | [XLOC_l2_009867](http://www.ncbi.nlm.nih.gov/entrez/query.fcgi?cmd=search&db=gene&term=XLOC_l2_009867) |  |  | hs\|3q21.3 |
| 0.005279 | 0.137 | 0.015873 | 1.78 | 0.67 | 0.37 | [COL27A1](http://www.ncbi.nlm.nih.gov/entrez/query.fcgi?cmd=search&db=gene&term=COL27A1) | collagen, type XXVII, alpha 1 | [NM_032888](http://www.ncbi.nlm.nih.gov/entrez/query.fcgi?db=Nucleotide&term=NM_032888) | hs\|9q32 |
| 0.005391 | 0.137 | 0.015873 | 1.96 | 0.73 | 0.37 | [DHRS12](http://www.ncbi.nlm.nih.gov/entrez/query.fcgi?cmd=search&db=gene&term=DHRS12) | dehydrogenase/reductase (SDR family) member 12 | [NM_024705](http://www.ncbi.nlm.nih.gov/entrez/query.fcgi?db=Nucleotide&term=NM_024705) | hs\|13q14.3 |
| 0.0057509 | 0.137 | 0.0238095 | 2.19 | 0.82 | 0.37 | [DTNA](http://www.ncbi.nlm.nih.gov/entrez/query.fcgi?cmd=search&db=gene&term=DTNA) | dystrobrevin, alpha | [NM_001128175](http://www.ncbi.nlm.nih.gov/entrez/query.fcgi?db=Nucleotide&term=NM_001128175) | hs\|18q12.1 |
| 0.0003814 | 0.137 | 0.0079365 | 1.5 | 0.56 | 0.37 | [HRC](http://www.ncbi.nlm.nih.gov/entrez/query.fcgi?cmd=search&db=gene&term=HRC) | histidine rich calcium binding protein | [NM_002152](http://www.ncbi.nlm.nih.gov/entrez/query.fcgi?db=Nucleotide&term=NM_002152) | hs\|19q13.33 |
| 0.0022515 | 0.137 | 0.015873 | 2.16 | 0.81 | 0.37 | [lnc-SYT13-2](http://www.ncbi.nlm.nih.gov/entrez/query.fcgi?cmd=search&db=gene&term=lnc-SYT13-2) | lnc-SYT13-2:1 |  | hs\|11p11.2 |
| 0.0011647 | 0.137 | 0.015873 | 2.18 | 0.81 | 0.37 | [SEC13](http://www.ncbi.nlm.nih.gov/entrez/query.fcgi?cmd=search&db=gene&term=SEC13) | SEC13 homolog (S. cerevisiae) | [AK095629](http://www.ncbi.nlm.nih.gov/entrez/query.fcgi?db=Nucleotide&term=AK095629) | hs\|3p25.3 |
| 0.0052447 | 0.137 | 0.031746 | 2.04 | 0.76 | 0.37 | [ZNF254](http://www.ncbi.nlm.nih.gov/entrez/query.fcgi?cmd=search&db=gene&term=ZNF254) | zinc finger protein 254 | [NM_001278663](http://www.ncbi.nlm.nih.gov/entrez/query.fcgi?db=Nucleotide&term=NM_001278663) | hs\|19p12 |
| 0.0069294 | 0.138 | 0.015873 | 1.83 | 0.68 | 0.37 | [GATA2-AS1](http://www.ncbi.nlm.nih.gov/entrez/query.fcgi?cmd=search&db=gene&term=GATA2-AS1) | GATA2 antisense RNA 1 | [AK024653](http://www.ncbi.nlm.nih.gov/entrez/query.fcgi?db=Nucleotide&term=AK024653) | hs\|3q21.3 |
| 0.0036829 | 0.137 | 0.031746 | 2.12 | 0.79 | 0.37 | [KANK3](http://www.ncbi.nlm.nih.gov/entrez/query.fcgi?cmd=search&db=gene&term=KANK3) | KN motif and ankyrin repeat domains 3 | [AY203940](http://www.ncbi.nlm.nih.gov/entrez/query.fcgi?db=Nucleotide&term=AY203940) | hs\|19p13.2 |
| 0.0025546 | 0.137 | 0.015873 | 1.62 | 0.6 | 0.37 | [KIF26A](http://www.ncbi.nlm.nih.gov/entrez/query.fcgi?cmd=search&db=gene&term=KIF26A) | kinesin family member 26A | [NM_015656](http://www.ncbi.nlm.nih.gov/entrez/query.fcgi?db=Nucleotide&term=NM_015656) | hs\|14q32.33 |
| 0.0064732 | 0.137 | 0.015873 | 1.73 | 0.64 | 0.37 | [LMF2](http://www.ncbi.nlm.nih.gov/entrez/query.fcgi?cmd=search&db=gene&term=LMF2) | lipase maturation factor 2 | [NM_033200](http://www.ncbi.nlm.nih.gov/entrez/query.fcgi?db=Nucleotide&term=NM_033200) | hs\|22q13.33 |
| 0.0016819 | 0.137 | 0.0079365 | 1.59 | 0.58 | 0.37 | [lnc-IL12RB2-1](http://www.ncbi.nlm.nih.gov/entrez/query.fcgi?cmd=search&db=gene&term=lnc-IL12RB2-1) | lnc-IL12RB2-1:1 | [AK123656](http://www.ncbi.nlm.nih.gov/entrez/query.fcgi?db=Nucleotide&term=AK123656) | hs\|1p31.3 |
| 0.0081154 | 0.141 | 0.0238095 | 2.38 | 0.88 | 0.37 | [SEL1L](http://www.ncbi.nlm.nih.gov/entrez/query.fcgi?cmd=search&db=gene&term=SEL1L) | sel-1 suppressor of lin-12-like (C. elegans) | [NM_005065](http://www.ncbi.nlm.nih.gov/entrez/query.fcgi?db=Nucleotide&term=NM_005065) | hs\|14q31.1 |
| 0.0075287 | 0.139 | 0.015873 | 1.17 | 0.43 | 0.37 |  |  | [XM_006726418](http://www.ncbi.nlm.nih.gov/entrez/query.fcgi?db=Nucleotide&term=XM_006726418) | hs\|5p15.33 |
| 0.0079624 | 0.14 | 0.015873 | 1.89 | 0.7 | 0.37 |  |  |  | hs\|2q33.3 |
| 0.0063358 | 0.137 | 0.0079365 | 1.91 | 0.7 | 0.37 | [lnc-DLX2-4](http://www.ncbi.nlm.nih.gov/entrez/query.fcgi?cmd=search&db=gene&term=lnc-DLX2-4) | lnc-DLX2-4:3 | [BQ944104](http://www.ncbi.nlm.nih.gov/entrez/query.fcgi?db=Nucleotide&term=BQ944104) | hs\|2q31.1 |
| 0.002244 | 0.137 | 0.0238095 | 2.06 | 0.76 | 0.37 |  |  |  | hs\|6q25.3 |
| 0.0056898 | 0.137 | 0.0238095 | 2.11 | 0.77 | 0.37 | [KRTAP3-1](http://www.ncbi.nlm.nih.gov/entrez/query.fcgi?cmd=search&db=gene&term=KRTAP3-1) | keratin associated protein 3-1 | [NM_031958](http://www.ncbi.nlm.nih.gov/entrez/query.fcgi?db=Nucleotide&term=NM_031958) | hs\|17q21.2 |
| 0.0009429 | 0.137 | 0.015873 | 2.14 | 0.79 | 0.37 | [lnc-CEACAM18-2](http://www.ncbi.nlm.nih.gov/entrez/query.fcgi?cmd=search&db=gene&term=lnc-CEACAM18-2) | lnc-CEACAM18-2:1 | [DB453801](http://www.ncbi.nlm.nih.gov/entrez/query.fcgi?db=Nucleotide&term=DB453801) | hs\|19q13.41 |
| 0.006538 | 0.137 | 0.0238095 | 2.07 | 0.76 | 0.37 | [LOC101927391](http://www.ncbi.nlm.nih.gov/entrez/query.fcgi?cmd=search&db=gene&term=LOC101927391) | uncharacterized LOC101927391 | [NR_110084](http://www.ncbi.nlm.nih.gov/entrez/query.fcgi?db=Nucleotide&term=NR_110084) | hs\|7p21.3 |
| 0.0008798 | 0.137 | 0.0079365 | 1.71 | 0.63 | 0.37 |  |  | [BC032117](http://www.ncbi.nlm.nih.gov/entrez/query.fcgi?db=Nucleotide&term=BC032117) | hs\|9q34.11 |
| 0.0035048 | 0.137 | 0.0238095 | 2.14 | 0.78 | 0.36 | [LOC145845](http://www.ncbi.nlm.nih.gov/entrez/query.fcgi?cmd=search&db=gene&term=LOC145845) | uncharacterized LOC145845 |  | hs\|15q14 |
| 0.0013596 | 0.137 | 0.0079365 | 2.32 | 0.84 | 0.36 | [SSPO](http://www.ncbi.nlm.nih.gov/entrez/query.fcgi?cmd=search&db=gene&term=SSPO) | SCO-spondin | [NM_198455](http://www.ncbi.nlm.nih.gov/entrez/query.fcgi?db=Nucleotide&term=NM_198455) | hs\|7q36.1 |
| 0.0028604 | 0.137 | 0.015873 | 2.26 | 0.82 | 0.36 | [CLHC1](http://www.ncbi.nlm.nih.gov/entrez/query.fcgi?cmd=search&db=gene&term=CLHC1) | clathrin heavy chain linker domain containing 1 |  | hs\|2p16.1 |
| 0.0098166 | 0.144 | 0.031746 | 1.78 | 0.65 | 0.36 | [lnc-WFIKKN2-1](http://www.ncbi.nlm.nih.gov/entrez/query.fcgi?cmd=search&db=gene&term=lnc-WFIKKN2-1) | lnc-WFIKKN2-1:5 |  | hs\|17q21.33 |
| 0.0010023 | 0.137 | 0.0079365 | 2.04 | 0.74 | 0.36 | [LOC286071](http://www.ncbi.nlm.nih.gov/entrez/query.fcgi?cmd=search&db=gene&term=LOC286071) | uncharacterized LOC286071 | [AK091759](http://www.ncbi.nlm.nih.gov/entrez/query.fcgi?db=Nucleotide&term=AK091759) | hs\|8q11.23 |
| 0.0034727 | 0.137 | 0.0238095 | 2.22 | 0.8 | 0.36 | [SSR4](http://www.ncbi.nlm.nih.gov/entrez/query.fcgi?cmd=search&db=gene&term=SSR4) | signal sequence receptor, delta | [NM_006280](http://www.ncbi.nlm.nih.gov/entrez/query.fcgi?db=Nucleotide&term=NM_006280) | hs\|Xq28 |
| 0.0058607 | 0.137 | 0.0396825 | 2.4 | 0.87 | 0.36 | [EYS](http://www.ncbi.nlm.nih.gov/entrez/query.fcgi?cmd=search&db=gene&term=EYS) | eyes shut homolog (Drosophila) | [NM_001142800](http://www.ncbi.nlm.nih.gov/entrez/query.fcgi?db=Nucleotide&term=NM_001142800) | hs\|6q12 |
| 0.0016637 | 0.137 | 0.0079365 | 1.72 | 0.62 | 0.36 | [LOC101929574](http://www.ncbi.nlm.nih.gov/entrez/query.fcgi?cmd=search&db=gene&term=LOC101929574) | uncharacterized LOC101929574 |  | hs\|10q23.1 |
| 0.00591 | 0.137 | 0.0396825 | 2.31 | 0.83 | 0.36 | [LOC100133985](http://www.ncbi.nlm.nih.gov/entrez/query.fcgi?cmd=search&db=gene&term=LOC100133985) | uncharacterized LOC100133985 | [NR_024444](http://www.ncbi.nlm.nih.gov/entrez/query.fcgi?db=Nucleotide&term=NR_024444) | hs\|2p13.3 |
| 0.0015066 | 0.137 | 0.0238095 | 1.34 | 0.48 | 0.36 | [MT1E](http://www.ncbi.nlm.nih.gov/entrez/query.fcgi?cmd=search&db=gene&term=MT1E) | metallothionein 1E | [NM_175617](http://www.ncbi.nlm.nih.gov/entrez/query.fcgi?db=Nucleotide&term=NM_175617) | hs\|16q12.2 |
| 0.0029111 | 0.137 | 0.0079365 | 2.34 | 0.84 | 0.36 | [SSPO](http://www.ncbi.nlm.nih.gov/entrez/query.fcgi?cmd=search&db=gene&term=SSPO) | SCO-spondin | [NM_198455](http://www.ncbi.nlm.nih.gov/entrez/query.fcgi?db=Nucleotide&term=NM_198455) | hs\|7q36.1 |
| 0.001085 | 0.137 | 0.0079365 | 1.96 | 0.7 | 0.36 | [XBP1](http://www.ncbi.nlm.nih.gov/entrez/query.fcgi?cmd=search&db=gene&term=XBP1) | X-box binding protein 1 | [NM_005080](http://www.ncbi.nlm.nih.gov/entrez/query.fcgi?db=Nucleotide&term=NM_005080) | hs\|22q12.1 |
| 0.0095357 | 0.143 | 0.031746 | 1.29 | 0.46 | 0.36 | [CXCL12](http://www.ncbi.nlm.nih.gov/entrez/query.fcgi?cmd=search&db=gene&term=CXCL12) | chemokine (C-X-C motif) ligand 12 | [NM_199168](http://www.ncbi.nlm.nih.gov/entrez/query.fcgi?db=Nucleotide&term=NM_199168) | hs\|10q11.21 |
| 0.0071604 | 0.138 | 0.0238095 | 1.49 | 0.53 | 0.36 | [FAM181B](http://www.ncbi.nlm.nih.gov/entrez/query.fcgi?cmd=search&db=gene&term=FAM181B) | family with sequence similarity 181, member B | [NM_175885](http://www.ncbi.nlm.nih.gov/entrez/query.fcgi?db=Nucleotide&term=NM_175885) | hs\|11q14.1 |
| 0.0021168 | 0.137 | 0.0079365 | 1.62 | 0.58 | 0.36 | [LOC100506834](http://www.ncbi.nlm.nih.gov/entrez/query.fcgi?cmd=search&db=gene&term=LOC100506834) | uncharacterized LOC100506834 | [NR_049794](http://www.ncbi.nlm.nih.gov/entrez/query.fcgi?db=Nucleotide&term=NR_049794) | hs\|9q21.33 |
| 0.0055196 | 0.137 | 0.015873 | 1.94 | 0.69 | 0.36 | [NUPR1](http://www.ncbi.nlm.nih.gov/entrez/query.fcgi?cmd=search&db=gene&term=NUPR1) | nuclear protein, transcriptional regulator, 1 | [NM_001042483](http://www.ncbi.nlm.nih.gov/entrez/query.fcgi?db=Nucleotide&term=NM_001042483) | hs\|16p11.2 |
| 0.0046963 | 0.137 | 0.0238095 | 1.98 | 0.71 | 0.36 | [PDCD4](http://www.ncbi.nlm.nih.gov/entrez/query.fcgi?cmd=search&db=gene&term=PDCD4) | programmed cell death 4 (neoplastic transformation inhibitor) | [NM_145341](http://www.ncbi.nlm.nih.gov/entrez/query.fcgi?db=Nucleotide&term=NM_145341) | hs\|10q25.2 |
| 0.0013793 | 0.137 | 0.015873 | 2.01 | 0.72 | 0.36 | [XLOC_l2_015593](http://www.ncbi.nlm.nih.gov/entrez/query.fcgi?cmd=search&db=gene&term=XLOC_l2_015593) |  |  | hs\|Xq22.3 |
| 0.0087272 | 0.142 | 0.0238095 | 1.23 | 0.44 | 0.36 | [ALDH1A1](http://www.ncbi.nlm.nih.gov/entrez/query.fcgi?cmd=search&db=gene&term=ALDH1A1) | aldehyde dehydrogenase 1 family, member A1 | [NM_000689](http://www.ncbi.nlm.nih.gov/entrez/query.fcgi?db=Nucleotide&term=NM_000689) | hs\|9q21.13 |
| 0.000722 | 0.137 | 0.0079365 | 1.27 | 0.45 | 0.36 | [ITIH5](http://www.ncbi.nlm.nih.gov/entrez/query.fcgi?cmd=search&db=gene&term=ITIH5) | inter-alpha-trypsin inhibitor heavy chain family, member 5 | [NM_001001851](http://www.ncbi.nlm.nih.gov/entrez/query.fcgi?db=Nucleotide&term=NM_001001851) | hs\|10p14 |
| 0.0051391 | 0.137 | 0.031746 | 2.37 | 0.84 | 0.36 | [lnc-KCNH8-1](http://www.ncbi.nlm.nih.gov/entrez/query.fcgi?cmd=search&db=gene&term=lnc-KCNH8-1) | lnc-KCNH8-1:1 |  | hs\|3p24.3 |
| 8.63E-05 | 0.137 | 0.0079365 | 1.85 | 0.66 | 0.36 | [LOC102724330](http://www.ncbi.nlm.nih.gov/entrez/query.fcgi?cmd=search&db=gene&term=LOC102724330) | uncharacterized LOC102724330 | [XR_428344](http://www.ncbi.nlm.nih.gov/entrez/query.fcgi?db=Nucleotide&term=XR_428344) | hs\|8q11.23 |
| 0.0085933 | 0.142 | 0.0238095 | 1.67 | 0.59 | 0.36 | [MYEF2](http://www.ncbi.nlm.nih.gov/entrez/query.fcgi?cmd=search&db=gene&term=MYEF2) | myelin expression factor 2 | [NM_016132](http://www.ncbi.nlm.nih.gov/entrez/query.fcgi?db=Nucleotide&term=NM_016132) | hs\|15q21.1 |
| 0.002074 | 0.137 | 0.015873 | 2.17 | 0.77 | 0.35 | [LOC100034248](http://www.ncbi.nlm.nih.gov/entrez/query.fcgi?cmd=search&db=gene&term=LOC100034248) | uncharacterized LOC100034248 | [AK128820](http://www.ncbi.nlm.nih.gov/entrez/query.fcgi?db=Nucleotide&term=AK128820) | hs\|19p13.2 |
| 0.0042199 | 0.137 | 0.031746 | 1.33 | 0.47 | 0.35 | [MT1B](http://www.ncbi.nlm.nih.gov/entrez/query.fcgi?cmd=search&db=gene&term=MT1B) | metallothionein 1B | [NM_005947](http://www.ncbi.nlm.nih.gov/entrez/query.fcgi?db=Nucleotide&term=NM_005947) | hs\|16q12.2 |
| 0.0057092 | 0.137 | 0.0238095 | 2.13 | 0.75 | 0.35 | [HYKK](http://www.ncbi.nlm.nih.gov/entrez/query.fcgi?cmd=search&db=gene&term=HYKK) | hydroxylysine kinase | [NM_001083612](http://www.ncbi.nlm.nih.gov/entrez/query.fcgi?db=Nucleotide&term=NM_001083612) | hs\|15q25.1 |
| 0.0010833 | 0.137 | 0.0079365 | 1.63 | 0.58 | 0.35 | [LOC102723927](http://www.ncbi.nlm.nih.gov/entrez/query.fcgi?cmd=search&db=gene&term=LOC102723927) | uncharacterized LOC102723927 |  | hs\|2q37.3 |
| 0.0045704 | 0.137 | 0.0396825 | 2.32 | 0.82 | 0.35 | [lnc-POLR2L-1](http://www.ncbi.nlm.nih.gov/entrez/query.fcgi?cmd=search&db=gene&term=lnc-POLR2L-1) | lnc-POLR2L-1:2 |  | hs\|11p15.5 |
| 0.0039962 | 0.137 | 0.015873 | 1.94 | 0.68 | 0.35 | [lnc-TPCN2-5](http://www.ncbi.nlm.nih.gov/entrez/query.fcgi?cmd=search&db=gene&term=lnc-TPCN2-5) | lnc-TPCN2-5:1 | [AK124882](http://www.ncbi.nlm.nih.gov/entrez/query.fcgi?db=Nucleotide&term=AK124882) | hs\|11q13.3 |
| 0.0006047 | 0.137 | 0.0079365 | 1.7 | 0.6 | 0.35 | [LOC102723927](http://www.ncbi.nlm.nih.gov/entrez/query.fcgi?cmd=search&db=gene&term=LOC102723927) | uncharacterized LOC102723927 | [NR_110592](http://www.ncbi.nlm.nih.gov/entrez/query.fcgi?db=Nucleotide&term=NR_110592) | hs\|2q37.3 |
| 0.0010618 | 0.137 | 0.0079365 | 2.29 | 0.8 | 0.35 | [NIP7](http://www.ncbi.nlm.nih.gov/entrez/query.fcgi?cmd=search&db=gene&term=NIP7) | NIP7, nucleolar pre-rRNA processing protein | [NM_016101](http://www.ncbi.nlm.nih.gov/entrez/query.fcgi?db=Nucleotide&term=NM_016101) | hs\|16q22.1 |
| 0.0057902 | 0.137 | 0.0079365 | 2.12 | 0.75 | 0.35 | [PSMD6](http://www.ncbi.nlm.nih.gov/entrez/query.fcgi?cmd=search&db=gene&term=PSMD6) | proteasome (prosome, macropain) 26S subunit, non-ATPase, 6 | [NM_014814](http://www.ncbi.nlm.nih.gov/entrez/query.fcgi?db=Nucleotide&term=NM_014814) | hs\|3p14.1 |
| 0.0074532 | 0.139 | 0.0396825 | 2.13 | 0.75 | 0.35 |  |  | [DB525580](http://www.ncbi.nlm.nih.gov/entrez/query.fcgi?db=Nucleotide&term=DB525580) | hs\|7q11.21 |
| 0.001043 | 0.137 | 0.0079365 | 2.39 | 0.84 | 0.35 | [COL28A1](http://www.ncbi.nlm.nih.gov/entrez/query.fcgi?cmd=search&db=gene&term=COL28A1) | collagen, type XXVIII, alpha 1 | [NM_001037763](http://www.ncbi.nlm.nih.gov/entrez/query.fcgi?db=Nucleotide&term=NM_001037763) | hs\|7p21.3 |
| 0.0002816 | 0.137 | 0.0079365 | 1.84 | 0.64 | 0.35 | [LINC00689](http://www.ncbi.nlm.nih.gov/entrez/query.fcgi?cmd=search&db=gene&term=LINC00689) | long intergenic non-protein coding RNA 689 |  | hs\|7q36.3 |
| 0.0021034 | 0.137 | 0.0079365 | 2.11 | 0.74 | 0.35 |  |  |  | hs\|7q11.21 |
| 0.008627 | 0.142 | 0.0238095 | 1.59 | 0.55 | 0.35 | [SHC3](http://www.ncbi.nlm.nih.gov/entrez/query.fcgi?cmd=search&db=gene&term=SHC3) | SHC (Src homology 2 domain containing) transforming protein 3 | [NM_016848](http://www.ncbi.nlm.nih.gov/entrez/query.fcgi?db=Nucleotide&term=NM_016848) | hs\|9q22.1 |
| 0.0027578 | 0.137 | 0.0238095 | 2.24 | 0.78 | 0.35 | [SIGLEC14](http://www.ncbi.nlm.nih.gov/entrez/query.fcgi?cmd=search&db=gene&term=SIGLEC14) | sialic acid binding Ig-like lectin 14 | [NM_001098612](http://www.ncbi.nlm.nih.gov/entrez/query.fcgi?db=Nucleotide&term=NM_001098612) | hs\|19q13.41 |
| 0.0066765 | 0.137 | 0.0079365 | 1.88 | 0.65 | 0.35 |  |  |  | hs\|8q24.21 |
| 0.0057454 | 0.137 | 0.0238095 | 2.08 | 0.72 | 0.35 | [lnc-ENGASE-1](http://www.ncbi.nlm.nih.gov/entrez/query.fcgi?cmd=search&db=gene&term=lnc-ENGASE-1) | lnc-ENGASE-1:1 | [DB009474](http://www.ncbi.nlm.nih.gov/entrez/query.fcgi?db=Nucleotide&term=DB009474) | hs\|17q25.3 |
| 0.0083039 | 0.141 | 0.031746 | 1.98 | 0.68 | 0.35 | [lnc-SARM1-2](http://www.ncbi.nlm.nih.gov/entrez/query.fcgi?cmd=search&db=gene&term=lnc-SARM1-2) | lnc-SARM1-2:1 | [BI828099](http://www.ncbi.nlm.nih.gov/entrez/query.fcgi?db=Nucleotide&term=BI828099) | hs\|17q11.2 |
| 0.0055136 | 0.137 | 0.0396825 | 2.41 | 0.83 | 0.35 | [SLC39A8](http://www.ncbi.nlm.nih.gov/entrez/query.fcgi?cmd=search&db=gene&term=SLC39A8) | solute carrier family 39 (zinc transporter), member 8 | [NM_022154](http://www.ncbi.nlm.nih.gov/entrez/query.fcgi?db=Nucleotide&term=NM_022154) | hs\|4q24 |
| 0.002225 | 0.137 | 0.015873 | 2.17 | 0.75 | 0.35 | [XLOC_l2_015410](http://www.ncbi.nlm.nih.gov/entrez/query.fcgi?cmd=search&db=gene&term=XLOC_l2_015410) |  |  | hs\|9q34.3 |
| 0.0079109 | 0.14 | 0.031746 | 2.33 | 0.8 | 0.34 | [lnc-ATG16L2-2](http://www.ncbi.nlm.nih.gov/entrez/query.fcgi?cmd=search&db=gene&term=lnc-ATG16L2-2) | lnc-ATG16L2-2:1 | [AK125753](http://www.ncbi.nlm.nih.gov/entrez/query.fcgi?db=Nucleotide&term=AK125753) | hs\|11q13.4 |
| 0.0014664 | 0.137 | 0.0079365 | 1.51 | 0.52 | 0.34 | [MPV17L](http://www.ncbi.nlm.nih.gov/entrez/query.fcgi?cmd=search&db=gene&term=MPV17L) | MPV17 mitochondrial membrane protein-like | [NM_173803](http://www.ncbi.nlm.nih.gov/entrez/query.fcgi?db=Nucleotide&term=NM_173803) | hs\|16p13.11 |
| 0.0045194 | 0.137 | 0.0238095 | 1.73 | 0.6 | 0.34 | [GATA2-AS1](http://www.ncbi.nlm.nih.gov/entrez/query.fcgi?cmd=search&db=gene&term=GATA2-AS1) | GATA2 antisense RNA 1 | [NR_125398](http://www.ncbi.nlm.nih.gov/entrez/query.fcgi?db=Nucleotide&term=NR_125398) | hs\|3q21.3 |
| 0.002272 | 0.137 | 0.0079365 | 1.78 | 0.61 | 0.34 | [lnc-KLHL36-1](http://www.ncbi.nlm.nih.gov/entrez/query.fcgi?cmd=search&db=gene&term=lnc-KLHL36-1) | lnc-KLHL36-1:1 | [AK125484](http://www.ncbi.nlm.nih.gov/entrez/query.fcgi?db=Nucleotide&term=AK125484) | hs\|16q24.1 |
| 0.0080719 | 0.141 | 0.0238095 | 2.27 | 0.78 | 0.34 | [BACE1](http://www.ncbi.nlm.nih.gov/entrez/query.fcgi?cmd=search&db=gene&term=BACE1) | beta-site APP-cleaving enzyme 1 | [NM_012104](http://www.ncbi.nlm.nih.gov/entrez/query.fcgi?db=Nucleotide&term=NM_012104) | hs\|11q23.3 |
| 0.0008709 | 0.137 | 0.0079365 | 1.86 | 0.64 | 0.34 | [LOC100505625](http://www.ncbi.nlm.nih.gov/entrez/query.fcgi?cmd=search&db=gene&term=LOC100505625) | uncharacterized LOC100505625 | [NR_103771](http://www.ncbi.nlm.nih.gov/entrez/query.fcgi?db=Nucleotide&term=NR_103771) | hs\|5p15.31 |
| 0.007386 | 0.139 | 0.0238095 | 1.99 | 0.68 | 0.34 | [LOC100506127](http://www.ncbi.nlm.nih.gov/entrez/query.fcgi?cmd=search&db=gene&term=LOC100506127) | putative uncharacterized protein FLJ37770-like |  | hs\|11q13.5 |
| 0.0050941 | 0.137 | 0.0396825 | 2.49 | 0.85 | 0.34 | [ACTR3BP5](http://www.ncbi.nlm.nih.gov/entrez/query.fcgi?cmd=search&db=gene&term=ACTR3BP5) | ACTR3B pseudogene 5 | [NR_045000](http://www.ncbi.nlm.nih.gov/entrez/query.fcgi?db=Nucleotide&term=NR_045000) | hs\|10p11.1 |
| 0.0044372 | 0.137 | 0.0238095 | 1.22 | 0.42 | 0.34 | [GPC3](http://www.ncbi.nlm.nih.gov/entrez/query.fcgi?cmd=search&db=gene&term=GPC3) | glypican 3 | [NM_001164617](http://www.ncbi.nlm.nih.gov/entrez/query.fcgi?db=Nucleotide&term=NM_001164617) | hs\|Xq26.2 |
| 0.0025005 | 0.137 | 0.015873 | 1.98 | 0.67 | 0.34 |  |  | [BC036435](http://www.ncbi.nlm.nih.gov/entrez/query.fcgi?db=Nucleotide&term=BC036435) | hs\|1p36.13 |
| 0.0053004 | 0.137 | 0.0238095 | 2.3 | 0.78 | 0.34 | [GYLTL1B](http://www.ncbi.nlm.nih.gov/entrez/query.fcgi?cmd=search&db=gene&term=GYLTL1B) | glycosyltransferase-like 1B | [NM_152312](http://www.ncbi.nlm.nih.gov/entrez/query.fcgi?db=Nucleotide&term=NM_152312) | hs\|11p11.2 |
| 0.0083762 | 0.141 | 0.015873 | 1.68 | 0.57 | 0.34 | [TTN](http://www.ncbi.nlm.nih.gov/entrez/query.fcgi?cmd=search&db=gene&term=TTN) | titin | [NM_133378](http://www.ncbi.nlm.nih.gov/entrez/query.fcgi?db=Nucleotide&term=NM_133378) | hs\|2q31.2 |
| 0.0029543 | 0.137 | 0.0238095 | 2.04 | 0.68 | 0.34 | [lnc-ILK-1](http://www.ncbi.nlm.nih.gov/entrez/query.fcgi?cmd=search&db=gene&term=lnc-ILK-1) | lnc-ILK-1:1 | [BX109589](http://www.ncbi.nlm.nih.gov/entrez/query.fcgi?db=Nucleotide&term=BX109589) | hs\|11p15.4 |
| 0.0008402 | 0.137 | 0.0079365 | 2.28 | 0.77 | 0.34 | [SEL1L](http://www.ncbi.nlm.nih.gov/entrez/query.fcgi?cmd=search&db=gene&term=SEL1L) | sel-1 suppressor of lin-12-like (C. elegans) | [NM_005065](http://www.ncbi.nlm.nih.gov/entrez/query.fcgi?db=Nucleotide&term=NM_005065) | hs\|14q31.1 |
| 0.0007626 | 0.137 | 0.0079365 | 1.53 | 0.51 | 0.33 | [ADHFE1](http://www.ncbi.nlm.nih.gov/entrez/query.fcgi?cmd=search&db=gene&term=ADHFE1) | alcohol dehydrogenase, iron containing, 1 | [NM_144650](http://www.ncbi.nlm.nih.gov/entrez/query.fcgi?db=Nucleotide&term=NM_144650) | hs\|8q13.1 |
| 0.0091646 | 0.143 | 0.031746 | 1.46 | 0.49 | 0.33 | [FLJ37035](http://www.ncbi.nlm.nih.gov/entrez/query.fcgi?cmd=search&db=gene&term=FLJ37035) | uncharacterized LOC399821 |  | hs\|10q26.13 |
| 0.0075147 | 0.139 | 0.031746 | 1.82 | 0.61 | 0.33 | [LOC100506691](http://www.ncbi.nlm.nih.gov/entrez/query.fcgi?cmd=search&db=gene&term=LOC100506691) | uncharacterized LOC100506691 | [XR_424420](http://www.ncbi.nlm.nih.gov/entrez/query.fcgi?db=Nucleotide&term=XR_424420) | hs\|12q24.31 |
| 0.0051413 | 0.137 | 0.0396825 | 2.47 | 0.82 | 0.33 |  |  | [XR_424607](http://www.ncbi.nlm.nih.gov/entrez/query.fcgi?db=Nucleotide&term=XR_424607) | hs\|15q22.32 |
| 0.0097932 | 0.144 | 0.031746 | 2.03 | 0.67 | 0.33 | [lnc-SLC7A11-1](http://www.ncbi.nlm.nih.gov/entrez/query.fcgi?cmd=search&db=gene&term=lnc-SLC7A11-1) | lnc-SLC7A11-1:1 |  | hs\|4q28.3 |
| 0.0027644 | 0.137 | 0.0079365 | 2.28 | 0.76 | 0.33 | [LOC101060085](http://www.ncbi.nlm.nih.gov/entrez/query.fcgi?cmd=search&db=gene&term=LOC101060085) | uncharacterized LOC101060085 | [XM_003960901](http://www.ncbi.nlm.nih.gov/entrez/query.fcgi?db=Nucleotide&term=XM_003960901) | hs\|1p36.13 |
| 0.0043087 | 0.137 | 0.0238095 | 2.51 | 0.82 | 0.33 | [lnc-SNX27-1](http://www.ncbi.nlm.nih.gov/entrez/query.fcgi?cmd=search&db=gene&term=lnc-SNX27-1) | lnc-SNX27-1:1 |  | hs\|1q21.3 |
| 0.0015439 | 0.137 | 0.0079365 | 2.61 | 0.86 | 0.33 | [lnc-PHTF2-1](http://www.ncbi.nlm.nih.gov/entrez/query.fcgi?cmd=search&db=gene&term=lnc-PHTF2-1) | lnc-PHTF2-1:1 |  | hs\|7q21.11 |
| 0.0029135 | 0.137 | 0.0238095 | 1.44 | 0.47 | 0.33 | [SLMAP](http://www.ncbi.nlm.nih.gov/entrez/query.fcgi?cmd=search&db=gene&term=SLMAP) | sarcolemma associated protein | [AK124200](http://www.ncbi.nlm.nih.gov/entrez/query.fcgi?db=Nucleotide&term=AK124200) | hs\|3p14.3 |
| 0.0076889 | 0.14 | 0.0079365 | 2.39 | 0.78 | 0.33 | [XLOC_l2_006780](http://www.ncbi.nlm.nih.gov/entrez/query.fcgi?cmd=search&db=gene&term=XLOC_l2_006780) |  |  | hs\|19q13.33 |
| 0.0060876 | 0.137 | 0.0396825 | 2.61 | 0.85 | 0.32 | [GPR97](http://www.ncbi.nlm.nih.gov/entrez/query.fcgi?cmd=search&db=gene&term=GPR97) | G protein-coupled receptor 97 | [NM_170776](http://www.ncbi.nlm.nih.gov/entrez/query.fcgi?db=Nucleotide&term=NM_170776) | hs\|16q21 |
| 0.0052398 | 0.137 | 0.0238095 | 1.99 | 0.65 | 0.32 | [PLCE1](http://www.ncbi.nlm.nih.gov/entrez/query.fcgi?cmd=search&db=gene&term=PLCE1) | phospholipase C, epsilon 1 | [NM_016341](http://www.ncbi.nlm.nih.gov/entrez/query.fcgi?db=Nucleotide&term=NM_016341) | hs\|10q23.33 |
| 0.0056987 | 0.137 | 0.031746 | 2.03 | 0.66 | 0.32 | [CALML6](http://www.ncbi.nlm.nih.gov/entrez/query.fcgi?cmd=search&db=gene&term=CALML6) | calmodulin-like 6 | [NM_138705](http://www.ncbi.nlm.nih.gov/entrez/query.fcgi?db=Nucleotide&term=NM_138705) | hs\|1p36.33 |
| 0.0064516 | 0.137 | 0.0238095 | 1.89 | 0.61 | 0.32 | [GSG1](http://www.ncbi.nlm.nih.gov/entrez/query.fcgi?cmd=search&db=gene&term=GSG1) | germ cell associated 1 | [NM_001080554](http://www.ncbi.nlm.nih.gov/entrez/query.fcgi?db=Nucleotide&term=NM_001080554) | hs\|12p13.1 |
| 0.0030233 | 0.137 | 0.031746 | 2.59 | 0.84 | 0.32 | [LOC100129129](http://www.ncbi.nlm.nih.gov/entrez/query.fcgi?cmd=search&db=gene&term=LOC100129129) | uncharacterized LOC100129129 |  | hs\|8p23.1 |
| 0.0073203 | 0.139 | 0.015873 | 2.56 | 0.83 | 0.32 | [SLC35E1](http://www.ncbi.nlm.nih.gov/entrez/query.fcgi?cmd=search&db=gene&term=SLC35E1) | solute carrier family 35, member E1 | [NM_024881](http://www.ncbi.nlm.nih.gov/entrez/query.fcgi?db=Nucleotide&term=NM_024881) | hs\|19p13.11 |
| 0.00683 | 0.137 | 0.031746 | 2.2 | 0.71 | 0.32 | [ERC2](http://www.ncbi.nlm.nih.gov/entrez/query.fcgi?cmd=search&db=gene&term=ERC2) | ELKS/RAB6-interacting/CAST family member 2 | [NM_015576](http://www.ncbi.nlm.nih.gov/entrez/query.fcgi?db=Nucleotide&term=NM_015576) | hs\|3p14.3 |
| 0.0076933 | 0.14 | 0.0238095 | 2.15 | 0.69 | 0.32 |  |  |  | hs\|10q26.3 |
| 0.0070601 | 0.138 | 0.031746 | 2.45 | 0.79 | 0.32 | [LOC100288846](http://www.ncbi.nlm.nih.gov/entrez/query.fcgi?cmd=search&db=gene&term=LOC100288846) | uncharacterized LOC100288846 | [NR_038935](http://www.ncbi.nlm.nih.gov/entrez/query.fcgi?db=Nucleotide&term=NR_038935) | hs\|14q21.1 |
| 0.0059415 | 0.137 | 0.0079365 | 1.99 | 0.64 | 0.32 | [TTTY14](http://www.ncbi.nlm.nih.gov/entrez/query.fcgi?cmd=search&db=gene&term=TTTY14) | testis-specific transcript, Y-linked 14 (non-protein coding) |  | hs\|Yq11.222 |
| 0.006263 | 0.137 | 0.0396825 | 2.6 | 0.83 | 0.32 | [FER1L6-AS2](http://www.ncbi.nlm.nih.gov/entrez/query.fcgi?cmd=search&db=gene&term=FER1L6-AS2) | FER1L6 antisense RNA 2 | [BE729513](http://www.ncbi.nlm.nih.gov/entrez/query.fcgi?db=Nucleotide&term=BE729513) | hs\|8q24.13 |
| 0.0089531 | 0.142 | 0.031746 | 1.35 | 0.43 | 0.32 | [TMEM25](http://www.ncbi.nlm.nih.gov/entrez/query.fcgi?cmd=search&db=gene&term=TMEM25) | transmembrane protein 25 | [NM_032780](http://www.ncbi.nlm.nih.gov/entrez/query.fcgi?db=Nucleotide&term=NM_032780) | hs\|11q23.3 |
| 0.0041532 | 0.137 | 0.0079365 | 1.22 | 0.39 | 0.32 | [ALDH1A1](http://www.ncbi.nlm.nih.gov/entrez/query.fcgi?cmd=search&db=gene&term=ALDH1A1) | aldehyde dehydrogenase 1 family, member A1 | [NM_000689](http://www.ncbi.nlm.nih.gov/entrez/query.fcgi?db=Nucleotide&term=NM_000689) | hs\|9q21.13 |
| 0.0017104 | 0.137 | 0.015873 | 2.45 | 0.78 | 0.32 | [FKBP11](http://www.ncbi.nlm.nih.gov/entrez/query.fcgi?cmd=search&db=gene&term=FKBP11) | FK506 binding protein 11, 19 kDa | [NM_016594](http://www.ncbi.nlm.nih.gov/entrez/query.fcgi?db=Nucleotide&term=NM_016594) | hs\|12q13.12 |
| 0.0014174 | 0.137 | 0.0238095 | 2.14 | 0.68 | 0.32 | [LINC00671](http://www.ncbi.nlm.nih.gov/entrez/query.fcgi?cmd=search&db=gene&term=LINC00671) | long intergenic non-protein coding RNA 671 | [NR_027254](http://www.ncbi.nlm.nih.gov/entrez/query.fcgi?db=Nucleotide&term=NR_027254) | hs\|17q21.31 |
| 0.0002246 | 0.137 | 0.0079365 | 1.94 | 0.62 | 0.32 | [lnc-ZNF227-1](http://www.ncbi.nlm.nih.gov/entrez/query.fcgi?cmd=search&db=gene&term=lnc-ZNF227-1) | lnc-ZNF227-1:2 |  | hs\|19q13.31 |
| 0.0011455 | 0.137 | 0.015873 | 2.23 | 0.7 | 0.32 | [lnc-LSG1-1](http://www.ncbi.nlm.nih.gov/entrez/query.fcgi?cmd=search&db=gene&term=lnc-LSG1-1) | lnc-LSG1-1:2 |  | hs\|3q29 |
| 0.0017472 | 0.137 | 0.015873 | 2.44 | 0.77 | 0.32 | [FKBP11](http://www.ncbi.nlm.nih.gov/entrez/query.fcgi?cmd=search&db=gene&term=FKBP11) | FK506 binding protein 11, 19 kDa | [NM_016594](http://www.ncbi.nlm.nih.gov/entrez/query.fcgi?db=Nucleotide&term=NM_016594) | hs\|12q13.12 |
| 0.0016722 | 0.137 | 0.015873 | 2.26 | 0.71 | 0.32 | [LDB3](http://www.ncbi.nlm.nih.gov/entrez/query.fcgi?cmd=search&db=gene&term=LDB3) | LIM domain binding 3 | [NM_001171610](http://www.ncbi.nlm.nih.gov/entrez/query.fcgi?db=Nucleotide&term=NM_001171610) | hs\|10q23.2 |
| 0.0007086 | 0.137 | 0.0079365 | 1.58 | 0.5 | 0.32 | [TNNT3](http://www.ncbi.nlm.nih.gov/entrez/query.fcgi?cmd=search&db=gene&term=TNNT3) | troponin T type 3 (skeletal, fast) | [NM_001297646](http://www.ncbi.nlm.nih.gov/entrez/query.fcgi?db=Nucleotide&term=NM_001297646) | hs\|11p15.5 |
| 0.006165 | 0.137 | 0.0238095 | 2.26 | 0.71 | 0.31 | [CCDC57](http://www.ncbi.nlm.nih.gov/entrez/query.fcgi?cmd=search&db=gene&term=CCDC57) | coiled-coil domain containing 57 | [XM_005256345](http://www.ncbi.nlm.nih.gov/entrez/query.fcgi?db=Nucleotide&term=XM_005256345) | hs\|17q25.3 |
| 0.0006937 | 0.137 | 0.0079365 | 2.26 | 0.71 | 0.31 | [lnc-POU4F2-2](http://www.ncbi.nlm.nih.gov/entrez/query.fcgi?cmd=search&db=gene&term=lnc-POU4F2-2) | lnc-POU4F2-2:1 | [DB062595](http://www.ncbi.nlm.nih.gov/entrez/query.fcgi?db=Nucleotide&term=DB062595) | hs\|4q31.22 |
| 0.0032359 | 0.137 | 0.015873 | 1.65 | 0.52 | 0.31 |  |  | [AL109704](http://www.ncbi.nlm.nih.gov/entrez/query.fcgi?db=Nucleotide&term=AL109704) | hs\|15q26.1 |
| 0.0044714 | 0.137 | 0.0238095 | 2.3 | 0.72 | 0.31 |  |  | [XR_132973](http://www.ncbi.nlm.nih.gov/entrez/query.fcgi?db=Nucleotide&term=XR_132973) | hs\|11q23.3 |
| 0.0047238 | 0.137 | 0.015873 | 1.32 | 0.41 | 0.31 | [C1QTNF9B](http://www.ncbi.nlm.nih.gov/entrez/query.fcgi?cmd=search&db=gene&term=C1QTNF9B) | C1q and tumor necrosis factor related protein 9B | [NM_001007537](http://www.ncbi.nlm.nih.gov/entrez/query.fcgi?db=Nucleotide&term=NM_001007537) | hs\|13q12.12 |
| 0.0045887 | 0.137 | 0.015873 | 1.78 | 0.56 | 0.31 | [LOC100289580](http://www.ncbi.nlm.nih.gov/entrez/query.fcgi?cmd=search&db=gene&term=LOC100289580) | uncharacterized LOC100289580 | [NR_103774](http://www.ncbi.nlm.nih.gov/entrez/query.fcgi?db=Nucleotide&term=NR_103774) | hs\|16q24.3 |
| 0.009952 | 0.144 | 0.031746 | 1.89 | 0.59 | 0.31 | [RABL2A](http://www.ncbi.nlm.nih.gov/entrez/query.fcgi?cmd=search&db=gene&term=RABL2A) | RAB, member of RAS oncogene family-like 2A | [BC040495](http://www.ncbi.nlm.nih.gov/entrez/query.fcgi?db=Nucleotide&term=BC040495) | hs\|2q13 |
| 0.006647 | 0.137 | 0.0238095 | 2.37 | 0.74 | 0.31 |  |  |  | hs\|20p12.1 |
| 0.0030433 | 0.137 | 0.0079365 | 1.75 | 0.54 | 0.31 | [KCNG1](http://www.ncbi.nlm.nih.gov/entrez/query.fcgi?cmd=search&db=gene&term=KCNG1) | potassium channel, voltage gated modifier subfamily G, member 1 | [NM_002237](http://www.ncbi.nlm.nih.gov/entrez/query.fcgi?db=Nucleotide&term=NM_002237) | hs\|20q13.13 |
| 0.0086558 | 0.142 | 0.031746 | 1.7 | 0.53 | 0.31 |  |  | [Z47263](http://www.ncbi.nlm.nih.gov/entrez/query.fcgi?db=Nucleotide&term=Z47263) | hs\|14q32.33 |
| 0.0019988 | 0.137 | 0.0238095 | 2.15 | 0.67 | 0.31 | [lnc-CEBPB-1](http://www.ncbi.nlm.nih.gov/entrez/query.fcgi?cmd=search&db=gene&term=lnc-CEBPB-1) | lnc-CEBPB-1:1 |  | hs\|20q13.13 |
| 0.0094406 | 0.143 | 0.047619 | 2.47 | 0.77 | 0.31 | [LOC101927488](http://www.ncbi.nlm.nih.gov/entrez/query.fcgi?cmd=search&db=gene&term=LOC101927488) | uncharacterized LOC101927488 | [NR_105039](http://www.ncbi.nlm.nih.gov/entrez/query.fcgi?db=Nucleotide&term=NR_105039) | hs\|5q23.2 |
| 0.0004664 | 0.137 | 0.015873 | 1.76 | 0.54 | 0.31 | [CUX2](http://www.ncbi.nlm.nih.gov/entrez/query.fcgi?cmd=search&db=gene&term=CUX2) | cut-like homeobox 2 | [NM_015267](http://www.ncbi.nlm.nih.gov/entrez/query.fcgi?db=Nucleotide&term=NM_015267) | hs\|12q24.11 |
| 0.0026516 | 0.137 | 0.015873 | 2.15 | 0.66 | 0.31 | [PTGDS](http://www.ncbi.nlm.nih.gov/entrez/query.fcgi?cmd=search&db=gene&term=PTGDS) | prostaglandin D2 synthase 21kDa (brain) |  | hs\|9q34.3 |
| 0.0022089 | 0.137 | 0.015873 | 2.33 | 0.72 | 0.31 | [SERPINI1](http://www.ncbi.nlm.nih.gov/entrez/query.fcgi?cmd=search&db=gene&term=SERPINI1) | serpin peptidase inhibitor, clade I (neuroserpin), member 1 | [NM_005025](http://www.ncbi.nlm.nih.gov/entrez/query.fcgi?db=Nucleotide&term=NM_005025) | hs\|3q26.1 |
| 0.0043265 | 0.137 | 0.0238095 | 2.2 | 0.67 | 0.31 | [LOC285095](http://www.ncbi.nlm.nih.gov/entrez/query.fcgi?cmd=search&db=gene&term=LOC285095) | uncharacterized LOC285095 | [XR_430644](http://www.ncbi.nlm.nih.gov/entrez/query.fcgi?db=Nucleotide&term=XR_430644) | hs\|2q37.3 |
| 0.0017226 | 0.137 | 0.0079365 | 1.76 | 0.54 | 0.31 | [DMD](http://www.ncbi.nlm.nih.gov/entrez/query.fcgi?cmd=search&db=gene&term=DMD) | dystrophin | [NM_004010](http://www.ncbi.nlm.nih.gov/entrez/query.fcgi?db=Nucleotide&term=NM_004010) | hs\|Xp21.2 |
| 0.0008389 | 0.137 | 0.0079365 | 2.33 | 0.71 | 0.31 |  |  |  | hs\|4q35.1 |
| 0.0039108 | 0.137 | 0.0238095 | 2.36 | 0.72 | 0.31 |  |  |  | hs\|6p25.3 |
| 0.001538 | 0.137 | 0.0079365 | 1.82 | 0.55 | 0.3 | [REEP1](http://www.ncbi.nlm.nih.gov/entrez/query.fcgi?cmd=search&db=gene&term=REEP1) | receptor accessory protein 1 | [NM_022912](http://www.ncbi.nlm.nih.gov/entrez/query.fcgi?db=Nucleotide&term=NM_022912) | hs\|2p11.2 |
| 0.0029161 | 0.137 | 0.0238095 | 2.16 | 0.66 | 0.3 | [C5orf45](http://www.ncbi.nlm.nih.gov/entrez/query.fcgi?cmd=search&db=gene&term=C5orf45) | chromosome 5 open reading frame 45 | [NM_016175](http://www.ncbi.nlm.nih.gov/entrez/query.fcgi?db=Nucleotide&term=NM_016175) | hs\|5q35.3 |
| 0.0014104 | 0.137 | 0.0238095 | 2.16 | 0.66 | 0.3 | [LINC01568](http://www.ncbi.nlm.nih.gov/entrez/query.fcgi?cmd=search&db=gene&term=LINC01568) | long intergenic non-protein coding RNA 1568 | [NR_038234](http://www.ncbi.nlm.nih.gov/entrez/query.fcgi?db=Nucleotide&term=NR_038234) | hs\|16q22.3 |
| 0.004809 | 0.137 | 0.0238095 | 2.06 | 0.62 | 0.3 | [KRT74](http://www.ncbi.nlm.nih.gov/entrez/query.fcgi?cmd=search&db=gene&term=KRT74) | keratin 74, type II | [NM_175053](http://www.ncbi.nlm.nih.gov/entrez/query.fcgi?db=Nucleotide&term=NM_175053) | hs\|12q13.13 |
| 0.0028992 | 0.137 | 0.015873 | 2.24 | 0.68 | 0.3 | [LINC01163](http://www.ncbi.nlm.nih.gov/entrez/query.fcgi?cmd=search&db=gene&term=LINC01163) | long intergenic non-protein coding RNA 1163 |  | hs\|10q26.2 |
| 0.0053389 | 0.137 | 0.0238095 | 2.06 | 0.62 | 0.3 | [lnc-DUSP1-2](http://www.ncbi.nlm.nih.gov/entrez/query.fcgi?cmd=search&db=gene&term=lnc-DUSP1-2) | lnc-DUSP1-2:1 | [DB065448](http://www.ncbi.nlm.nih.gov/entrez/query.fcgi?db=Nucleotide&term=DB065448) | hs\|5q35.1 |
| 0.0063586 | 0.137 | 0.0238095 | 2.21 | 0.67 | 0.3 | [ZNF592](http://www.ncbi.nlm.nih.gov/entrez/query.fcgi?cmd=search&db=gene&term=ZNF592) | zinc finger protein 592 | [NM_014630](http://www.ncbi.nlm.nih.gov/entrez/query.fcgi?db=Nucleotide&term=NM_014630) | hs\|15q25.3 |
| 0.0098734 | 0.144 | 0.0238095 | 2.13 | 0.64 | 0.3 | [lnc-NFKBIL1-7](http://www.ncbi.nlm.nih.gov/entrez/query.fcgi?cmd=search&db=gene&term=lnc-NFKBIL1-7) | lnc-NFKBIL1-7:1 |  | hs\|6p21.33 |
| 0.0014868 | 0.137 | 0.015873 | 2.66 | 0.8 | 0.3 | [BMP7-AS1](http://www.ncbi.nlm.nih.gov/entrez/query.fcgi?cmd=search&db=gene&term=BMP7-AS1) | BMP7 antisense RNA 1 | [NR_110631](http://www.ncbi.nlm.nih.gov/entrez/query.fcgi?db=Nucleotide&term=NR_110631) | hs\|20q13.31 |
| 0.0042714 | 0.137 | 0.0079365 | 2.51 | 0.75 | 0.3 | [VPS53](http://www.ncbi.nlm.nih.gov/entrez/query.fcgi?cmd=search&db=gene&term=VPS53) | vacuolar protein sorting 53 homolog (S. cerevisiae) | [BC029560](http://www.ncbi.nlm.nih.gov/entrez/query.fcgi?db=Nucleotide&term=BC029560) | hs\|17p13.3 |
| 0.0069291 | 0.138 | 0.0238095 | 2.57 | 0.77 | 0.3 | [LOC100133669](http://www.ncbi.nlm.nih.gov/entrez/query.fcgi?cmd=search&db=gene&term=LOC100133669) | uncharacterized LOC100133669 | [BC007589](http://www.ncbi.nlm.nih.gov/entrez/query.fcgi?db=Nucleotide&term=BC007589) | hs\|8q24.3 |
| 0.0057416 | 0.137 | 0.031746 | 2.49 | 0.74 | 0.3 | [MYCL](http://www.ncbi.nlm.nih.gov/entrez/query.fcgi?cmd=search&db=gene&term=MYCL) | v-myc avian myelocytomatosis viral oncogene lung carcinoma derived homolog | [NM_001033081](http://www.ncbi.nlm.nih.gov/entrez/query.fcgi?db=Nucleotide&term=NM_001033081) | hs\|1p34.2 |
| 0.0078626 | 0.14 | 0.0238095 | 2.32 | 0.69 | 0.3 | [lnc-THSD4-4](http://www.ncbi.nlm.nih.gov/entrez/query.fcgi?cmd=search&db=gene&term=lnc-THSD4-4) | lnc-THSD4-4:1 |  | hs\|15q23 |
| 0.004039 | 0.137 | 0.015873 | 2.77 | 0.82 | 0.29 | [LINC00523](http://www.ncbi.nlm.nih.gov/entrez/query.fcgi?cmd=search&db=gene&term=LINC00523) | long intergenic non-protein coding RNA 523 | [NR_024096](http://www.ncbi.nlm.nih.gov/entrez/query.fcgi?db=Nucleotide&term=NR_024096) | hs\|14q32.2 |
| 0.0046835 | 0.137 | 0.015873 | 2.52 | 0.74 | 0.29 | [LOC100129434](http://www.ncbi.nlm.nih.gov/entrez/query.fcgi?cmd=search&db=gene&term=LOC100129434) | uncharacterized LOC100129434 | [NR_125368](http://www.ncbi.nlm.nih.gov/entrez/query.fcgi?db=Nucleotide&term=NR_125368) | hs\|2p16.1 |
| 0.0037456 | 0.137 | 0.031746 | 2.81 | 0.83 | 0.29 | [LOC220729](http://www.ncbi.nlm.nih.gov/entrez/query.fcgi?cmd=search&db=gene&term=LOC220729) | succinate dehydrogenase complex, subunit A, flavoprotein (Fp) pseudogene | [XR_425697](http://www.ncbi.nlm.nih.gov/entrez/query.fcgi?db=Nucleotide&term=XR_425697) | hs\|3q29 |
| 0.0031763 | 0.137 | 0.015873 | 2.72 | 0.8 | 0.29 | [lnc-ALDH3A2-2](http://www.ncbi.nlm.nih.gov/entrez/query.fcgi?cmd=search&db=gene&term=lnc-ALDH3A2-2) | lnc-ALDH3A2-2:1 |  | hs\|17p11.2 |
| 0.0010153 | 0.137 | 0.0079365 | 2.4 | 0.7 | 0.29 | [SLC22A20](http://www.ncbi.nlm.nih.gov/entrez/query.fcgi?cmd=search&db=gene&term=SLC22A20) | solute carrier family 22, member 20 | [NM_001004326](http://www.ncbi.nlm.nih.gov/entrez/query.fcgi?db=Nucleotide&term=NM_001004326) | hs\|11q13.1 |
| 0.0062569 | 0.137 | 0.031746 | 2.47 | 0.72 | 0.29 | [lnc-C6orf221-1](http://www.ncbi.nlm.nih.gov/entrez/query.fcgi?cmd=search&db=gene&term=lnc-C6orf221-1) | lnc-C6orf221-1:1 |  | hs\|6q13 |
| 0.0050876 | 0.137 | 0.0238095 | 2.21 | 0.65 | 0.29 | [LOC100130540](http://www.ncbi.nlm.nih.gov/entrez/query.fcgi?cmd=search&db=gene&term=LOC100130540) | uncharacterized LOC100130540 | [AK125068](http://www.ncbi.nlm.nih.gov/entrez/query.fcgi?db=Nucleotide&term=AK125068) | hs\|3q21.3 |
| 0.0053572 | 0.137 | 0.015873 | 1.72 | 0.5 | 0.29 | [RADIL](http://www.ncbi.nlm.nih.gov/entrez/query.fcgi?cmd=search&db=gene&term=RADIL) | Ras association and DIL domains | [NM_018059](http://www.ncbi.nlm.nih.gov/entrez/query.fcgi?db=Nucleotide&term=NM_018059) | hs\|7p22.1 |
| 0.0001823 | 0.137 | 0.0079365 | 1.78 | 0.52 | 0.29 | [TTN](http://www.ncbi.nlm.nih.gov/entrez/query.fcgi?cmd=search&db=gene&term=TTN) | titin | [NM_001267550](http://www.ncbi.nlm.nih.gov/entrez/query.fcgi?db=Nucleotide&term=NM_001267550) | hs\|2q31.2 |
| 0.0029075 | 0.137 | 0.015873 | 2.1 | 0.61 | 0.29 | [ZC2HC1B](http://www.ncbi.nlm.nih.gov/entrez/query.fcgi?cmd=search&db=gene&term=ZC2HC1B) | zinc finger, C2HC-type containing 1B | [NM_001013623](http://www.ncbi.nlm.nih.gov/entrez/query.fcgi?db=Nucleotide&term=NM_001013623) | hs\|6q24.2 |
| 0.0076532 | 0.139 | 0.031746 | 2.37 | 0.69 | 0.29 | [KATNB1](http://www.ncbi.nlm.nih.gov/entrez/query.fcgi?cmd=search&db=gene&term=KATNB1) | katanin p80 (WD repeat containing) subunit B 1 | [AB209250](http://www.ncbi.nlm.nih.gov/entrez/query.fcgi?db=Nucleotide&term=AB209250) | hs\|16q21 |
| 0.002495 | 0.137 | 0.015873 | 2.25 | 0.65 | 0.29 | [IRAIN](http://www.ncbi.nlm.nih.gov/entrez/query.fcgi?cmd=search&db=gene&term=IRAIN) | IGF1R antisense imprinted non-protein coding RNA | [NR_126453](http://www.ncbi.nlm.nih.gov/entrez/query.fcgi?db=Nucleotide&term=NR_126453) | hs\|15q26.3 |
| 0.0012939 | 0.137 | 0.0079365 | 1.79 | 0.52 | 0.29 | [SPAG4](http://www.ncbi.nlm.nih.gov/entrez/query.fcgi?cmd=search&db=gene&term=SPAG4) | sperm associated antigen 4 | [NM_003116](http://www.ncbi.nlm.nih.gov/entrez/query.fcgi?db=Nucleotide&term=NM_003116) | hs\|20q11.22 |
| 0.0027304 | 0.137 | 0.0238095 | 2.61 | 0.75 | 0.29 | [lnc-CLEC3A-4](http://www.ncbi.nlm.nih.gov/entrez/query.fcgi?cmd=search&db=gene&term=lnc-CLEC3A-4) | lnc-CLEC3A-4:1 |  | hs\|16q23.1 |
| 0.0098728 | 0.144 | 0.0238095 | 2.12 | 0.61 | 0.29 | [ZNF544](http://www.ncbi.nlm.nih.gov/entrez/query.fcgi?cmd=search&db=gene&term=ZNF544) | zinc finger protein 544 | [NM_014480](http://www.ncbi.nlm.nih.gov/entrez/query.fcgi?db=Nucleotide&term=NM_014480) | hs\|19q13.43 |
| 0.0043223 | 0.137 | 0.0238095 | 2.42 | 0.7 | 0.29 | [ZNF236](http://www.ncbi.nlm.nih.gov/entrez/query.fcgi?cmd=search&db=gene&term=ZNF236) | zinc finger protein 236 | [AF085243](http://www.ncbi.nlm.nih.gov/entrez/query.fcgi?db=Nucleotide&term=AF085243) | hs\|18q23 |
| 0.002673 | 0.137 | 0.015873 | 2.94 | 0.84 | 0.29 | [CDX1](http://www.ncbi.nlm.nih.gov/entrez/query.fcgi?cmd=search&db=gene&term=CDX1) | caudal type homeobox 1 | [NM_001804](http://www.ncbi.nlm.nih.gov/entrez/query.fcgi?db=Nucleotide&term=NM_001804) | hs\|5q32 |
| 0.0031743 | 0.137 | 0.0079365 | 2.79 | 0.8 | 0.29 | [LINC00887](http://www.ncbi.nlm.nih.gov/entrez/query.fcgi?cmd=search&db=gene&term=LINC00887) | long intergenic non-protein coding RNA 887 | [NR_024480](http://www.ncbi.nlm.nih.gov/entrez/query.fcgi?db=Nucleotide&term=NR_024480) | hs\|3q29 |
| 0.006012 | 0.137 | 0.031746 | 2.72 | 0.78 | 0.28 | [KRT78](http://www.ncbi.nlm.nih.gov/entrez/query.fcgi?cmd=search&db=gene&term=KRT78) | keratin 78, type II | [NM_173352](http://www.ncbi.nlm.nih.gov/entrez/query.fcgi?db=Nucleotide&term=NM_173352) | hs\|12q13.13 |
| 0.0016517 | 0.137 | 0.0079365 | 2.22 | 0.63 | 0.28 | [ZDHHC11](http://www.ncbi.nlm.nih.gov/entrez/query.fcgi?cmd=search&db=gene&term=ZDHHC11) | zinc finger, DHHC-type containing 11 | [NM_024786](http://www.ncbi.nlm.nih.gov/entrez/query.fcgi?db=Nucleotide&term=NM_024786) | hs\|5p15.33 |
| 0.0008254 | 0.137 | 0.0079365 | 1.33 | 0.38 | 0.28 | [CLEC3B](http://www.ncbi.nlm.nih.gov/entrez/query.fcgi?cmd=search&db=gene&term=CLEC3B) | C-type lectin domain family 3, member B | [NM_003278](http://www.ncbi.nlm.nih.gov/entrez/query.fcgi?db=Nucleotide&term=NM_003278) | hs\|3p21.31 |
| 0.0035341 | 0.137 | 0.0079365 | 1.88 | 0.53 | 0.28 | [PPM1L](http://www.ncbi.nlm.nih.gov/entrez/query.fcgi?cmd=search&db=gene&term=PPM1L) | protein phosphatase, Mg2+/Mn2+ dependent, 1L | [NM_139245](http://www.ncbi.nlm.nih.gov/entrez/query.fcgi?db=Nucleotide&term=NM_139245) | hs\|3q26.1 |
| 0.002562 | 0.137 | 0.0238095 | 2.79 | 0.79 | 0.28 | [lnc-RP11-736N17.6.1-1](http://www.ncbi.nlm.nih.gov/entrez/query.fcgi?cmd=search&db=gene&term=lnc-RP11-736N17.6.1-1) | lnc-RP11-736N17.6.1-1:1 |  | hs\|14q32.32 |
| 0.0020819 | 0.137 | 0.015873 | 1.7 | 0.48 | 0.28 | [PRIMA1](http://www.ncbi.nlm.nih.gov/entrez/query.fcgi?cmd=search&db=gene&term=PRIMA1) | proline rich membrane anchor 1 | [NM_178013](http://www.ncbi.nlm.nih.gov/entrez/query.fcgi?db=Nucleotide&term=NM_178013) | hs\|14q32.12 |
| 0.0011923 | 0.137 | 0.015873 | 1.85 | 0.52 | 0.28 | [DIAPH2-AS1](http://www.ncbi.nlm.nih.gov/entrez/query.fcgi?cmd=search&db=gene&term=DIAPH2-AS1) | DIAPH2 antisense RNA 1 | [NR_125391](http://www.ncbi.nlm.nih.gov/entrez/query.fcgi?db=Nucleotide&term=NR_125391) | hs\|Xq21.33 |
| 0.0050252 | 0.137 | 0.0238095 | 2.2 | 0.61 | 0.28 |  |  |  | hs\|8q11.23 |
| 0.000644 | 0.137 | 0.0079365 | 2.09 | 0.58 | 0.28 | [DMD](http://www.ncbi.nlm.nih.gov/entrez/query.fcgi?cmd=search&db=gene&term=DMD) | dystrophin | [NM_004021](http://www.ncbi.nlm.nih.gov/entrez/query.fcgi?db=Nucleotide&term=NM_004021) | hs\|Xp21.2 |
| 0.0085079 | 0.141 | 0.031746 | 1.9 | 0.53 | 0.28 | [SHISA5](http://www.ncbi.nlm.nih.gov/entrez/query.fcgi?cmd=search&db=gene&term=SHISA5) | shisa family member 5 | [NM_001272068](http://www.ncbi.nlm.nih.gov/entrez/query.fcgi?db=Nucleotide&term=NM_001272068) | hs\|3p21.31 |
| 0.006262 | 0.137 | 0.0238095 | 2.53 | 0.7 | 0.28 | [lnc-RP1-32I10.10.1-1](http://www.ncbi.nlm.nih.gov/entrez/query.fcgi?cmd=search&db=gene&term=lnc-RP1-32I10.10.1-1) | lnc-RP1-32I10.10.1-1:1 |  | hs\|22q13.31 |
| 0.001373 | 0.137 | 0.0238095 | 2.89 | 0.8 | 0.28 | [LINC01529](http://www.ncbi.nlm.nih.gov/entrez/query.fcgi?cmd=search&db=gene&term=LINC01529) | long intergenic non-protein coding RNA 1529 | [NR_104176](http://www.ncbi.nlm.nih.gov/entrez/query.fcgi?db=Nucleotide&term=NR_104176) | hs\|19q13.12 |
| 0.0052855 | 0.137 | 0.0396825 | 2.78 | 0.77 | 0.28 | [lnc-B4GALNT1-2](http://www.ncbi.nlm.nih.gov/entrez/query.fcgi?cmd=search&db=gene&term=lnc-B4GALNT1-2) | lnc-B4GALNT1-2:2 | [BC073932](http://www.ncbi.nlm.nih.gov/entrez/query.fcgi?db=Nucleotide&term=BC073932) | hs\|12q13.3 |
| 0.004021 | 0.137 | 0.015873 | 2.34 | 0.65 | 0.28 | [lnc-FOXA2-6](http://www.ncbi.nlm.nih.gov/entrez/query.fcgi?cmd=search&db=gene&term=lnc-FOXA2-6) | lnc-FOXA2-6:1 |  | hs\|20p11.21 |
| 0.0088862 | 0.142 | 0.0238095 | 2.56 | 0.7 | 0.27 | [ADAMTSL5](http://www.ncbi.nlm.nih.gov/entrez/query.fcgi?cmd=search&db=gene&term=ADAMTSL5) | ADAMTS-like 5 | [NM_213604](http://www.ncbi.nlm.nih.gov/entrez/query.fcgi?db=Nucleotide&term=NM_213604) | hs\|19p13.3 |
| 0.0018679 | 0.137 | 0.015873 | 1.99 | 0.55 | 0.27 | [GATA2-AS1](http://www.ncbi.nlm.nih.gov/entrez/query.fcgi?cmd=search&db=gene&term=GATA2-AS1) | GATA2 antisense RNA 1 | [NR_125398](http://www.ncbi.nlm.nih.gov/entrez/query.fcgi?db=Nucleotide&term=NR_125398) | hs\|3q21.3 |
| 0.0042767 | 0.137 | 0.0238095 | 2.73 | 0.75 | 0.27 | [TTC31](http://www.ncbi.nlm.nih.gov/entrez/query.fcgi?cmd=search&db=gene&term=TTC31) | tetratricopeptide repeat domain 31 | [NM_022492](http://www.ncbi.nlm.nih.gov/entrez/query.fcgi?db=Nucleotide&term=NM_022492) | hs\|2p13.1 |
| 0.0053058 | 0.137 | 0.0396825 | 2.81 | 0.76 | 0.27 | [lnc-CDC42EP1-1](http://www.ncbi.nlm.nih.gov/entrez/query.fcgi?cmd=search&db=gene&term=lnc-CDC42EP1-1) | lnc-CDC42EP1-1:1 |  | hs\|22q13.1 |
| 0.0040205 | 0.137 | 0.031746 | 2.89 | 0.78 | 0.27 |  |  |  | hs\|16q12.1 |
| 0.0057851 | 0.137 | 0.031746 | 2.79 | 0.75 | 0.27 | [lnc-GMNN-1](http://www.ncbi.nlm.nih.gov/entrez/query.fcgi?cmd=search&db=gene&term=lnc-GMNN-1) | lnc-GMNN-1:1 |  | hs\|6p22.3 |
| 0.0004058 | 0.137 | 0.0079365 | 1.59 | 0.43 | 0.27 |  |  | [KJ149306](http://www.ncbi.nlm.nih.gov/entrez/query.fcgi?db=Nucleotide&term=KJ149306) | hs\|22q11.22 |
| 0.003767 | 0.137 | 0.015873 | 2.77 | 0.74 | 0.27 | [lnc-UCHL3-5](http://www.ncbi.nlm.nih.gov/entrez/query.fcgi?cmd=search&db=gene&term=lnc-UCHL3-5) | lnc-UCHL3-5:1 |  | hs\|13q22.1 |
| 0.0054926 | 0.137 | 0.0238095 | 2.47 | 0.66 | 0.27 | [lnc-IFRD2-2](http://www.ncbi.nlm.nih.gov/entrez/query.fcgi?cmd=search&db=gene&term=lnc-IFRD2-2) | lnc-IFRD2-2:1 | [AK125500](http://www.ncbi.nlm.nih.gov/entrez/query.fcgi?db=Nucleotide&term=AK125500) | hs\|3p21.31 |
| 0.0024376 | 0.137 | 0.015873 | 2.07 | 0.55 | 0.27 | [lnc-C15orf2-2](http://www.ncbi.nlm.nih.gov/entrez/query.fcgi?cmd=search&db=gene&term=lnc-C15orf2-2) | lnc-C15orf2-2:9 |  | hs\|15q11.2 |
| 0.0040095 | 0.137 | 0.031746 | 2.77 | 0.74 | 0.27 | [BACE1](http://www.ncbi.nlm.nih.gov/entrez/query.fcgi?cmd=search&db=gene&term=BACE1) | beta-site APP-cleaving enzyme 1 | [NM_012104](http://www.ncbi.nlm.nih.gov/entrez/query.fcgi?db=Nucleotide&term=NM_012104) | hs\|11q23.3 |
| 0.0026644 | 0.137 | 0.0238095 | 2.87 | 0.76 | 0.27 | [CTBP2](http://www.ncbi.nlm.nih.gov/entrez/query.fcgi?cmd=search&db=gene&term=CTBP2) | C-terminal binding protein 2 | [NM_001290214](http://www.ncbi.nlm.nih.gov/entrez/query.fcgi?db=Nucleotide&term=NM_001290214) | hs\|10q26.13 |
| 0.0031327 | 0.137 | 0.015873 | 1.93 | 0.51 | 0.26 | [lnc-KLF4-4](http://www.ncbi.nlm.nih.gov/entrez/query.fcgi?cmd=search&db=gene&term=lnc-KLF4-4) | lnc-KLF4-4:2 |  | hs\|9q31.2 |
| 0.0064071 | 0.137 | 0.031746 | 2.69 | 0.7 | 0.26 | [DGAT1](http://www.ncbi.nlm.nih.gov/entrez/query.fcgi?cmd=search&db=gene&term=DGAT1) | diacylglycerol O-acyltransferase 1 | [NM_012079](http://www.ncbi.nlm.nih.gov/entrez/query.fcgi?db=Nucleotide&term=NM_012079) | hs\|8q24.3 |
| 0.0016525 | 0.137 | 0.0079365 | 1.71 | 0.44 | 0.26 |  |  |  | hs\|15q11.2 |
| 0.0021698 | 0.137 | 0.0079365 | 2.96 | 0.77 | 0.26 | [CFAP99](http://www.ncbi.nlm.nih.gov/entrez/query.fcgi?cmd=search&db=gene&term=CFAP99) | cilia and flagella associated protein 99 | [NM_001193282](http://www.ncbi.nlm.nih.gov/entrez/query.fcgi?db=Nucleotide&term=NM_001193282) | hs\|4p16.3 |
| 0.0041292 | 0.137 | 0.0238095 | 2.67 | 0.69 | 0.26 | [lnc-CBLB-4](http://www.ncbi.nlm.nih.gov/entrez/query.fcgi?cmd=search&db=gene&term=lnc-CBLB-4) | lnc-CBLB-4:1 |  | hs\|3q13.11 |
| 0.0098346 | 0.144 | 0.031746 | 2.57 | 0.66 | 0.26 | [LINC00029](http://www.ncbi.nlm.nih.gov/entrez/query.fcgi?cmd=search&db=gene&term=LINC00029) | long intergenic non-protein coding RNA 29 | [NR_028295](http://www.ncbi.nlm.nih.gov/entrez/query.fcgi?db=Nucleotide&term=NR_028295) | hs\|20q13.33 |
| 0.0006731 | 0.137 | 0.0079365 | 2.5 | 0.64 | 0.26 | [XLOC_l2_015098](http://www.ncbi.nlm.nih.gov/entrez/query.fcgi?cmd=search&db=gene&term=XLOC_l2_015098) |  |  | hs\|9p24.2 |
| 0.0096829 | 0.144 | 0.047619 | 3.1 | 0.8 | 0.26 | [CXCR3](http://www.ncbi.nlm.nih.gov/entrez/query.fcgi?cmd=search&db=gene&term=CXCR3) | chemokine (C-X-C motif) receptor 3 | [NM_001142797](http://www.ncbi.nlm.nih.gov/entrez/query.fcgi?db=Nucleotide&term=NM_001142797) | hs\|Xq13.1 |
| 0.0029891 | 0.137 | 0.015873 | 2.5 | 0.64 | 0.26 | [LINC01229](http://www.ncbi.nlm.nih.gov/entrez/query.fcgi?cmd=search&db=gene&term=LINC01229) | long intergenic non-protein coding RNA 1229 |  | hs\|16q23.2 |
| 0.0064719 | 0.137 | 0.031746 | 2.42 | 0.62 | 0.26 | [LRRC16B](http://www.ncbi.nlm.nih.gov/entrez/query.fcgi?cmd=search&db=gene&term=LRRC16B) | leucine rich repeat containing 16B | [NM_138360](http://www.ncbi.nlm.nih.gov/entrez/query.fcgi?db=Nucleotide&term=NM_138360) | hs\|14q11.2 |
| 0.0063181 | 0.137 | 0.031746 | 1.61 | 0.41 | 0.26 | [MT1X](http://www.ncbi.nlm.nih.gov/entrez/query.fcgi?cmd=search&db=gene&term=MT1X) | metallothionein 1X | [NM_005952](http://www.ncbi.nlm.nih.gov/entrez/query.fcgi?db=Nucleotide&term=NM_005952) | hs\|16q13 |
| 0.0018813 | 0.137 | 0.015873 | 2.46 | 0.63 | 0.26 | [OR9Q1](http://www.ncbi.nlm.nih.gov/entrez/query.fcgi?cmd=search&db=gene&term=OR9Q1) | olfactory receptor, family 9, subfamily Q, member 1 | [NM_001005212](http://www.ncbi.nlm.nih.gov/entrez/query.fcgi?db=Nucleotide&term=NM_001005212) | hs\|11q12.1 |
| 0.0092286 | 0.143 | 0.031746 | 2.19 | 0.56 | 0.26 | [RNF212](http://www.ncbi.nlm.nih.gov/entrez/query.fcgi?cmd=search&db=gene&term=RNF212) | ring finger protein 212 | [NM_194439](http://www.ncbi.nlm.nih.gov/entrez/query.fcgi?db=Nucleotide&term=NM_194439) | hs\|4p16.3 |
| 0.001952 | 0.137 | 0.015873 | 2.47 | 0.63 | 0.25 | [ITGA2B](http://www.ncbi.nlm.nih.gov/entrez/query.fcgi?cmd=search&db=gene&term=ITGA2B) | integrin, alpha 2b (platelet glycoprotein IIb of IIb/IIIa complex, antigen CD41) | [NM_000419](http://www.ncbi.nlm.nih.gov/entrez/query.fcgi?db=Nucleotide&term=NM_000419) | hs\|17q21.31 |
| 0.0028923 | 0.137 | 0.015873 | 2.28 | 0.58 | 0.25 | [NKX6-2](http://www.ncbi.nlm.nih.gov/entrez/query.fcgi?cmd=search&db=gene&term=NKX6-2) | NK6 homeobox 2 | [NM_177400](http://www.ncbi.nlm.nih.gov/entrez/query.fcgi?db=Nucleotide&term=NM_177400) | hs\|10q26.3 |
| 0.0081064 | 0.141 | 0.031746 | 2.7 | 0.68 | 0.25 | [DNASE1L2](http://www.ncbi.nlm.nih.gov/entrez/query.fcgi?cmd=search&db=gene&term=DNASE1L2) | deoxyribonuclease I-like 2 | [NM_001374](http://www.ncbi.nlm.nih.gov/entrez/query.fcgi?db=Nucleotide&term=NM_001374) | hs\|16p13.3 |
| 0.004629 | 0.137 | 0.0238095 | 2.75 | 0.7 | 0.25 | [SPG20-AS1](http://www.ncbi.nlm.nih.gov/entrez/query.fcgi?cmd=search&db=gene&term=SPG20-AS1) | SPG20 antisense RNA 1 | [NR_045180](http://www.ncbi.nlm.nih.gov/entrez/query.fcgi?db=Nucleotide&term=NR_045180) | hs\|13q13.3 |
| 0.0042246 | 0.137 | 0.0238095 | 2.71 | 0.68 | 0.25 | [PABPC1L2B-AS1](http://www.ncbi.nlm.nih.gov/entrez/query.fcgi?cmd=search&db=gene&term=PABPC1L2B-AS1) | PABPC1L2B antisense RNA 1 (head to head) |  | hs\|Xq13.2 |
| 0.0020951 | 0.137 | 0.015873 | 2.74 | 0.69 | 0.25 | [PRDX4](http://www.ncbi.nlm.nih.gov/entrez/query.fcgi?cmd=search&db=gene&term=PRDX4) | peroxiredoxin 4 | [NM_006406](http://www.ncbi.nlm.nih.gov/entrez/query.fcgi?db=Nucleotide&term=NM_006406) | hs\|Xp22.11 |
| 0.0027966 | 0.137 | 0.0079365 | 1.59 | 0.4 | 0.25 | [KTN1-AS1](http://www.ncbi.nlm.nih.gov/entrez/query.fcgi?cmd=search&db=gene&term=KTN1-AS1) | KTN1 antisense RNA 1 | [NR_027123](http://www.ncbi.nlm.nih.gov/entrez/query.fcgi?db=Nucleotide&term=NR_027123) | hs\|14q22.3 |
| 0.0059478 | 0.137 | 0.0079365 | 1.54 | 0.39 | 0.25 | [NPY1R](http://www.ncbi.nlm.nih.gov/entrez/query.fcgi?cmd=search&db=gene&term=NPY1R) | neuropeptide Y receptor Y1 | [NM_000909](http://www.ncbi.nlm.nih.gov/entrez/query.fcgi?db=Nucleotide&term=NM_000909) | hs\|4q32.2 |
| 0.0075424 | 0.139 | 0.015873 | 2.31 | 0.58 | 0.25 | [PACSIN1](http://www.ncbi.nlm.nih.gov/entrez/query.fcgi?cmd=search&db=gene&term=PACSIN1) | protein kinase C and casein kinase substrate in neurons 1 | [NM_020804](http://www.ncbi.nlm.nih.gov/entrez/query.fcgi?db=Nucleotide&term=NM_020804) | hs\|6p21.31 |
| 0.0049822 | 0.137 | 0.0238095 | 2.35 | 0.59 | 0.25 |  |  | [XR_109346](http://www.ncbi.nlm.nih.gov/entrez/query.fcgi?db=Nucleotide&term=XR_109346) | hs\|1p36.33 |
| 0.0041272 | 0.137 | 0.0238095 | 2.34 | 0.59 | 0.25 | [NUCB2](http://www.ncbi.nlm.nih.gov/entrez/query.fcgi?cmd=search&db=gene&term=NUCB2) | nucleobindin 2 | [NM_005013](http://www.ncbi.nlm.nih.gov/entrez/query.fcgi?db=Nucleotide&term=NM_005013) | hs\|11p15.1 |
| 0.0039343 | 0.137 | 0.0238095 | 2.35 | 0.59 | 0.25 | [XLOC_l2_013808](http://www.ncbi.nlm.nih.gov/entrez/query.fcgi?cmd=search&db=gene&term=XLOC_l2_013808) |  |  | hs\|7p12.3 |
| 0.005162 | 0.137 | 0.031746 | 2.68 | 0.67 | 0.25 | [SHISA4](http://www.ncbi.nlm.nih.gov/entrez/query.fcgi?cmd=search&db=gene&term=SHISA4) | shisa family member 4 | [NM_198149](http://www.ncbi.nlm.nih.gov/entrez/query.fcgi?db=Nucleotide&term=NM_198149) | hs\|1q32.1 |
| 0.0057635 | 0.137 | 0.0238095 | 2.43 | 0.61 | 0.25 |  |  | [AK090448](http://www.ncbi.nlm.nih.gov/entrez/query.fcgi?db=Nucleotide&term=AK090448) | hs\|19q13.2 |
| 0.0081703 | 0.141 | 0.0238095 | 1.48 | 0.37 | 0.25 | [SPTB](http://www.ncbi.nlm.nih.gov/entrez/query.fcgi?cmd=search&db=gene&term=SPTB) | spectrin, beta, erythrocytic | [NM_001024858](http://www.ncbi.nlm.nih.gov/entrez/query.fcgi?db=Nucleotide&term=NM_001024858) | hs\|14q23.3 |
| 0.0083272 | 0.141 | 0.0238095 | 1.48 | 0.36 | 0.25 | [LOC101926956](http://www.ncbi.nlm.nih.gov/entrez/query.fcgi?cmd=search&db=gene&term=LOC101926956) | uncharacterized LOC101926956 | [XR_242403](http://www.ncbi.nlm.nih.gov/entrez/query.fcgi?db=Nucleotide&term=XR_242403) | hs\|8q22.1 |
| 0.0023506 | 0.137 | 0.015873 | 2.66 | 0.66 | 0.25 | [SOX30](http://www.ncbi.nlm.nih.gov/entrez/query.fcgi?cmd=search&db=gene&term=SOX30) | SRY (sex determining region Y)-box 30 | [NM_178424](http://www.ncbi.nlm.nih.gov/entrez/query.fcgi?db=Nucleotide&term=NM_178424) | hs\|5q33.3 |
| 0.0078394 | 0.14 | 0.031746 | 2.35 | 0.58 | 0.25 | [GSG1](http://www.ncbi.nlm.nih.gov/entrez/query.fcgi?cmd=search&db=gene&term=GSG1) | germ cell associated 1 | [NM_031289](http://www.ncbi.nlm.nih.gov/entrez/query.fcgi?db=Nucleotide&term=NM_031289) | hs\|12p13.1 |
| 0.0038685 | 0.137 | 0.031746 | 3.12 | 0.77 | 0.25 | [lnc-CXCR5-1](http://www.ncbi.nlm.nih.gov/entrez/query.fcgi?cmd=search&db=gene&term=lnc-CXCR5-1) | lnc-CXCR5-1:2 |  | hs\|11q23.3 |
| 0.006013 | 0.137 | 0.0238095 | 2.56 | 0.63 | 0.25 | [LOC101930595](http://www.ncbi.nlm.nih.gov/entrez/query.fcgi?cmd=search&db=gene&term=LOC101930595) | uncharacterized LOC101930595 | [XR_247607](http://www.ncbi.nlm.nih.gov/entrez/query.fcgi?db=Nucleotide&term=XR_247607) | hs\|11p15.5 |
| 0.004043 | 0.137 | 0.031746 | 3.39 | 0.83 | 0.25 | [CCL16](http://www.ncbi.nlm.nih.gov/entrez/query.fcgi?cmd=search&db=gene&term=CCL16) | chemokine (C-C motif) ligand 16 | [XM_006725334](http://www.ncbi.nlm.nih.gov/entrez/query.fcgi?db=Nucleotide&term=XM_006725334) | hs\|17q12 |
| 0.0092875 | 0.143 | 0.0396825 | 1.79 | 0.44 | 0.25 | [SLC25A21-AS1](http://www.ncbi.nlm.nih.gov/entrez/query.fcgi?cmd=search&db=gene&term=SLC25A21-AS1) | SLC25A21 antisense RNA 1 | [NR_033240](http://www.ncbi.nlm.nih.gov/entrez/query.fcgi?db=Nucleotide&term=NR_033240) | hs\|14q13.3 |
| 0.005362 | 0.137 | 0.015873 | 3.31 | 0.81 | 0.25 | [ZNF337-AS1](http://www.ncbi.nlm.nih.gov/entrez/query.fcgi?cmd=search&db=gene&term=ZNF337-AS1) | ZNF337 antisense RNA 1 | [NR_126467](http://www.ncbi.nlm.nih.gov/entrez/query.fcgi?db=Nucleotide&term=NR_126467) | hs\|20p11.1 |
| 0.0023257 | 0.137 | 0.0238095 | 2.69 | 0.66 | 0.24 | [LOC101926960](http://www.ncbi.nlm.nih.gov/entrez/query.fcgi?cmd=search&db=gene&term=LOC101926960) | uncharacterized LOC101926960 | [NR_104635](http://www.ncbi.nlm.nih.gov/entrez/query.fcgi?db=Nucleotide&term=NR_104635) | hs\|5p13.1 |
| 0.0066874 | 0.137 | 0.0079365 | 1.55 | 0.38 | 0.24 | [RAPGEF5](http://www.ncbi.nlm.nih.gov/entrez/query.fcgi?cmd=search&db=gene&term=RAPGEF5) | Rap guanine nucleotide exchange factor (GEF) 5 | [AL833195](http://www.ncbi.nlm.nih.gov/entrez/query.fcgi?db=Nucleotide&term=AL833195) | hs\|7p15.3 |
| 0.0017488 | 0.137 | 0.0238095 | 2.52 | 0.62 | 0.24 | [DGCR5](http://www.ncbi.nlm.nih.gov/entrez/query.fcgi?cmd=search&db=gene&term=DGCR5) | DiGeorge syndrome critical region gene 5 (non-protein coding) | [NR_110533](http://www.ncbi.nlm.nih.gov/entrez/query.fcgi?db=Nucleotide&term=NR_110533) | hs\|22q11.21 |
| 0.0076244 | 0.139 | 0.031746 | 2.39 | 0.58 | 0.24 | [lnc-OBFC2A-2](http://www.ncbi.nlm.nih.gov/entrez/query.fcgi?cmd=search&db=gene&term=lnc-OBFC2A-2) | lnc-OBFC2A-2:2 |  | hs\|2q32.3 |
| 0.0016239 | 0.137 | 0.015873 | 3.24 | 0.79 | 0.24 | [KCNH3](http://www.ncbi.nlm.nih.gov/entrez/query.fcgi?cmd=search&db=gene&term=KCNH3) | potassium channel, voltage gated eag related subfamily H, member 3 | [NM_012284](http://www.ncbi.nlm.nih.gov/entrez/query.fcgi?db=Nucleotide&term=NM_012284) | hs\|12q13.12 |
| 0.0058223 | 0.137 | 0.0238095 | 2.45 | 0.59 | 0.24 | [lnc-POLN-1](http://www.ncbi.nlm.nih.gov/entrez/query.fcgi?cmd=search&db=gene&term=lnc-POLN-1) | lnc-POLN-1:3 |  | hs\|4p16.3 |
| 0.0007196 | 0.137 | 0.015873 | 1.87 | 0.45 | 0.24 | [IGSF10](http://www.ncbi.nlm.nih.gov/entrez/query.fcgi?cmd=search&db=gene&term=IGSF10) | immunoglobulin superfamily, member 10 | [NM_178822](http://www.ncbi.nlm.nih.gov/entrez/query.fcgi?db=Nucleotide&term=NM_178822) | hs\|3q25.1 |
| 0.0038607 | 0.137 | 0.0238095 | 3.06 | 0.74 | 0.24 | [LOC101929124](http://www.ncbi.nlm.nih.gov/entrez/query.fcgi?cmd=search&db=gene&term=LOC101929124) | uncharacterized LOC101929124 | [NR_110427](http://www.ncbi.nlm.nih.gov/entrez/query.fcgi?db=Nucleotide&term=NR_110427) | hs\|19p12 |
| 0.0059986 | 0.137 | 0.031746 | 2.85 | 0.69 | 0.24 | [ABAT](http://www.ncbi.nlm.nih.gov/entrez/query.fcgi?cmd=search&db=gene&term=ABAT) | 4-aminobutyrate aminotransferase | [NM_000663](http://www.ncbi.nlm.nih.gov/entrez/query.fcgi?db=Nucleotide&term=NM_000663) | hs\|16p13.2 |
| 0.0051223 | 0.137 | 0.0238095 | 2.67 | 0.64 | 0.24 |  |  |  | hs\|1q21.1 |
| 0.0071771 | 0.138 | 0.031746 | 2.65 | 0.64 | 0.24 | [lnc-RP11-44L9.1.1-8](http://www.ncbi.nlm.nih.gov/entrez/query.fcgi?cmd=search&db=gene&term=lnc-RP11-44L9.1.1-8) | lnc-RP11-44L9.1.1-8:1 |  | hs\|16q22.3 |
| 0.0014849 | 0.137 | 0.015873 | 2.91 | 0.7 | 0.24 | [lnc-POLE2-1](http://www.ncbi.nlm.nih.gov/entrez/query.fcgi?cmd=search&db=gene&term=lnc-POLE2-1) | lnc-POLE2-1:1 |  | hs\|14q21.3 |
| 0.0036195 | 0.137 | 0.0238095 | 3.08 | 0.74 | 0.24 |  |  |  | hs\|15q13.2 |
| 0.0057731 | 0.137 | 0.031746 | 2.96 | 0.71 | 0.24 | [NEUROG3](http://www.ncbi.nlm.nih.gov/entrez/query.fcgi?cmd=search&db=gene&term=NEUROG3) | neurogenin 3 | [NM_020999](http://www.ncbi.nlm.nih.gov/entrez/query.fcgi?db=Nucleotide&term=NM_020999) | hs\|10q22.1 |
| 0.0039 | 0.137 | 0.0238095 | 2.76 | 0.66 | 0.24 |  |  |  | hs\|1q43 |
| 0.0034083 | 0.137 | 0.031746 | 3.12 | 0.74 | 0.24 | [LOC728254](http://www.ncbi.nlm.nih.gov/entrez/query.fcgi?cmd=search&db=gene&term=LOC728254) | uncharacterized LOC728254 | [AB002446](http://www.ncbi.nlm.nih.gov/entrez/query.fcgi?db=Nucleotide&term=AB002446) | hs\|5q22.3 |
| 0.0031633 | 0.137 | 0.0238095 | 2.87 | 0.68 | 0.24 | [lnc-DYNLRB2-1](http://www.ncbi.nlm.nih.gov/entrez/query.fcgi?cmd=search&db=gene&term=lnc-DYNLRB2-1) | lnc-DYNLRB2-1:1 | [BX091138](http://www.ncbi.nlm.nih.gov/entrez/query.fcgi?db=Nucleotide&term=BX091138) | hs\|16q23.2 |
| 0.0050752 | 0.137 | 0.0079365 | 3.03 | 0.72 | 0.24 | [LOC285191](http://www.ncbi.nlm.nih.gov/entrez/query.fcgi?cmd=search&db=gene&term=LOC285191) | uncharacterized LOC285191 | [XR_241442](http://www.ncbi.nlm.nih.gov/entrez/query.fcgi?db=Nucleotide&term=XR_241442) | hs\|2q37.3 |
| 0.0071618 | 0.138 | 0.015873 | 3.1 | 0.73 | 0.24 |  |  | [BC027847](http://www.ncbi.nlm.nih.gov/entrez/query.fcgi?db=Nucleotide&term=BC027847) | hs\|10q26.3 |
| 0.0097791 | 0.144 | 0.0396825 | 1.42 | 0.33 | 0.24 | [MYLK2](http://www.ncbi.nlm.nih.gov/entrez/query.fcgi?cmd=search&db=gene&term=MYLK2) | myosin light chain kinase 2 | [NM_033118](http://www.ncbi.nlm.nih.gov/entrez/query.fcgi?db=Nucleotide&term=NM_033118) | hs\|20q11.21 |
| 0.0034728 | 0.137 | 0.0238095 | 2.97 | 0.7 | 0.24 | [RNASE1](http://www.ncbi.nlm.nih.gov/entrez/query.fcgi?cmd=search&db=gene&term=RNASE1) | ribonuclease, RNase A family, 1 (pancreatic) | [NM_198232](http://www.ncbi.nlm.nih.gov/entrez/query.fcgi?db=Nucleotide&term=NM_198232) | hs\|14q11.2 |
| 0.0032704 | 0.137 | 0.015873 | 2.44 | 0.57 | 0.23 | [RIMS4](http://www.ncbi.nlm.nih.gov/entrez/query.fcgi?cmd=search&db=gene&term=RIMS4) | regulating synaptic membrane exocytosis 4 | [NM_182970](http://www.ncbi.nlm.nih.gov/entrez/query.fcgi?db=Nucleotide&term=NM_182970) | hs\|20q13.12 |
| 0.0019607 | 0.137 | 0.015873 | 2.41 | 0.56 | 0.23 | [lnc-CHRD-1](http://www.ncbi.nlm.nih.gov/entrez/query.fcgi?cmd=search&db=gene&term=lnc-CHRD-1) | lnc-CHRD-1:1 |  | hs\|3q27.1 |
| 0.0049977 | 0.137 | 0.0238095 | 2.4 | 0.56 | 0.23 | [LOC400682](http://www.ncbi.nlm.nih.gov/entrez/query.fcgi?cmd=search&db=gene&term=LOC400682) | zinc finger protein 100-like | [XM_003846486](http://www.ncbi.nlm.nih.gov/entrez/query.fcgi?db=Nucleotide&term=XM_003846486) | hs\|19p12 |
| 0.0025058 | 0.137 | 0.0079365 | 1.63 | 0.38 | 0.23 | [VIPR2](http://www.ncbi.nlm.nih.gov/entrez/query.fcgi?cmd=search&db=gene&term=VIPR2) | vasoactive intestinal peptide receptor 2 | [NM_003382](http://www.ncbi.nlm.nih.gov/entrez/query.fcgi?db=Nucleotide&term=NM_003382) | hs\|7q36.3 |
| 0.0006367 | 0.137 | 0.015873 | 2.68 | 0.63 | 0.23 |  |  |  | hs\|Xp11.4 |
| 0.00719 | 0.138 | 0.031746 | 2.61 | 0.61 | 0.23 | [CLDN6](http://www.ncbi.nlm.nih.gov/entrez/query.fcgi?cmd=search&db=gene&term=CLDN6) | claudin 6 | [NM_021195](http://www.ncbi.nlm.nih.gov/entrez/query.fcgi?db=Nucleotide&term=NM_021195) | hs\|16p13.3 |
| 0.0047235 | 0.137 | 0.031746 | 3.03 | 0.7 | 0.23 | [GCAT](http://www.ncbi.nlm.nih.gov/entrez/query.fcgi?cmd=search&db=gene&term=GCAT) | glycine C-acetyltransferase | [NM_014291](http://www.ncbi.nlm.nih.gov/entrez/query.fcgi?db=Nucleotide&term=NM_014291) | hs\|22q13.1 |
| 0.0046001 | 0.137 | 0.0079365 | 1.45 | 0.34 | 0.23 | [lnc-TMEM18-12](http://www.ncbi.nlm.nih.gov/entrez/query.fcgi?cmd=search&db=gene&term=lnc-TMEM18-12) | lnc-TMEM18-12:1 | [XM_006710125](http://www.ncbi.nlm.nih.gov/entrez/query.fcgi?db=Nucleotide&term=XM_006710125) | hs\|2p25.3 |
| 0.0084308 | 0.141 | 0.0396825 | 2.56 | 0.59 | 0.23 |  |  | [AK098478](http://www.ncbi.nlm.nih.gov/entrez/query.fcgi?db=Nucleotide&term=AK098478) | hs\|9q34.11 |
| 0.0050577 | 0.137 | 0.031746 | 2.59 | 0.6 | 0.23 |  |  | [BX088900](http://www.ncbi.nlm.nih.gov/entrez/query.fcgi?db=Nucleotide&term=BX088900) | hs\|15q14 |
| 0.0064604 | 0.137 | 0.0238095 | 3.29 | 0.75 | 0.23 | [RNF126](http://www.ncbi.nlm.nih.gov/entrez/query.fcgi?cmd=search&db=gene&term=RNF126) | ring finger protein 126 | [NM_194460](http://www.ncbi.nlm.nih.gov/entrez/query.fcgi?db=Nucleotide&term=NM_194460) | hs\|19p13.3 |
| 0.0047615 | 0.137 | 0.015873 | 2.98 | 0.68 | 0.23 | [SGOL1-AS1](http://www.ncbi.nlm.nih.gov/entrez/query.fcgi?cmd=search&db=gene&term=SGOL1-AS1) | SGOL1 antisense RNA 1 | [NR_046723](http://www.ncbi.nlm.nih.gov/entrez/query.fcgi?db=Nucleotide&term=NR_046723) | hs\|3p24.3 |
| 0.0024727 | 0.137 | 0.0238095 | 2.96 | 0.68 | 0.23 | [NETO1](http://www.ncbi.nlm.nih.gov/entrez/query.fcgi?cmd=search&db=gene&term=NETO1) | neuropilin (NRP) and tolloid (TLL)-like 1 | [NM_138966](http://www.ncbi.nlm.nih.gov/entrez/query.fcgi?db=Nucleotide&term=NM_138966) | hs\|18q22.3 |
| 0.0069326 | 0.138 | 0.0238095 | 2.01 | 0.46 | 0.23 | [VLDLR](http://www.ncbi.nlm.nih.gov/entrez/query.fcgi?cmd=search&db=gene&term=VLDLR) | very low density lipoprotein receptor | [NM_003383](http://www.ncbi.nlm.nih.gov/entrez/query.fcgi?db=Nucleotide&term=NM_003383) | hs\|9p24.2 |
| 0.0096232 | 0.144 | 0.0238095 | 1.69 | 0.38 | 0.23 | [TRPM3](http://www.ncbi.nlm.nih.gov/entrez/query.fcgi?cmd=search&db=gene&term=TRPM3) | transient receptor potential cation channel, subfamily M, member 3 | [NM_001007471](http://www.ncbi.nlm.nih.gov/entrez/query.fcgi?db=Nucleotide&term=NM_001007471) | hs\|9q21.12 |
| 0.002331 | 0.137 | 0.015873 | 2.46 | 0.56 | 0.23 | [SARDH](http://www.ncbi.nlm.nih.gov/entrez/query.fcgi?cmd=search&db=gene&term=SARDH) | sarcosine dehydrogenase | [NM_007101](http://www.ncbi.nlm.nih.gov/entrez/query.fcgi?db=Nucleotide&term=NM_007101) | hs\|9q34.2 |
| 0.0026444 | 0.137 | 0.0079365 | 2.22 | 0.5 | 0.23 | [DIRAS1](http://www.ncbi.nlm.nih.gov/entrez/query.fcgi?cmd=search&db=gene&term=DIRAS1) | DIRAS family, GTP-binding RAS-like 1 | [NM_145173](http://www.ncbi.nlm.nih.gov/entrez/query.fcgi?db=Nucleotide&term=NM_145173) | hs\|19p13.3 |
| 0.0008315 | 0.137 | 0.015873 | 2.64 | 0.59 | 0.23 | [lnc-GAPVD1-2](http://www.ncbi.nlm.nih.gov/entrez/query.fcgi?cmd=search&db=gene&term=lnc-GAPVD1-2) | lnc-GAPVD1-2:3 |  | hs\|9q33.3 |
| 0.0070818 | 0.138 | 0.047619 | 3.84 | 0.86 | 0.22 | [HSH2D](http://www.ncbi.nlm.nih.gov/entrez/query.fcgi?cmd=search&db=gene&term=HSH2D) | hematopoietic SH2 domain containing | [NM_001291274](http://www.ncbi.nlm.nih.gov/entrez/query.fcgi?db=Nucleotide&term=NM_001291274) | hs\|19p13.12 |
| 0.0076148 | 0.139 | 0.031746 | 1.61 | 0.36 | 0.22 | [MT1E](http://www.ncbi.nlm.nih.gov/entrez/query.fcgi?cmd=search&db=gene&term=MT1E) | metallothionein 1E | [XM_005255956](http://www.ncbi.nlm.nih.gov/entrez/query.fcgi?db=Nucleotide&term=XM_005255956) | hs\|16q12.2 |
| 0.0018654 | 0.137 | 0.0079365 | 2.75 | 0.62 | 0.22 | [ATP1A2](http://www.ncbi.nlm.nih.gov/entrez/query.fcgi?cmd=search&db=gene&term=ATP1A2) | ATPase, Na+/K+ transporting, alpha 2 polypeptide | [NM_000702](http://www.ncbi.nlm.nih.gov/entrez/query.fcgi?db=Nucleotide&term=NM_000702) | hs\|1q23.2 |
| 0.0075373 | 0.139 | 0.0079365 | 2.11 | 0.47 | 0.22 |  |  |  | hs\|10q26.3 |
| 0.0026712 | 0.137 | 0.015873 | 3.11 | 0.7 | 0.22 | [LOC100130264](http://www.ncbi.nlm.nih.gov/entrez/query.fcgi?cmd=search&db=gene&term=LOC100130264) | uncharacterized LOC100130264 | [NR_024564](http://www.ncbi.nlm.nih.gov/entrez/query.fcgi?db=Nucleotide&term=NR_024564) | hs\|20p11.23 |
| 0.0010531 | 0.137 | 0.0079365 | 1.45 | 0.32 | 0.22 | [VIPR2](http://www.ncbi.nlm.nih.gov/entrez/query.fcgi?cmd=search&db=gene&term=VIPR2) | vasoactive intestinal peptide receptor 2 | [NM_003382](http://www.ncbi.nlm.nih.gov/entrez/query.fcgi?db=Nucleotide&term=NM_003382) | hs\|7q36.3 |
| 0.0039655 | 0.137 | 0.0238095 | 3.37 | 0.75 | 0.22 | [LINC00265](http://www.ncbi.nlm.nih.gov/entrez/query.fcgi?cmd=search&db=gene&term=LINC00265) | long intergenic non-protein coding RNA 265 | [NR_026999](http://www.ncbi.nlm.nih.gov/entrez/query.fcgi?db=Nucleotide&term=NR_026999) | hs\|7p14.1 |
| 0.0092159 | 0.143 | 0.015873 | 3.87 | 0.86 | 0.22 | [lnc-C1orf177-1](http://www.ncbi.nlm.nih.gov/entrez/query.fcgi?cmd=search&db=gene&term=lnc-C1orf177-1) | lnc-C1orf177-1:1 |  | hs\|1p32.3 |
| 0.0019302 | 0.137 | 0.0238095 | 2.43 | 0.54 | 0.22 |  |  |  | hs\|19q13.11 |
| 0.0035393 | 0.137 | 0.0079365 | 1.39 | 0.31 | 0.22 | [LIFR](http://www.ncbi.nlm.nih.gov/entrez/query.fcgi?cmd=search&db=gene&term=LIFR) | leukemia inhibitory factor receptor alpha | [NM_002310](http://www.ncbi.nlm.nih.gov/entrez/query.fcgi?db=Nucleotide&term=NM_002310) | hs\|5p13.1 |
| 0.0045143 | 0.137 | 0.031746 | 2.87 | 0.64 | 0.22 | [SEMA6C](http://www.ncbi.nlm.nih.gov/entrez/query.fcgi?cmd=search&db=gene&term=SEMA6C) | sema domain, transmembrane domain (TM), and cytoplasmic domain, (semaphorin) 6C | [NM_030913](http://www.ncbi.nlm.nih.gov/entrez/query.fcgi?db=Nucleotide&term=NM_030913) | hs\|1q21.3 |
| 0.0018795 | 0.137 | 0.015873 | 2.71 | 0.6 | 0.22 | [LINC00644](http://www.ncbi.nlm.nih.gov/entrez/query.fcgi?cmd=search&db=gene&term=LINC00644) | long intergenic non-protein coding RNA 644 | [NR_104063](http://www.ncbi.nlm.nih.gov/entrez/query.fcgi?db=Nucleotide&term=NR_104063) | hs\|14q23.2 |
| 0.006472 | 0.137 | 0.031746 | 2.9 | 0.64 | 0.22 | [lnc-TLE3-6](http://www.ncbi.nlm.nih.gov/entrez/query.fcgi?cmd=search&db=gene&term=lnc-TLE3-6) | lnc-TLE3-6:2 |  | hs\|15q23 |
| 0.0067526 | 0.137 | 0.0238095 | 1.81 | 0.4 | 0.22 | [PHGDH](http://www.ncbi.nlm.nih.gov/entrez/query.fcgi?cmd=search&db=gene&term=PHGDH) | phosphoglycerate dehydrogenase | [NM_006623](http://www.ncbi.nlm.nih.gov/entrez/query.fcgi?db=Nucleotide&term=NM_006623) | hs\|1p12 |
| 0.0059432 | 0.137 | 0.0238095 | 2.56 | 0.56 | 0.22 | [BRSK2](http://www.ncbi.nlm.nih.gov/entrez/query.fcgi?cmd=search&db=gene&term=BRSK2) | BR serine/threonine kinase 2 | [NM_003957](http://www.ncbi.nlm.nih.gov/entrez/query.fcgi?db=Nucleotide&term=NM_003957) | hs\|11p15.5 |
| 0.0058533 | 0.137 | 0.0238095 | 2.9 | 0.64 | 0.22 | [LOC101929089](http://www.ncbi.nlm.nih.gov/entrez/query.fcgi?cmd=search&db=gene&term=LOC101929089) | uncharacterized LOC101929089 | [NR_120574](http://www.ncbi.nlm.nih.gov/entrez/query.fcgi?db=Nucleotide&term=NR_120574) | hs\|11q23.3 |
| 0.0097177 | 0.144 | 0.0079365 | 2.89 | 0.63 | 0.22 | [PEBP4](http://www.ncbi.nlm.nih.gov/entrez/query.fcgi?cmd=search&db=gene&term=PEBP4) | phosphatidylethanolamine-binding protein 4 | [NM_144962](http://www.ncbi.nlm.nih.gov/entrez/query.fcgi?db=Nucleotide&term=NM_144962) | hs\|8p21.3 |
| 0.0041532 | 0.137 | 0.031746 | 3.12 | 0.68 | 0.22 | [lnc-SPINK9-1](http://www.ncbi.nlm.nih.gov/entrez/query.fcgi?cmd=search&db=gene&term=lnc-SPINK9-1) | lnc-SPINK9-1:1 | [DA214217](http://www.ncbi.nlm.nih.gov/entrez/query.fcgi?db=Nucleotide&term=DA214217) | hs\|5q32 |
| 0.0023807 | 0.137 | 0.015873 | 3.23 | 0.71 | 0.22 | [lnc-MAGEA4-1](http://www.ncbi.nlm.nih.gov/entrez/query.fcgi?cmd=search&db=gene&term=lnc-MAGEA4-1) | lnc-MAGEA4-1:1 | [BE622226](http://www.ncbi.nlm.nih.gov/entrez/query.fcgi?db=Nucleotide&term=BE622226) | hs\|Xq28 |
| 0.0004159 | 0.137 | 0.0079365 | 2.21 | 0.48 | 0.22 | [MT1F](http://www.ncbi.nlm.nih.gov/entrez/query.fcgi?cmd=search&db=gene&term=MT1F) | metallothionein 1F | [NM_005949](http://www.ncbi.nlm.nih.gov/entrez/query.fcgi?db=Nucleotide&term=NM_005949) | hs\|16q12.2 |
| 0.0039144 | 0.137 | 0.015873 | 2.69 | 0.59 | 0.22 | [C5](http://www.ncbi.nlm.nih.gov/entrez/query.fcgi?cmd=search&db=gene&term=C5) | complement component 5 | [NM_001735](http://www.ncbi.nlm.nih.gov/entrez/query.fcgi?db=Nucleotide&term=NM_001735) | hs\|9q33.2 |
| 0.0032882 | 0.137 | 0.0238095 | 2.97 | 0.65 | 0.22 | [lnc-MRM1-1](http://www.ncbi.nlm.nih.gov/entrez/query.fcgi?cmd=search&db=gene&term=lnc-MRM1-1) | lnc-MRM1-1:1 |  | hs\|17q12 |
| 0.0020486 | 0.137 | 0.0079365 | 2.44 | 0.53 | 0.22 | [KCNK3](http://www.ncbi.nlm.nih.gov/entrez/query.fcgi?cmd=search&db=gene&term=KCNK3) | potassium channel, two pore domain subfamily K, member 3 | [NM_002246](http://www.ncbi.nlm.nih.gov/entrez/query.fcgi?db=Nucleotide&term=NM_002246) | hs\|2p23.3 |
| 0.0053169 | 0.137 | 0.0238095 | 2.89 | 0.63 | 0.22 |  |  |  | hs\|4q13.3 |
| 0.0046714 | 0.137 | 0.0238095 | 3.05 | 0.66 | 0.22 | [lnc-RBM11-2](http://www.ncbi.nlm.nih.gov/entrez/query.fcgi?cmd=search&db=gene&term=lnc-RBM11-2) | lnc-RBM11-2:3 | [BC048201](http://www.ncbi.nlm.nih.gov/entrez/query.fcgi?db=Nucleotide&term=BC048201) | hs\|21q11.2 |
| 0.0044083 | 0.137 | 0.0238095 | 2.47 | 0.53 | 0.22 | [lnc-RP11-158I9.5.1-2](http://www.ncbi.nlm.nih.gov/entrez/query.fcgi?cmd=search&db=gene&term=lnc-RP11-158I9.5.1-2) | lnc-RP11-158I9.5.1-2:1 |  | hs\|11q23.3 |
| 0.0007711 | 0.137 | 0.015873 | 2.77 | 0.6 | 0.21 | [BEGAIN](http://www.ncbi.nlm.nih.gov/entrez/query.fcgi?cmd=search&db=gene&term=BEGAIN) | brain-enriched guanylate kinase-associated | [NM_001159531](http://www.ncbi.nlm.nih.gov/entrez/query.fcgi?db=Nucleotide&term=NM_001159531) | hs\|14q32.2 |
| 0.0020188 | 0.137 | 0.015873 | 2.5 | 0.53 | 0.21 |  |  | [DA209974](http://www.ncbi.nlm.nih.gov/entrez/query.fcgi?db=Nucleotide&term=DA209974) | hs\|14q32.2 |
| 0.0047186 | 0.137 | 0.0238095 | 3.57 | 0.76 | 0.21 | [GPHB5](http://www.ncbi.nlm.nih.gov/entrez/query.fcgi?cmd=search&db=gene&term=GPHB5) | glycoprotein hormone beta 5 | [NM_145171](http://www.ncbi.nlm.nih.gov/entrez/query.fcgi?db=Nucleotide&term=NM_145171) | hs\|14q23.2 |
| 0.0049242 | 0.137 | 0.031746 | 3.16 | 0.67 | 0.21 | [LOC200772](http://www.ncbi.nlm.nih.gov/entrez/query.fcgi?cmd=search&db=gene&term=LOC200772) | uncharacterized LOC200772 | [NR_033841](http://www.ncbi.nlm.nih.gov/entrez/query.fcgi?db=Nucleotide&term=NR_033841) | hs\|2q37.3 |
| 0.0008953 | 0.137 | 0.015873 | 3.09 | 0.65 | 0.21 | [SLC43A1](http://www.ncbi.nlm.nih.gov/entrez/query.fcgi?cmd=search&db=gene&term=SLC43A1) | solute carrier family 43 (amino acid system L transporter), member 1 | [NM_003627](http://www.ncbi.nlm.nih.gov/entrez/query.fcgi?db=Nucleotide&term=NM_003627) | hs\|11q12.1 |
| 0.0042605 | 0.137 | 0.015873 | 1.59 | 0.33 | 0.21 | [FGF12](http://www.ncbi.nlm.nih.gov/entrez/query.fcgi?cmd=search&db=gene&term=FGF12) | fibroblast growth factor 12 | [NM_004113](http://www.ncbi.nlm.nih.gov/entrez/query.fcgi?db=Nucleotide&term=NM_004113) | hs\|3q28 |
| 0.0067425 | 0.137 | 0.0396825 | 2.97 | 0.62 | 0.21 | [IGFBP2](http://www.ncbi.nlm.nih.gov/entrez/query.fcgi?cmd=search&db=gene&term=IGFBP2) | insulin-like growth factor binding protein 2, 36kDa | [NM_000597](http://www.ncbi.nlm.nih.gov/entrez/query.fcgi?db=Nucleotide&term=NM_000597) | hs\|2q35 |
| 0.0094283 | 0.143 | 0.031746 | 2.66 | 0.55 | 0.21 | [lnc-TEX261-2](http://www.ncbi.nlm.nih.gov/entrez/query.fcgi?cmd=search&db=gene&term=lnc-TEX261-2) | lnc-TEX261-2:1 |  | hs\|2p13.3 |
| 0.0087772 | 0.142 | 0.031746 | 2.49 | 0.52 | 0.21 | [LOC102723493](http://www.ncbi.nlm.nih.gov/entrez/query.fcgi?cmd=search&db=gene&term=LOC102723493) | uncharacterized LOC102723493 | [XR_424608](http://www.ncbi.nlm.nih.gov/entrez/query.fcgi?db=Nucleotide&term=XR_424608) | hs\|15q22.32 |
| 0.0057352 | 0.137 | 0.0238095 | 3.05 | 0.64 | 0.21 | [ACBD4](http://www.ncbi.nlm.nih.gov/entrez/query.fcgi?cmd=search&db=gene&term=ACBD4) | acyl-CoA binding domain containing 4 | [NM_024722](http://www.ncbi.nlm.nih.gov/entrez/query.fcgi?db=Nucleotide&term=NM_024722) | hs\|17q21.31 |
| 0.0041639 | 0.137 | 0.0238095 | 3.12 | 0.65 | 0.21 | [LOC100507642](http://www.ncbi.nlm.nih.gov/entrez/query.fcgi?cmd=search&db=gene&term=LOC100507642) | uncharacterized LOC100507642 | [NR_108064](http://www.ncbi.nlm.nih.gov/entrez/query.fcgi?db=Nucleotide&term=NR_108064) | hs\|7p22.3 |
| 0.0031613 | 0.137 | 0.015873 | 2.67 | 0.55 | 0.21 |  |  |  | hs\|7q22.1 |
| 0.0095332 | 0.143 | 0.0396825 | 2.41 | 0.5 | 0.21 | [LOC645769](http://www.ncbi.nlm.nih.gov/entrez/query.fcgi?cmd=search&db=gene&term=LOC645769) | uncharacterized LOC645769 | [AK123791](http://www.ncbi.nlm.nih.gov/entrez/query.fcgi?db=Nucleotide&term=AK123791) | hs\|Xp22.2 |
| 4.74E-05 | 0.137 | 0.0079365 | 1.88 | 0.38 | 0.2 | [PAX6](http://www.ncbi.nlm.nih.gov/entrez/query.fcgi?cmd=search&db=gene&term=PAX6) | paired box 6 |  | hs\|11p13 |
| 0.0030604 | 0.137 | 0.015873 | 2.47 | 0.51 | 0.2 |  |  | [BC059953](http://www.ncbi.nlm.nih.gov/entrez/query.fcgi?db=Nucleotide&term=BC059953) | hs\|19p13.3 |
| 0.0002904 | 0.137 | 0.0079365 | 2.75 | 0.56 | 0.2 | [LINC01016](http://www.ncbi.nlm.nih.gov/entrez/query.fcgi?cmd=search&db=gene&term=LINC01016) | long intergenic non-protein coding RNA 1016 | [NR_038989](http://www.ncbi.nlm.nih.gov/entrez/query.fcgi?db=Nucleotide&term=NR_038989) | hs\|6p21.31 |
| 0.0080595 | 0.141 | 0.015873 | 1.94 | 0.39 | 0.2 | [CYP2E1](http://www.ncbi.nlm.nih.gov/entrez/query.fcgi?cmd=search&db=gene&term=CYP2E1) | cytochrome P450, family 2, subfamily E, polypeptide 1 | [NM_000773](http://www.ncbi.nlm.nih.gov/entrez/query.fcgi?db=Nucleotide&term=NM_000773) | hs\|10q26.3 |
| 0.0097976 | 0.144 | 0.0396825 | 3.81 | 0.76 | 0.2 | [OR1L6](http://www.ncbi.nlm.nih.gov/entrez/query.fcgi?cmd=search&db=gene&term=OR1L6) | olfactory receptor, family 1, subfamily L, member 6 | [AB529307](http://www.ncbi.nlm.nih.gov/entrez/query.fcgi?db=Nucleotide&term=AB529307) | hs\|9q33.2 |
| 0.0023796 | 0.137 | 0.015873 | 2.82 | 0.56 | 0.2 | [XLOC_l2_001089](http://www.ncbi.nlm.nih.gov/entrez/query.fcgi?cmd=search&db=gene&term=XLOC_l2_001089) |  |  | hs\|1q21.1 |
| 0.0060638 | 0.137 | 0.0238095 | 2.99 | 0.6 | 0.2 | [ATRNL1](http://www.ncbi.nlm.nih.gov/entrez/query.fcgi?cmd=search&db=gene&term=ATRNL1) | attractin-like 1 | [NM_207303](http://www.ncbi.nlm.nih.gov/entrez/query.fcgi?db=Nucleotide&term=NM_207303) | hs\|10q25.3 |
| 0.003667 | 0.137 | 0.0238095 | 2.2 | 0.44 | 0.2 | [GAMT](http://www.ncbi.nlm.nih.gov/entrez/query.fcgi?cmd=search&db=gene&term=GAMT) | guanidinoacetate N-methyltransferase | [NM_138924](http://www.ncbi.nlm.nih.gov/entrez/query.fcgi?db=Nucleotide&term=NM_138924) | hs\|19p13.3 |
| 0.0024002 | 0.137 | 0.015873 | 2.97 | 0.59 | 0.2 | [MSH6](http://www.ncbi.nlm.nih.gov/entrez/query.fcgi?cmd=search&db=gene&term=MSH6) | mutS homolog 6 |  | hs\|2p16.3 |
| 0.0060328 | 0.137 | 0.0238095 | 2.9 | 0.56 | 0.19 | [ADAM33](http://www.ncbi.nlm.nih.gov/entrez/query.fcgi?cmd=search&db=gene&term=ADAM33) | ADAM metallopeptidase domain 33 | [NM_153202](http://www.ncbi.nlm.nih.gov/entrez/query.fcgi?db=Nucleotide&term=NM_153202) | hs\|20p13 |
| 0.0022455 | 0.137 | 0.0238095 | 2.94 | 0.57 | 0.19 | [NUDT10](http://www.ncbi.nlm.nih.gov/entrez/query.fcgi?cmd=search&db=gene&term=NUDT10) | nudix (nucleoside diphosphate linked moiety X)-type motif 10 | [NM_153183](http://www.ncbi.nlm.nih.gov/entrez/query.fcgi?db=Nucleotide&term=NM_153183) | hs\|Xp11.22 |
| 0.0038118 | 0.137 | 0.0079365 | 1.63 | 0.32 | 0.19 | [AOX1](http://www.ncbi.nlm.nih.gov/entrez/query.fcgi?cmd=search&db=gene&term=AOX1) | aldehyde oxidase 1 | [NM_001159](http://www.ncbi.nlm.nih.gov/entrez/query.fcgi?db=Nucleotide&term=NM_001159) | hs\|2q33.1 |
| 0.0055919 | 0.137 | 0.0238095 | 3.29 | 0.64 | 0.19 | [AHDC1](http://www.ncbi.nlm.nih.gov/entrez/query.fcgi?cmd=search&db=gene&term=AHDC1) | AT hook, DNA binding motif, containing 1 | [NM_001029882](http://www.ncbi.nlm.nih.gov/entrez/query.fcgi?db=Nucleotide&term=NM_001029882) | hs\|1p36.11 |
| 0.0039357 | 0.137 | 0.0238095 | 3.47 | 0.67 | 0.19 |  |  | [XR_428276](http://www.ncbi.nlm.nih.gov/entrez/query.fcgi?db=Nucleotide&term=XR_428276) | hs\|7p11.2 |
| 0.0080721 | 0.141 | 0.0238095 | 2.59 | 0.5 | 0.19 |  |  |  | hs\|6p25.1 |
| 0.002604 | 0.137 | 0.015873 | 2.74 | 0.53 | 0.19 | [CSF2RB](http://www.ncbi.nlm.nih.gov/entrez/query.fcgi?cmd=search&db=gene&term=CSF2RB) | colony stimulating factor 2 receptor, beta, low-affinity (granulocyte-macrophage) | [NM_000395](http://www.ncbi.nlm.nih.gov/entrez/query.fcgi?db=Nucleotide&term=NM_000395) | hs\|22q12.3 |
| 0.0058723 | 0.137 | 0.031746 | 3.78 | 0.73 | 0.19 | [HRH2](http://www.ncbi.nlm.nih.gov/entrez/query.fcgi?cmd=search&db=gene&term=HRH2) | histamine receptor H2 | [NM_022304](http://www.ncbi.nlm.nih.gov/entrez/query.fcgi?db=Nucleotide&term=NM_022304) | hs\|5q35.2 |
| 0.0044468 | 0.137 | 0.015873 | 2.24 | 0.43 | 0.19 |  |  | [BC023973](http://www.ncbi.nlm.nih.gov/entrez/query.fcgi?db=Nucleotide&term=BC023973) | hs\|22q11.22 |
| 0.0012501 | 0.137 | 0.0079365 | 3.9 | 0.75 | 0.19 |  |  |  | hs\|7q36.1 |
| 0.0089791 | 0.142 | 0.0396825 | 1.55 | 0.3 | 0.19 | [lnc-RNF40-1](http://www.ncbi.nlm.nih.gov/entrez/query.fcgi?cmd=search&db=gene&term=lnc-RNF40-1) | lnc-RNF40-1:1 |  | hs\|16p11.2 |
| 0.0027408 | 0.137 | 0.0238095 | 3.45 | 0.66 | 0.19 | [TPST2](http://www.ncbi.nlm.nih.gov/entrez/query.fcgi?cmd=search&db=gene&term=TPST2) | tyrosylprotein sulfotransferase 2 | [NM_001008566](http://www.ncbi.nlm.nih.gov/entrez/query.fcgi?db=Nucleotide&term=NM_001008566) | hs\|22q12.1 |
| 0.0096868 | 0.144 | 0.0396825 | 2.73 | 0.52 | 0.19 | [FUT1](http://www.ncbi.nlm.nih.gov/entrez/query.fcgi?cmd=search&db=gene&term=FUT1) | fucosyltransferase 1 (galactoside 2-alpha-L-fucosyltransferase, H blood group) | [NM_000148](http://www.ncbi.nlm.nih.gov/entrez/query.fcgi?db=Nucleotide&term=NM_000148) | hs\|19q13.33 |
| 0.0057042 | 0.137 | 0.0238095 | 2.76 | 0.52 | 0.19 | [lnc-OXNAD1-1](http://www.ncbi.nlm.nih.gov/entrez/query.fcgi?cmd=search&db=gene&term=lnc-OXNAD1-1) | lnc-OXNAD1-1:2 |  | hs\|3p24.3 |
| 0.0064299 | 0.137 | 0.0238095 | 2.97 | 0.55 | 0.19 | [SLC16A10](http://www.ncbi.nlm.nih.gov/entrez/query.fcgi?cmd=search&db=gene&term=SLC16A10) | solute carrier family 16 (aromatic amino acid transporter), member 10 | [NM_018593](http://www.ncbi.nlm.nih.gov/entrez/query.fcgi?db=Nucleotide&term=NM_018593) | hs\|6q21 |
| 0.0062876 | 0.137 | 0.015873 | 2.28 | 0.42 | 0.19 | [FLJ38379](http://www.ncbi.nlm.nih.gov/entrez/query.fcgi?cmd=search&db=gene&term=FLJ38379) | uncharacterized FLJ38379 | [XR_108456](http://www.ncbi.nlm.nih.gov/entrez/query.fcgi?db=Nucleotide&term=XR_108456) | hs\|2q37.3 |
| 0.0099873 | 0.144 | 0.0396825 | 2.93 | 0.54 | 0.19 | [lnc-GOLGA8J-2](http://www.ncbi.nlm.nih.gov/entrez/query.fcgi?cmd=search&db=gene&term=lnc-GOLGA8J-2) | lnc-GOLGA8J-2:1 | [AK124942](http://www.ncbi.nlm.nih.gov/entrez/query.fcgi?db=Nucleotide&term=AK124942) | hs\|15q13.1 |
| 0.0044399 | 0.137 | 0.0238095 | 3.47 | 0.64 | 0.18 | [ANKRD33](http://www.ncbi.nlm.nih.gov/entrez/query.fcgi?cmd=search&db=gene&term=ANKRD33) | ankyrin repeat domain 33 | [NM_182608](http://www.ncbi.nlm.nih.gov/entrez/query.fcgi?db=Nucleotide&term=NM_182608) | hs\|12q13.13 |
| 0.0021959 | 0.137 | 0.0238095 | 3.53 | 0.65 | 0.18 | [lnc-CHMP7-2](http://www.ncbi.nlm.nih.gov/entrez/query.fcgi?cmd=search&db=gene&term=lnc-CHMP7-2) | lnc-CHMP7-2:1 |  | hs\|8p21.3 |
| 0.001802 | 0.137 | 0.0238095 | 3.49 | 0.63 | 0.18 |  |  | [DB459582](http://www.ncbi.nlm.nih.gov/entrez/query.fcgi?db=Nucleotide&term=DB459582) | hs\|12q13.13 |
| 0.0012218 | 0.137 | 0.015873 | 2.24 | 0.4 | 0.18 | [NRTN](http://www.ncbi.nlm.nih.gov/entrez/query.fcgi?cmd=search&db=gene&term=NRTN) | neurturin | [NM_004558](http://www.ncbi.nlm.nih.gov/entrez/query.fcgi?db=Nucleotide&term=NM_004558) | hs\|19p13.3 |
| 0.0080782 | 0.141 | 0.0238095 | 2.52 | 0.45 | 0.18 | [ZNF385D](http://www.ncbi.nlm.nih.gov/entrez/query.fcgi?cmd=search&db=gene&term=ZNF385D) | zinc finger protein 385D | [BX104753](http://www.ncbi.nlm.nih.gov/entrez/query.fcgi?db=Nucleotide&term=BX104753) | hs\|3p24.3 |
| 0.0023207 | 0.137 | 0.0079365 | 2.06 | 0.37 | 0.18 | [FIGF](http://www.ncbi.nlm.nih.gov/entrez/query.fcgi?cmd=search&db=gene&term=FIGF) | c-fos induced growth factor (vascular endothelial growth factor D) | [NM_004469](http://www.ncbi.nlm.nih.gov/entrez/query.fcgi?db=Nucleotide&term=NM_004469) | hs\|Xp22.2 |
| 0.0038244 | 0.137 | 0.015873 | 3.51 | 0.63 | 0.18 | [lnc-SLC25A6-1](http://www.ncbi.nlm.nih.gov/entrez/query.fcgi?cmd=search&db=gene&term=lnc-SLC25A6-1) | lnc-SLC25A6-1:2 |  | hs\|Xp22.33 |
| 0.0066044 | 0.137 | 0.0238095 | 1.28 | 0.23 | 0.18 | [LMO3](http://www.ncbi.nlm.nih.gov/entrez/query.fcgi?cmd=search&db=gene&term=LMO3) | LIM domain only 3 (rhombotin-like 2) | [NM_018640](http://www.ncbi.nlm.nih.gov/entrez/query.fcgi?db=Nucleotide&term=NM_018640) | hs\|12p12.3 |
| 0.004259 | 0.137 | 0.0238095 | 3.16 | 0.56 | 0.18 | [LOC100507388](http://www.ncbi.nlm.nih.gov/entrez/query.fcgi?cmd=search&db=gene&term=LOC100507388) | uncharacterized LOC100507388 | [NR_121617](http://www.ncbi.nlm.nih.gov/entrez/query.fcgi?db=Nucleotide&term=NR_121617) | hs\|4q13.3 |
| 0.0045054 | 0.137 | 0.0238095 | 4.09 | 0.73 | 0.18 | [TNRC18P1](http://www.ncbi.nlm.nih.gov/entrez/query.fcgi?cmd=search&db=gene&term=TNRC18P1) | TNRC18P1 | [NR_077215](http://www.ncbi.nlm.nih.gov/entrez/query.fcgi?db=Nucleotide&term=NR_077215) | hs\|4q31.21 |
| 0.0042401 | 0.137 | 0.0079365 | 3.16 | 0.56 | 0.18 | [COCH](http://www.ncbi.nlm.nih.gov/entrez/query.fcgi?cmd=search&db=gene&term=COCH) | cochlin | [NM_004086](http://www.ncbi.nlm.nih.gov/entrez/query.fcgi?db=Nucleotide&term=NM_004086) | hs\|14q12 |
| 0.0029111 | 0.137 | 0.0238095 | 3.4 | 0.6 | 0.18 | [LOC101928371](http://www.ncbi.nlm.nih.gov/entrez/query.fcgi?cmd=search&db=gene&term=LOC101928371) | uncharacterized LOC101928371 | [NR_110236](http://www.ncbi.nlm.nih.gov/entrez/query.fcgi?db=Nucleotide&term=NR_110236) | hs\|2p11.2 |
| 0.0037032 | 0.137 | 0.0238095 | 2.69 | 0.47 | 0.18 | [PAIP2B](http://www.ncbi.nlm.nih.gov/entrez/query.fcgi?cmd=search&db=gene&term=PAIP2B) | poly(A) binding protein interacting protein 2B | [NM_020459](http://www.ncbi.nlm.nih.gov/entrez/query.fcgi?db=Nucleotide&term=NM_020459) | hs\|2p13.3 |
| 0.0055327 | 0.137 | 0.0238095 | 3.05 | 0.53 | 0.17 | [lnc-PARN-3](http://www.ncbi.nlm.nih.gov/entrez/query.fcgi?cmd=search&db=gene&term=lnc-PARN-3) | lnc-PARN-3:1 | [AK023827](http://www.ncbi.nlm.nih.gov/entrez/query.fcgi?db=Nucleotide&term=AK023827) | hs\|16p13.11 |
| 0.0081268 | 0.141 | 0.031746 | 3.64 | 0.63 | 0.17 | [STEAP3](http://www.ncbi.nlm.nih.gov/entrez/query.fcgi?cmd=search&db=gene&term=STEAP3) | STEAP family member 3, metalloreductase | [NM_182915](http://www.ncbi.nlm.nih.gov/entrez/query.fcgi?db=Nucleotide&term=NM_182915) | hs\|2q14.2 |
| 0.0026149 | 0.137 | 0.015873 | 3.71 | 0.64 | 0.17 | [LINC01204](http://www.ncbi.nlm.nih.gov/entrez/query.fcgi?cmd=search&db=gene&term=LINC01204) | long intergenic non-protein coding RNA 1204 |  | hs\|Xp11.3 |
| 0.004197 | 0.137 | 0.0238095 | 3.52 | 0.6 | 0.17 | [lnc-NMNAT3-3](http://www.ncbi.nlm.nih.gov/entrez/query.fcgi?cmd=search&db=gene&term=lnc-NMNAT3-3) | lnc-NMNAT3-3:2 |  | hs\|3q23 |
| 0.0041526 | 0.137 | 0.015873 | 2.84 | 0.49 | 0.17 | [VTRNA2-1](http://www.ncbi.nlm.nih.gov/entrez/query.fcgi?cmd=search&db=gene&term=VTRNA2-1) | vault RNA 2-1 | [NR_030583](http://www.ncbi.nlm.nih.gov/entrez/query.fcgi?db=Nucleotide&term=NR_030583) | hs\|5q31.1 |
| 0.0085607 | 0.142 | 0.015873 | 1.21 | 0.21 | 0.17 | [ERBB4](http://www.ncbi.nlm.nih.gov/entrez/query.fcgi?cmd=search&db=gene&term=ERBB4) | erb-b2 receptor tyrosine kinase 4 | [NM_005235](http://www.ncbi.nlm.nih.gov/entrez/query.fcgi?db=Nucleotide&term=NM_005235) | hs\|2q34 |
| 0.00844 | 0.141 | 0.0238095 | 3.04 | 0.52 | 0.17 | [lnc-ABHD12B-3](http://www.ncbi.nlm.nih.gov/entrez/query.fcgi?cmd=search&db=gene&term=lnc-ABHD12B-3) | lnc-ABHD12B-3:1 |  | hs\|14q22.1 |
| 0.0017564 | 0.137 | 0.0238095 | 3.25 | 0.55 | 0.17 | [P2RX1](http://www.ncbi.nlm.nih.gov/entrez/query.fcgi?cmd=search&db=gene&term=P2RX1) | purinergic receptor P2X, ligand gated ion channel, 1 | [NM_002558](http://www.ncbi.nlm.nih.gov/entrez/query.fcgi?db=Nucleotide&term=NM_002558) | hs\|17p13.2 |
| 0.0038596 | 0.137 | 0.0238095 | 3.31 | 0.56 | 0.17 | [SLC22A8](http://www.ncbi.nlm.nih.gov/entrez/query.fcgi?cmd=search&db=gene&term=SLC22A8) | solute carrier family 22 (organic anion transporter), member 8 | [AK123243](http://www.ncbi.nlm.nih.gov/entrez/query.fcgi?db=Nucleotide&term=AK123243) | hs\|11q12.3 |
| 0.0097198 | 0.144 | 0.031746 | 1.7 | 0.29 | 0.17 | [FAM153B](http://www.ncbi.nlm.nih.gov/entrez/query.fcgi?cmd=search&db=gene&term=FAM153B) | family with sequence similarity 153, member B | [NM_001265615](http://www.ncbi.nlm.nih.gov/entrez/query.fcgi?db=Nucleotide&term=NM_001265615) | hs\|5q35.2 |
| 0.004236 | 0.137 | 0.0238095 | 3.52 | 0.6 | 0.17 | [LOC101929151](http://www.ncbi.nlm.nih.gov/entrez/query.fcgi?cmd=search&db=gene&term=LOC101929151) | uncharacterized LOC101929151 | [XR_243177](http://www.ncbi.nlm.nih.gov/entrez/query.fcgi?db=Nucleotide&term=XR_243177) | hs\|15q23 |
| 0.0003743 | 0.137 | 0.0079365 | 3.33 | 0.56 | 0.17 | [IGFN1](http://www.ncbi.nlm.nih.gov/entrez/query.fcgi?cmd=search&db=gene&term=IGFN1) | immunoglobulin-like and fibronectin type III domain containing 1 | [NM_001164586](http://www.ncbi.nlm.nih.gov/entrez/query.fcgi?db=Nucleotide&term=NM_001164586) | hs\|1q32.1 |
| 0.0077248 | 0.14 | 0.0238095 | 3.11 | 0.52 | 0.17 | [PHACTR4](http://www.ncbi.nlm.nih.gov/entrez/query.fcgi?cmd=search&db=gene&term=PHACTR4) | phosphatase and actin regulator 4 | [XM_006710850](http://www.ncbi.nlm.nih.gov/entrez/query.fcgi?db=Nucleotide&term=XM_006710850) | hs\|1p35.3 |
| 0.0048461 | 0.137 | 0.0238095 | 2.81 | 0.47 | 0.17 | [AGBL1](http://www.ncbi.nlm.nih.gov/entrez/query.fcgi?cmd=search&db=gene&term=AGBL1) | ATP/GTP binding protein-like 1 | [NM_152336](http://www.ncbi.nlm.nih.gov/entrez/query.fcgi?db=Nucleotide&term=NM_152336) | hs\|15q25.3 |
| 0.0056192 | 0.137 | 0.015873 | 1.81 | 0.3 | 0.17 | [AOX1](http://www.ncbi.nlm.nih.gov/entrez/query.fcgi?cmd=search&db=gene&term=AOX1) | aldehyde oxidase 1 | [NM_001159](http://www.ncbi.nlm.nih.gov/entrez/query.fcgi?db=Nucleotide&term=NM_001159) | hs\|2q33.1 |
| 0.0020762 | 0.137 | 0.0238095 | 3.91 | 0.65 | 0.17 | [lnc-CCDC113-1](http://www.ncbi.nlm.nih.gov/entrez/query.fcgi?cmd=search&db=gene&term=lnc-CCDC113-1) | lnc-CCDC113-1:1 |  | hs\|16q21 |
| 0.0049334 | 0.137 | 0.0238095 | 3.37 | 0.56 | 0.17 | [lnc-LYPD6-1](http://www.ncbi.nlm.nih.gov/entrez/query.fcgi?cmd=search&db=gene&term=lnc-LYPD6-1) | lnc-LYPD6-1:2 |  | hs\|2q23.2 |
| 0.0084652 | 0.141 | 0.015873 | 2.58 | 0.43 | 0.17 | [LINC01561](http://www.ncbi.nlm.nih.gov/entrez/query.fcgi?cmd=search&db=gene&term=LINC01561) | long intergenic non-protein coding RNA 1561 | [NR_103717](http://www.ncbi.nlm.nih.gov/entrez/query.fcgi?db=Nucleotide&term=NR_103717) | hs\|10q26.12 |
| 0.0067868 | 0.137 | 0.0396825 | 3.48 | 0.57 | 0.16 | [LOC79999](http://www.ncbi.nlm.nih.gov/entrez/query.fcgi?cmd=search&db=gene&term=LOC79999) | uncharacterized LOC79999 | [NM_001291904](http://www.ncbi.nlm.nih.gov/entrez/query.fcgi?db=Nucleotide&term=NM_001291904) | hs\|17p11.2 |
| 0.0023489 | 0.137 | 0.015873 | 4.16 | 0.68 | 0.16 | [SPRNP1](http://www.ncbi.nlm.nih.gov/entrez/query.fcgi?cmd=search&db=gene&term=SPRNP1) | shadow of prion protein homolog (zebrafish) pseudogene 1 | [NR_033789](http://www.ncbi.nlm.nih.gov/entrez/query.fcgi?db=Nucleotide&term=NR_033789) | hs\|10q26.3 |
| 0.0096039 | 0.144 | 0.0238095 | 1.85 | 0.3 | 0.16 | [ANO3](http://www.ncbi.nlm.nih.gov/entrez/query.fcgi?cmd=search&db=gene&term=ANO3) | anoctamin 3 | [NM_031418](http://www.ncbi.nlm.nih.gov/entrez/query.fcgi?db=Nucleotide&term=NM_031418) | hs\|11p14.2 |
| 0.0044577 | 0.137 | 0.0238095 | 3.21 | 0.52 | 0.16 | [CYP11A1](http://www.ncbi.nlm.nih.gov/entrez/query.fcgi?cmd=search&db=gene&term=CYP11A1) | cytochrome P450, family 11, subfamily A, polypeptide 1 | [NM_000781](http://www.ncbi.nlm.nih.gov/entrez/query.fcgi?db=Nucleotide&term=NM_000781) | hs\|15q24.1 |
| 0.0064021 | 0.137 | 0.031746 | 2.83 | 0.46 | 0.16 | [DPEP1](http://www.ncbi.nlm.nih.gov/entrez/query.fcgi?cmd=search&db=gene&term=DPEP1) | dipeptidase 1 (renal) | [NM_004413](http://www.ncbi.nlm.nih.gov/entrez/query.fcgi?db=Nucleotide&term=NM_004413) | hs\|16q24.3 |
| 0.0042108 | 0.137 | 0.0238095 | 2.71 | 0.44 | 0.16 |  |  |  | hs\|19p12 |
| 0.0095427 | 0.143 | 0.0238095 | 1.21 | 0.19 | 0.16 | [LOC101060019](http://www.ncbi.nlm.nih.gov/entrez/query.fcgi?cmd=search&db=gene&term=LOC101060019) | uncharacterized LOC101060019 | [NR_110169](http://www.ncbi.nlm.nih.gov/entrez/query.fcgi?db=Nucleotide&term=NR_110169) | hs\|2p14 |
| 0.0040546 | 0.137 | 0.0238095 | 3.78 | 0.61 | 0.16 | [SEL1L](http://www.ncbi.nlm.nih.gov/entrez/query.fcgi?cmd=search&db=gene&term=SEL1L) | sel-1 suppressor of lin-12-like (C. elegans) | [NM_005065](http://www.ncbi.nlm.nih.gov/entrez/query.fcgi?db=Nucleotide&term=NM_005065) | hs\|14q31.1 |
| 0.0017007 | 0.137 | 0.0238095 | 3.3 | 0.53 | 0.16 | [BACE1](http://www.ncbi.nlm.nih.gov/entrez/query.fcgi?cmd=search&db=gene&term=BACE1) | beta-site APP-cleaving enzyme 1 | [NM_012104](http://www.ncbi.nlm.nih.gov/entrez/query.fcgi?db=Nucleotide&term=NM_012104) | hs\|11q23.3 |
| 0.0027578 | 0.137 | 0.0238095 | 3.03 | 0.48 | 0.16 | [TDRD10](http://www.ncbi.nlm.nih.gov/entrez/query.fcgi?cmd=search&db=gene&term=TDRD10) | tudor domain containing 10 | [NM_182499](http://www.ncbi.nlm.nih.gov/entrez/query.fcgi?db=Nucleotide&term=NM_182499) | hs\|1q21.3 |
| 0.0043824 | 0.137 | 0.031746 | 4.19 | 0.65 | 0.16 | [lnc-SIK1-5](http://www.ncbi.nlm.nih.gov/entrez/query.fcgi?cmd=search&db=gene&term=lnc-SIK1-5) | lnc-SIK1-5:4 |  | hs\|21q22.3 |
| 0.0051114 | 0.137 | 0.0238095 | 3.15 | 0.49 | 0.15 | [TEX26-AS1](http://www.ncbi.nlm.nih.gov/entrez/query.fcgi?cmd=search&db=gene&term=TEX26-AS1) | TEX26 antisense RNA 1 | [NR_038287](http://www.ncbi.nlm.nih.gov/entrez/query.fcgi?db=Nucleotide&term=NR_038287) | hs\|13q12.3 |
| 0.0025088 | 0.137 | 0.015873 | 4.35 | 0.67 | 0.15 | [MIR646HG](http://www.ncbi.nlm.nih.gov/entrez/query.fcgi?cmd=search&db=gene&term=MIR646HG) | MIR646 host gene (non-protein coding) | [AK128288](http://www.ncbi.nlm.nih.gov/entrez/query.fcgi?db=Nucleotide&term=AK128288) | hs\|20q13.33 |
| 0.0037958 | 0.137 | 0.015873 | 2.16 | 0.33 | 0.15 | [FGF14-IT1](http://www.ncbi.nlm.nih.gov/entrez/query.fcgi?cmd=search&db=gene&term=FGF14-IT1) | FGF14 intronic transcript 1 (non-protein coding) | [NR_036486](http://www.ncbi.nlm.nih.gov/entrez/query.fcgi?db=Nucleotide&term=NR_036486) | hs\|13q33.1 |
| 0.0028309 | 0.137 | 0.0238095 | 3.57 | 0.55 | 0.15 | [LOC102724030](http://www.ncbi.nlm.nih.gov/entrez/query.fcgi?cmd=search&db=gene&term=LOC102724030) | uncharacterized LOC102724030 | [XR_424361](http://www.ncbi.nlm.nih.gov/entrez/query.fcgi?db=Nucleotide&term=XR_424361) | hs\|12q13.13 |
| 0.0082078 | 0.141 | 0.0238095 | 3.52 | 0.54 | 0.15 | [SLC17A4](http://www.ncbi.nlm.nih.gov/entrez/query.fcgi?cmd=search&db=gene&term=SLC17A4) | solute carrier family 17, member 4 | [XM_006714946](http://www.ncbi.nlm.nih.gov/entrez/query.fcgi?db=Nucleotide&term=XM_006714946) | hs\|6p22.2 |
| 0.006676 | 0.137 | 0.0238095 | 2.47 | 0.38 | 0.15 | [MYH7](http://www.ncbi.nlm.nih.gov/entrez/query.fcgi?cmd=search&db=gene&term=MYH7) | myosin, heavy chain 7, cardiac muscle, beta | [NM_000257](http://www.ncbi.nlm.nih.gov/entrez/query.fcgi?db=Nucleotide&term=NM_000257) | hs\|14q11.2 |
| 0.0081773 | 0.141 | 0.031746 | 3.97 | 0.6 | 0.15 | [PSAT1](http://www.ncbi.nlm.nih.gov/entrez/query.fcgi?cmd=search&db=gene&term=PSAT1) | phosphoserine aminotransferase 1 | [NM_058179](http://www.ncbi.nlm.nih.gov/entrez/query.fcgi?db=Nucleotide&term=NM_058179) | hs\|9q21.2 |
| 0.0062245 | 0.137 | 0.031746 | 4.51 | 0.68 | 0.15 |  |  |  | hs\|7p21.2 |
| 0.0011378 | 0.137 | 0.015873 | 2.58 | 0.39 | 0.15 | [CADM2](http://www.ncbi.nlm.nih.gov/entrez/query.fcgi?cmd=search&db=gene&term=CADM2) | cell adhesion molecule 2 | [NM_001167674](http://www.ncbi.nlm.nih.gov/entrez/query.fcgi?db=Nucleotide&term=NM_001167674) | hs\|3p12.1 |
| 0.0013808 | 0.137 | 0.0079365 | 2.42 | 0.36 | 0.15 | [HS6ST2](http://www.ncbi.nlm.nih.gov/entrez/query.fcgi?cmd=search&db=gene&term=HS6ST2) | heparan sulfate 6-O-sulfotransferase 2 | [NM_001077188](http://www.ncbi.nlm.nih.gov/entrez/query.fcgi?db=Nucleotide&term=NM_001077188) | hs\|Xq26.2 |
| 0.0074129 | 0.139 | 0.031746 | 2.94 | 0.43 | 0.15 | [lnc-FOS-1](http://www.ncbi.nlm.nih.gov/entrez/query.fcgi?cmd=search&db=gene&term=lnc-FOS-1) | lnc-FOS-1:2 |  | hs\|14q24.3 |
| 0.0050374 | 0.137 | 0.0238095 | 4.46 | 0.66 | 0.15 | [LOC101928785](http://www.ncbi.nlm.nih.gov/entrez/query.fcgi?cmd=search&db=gene&term=LOC101928785) | uncharacterized LOC101928785 | [XR_245261](http://www.ncbi.nlm.nih.gov/entrez/query.fcgi?db=Nucleotide&term=XR_245261) | hs\|4q12 |
| 0.0054101 | 0.137 | 0.0238095 | 2.93 | 0.42 | 0.14 |  |  |  | hs\|12q23.3 |
| 0.0017424 | 0.137 | 0.0238095 | 3.66 | 0.53 | 0.14 | [lnc-ACSBG1-1](http://www.ncbi.nlm.nih.gov/entrez/query.fcgi?cmd=search&db=gene&term=lnc-ACSBG1-1) | lnc-ACSBG1-1:1 | [BX090406](http://www.ncbi.nlm.nih.gov/entrez/query.fcgi?db=Nucleotide&term=BX090406) | hs\|15q25.1 |
| 0.0057006 | 0.137 | 0.0238095 | 4.31 | 0.62 | 0.14 |  |  | [BX096648](http://www.ncbi.nlm.nih.gov/entrez/query.fcgi?db=Nucleotide&term=BX096648) | hs\|14q32.11 |
| 0.0002336 | 0.137 | 0.0079365 | 2.34 | 0.34 | 0.14 | [IGFN1](http://www.ncbi.nlm.nih.gov/entrez/query.fcgi?cmd=search&db=gene&term=IGFN1) | immunoglobulin-like and fibronectin type III domain containing 1 | [NM_001164586](http://www.ncbi.nlm.nih.gov/entrez/query.fcgi?db=Nucleotide&term=NM_001164586) | hs\|1q32.1 |
| 0.004181 | 0.137 | 0.015873 | 3.69 | 0.53 | 0.14 | [H2AFB2](http://www.ncbi.nlm.nih.gov/entrez/query.fcgi?cmd=search&db=gene&term=H2AFB2) | H2A histone family, member B2 | [NM_001017991](http://www.ncbi.nlm.nih.gov/entrez/query.fcgi?db=Nucleotide&term=NM_001017991) | hs\|Xq28 |
| 0.0012502 | 0.137 | 0.0238095 | 3.87 | 0.55 | 0.14 | [ANKRD20A2](http://www.ncbi.nlm.nih.gov/entrez/query.fcgi?cmd=search&db=gene&term=ANKRD20A2) | ankyrin repeat domain 20 family, member A2 | [NM_001012421](http://www.ncbi.nlm.nih.gov/entrez/query.fcgi?db=Nucleotide&term=NM_001012421) | hs\|9p12 |
| 0.0019734 | 0.137 | 0.0079365 | 2.58 | 0.37 | 0.14 | [GDF10](http://www.ncbi.nlm.nih.gov/entrez/query.fcgi?cmd=search&db=gene&term=GDF10) | growth differentiation factor 10 | [NM_004962](http://www.ncbi.nlm.nih.gov/entrez/query.fcgi?db=Nucleotide&term=NM_004962) | hs\|10q11.22 |
| 0.0081826 | 0.141 | 0.0396825 | 2.12 | 0.3 | 0.14 | [ERO1LB](http://www.ncbi.nlm.nih.gov/entrez/query.fcgi?cmd=search&db=gene&term=ERO1LB) | ERO1-like beta (S. cerevisiae) | [NM_019891](http://www.ncbi.nlm.nih.gov/entrez/query.fcgi?db=Nucleotide&term=NM_019891) | hs\|1q42.3 |
| 0.0099823 | 0.144 | 0.0238095 | 3 | 0.42 | 0.14 | [GAFA2](http://www.ncbi.nlm.nih.gov/entrez/query.fcgi?cmd=search&db=gene&term=GAFA2) | FGF-2 activity-associated protein 2 | [AF220234](http://www.ncbi.nlm.nih.gov/entrez/query.fcgi?db=Nucleotide&term=AF220234) | hs\|16q23.2 |
| 0.0069525 | 0.138 | 0.0238095 | 2.18 | 0.3 | 0.14 | [AMHR2](http://www.ncbi.nlm.nih.gov/entrez/query.fcgi?cmd=search&db=gene&term=AMHR2) | anti-Mullerian hormone receptor, type II | [NM_020547](http://www.ncbi.nlm.nih.gov/entrez/query.fcgi?db=Nucleotide&term=NM_020547) | hs\|12q13.13 |
| 0.0028577 | 0.137 | 0.0238095 | 2.86 | 0.39 | 0.14 | [lnc-SLC25A30-1](http://www.ncbi.nlm.nih.gov/entrez/query.fcgi?cmd=search&db=gene&term=lnc-SLC25A30-1) | lnc-SLC25A30-1:1 |  | hs\|13q14.13 |
| 0.0026571 | 0.137 | 0.0238095 | 4.18 | 0.57 | 0.14 | [KRT85](http://www.ncbi.nlm.nih.gov/entrez/query.fcgi?cmd=search&db=gene&term=KRT85) | keratin 85, type II | [NM_002283](http://www.ncbi.nlm.nih.gov/entrez/query.fcgi?db=Nucleotide&term=NM_002283) | hs\|12q13.13 |
| 0.005312 | 0.137 | 0.0238095 | 4.11 | 0.56 | 0.14 | [lnc-KCNJ12-1](http://www.ncbi.nlm.nih.gov/entrez/query.fcgi?cmd=search&db=gene&term=lnc-KCNJ12-1) | lnc-KCNJ12-1:1 |  | hs\|17p11.2 |
| 0.0034909 | 0.137 | 0.0238095 | 3.47 | 0.47 | 0.14 | [lnc-WNT7A-1](http://www.ncbi.nlm.nih.gov/entrez/query.fcgi?cmd=search&db=gene&term=lnc-WNT7A-1) | lnc-WNT7A-1:1 |  | hs\|3p25.1 |
| 0.0011431 | 0.137 | 0.015873 | 3.65 | 0.49 | 0.14 | [CBS](http://www.ncbi.nlm.nih.gov/entrez/query.fcgi?cmd=search&db=gene&term=CBS) | cystathionine-beta-synthase | [NM_000071](http://www.ncbi.nlm.nih.gov/entrez/query.fcgi?db=Nucleotide&term=NM_000071) | hs\|21q22.3 |
| 0.0048696 | 0.137 | 0.0238095 | 5.16 | 0.68 | 0.13 | [LINC00502](http://www.ncbi.nlm.nih.gov/entrez/query.fcgi?cmd=search&db=gene&term=LINC00502) | long intergenic non-protein coding RNA 502 |  | hs\|10q23.31 |
| 0.0055371 | 0.137 | 0.031746 | 3.56 | 0.47 | 0.13 | [CDH18](http://www.ncbi.nlm.nih.gov/entrez/query.fcgi?cmd=search&db=gene&term=CDH18) | cadherin 18, type 2 | [NM_004934](http://www.ncbi.nlm.nih.gov/entrez/query.fcgi?db=Nucleotide&term=NM_004934) | hs\|5p14.3 |
| 0.0071838 | 0.138 | 0.031746 | 3.67 | 0.48 | 0.13 | [LINC01499](http://www.ncbi.nlm.nih.gov/entrez/query.fcgi?cmd=search&db=gene&term=LINC01499) | long intergenic non-protein coding RNA 1499 | [NR_120584](http://www.ncbi.nlm.nih.gov/entrez/query.fcgi?db=Nucleotide&term=NR_120584) | hs\|11p12 |
| 0.0033282 | 0.137 | 0.0238095 | 4.18 | 0.55 | 0.13 |  |  |  | hs\|1q25.3 |
| 0.00655 | 0.137 | 0.0238095 | 2.64 | 0.35 | 0.13 | [MOAP1](http://www.ncbi.nlm.nih.gov/entrez/query.fcgi?cmd=search&db=gene&term=MOAP1) | modulator of apoptosis 1 | [NM_022151](http://www.ncbi.nlm.nih.gov/entrez/query.fcgi?db=Nucleotide&term=NM_022151) | hs\|14q32.12 |
| 0.0023797 | 0.137 | 0.015873 | 1.34 | 0.18 | 0.13 | [PEG10](http://www.ncbi.nlm.nih.gov/entrez/query.fcgi?cmd=search&db=gene&term=PEG10) | paternally expressed 10 | [NM_001040152](http://www.ncbi.nlm.nih.gov/entrez/query.fcgi?db=Nucleotide&term=NM_001040152) | hs\|7q21.3 |
| 0.0045325 | 0.137 | 0.031746 | 5.05 | 0.66 | 0.13 | [KRT9](http://www.ncbi.nlm.nih.gov/entrez/query.fcgi?cmd=search&db=gene&term=KRT9) | keratin 9, type I | [NM_000226](http://www.ncbi.nlm.nih.gov/entrez/query.fcgi?db=Nucleotide&term=NM_000226) | hs\|17q21.2 |
| 0.0073803 | 0.139 | 0.031746 | 3.9 | 0.51 | 0.13 | [FRG2](http://www.ncbi.nlm.nih.gov/entrez/query.fcgi?cmd=search&db=gene&term=FRG2) | FSHD region gene 2 | [NM_001005217](http://www.ncbi.nlm.nih.gov/entrez/query.fcgi?db=Nucleotide&term=NM_001005217) | hs\|4q35.2 |
| 0.0012435 | 0.137 | 0.015873 | 3.52 | 0.44 | 0.13 |  |  |  | hs\|7p12.3 |
| 0.0068982 | 0.138 | 0.0238095 | 4.74 | 0.59 | 0.13 | [GPR161](http://www.ncbi.nlm.nih.gov/entrez/query.fcgi?cmd=search&db=gene&term=GPR161) | G protein-coupled receptor 161 | [NM_001267609](http://www.ncbi.nlm.nih.gov/entrez/query.fcgi?db=Nucleotide&term=NM_001267609) | hs\|1q24.2 |
| 0.0024493 | 0.137 | 0.0238095 | 2.72 | 0.34 | 0.13 | [lnc-NPDC1-1](http://www.ncbi.nlm.nih.gov/entrez/query.fcgi?cmd=search&db=gene&term=lnc-NPDC1-1) | lnc-NPDC1-1:1 |  | hs\|9q34.3 |
| 0.0025104 | 0.137 | 0.015873 | 4.91 | 0.61 | 0.12 | [LOC101929947](http://www.ncbi.nlm.nih.gov/entrez/query.fcgi?cmd=search&db=gene&term=LOC101929947) | uncharacterized LOC101929947 | [XR_251218](http://www.ncbi.nlm.nih.gov/entrez/query.fcgi?db=Nucleotide&term=XR_251218) | hs\|2q31.1 |
| 0.0071476 | 0.138 | 0.015873 | 3.23 | 0.4 | 0.12 | [LOC101929726](http://www.ncbi.nlm.nih.gov/entrez/query.fcgi?cmd=search&db=gene&term=LOC101929726) | uncharacterized LOC101929726 | [XR_242025](http://www.ncbi.nlm.nih.gov/entrez/query.fcgi?db=Nucleotide&term=XR_242025) | hs\|6p21.1 |
| 0.0036744 | 0.137 | 0.0238095 | 3.47 | 0.43 | 0.12 | [PLA2G4C](http://www.ncbi.nlm.nih.gov/entrez/query.fcgi?cmd=search&db=gene&term=PLA2G4C) | phospholipase A2, group IVC (cytosolic, calcium-independent) | [AK124241](http://www.ncbi.nlm.nih.gov/entrez/query.fcgi?db=Nucleotide&term=AK124241) | hs\|19q13.33 |
| 0.0032577 | 0.137 | 0.015873 | 2.25 | 0.28 | 0.12 | [RAB26](http://www.ncbi.nlm.nih.gov/entrez/query.fcgi?cmd=search&db=gene&term=RAB26) | RAB26, member RAS oncogene family | [NM_014353](http://www.ncbi.nlm.nih.gov/entrez/query.fcgi?db=Nucleotide&term=NM_014353) | hs\|16p13.3 |
| 0.002792 | 0.137 | 0.0238095 | 2.87 | 0.35 | 0.12 | [KCNJ5](http://www.ncbi.nlm.nih.gov/entrez/query.fcgi?cmd=search&db=gene&term=KCNJ5) | potassium channel, inwardly rectifying subfamily J, member 5 | [NM_000890](http://www.ncbi.nlm.nih.gov/entrez/query.fcgi?db=Nucleotide&term=NM_000890) | hs\|11q24.3 |
| 0.0083414 | 0.141 | 0.0238095 | 2.47 | 0.3 | 0.12 | [LOC100505940](http://www.ncbi.nlm.nih.gov/entrez/query.fcgi?cmd=search&db=gene&term=LOC100505940) | uncharacterized LOC100505940 | [XR_110477](http://www.ncbi.nlm.nih.gov/entrez/query.fcgi?db=Nucleotide&term=XR_110477) | hs\|1p21.3 |
| 0.0019156 | 0.137 | 0.0079365 | 1.87 | 0.23 | 0.12 | [XLOC_l2_010724](http://www.ncbi.nlm.nih.gov/entrez/query.fcgi?cmd=search&db=gene&term=XLOC_l2_010724) |  |  | hs\|4q21.21 |
| 0.0042409 | 0.137 | 0.0238095 | 2.57 | 0.31 | 0.12 | [LOC285095](http://www.ncbi.nlm.nih.gov/entrez/query.fcgi?cmd=search&db=gene&term=LOC285095) | uncharacterized LOC285095 | [XR_430644](http://www.ncbi.nlm.nih.gov/entrez/query.fcgi?db=Nucleotide&term=XR_430644) | hs\|2q37.3 |
| 0.0067926 | 0.137 | 0.0238095 | 2.91 | 0.35 | 0.12 |  |  |  | hs\|4q35.1 |
| 0.0006317 | 0.137 | 0.015873 | 3.85 | 0.46 | 0.12 | [RBM20](http://www.ncbi.nlm.nih.gov/entrez/query.fcgi?cmd=search&db=gene&term=RBM20) | RNA binding motif protein 20 | [NM_001134363](http://www.ncbi.nlm.nih.gov/entrez/query.fcgi?db=Nucleotide&term=NM_001134363) | hs\|10q25.2 |
| 0.0049718 | 0.137 | 0.0238095 | 3.85 | 0.46 | 0.12 | [lnc-SEL1L-7](http://www.ncbi.nlm.nih.gov/entrez/query.fcgi?cmd=search&db=gene&term=lnc-SEL1L-7) | lnc-SEL1L-7:2 |  | hs\|14q31.2 |
| 0.0040358 | 0.137 | 0.015873 | 5.07 | 0.6 | 0.12 | [SLC22A23](http://www.ncbi.nlm.nih.gov/entrez/query.fcgi?cmd=search&db=gene&term=SLC22A23) | solute carrier family 22, member 23 | [NM_021945](http://www.ncbi.nlm.nih.gov/entrez/query.fcgi?db=Nucleotide&term=NM_021945) | hs\|6p25.2 |
| 0.0089208 | 0.142 | 0.031746 | 2.81 | 0.33 | 0.12 | [LOC101929726](http://www.ncbi.nlm.nih.gov/entrez/query.fcgi?cmd=search&db=gene&term=LOC101929726) | uncharacterized LOC101929726 | [XR_242025](http://www.ncbi.nlm.nih.gov/entrez/query.fcgi?db=Nucleotide&term=XR_242025) | hs\|6p21.1 |
| 0.0036376 | 0.137 | 0.015873 | 2.54 | 0.3 | 0.12 | [XLOC_l2_013963](http://www.ncbi.nlm.nih.gov/entrez/query.fcgi?cmd=search&db=gene&term=XLOC_l2_013963) |  |  | hs\|7q31.1 |
| 0.0021384 | 0.137 | 0.0238095 | 3.82 | 0.45 | 0.12 | [NRG4](http://www.ncbi.nlm.nih.gov/entrez/query.fcgi?cmd=search&db=gene&term=NRG4) | neuregulin 4 | [NM_138573](http://www.ncbi.nlm.nih.gov/entrez/query.fcgi?db=Nucleotide&term=NM_138573) | hs\|15q24.2 |
| 0.0035299 | 0.137 | 0.015873 | 1.77 | 0.2 | 0.12 | [PCP4L1](http://www.ncbi.nlm.nih.gov/entrez/query.fcgi?cmd=search&db=gene&term=PCP4L1) | Purkinje cell protein 4 like 1 | [NM_001102566](http://www.ncbi.nlm.nih.gov/entrez/query.fcgi?db=Nucleotide&term=NM_001102566) | hs\|1q23.3 |
| 0.0065718 | 0.137 | 0.0238095 | 3.4 | 0.39 | 0.11 | [LINC01550](http://www.ncbi.nlm.nih.gov/entrez/query.fcgi?cmd=search&db=gene&term=LINC01550) | long intergenic non-protein coding RNA 1550 | [AK091668](http://www.ncbi.nlm.nih.gov/entrez/query.fcgi?db=Nucleotide&term=AK091668) | hs\|14q32.2 |
| 0.0039624 | 0.137 | 0.0238095 | 2.18 | 0.25 | 0.11 |  |  |  | hs\|8p11.22 |
| 0.0047718 | 0.137 | 0.0238095 | 3.67 | 0.42 | 0.11 | [FAM9B](http://www.ncbi.nlm.nih.gov/entrez/query.fcgi?cmd=search&db=gene&term=FAM9B) | family with sequence similarity 9, member B | [NM_205849](http://www.ncbi.nlm.nih.gov/entrez/query.fcgi?db=Nucleotide&term=NM_205849) | hs\|Xp22.31 |
| 0.0020564 | 0.137 | 0.0238095 | 4.2 | 0.48 | 0.11 | [LINGO1-AS1](http://www.ncbi.nlm.nih.gov/entrez/query.fcgi?cmd=search&db=gene&term=LINGO1-AS1) | LINGO1 antisense RNA 1 | [NR_045123](http://www.ncbi.nlm.nih.gov/entrez/query.fcgi?db=Nucleotide&term=NR_045123) | hs\|15q24.3 |
| 0.006349 | 0.137 | 0.031746 | 3.52 | 0.4 | 0.11 |  |  |  | hs\|18q11.2 |
| 0.0003747 | 0.137 | 0.0079365 | 3.05 | 0.35 | 0.11 |  |  | [AK123491](http://www.ncbi.nlm.nih.gov/entrez/query.fcgi?db=Nucleotide&term=AK123491) | hs\|17q25.3 |
| 0.0037604 | 0.137 | 0.0238095 | 3.12 | 0.35 | 0.11 | [EPO](http://www.ncbi.nlm.nih.gov/entrez/query.fcgi?cmd=search&db=gene&term=EPO) | erythropoietin | [NM_000799](http://www.ncbi.nlm.nih.gov/entrez/query.fcgi?db=Nucleotide&term=NM_000799) | hs\|7q22.1 |
| 0.0059656 | 0.137 | 0.0238095 | 2.26 | 0.26 | 0.11 | [FLJ36777](http://www.ncbi.nlm.nih.gov/entrez/query.fcgi?cmd=search&db=gene&term=FLJ36777) | uncharacterized LOC730971 |  | hs\|4p16.1 |
| 0.0012404 | 0.137 | 0.015873 | 4.12 | 0.46 | 0.11 | [lnc-DUT-1](http://www.ncbi.nlm.nih.gov/entrez/query.fcgi?cmd=search&db=gene&term=lnc-DUT-1) | lnc-DUT-1:1 |  | hs\|15q21.1 |
| 0.0067434 | 0.137 | 0.031746 | 3.76 | 0.42 | 0.11 |  |  |  | hs\|1p36.11 |
| 0.0038685 | 0.137 | 0.031746 | 3.98 | 0.44 | 0.11 | [LINC01214](http://www.ncbi.nlm.nih.gov/entrez/query.fcgi?cmd=search&db=gene&term=LINC01214) | long intergenic non-protein coding RNA 1214 |  | hs\|3q25.1 |
| 0.0057398 | 0.137 | 0.0238095 | 4.11 | 0.46 | 0.11 | [lnc-F8A2-1](http://www.ncbi.nlm.nih.gov/entrez/query.fcgi?cmd=search&db=gene&term=lnc-F8A2-1) | lnc-F8A2-1:1 | [L23866](http://www.ncbi.nlm.nih.gov/entrez/query.fcgi?db=Nucleotide&term=L23866) | hs\|Xq28 |
| 0.0062494 | 0.137 | 0.0238095 | 3.02 | 0.33 | 0.11 | [IGSF3](http://www.ncbi.nlm.nih.gov/entrez/query.fcgi?cmd=search&db=gene&term=IGSF3) | immunoglobulin superfamily, member 3 | [NM_001007237](http://www.ncbi.nlm.nih.gov/entrez/query.fcgi?db=Nucleotide&term=NM_001007237) | hs\|1p13.1 |
| 0.0027463 | 0.137 | 0.0238095 | 2.63 | 0.29 | 0.11 | [CCDC110](http://www.ncbi.nlm.nih.gov/entrez/query.fcgi?cmd=search&db=gene&term=CCDC110) | coiled-coil domain containing 110 | [NM_152775](http://www.ncbi.nlm.nih.gov/entrez/query.fcgi?db=Nucleotide&term=NM_152775) | hs\|4q35.1 |
| 0.001757 | 0.137 | 0.0238095 | 3.2 | 0.35 | 0.11 | [LOC100507600](http://www.ncbi.nlm.nih.gov/entrez/query.fcgi?cmd=search&db=gene&term=LOC100507600) | uncharacterized LOC100507600 | [NR_045486](http://www.ncbi.nlm.nih.gov/entrez/query.fcgi?db=Nucleotide&term=NR_045486) | hs\|2q21.3 |
| 0.0056545 | 0.137 | 0.0238095 | 2.31 | 0.25 | 0.11 | [lnc-HMHB1-3](http://www.ncbi.nlm.nih.gov/entrez/query.fcgi?cmd=search&db=gene&term=lnc-HMHB1-3) | lnc-HMHB1-3:1 | [BX111358](http://www.ncbi.nlm.nih.gov/entrez/query.fcgi?db=Nucleotide&term=BX111358) | hs\|5q31.3 |
| 0.0079473 | 0.14 | 0.0238095 | 3.08 | 0.33 | 0.11 | [LOC101928449](http://www.ncbi.nlm.nih.gov/entrez/query.fcgi?cmd=search&db=gene&term=LOC101928449) | uncharacterized LOC101928449 | [NR_110089](http://www.ncbi.nlm.nih.gov/entrez/query.fcgi?db=Nucleotide&term=NR_110089) | hs\|12q21.31 |
| 0.0087853 | 0.142 | 0.031746 | 3.35 | 0.36 | 0.11 | [PTCHD3](http://www.ncbi.nlm.nih.gov/entrez/query.fcgi?cmd=search&db=gene&term=PTCHD3) | patched domain containing 3 | [NM_001034842](http://www.ncbi.nlm.nih.gov/entrez/query.fcgi?db=Nucleotide&term=NM_001034842) | hs\|10p12.1 |
| 0.0071163 | 0.138 | 0.0238095 | 2.42 | 0.26 | 0.11 |  |  |  |  |
| 0.0068573 | 0.137 | 0.0238095 | 3.68 | 0.39 | 0.11 | [ICMT](http://www.ncbi.nlm.nih.gov/entrez/query.fcgi?cmd=search&db=gene&term=ICMT) | isoprenylcysteine carboxyl methyltransferase | [BC017037](http://www.ncbi.nlm.nih.gov/entrez/query.fcgi?db=Nucleotide&term=BC017037) | hs\|1p36.31 |
| 0.0035339 | 0.137 | 0.0238095 | 4.77 | 0.5 | 0.11 | [MMP17](http://www.ncbi.nlm.nih.gov/entrez/query.fcgi?cmd=search&db=gene&term=MMP17) | matrix metallopeptidase 17 (membrane-inserted) | [NM_016155](http://www.ncbi.nlm.nih.gov/entrez/query.fcgi?db=Nucleotide&term=NM_016155) | hs\|12q24.33 |
| 0.0032081 | 0.137 | 0.015873 | 6.32 | 0.66 | 0.1 | [FAM167A-AS1](http://www.ncbi.nlm.nih.gov/entrez/query.fcgi?cmd=search&db=gene&term=FAM167A-AS1) | FAM167A antisense RNA 1 | [NR_026814](http://www.ncbi.nlm.nih.gov/entrez/query.fcgi?db=Nucleotide&term=NR_026814) | hs\|8p23.1 |
| 0.0040452 | 0.137 | 0.0079365 | 1.97 | 0.21 | 0.1 | [RERGL](http://www.ncbi.nlm.nih.gov/entrez/query.fcgi?cmd=search&db=gene&term=RERGL) | RERG/RAS-like | [NM_024730](http://www.ncbi.nlm.nih.gov/entrez/query.fcgi?db=Nucleotide&term=NM_024730) | hs\|12p12.3 |
| 0.0083791 | 0.141 | 0.031746 | 2.47 | 0.26 | 0.1 | [GATM](http://www.ncbi.nlm.nih.gov/entrez/query.fcgi?cmd=search&db=gene&term=GATM) | glycine amidinotransferase (L-arginine:glycine amidinotransferase) | [NM_001482](http://www.ncbi.nlm.nih.gov/entrez/query.fcgi?db=Nucleotide&term=NM_001482) | hs\|15q21.1 |
| 0.0094256 | 0.143 | 0.031746 | 3.92 | 0.4 | 0.1 |  |  |  | hs\|21q21.3 |
| 0.0039366 | 0.137 | 0.0238095 | 3.33 | 0.34 | 0.1 | [LINC01436](http://www.ncbi.nlm.nih.gov/entrez/query.fcgi?cmd=search&db=gene&term=LINC01436) | long intergenic non-protein coding RNA 1436 |  | hs\|21q22.12 |
| 0.0074099 | 0.139 | 0.0238095 | 2.07 | 0.21 | 0.1 | [PLK5](http://www.ncbi.nlm.nih.gov/entrez/query.fcgi?cmd=search&db=gene&term=PLK5) | polo-like kinase 5 | [NM_001243079](http://www.ncbi.nlm.nih.gov/entrez/query.fcgi?db=Nucleotide&term=NM_001243079) | hs\|19p13.3 |
| 0.0039627 | 0.137 | 0.0238095 | 5.14 | 0.51 | 0.1 |  |  |  | hs\|6q25.3 |
| 0.0047541 | 0.137 | 0.0238095 | 4.29 | 0.42 | 0.1 |  |  |  | hs\|2q35 |
| 0.0098545 | 0.144 | 0.0396825 | 2.56 | 0.25 | 0.1 | [lnc-FANCI-1](http://www.ncbi.nlm.nih.gov/entrez/query.fcgi?cmd=search&db=gene&term=lnc-FANCI-1) | lnc-FANCI-1:1 | [BC031940](http://www.ncbi.nlm.nih.gov/entrez/query.fcgi?db=Nucleotide&term=BC031940) | hs\|15q26.1 |
| 0.0059411 | 0.137 | 0.015873 | 4.78 | 0.47 | 0.1 | [CYP4F35P](http://www.ncbi.nlm.nih.gov/entrez/query.fcgi?cmd=search&db=gene&term=CYP4F35P) | cytochrome P450, family 4, subfamily F, polypeptide 35, pseudogene |  | hs\|18p11.21 |
| 0.0044532 | 0.137 | 0.0238095 | 4.95 | 0.48 | 0.1 | [SSX2](http://www.ncbi.nlm.nih.gov/entrez/query.fcgi?cmd=search&db=gene&term=SSX2) | synovial sarcoma, X breakpoint 2 | [NM_175698](http://www.ncbi.nlm.nih.gov/entrez/query.fcgi?db=Nucleotide&term=NM_175698) | hs\|Xp11.22 |
| 0.002785 | 0.137 | 0.0238095 | 3.96 | 0.38 | 0.1 | [FBXW12](http://www.ncbi.nlm.nih.gov/entrez/query.fcgi?cmd=search&db=gene&term=FBXW12) | F-box and WD repeat domain containing 12 | [NM_207102](http://www.ncbi.nlm.nih.gov/entrez/query.fcgi?db=Nucleotide&term=NM_207102) | hs\|3p21.31 |
| 0.0069591 | 0.138 | 0.0238095 | 2.85 | 0.27 | 0.1 | [C19orf81](http://www.ncbi.nlm.nih.gov/entrez/query.fcgi?cmd=search&db=gene&term=C19orf81) | chromosome 19 open reading frame 81 | [XM_006723188](http://www.ncbi.nlm.nih.gov/entrez/query.fcgi?db=Nucleotide&term=XM_006723188) | hs\|19q13.33 |
| 0.0070812 | 0.138 | 0.0238095 | 2.46 | 0.23 | 0.09 | [GAS2](http://www.ncbi.nlm.nih.gov/entrez/query.fcgi?cmd=search&db=gene&term=GAS2) | growth arrest-specific 2 | [NM_005256](http://www.ncbi.nlm.nih.gov/entrez/query.fcgi?db=Nucleotide&term=NM_005256) | hs\|11p14.3 |
| 0.0081823 | 0.141 | 0.031746 | 2.65 | 0.25 | 0.09 |  |  |  | hs\|12q12 |
| 0.0082348 | 0.141 | 0.0238095 | 4.84 | 0.45 | 0.09 | [FGFR4](http://www.ncbi.nlm.nih.gov/entrez/query.fcgi?cmd=search&db=gene&term=FGFR4) | fibroblast growth factor receptor 4 | [NM_213647](http://www.ncbi.nlm.nih.gov/entrez/query.fcgi?db=Nucleotide&term=NM_213647) | hs\|5q35.2 |
| 0.004143 | 0.137 | 0.0238095 | 3.59 | 0.34 | 0.09 | [C19orf54](http://www.ncbi.nlm.nih.gov/entrez/query.fcgi?cmd=search&db=gene&term=C19orf54) | chromosome 19 open reading frame 54 | [XM_006723153](http://www.ncbi.nlm.nih.gov/entrez/query.fcgi?db=Nucleotide&term=XM_006723153) | hs\|19q13.2 |
| 0.0069423 | 0.138 | 0.0238095 | 3.65 | 0.34 | 0.09 | [LOC440082](http://www.ncbi.nlm.nih.gov/entrez/query.fcgi?cmd=search&db=gene&term=LOC440082) | uncharacterized LOC440082 | [AK131474](http://www.ncbi.nlm.nih.gov/entrez/query.fcgi?db=Nucleotide&term=AK131474) | hs\|12p13.2 |
| 0.0067012 | 0.137 | 0.0238095 | 3.09 | 0.29 | 0.09 | [NPHS1](http://www.ncbi.nlm.nih.gov/entrez/query.fcgi?cmd=search&db=gene&term=NPHS1) | nephrosis 1, congenital, Finnish type (nephrin) | [NM_004646](http://www.ncbi.nlm.nih.gov/entrez/query.fcgi?db=Nucleotide&term=NM_004646) | hs\|19q13.12 |
| 0.0071084 | 0.138 | 0.0238095 | 3.16 | 0.3 | 0.09 | [lnc-FLNB-1](http://www.ncbi.nlm.nih.gov/entrez/query.fcgi?cmd=search&db=gene&term=lnc-FLNB-1) | lnc-FLNB-1:1 |  | hs\|3p14.3 |
| 0.0082428 | 0.141 | 0.031746 | 1.98 | 0.18 | 0.09 | [CRABP1](http://www.ncbi.nlm.nih.gov/entrez/query.fcgi?cmd=search&db=gene&term=CRABP1) | cellular retinoic acid binding protein 1 | [NM_004378](http://www.ncbi.nlm.nih.gov/entrez/query.fcgi?db=Nucleotide&term=NM_004378) | hs\|15q25.1 |
| 0.0085889 | 0.142 | 0.031746 | 3.93 | 0.37 | 0.09 | [GPHA2](http://www.ncbi.nlm.nih.gov/entrez/query.fcgi?cmd=search&db=gene&term=GPHA2) | glycoprotein hormone alpha 2 | [NM_130769](http://www.ncbi.nlm.nih.gov/entrez/query.fcgi?db=Nucleotide&term=NM_130769) | hs\|11q13.1 |
| 0.0088698 | 0.142 | 0.031746 | 2.61 | 0.24 | 0.09 | [SLC39A5](http://www.ncbi.nlm.nih.gov/entrez/query.fcgi?cmd=search&db=gene&term=SLC39A5) | solute carrier family 39 (zinc transporter), member 5 | [NM_001135195](http://www.ncbi.nlm.nih.gov/entrez/query.fcgi?db=Nucleotide&term=NM_001135195) | hs\|12q13.3 |
| 0.0042985 | 0.137 | 0.0238095 | 4.07 | 0.36 | 0.09 | [MAMSTR](http://www.ncbi.nlm.nih.gov/entrez/query.fcgi?cmd=search&db=gene&term=MAMSTR) | MEF2 activating motif and SAP domain containing transcriptional regulator | [NM_182574](http://www.ncbi.nlm.nih.gov/entrez/query.fcgi?db=Nucleotide&term=NM_182574) | hs\|19q13.33 |
| 0.0089134 | 0.142 | 0.0238095 | 3.19 | 0.28 | 0.09 | [LOC101927914](http://www.ncbi.nlm.nih.gov/entrez/query.fcgi?cmd=search&db=gene&term=LOC101927914) | uncharacterized LOC101927914 |  | hs\|7q36.3 |
| 0.0052182 | 0.137 | 0.0238095 | 3.89 | 0.34 | 0.09 | [lnc-FMN1-3](http://www.ncbi.nlm.nih.gov/entrez/query.fcgi?cmd=search&db=gene&term=lnc-FMN1-3) | lnc-FMN1-3:1 | [BX088994](http://www.ncbi.nlm.nih.gov/entrez/query.fcgi?db=Nucleotide&term=BX088994) | hs\|15q13.3 |
| 0.0077827 | 0.14 | 0.031746 | 4.03 | 0.35 | 0.09 | [SLC6A19](http://www.ncbi.nlm.nih.gov/entrez/query.fcgi?cmd=search&db=gene&term=SLC6A19) | solute carrier family 6 (neutral amino acid transporter), member 19 | [NM_001003841](http://www.ncbi.nlm.nih.gov/entrez/query.fcgi?db=Nucleotide&term=NM_001003841) | hs\|5p15.33 |
| 0.0039324 | 0.137 | 0.0238095 | 3.54 | 0.3 | 0.09 | [TMEM52](http://www.ncbi.nlm.nih.gov/entrez/query.fcgi?cmd=search&db=gene&term=TMEM52) | transmembrane protein 52 | [NM_178545](http://www.ncbi.nlm.nih.gov/entrez/query.fcgi?db=Nucleotide&term=NM_178545) | hs\|1p36.33 |
| 0.0029587 | 0.137 | 0.0238095 | 4.33 | 0.36 | 0.08 | [DNASE1](http://www.ncbi.nlm.nih.gov/entrez/query.fcgi?cmd=search&db=gene&term=DNASE1) | deoxyribonuclease I | [NM_005223](http://www.ncbi.nlm.nih.gov/entrez/query.fcgi?db=Nucleotide&term=NM_005223) | hs\|16p13.3 |
| 0.0063548 | 0.137 | 0.0238095 | 4.32 | 0.36 | 0.08 | [ITLN2](http://www.ncbi.nlm.nih.gov/entrez/query.fcgi?cmd=search&db=gene&term=ITLN2) | intelectin 2 | [NM_080878](http://www.ncbi.nlm.nih.gov/entrez/query.fcgi?db=Nucleotide&term=NM_080878) | hs\|1q23.3 |
| 0.0061094 | 0.137 | 0.0238095 | 3.57 | 0.3 | 0.08 | [LINC01122](http://www.ncbi.nlm.nih.gov/entrez/query.fcgi?cmd=search&db=gene&term=LINC01122) | long intergenic non-protein coding RNA 1122 | [NR_033873](http://www.ncbi.nlm.nih.gov/entrez/query.fcgi?db=Nucleotide&term=NR_033873) | hs\|2p16.1 |
| 0.0045214 | 0.137 | 0.0238095 | 4.66 | 0.39 | 0.08 | [ARHGDIG](http://www.ncbi.nlm.nih.gov/entrez/query.fcgi?cmd=search&db=gene&term=ARHGDIG) | Rho GDP dissociation inhibitor (GDI) gamma | [NM_001176](http://www.ncbi.nlm.nih.gov/entrez/query.fcgi?db=Nucleotide&term=NM_001176) | hs\|16p13.3 |
| 0.0055712 | 0.137 | 0.0238095 | 4.01 | 0.33 | 0.08 | [NHEG1](http://www.ncbi.nlm.nih.gov/entrez/query.fcgi?cmd=search&db=gene&term=NHEG1) | neuroblastoma highly expressed 1 | [NR_027994](http://www.ncbi.nlm.nih.gov/entrez/query.fcgi?db=Nucleotide&term=NR_027994) | hs\|6q23.3 |
| 0.0058004 | 0.137 | 0.0238095 | 2.97 | 0.24 | 0.08 |  |  |  | hs\|10q26.3 |
| 0.0085293 | 0.142 | 0.031746 | 2.08 | 0.17 | 0.08 | [MCOLN3](http://www.ncbi.nlm.nih.gov/entrez/query.fcgi?cmd=search&db=gene&term=MCOLN3) | mucolipin 3 | [NM_018298](http://www.ncbi.nlm.nih.gov/entrez/query.fcgi?db=Nucleotide&term=NM_018298) | hs\|1p22.3 |
| 0.0084436 | 0.141 | 0.031746 | 3.24 | 0.26 | 0.08 | [LOC100130264](http://www.ncbi.nlm.nih.gov/entrez/query.fcgi?cmd=search&db=gene&term=LOC100130264) | uncharacterized LOC100130264 | [NR_024564](http://www.ncbi.nlm.nih.gov/entrez/query.fcgi?db=Nucleotide&term=NR_024564) | hs\|20p11.23 |
| 0.0031333 | 0.137 | 0.0238095 | 3.32 | 0.26 | 0.08 | [lnc-MANSC4-1](http://www.ncbi.nlm.nih.gov/entrez/query.fcgi?cmd=search&db=gene&term=lnc-MANSC4-1) | lnc-MANSC4-1:1 |  | hs\|12p11.22 |
| 0.0048384 | 0.137 | 0.0238095 | 2.87 | 0.23 | 0.08 | [KIAA1324](http://www.ncbi.nlm.nih.gov/entrez/query.fcgi?cmd=search&db=gene&term=KIAA1324) | KIAA1324 | [NM_020775](http://www.ncbi.nlm.nih.gov/entrez/query.fcgi?db=Nucleotide&term=NM_020775) | hs\|1p13.3 |
| 0.0058443 | 0.137 | 0.0238095 | 2.88 | 0.23 | 0.08 | [ADCYAP1R1](http://www.ncbi.nlm.nih.gov/entrez/query.fcgi?cmd=search&db=gene&term=ADCYAP1R1) | adenylate cyclase activating polypeptide 1 (pituitary) receptor type I | [NM_001118](http://www.ncbi.nlm.nih.gov/entrez/query.fcgi?db=Nucleotide&term=NM_001118) | hs\|7p14.3 |
| 0.006732 | 0.137 | 0.031746 | 5.39 | 0.42 | 0.08 | [MAGEB17](http://www.ncbi.nlm.nih.gov/entrez/query.fcgi?cmd=search&db=gene&term=MAGEB17) | melanoma antigen family B, 17 |  | hs\|Xp22.2 |
| 0.0055256 | 0.137 | 0.0238095 | 6.74 | 0.53 | 0.08 | [FAM138E](http://www.ncbi.nlm.nih.gov/entrez/query.fcgi?cmd=search&db=gene&term=FAM138E) | family with sequence similarity 138, member E | [NR_026819](http://www.ncbi.nlm.nih.gov/entrez/query.fcgi?db=Nucleotide&term=NR_026819) | hs\|15q26.3 |
| 0.0033651 | 0.137 | 0.015873 | 2.38 | 0.18 | 0.08 | [ACADL](http://www.ncbi.nlm.nih.gov/entrez/query.fcgi?cmd=search&db=gene&term=ACADL) | acyl-CoA dehydrogenase, long chain | [NM_001608](http://www.ncbi.nlm.nih.gov/entrez/query.fcgi?db=Nucleotide&term=NM_001608) | hs\|2q34 |
| 0.0054146 | 0.137 | 0.031746 | 3.88 | 0.3 | 0.08 |  |  | [AK093872](http://www.ncbi.nlm.nih.gov/entrez/query.fcgi?db=Nucleotide&term=AK093872) | hs\|22q12.1 |
| 0.0015604 | 0.137 | 0.015873 | 3.95 | 0.3 | 0.08 | [lnc-CCRN4L-7](http://www.ncbi.nlm.nih.gov/entrez/query.fcgi?cmd=search&db=gene&term=lnc-CCRN4L-7) | lnc-CCRN4L-7:1 |  | hs\|4q28.3 |
| 5.22E-05 | 0.137 | 0.0079365 | 4.49 | 0.35 | 0.08 | [PGC](http://www.ncbi.nlm.nih.gov/entrez/query.fcgi?cmd=search&db=gene&term=PGC) | progastricsin (pepsinogen C) | [NM_001166424](http://www.ncbi.nlm.nih.gov/entrez/query.fcgi?db=Nucleotide&term=NM_001166424) | hs\|6p21.1 |
| 0.0037741 | 0.137 | 0.015873 | 4.46 | 0.34 | 0.08 |  |  |  | hs\|Xp22.33 |
| 0.0086531 | 0.142 | 0.0396825 | 2.25 | 0.17 | 0.08 | [LOC644838](http://www.ncbi.nlm.nih.gov/entrez/query.fcgi?cmd=search&db=gene&term=LOC644838) | uncharacterized LOC644838 |  | hs\|2p14 |
| 0.0032915 | 0.137 | 0.0238095 | 4.71 | 0.36 | 0.08 |  |  |  | hs\|5p15.2 |
| 0.0066922 | 0.137 | 0.0238095 | 3.8 | 0.29 | 0.08 | [LINC01389](http://www.ncbi.nlm.nih.gov/entrez/query.fcgi?cmd=search&db=gene&term=LINC01389) | long intergenic non-protein coding RNA 1389 | [NR_126355](http://www.ncbi.nlm.nih.gov/entrez/query.fcgi?db=Nucleotide&term=NR_126355) | hs\|1p33 |
| 0.0014205 | 0.137 | 0.0238095 | 3.69 | 0.27 | 0.07 | [lnc-CITED2-1](http://www.ncbi.nlm.nih.gov/entrez/query.fcgi?cmd=search&db=gene&term=lnc-CITED2-1) | lnc-CITED2-1:1 |  | hs\|6q24.1 |
| 0.0037375 | 0.137 | 0.0238095 | 5.04 | 0.37 | 0.07 | [lnc-DMRT2-1](http://www.ncbi.nlm.nih.gov/entrez/query.fcgi?cmd=search&db=gene&term=lnc-DMRT2-1) | lnc-DMRT2-1:3 |  | hs\|9p24.3 |
| 0.0017375 | 0.137 | 0.0238095 | 4.68 | 0.34 | 0.07 | [lnc-STT3B-1](http://www.ncbi.nlm.nih.gov/entrez/query.fcgi?cmd=search&db=gene&term=lnc-STT3B-1) | lnc-STT3B-1:1 |  | hs\|3p23 |
| 0.0096843 | 0.144 | 0.031746 | 2.53 | 0.18 | 0.07 | [LOC440390](http://www.ncbi.nlm.nih.gov/entrez/query.fcgi?cmd=search&db=gene&term=LOC440390) | uncharacterized LOC440390 | [NR_126008](http://www.ncbi.nlm.nih.gov/entrez/query.fcgi?db=Nucleotide&term=NR_126008) | hs\|16q24.1 |
| 0.0070024 | 0.138 | 0.031746 | 2.76 | 0.19 | 0.07 | [lnc-RALGDS-1](http://www.ncbi.nlm.nih.gov/entrez/query.fcgi?cmd=search&db=gene&term=lnc-RALGDS-1) | lnc-RALGDS-1:1 |  | hs\|9q34.2 |
| 0.0014251 | 0.137 | 0.015873 | 4.77 | 0.32 | 0.07 | [CLPSL2](http://www.ncbi.nlm.nih.gov/entrez/query.fcgi?cmd=search&db=gene&term=CLPSL2) | colipase-like 2 | [NM_001286550](http://www.ncbi.nlm.nih.gov/entrez/query.fcgi?db=Nucleotide&term=NM_001286550) | hs\|6p21.31 |
| 0.0067854 | 0.137 | 0.0238095 | 2.8 | 0.19 | 0.07 | [KIAA1324](http://www.ncbi.nlm.nih.gov/entrez/query.fcgi?cmd=search&db=gene&term=KIAA1324) | KIAA1324 | [NM_020775](http://www.ncbi.nlm.nih.gov/entrez/query.fcgi?db=Nucleotide&term=NM_020775) | hs\|1p13.3 |
| 0.0005089 | 0.137 | 0.0079365 | 4.07 | 0.27 | 0.07 | [MT1G](http://www.ncbi.nlm.nih.gov/entrez/query.fcgi?cmd=search&db=gene&term=MT1G) | metallothionein 1G | [NM_001301267](http://www.ncbi.nlm.nih.gov/entrez/query.fcgi?db=Nucleotide&term=NM_001301267) | hs\|16q13 |
| 0.0093461 | 0.143 | 0.0238095 | 1.58 | 0.1 | 0.07 | [PRSS3P2](http://www.ncbi.nlm.nih.gov/entrez/query.fcgi?cmd=search&db=gene&term=PRSS3P2) | protease, serine, 3 pseudogene 2 | [NR_001296](http://www.ncbi.nlm.nih.gov/entrez/query.fcgi?db=Nucleotide&term=NR_001296) | hs\|7q34 |
| 0.0005438 | 0.137 | 0.0079365 | 4.89 | 0.31 | 0.06 | [SPACA3](http://www.ncbi.nlm.nih.gov/entrez/query.fcgi?cmd=search&db=gene&term=SPACA3) | sperm acrosome associated 3 | [NM_173847](http://www.ncbi.nlm.nih.gov/entrez/query.fcgi?db=Nucleotide&term=NM_173847) | hs\|17q11.2 |
| 0.0074203 | 0.139 | 0.0238095 | 1.78 | 0.11 | 0.06 | [HS6ST3](http://www.ncbi.nlm.nih.gov/entrez/query.fcgi?cmd=search&db=gene&term=HS6ST3) | heparan sulfate 6-O-sulfotransferase 3 | [NM_153456](http://www.ncbi.nlm.nih.gov/entrez/query.fcgi?db=Nucleotide&term=NM_153456) | hs\|13q32.1 |
| 0.0064944 | 0.137 | 0.031746 | 5.65 | 0.36 | 0.06 | [DLX2-AS1](http://www.ncbi.nlm.nih.gov/entrez/query.fcgi?cmd=search&db=gene&term=DLX2-AS1) | DLX2 antisense RNA 1 (head to head) |  | hs\|2q31.1 |
| 0.0027614 | 0.137 | 0.015873 | 3.4 | 0.21 | 0.06 | [TRIM50](http://www.ncbi.nlm.nih.gov/entrez/query.fcgi?cmd=search&db=gene&term=TRIM50) | tripartite motif containing 50 | [NM_178125](http://www.ncbi.nlm.nih.gov/entrez/query.fcgi?db=Nucleotide&term=NM_178125) | hs\|7q11.23 |
| 0.002067 | 0.137 | 0.0238095 | 2.29 | 0.14 | 0.06 | [PRLR](http://www.ncbi.nlm.nih.gov/entrez/query.fcgi?cmd=search&db=gene&term=PRLR) | prolactin receptor | [NM_000949](http://www.ncbi.nlm.nih.gov/entrez/query.fcgi?db=Nucleotide&term=NM_000949) | hs\|5p13.2 |
| 0.0079532 | 0.14 | 0.0238095 | 2.45 | 0.15 | 0.06 | [KIF1A](http://www.ncbi.nlm.nih.gov/entrez/query.fcgi?cmd=search&db=gene&term=KIF1A) | kinesin family member 1A | [NM_001244008](http://www.ncbi.nlm.nih.gov/entrez/query.fcgi?db=Nucleotide&term=NM_001244008) | hs\|2q37.3 |
| 0.0054307 | 0.137 | 0.0238095 | 3.86 | 0.24 | 0.06 | [CFAP74](http://www.ncbi.nlm.nih.gov/entrez/query.fcgi?cmd=search&db=gene&term=CFAP74) | cilia and flagella associated protein 74 | [AK094642](http://www.ncbi.nlm.nih.gov/entrez/query.fcgi?db=Nucleotide&term=AK094642) | hs\|1p36.33 |
| 0.0054176 | 0.137 | 0.0238095 | 2.88 | 0.18 | 0.06 | [OR1L3](http://www.ncbi.nlm.nih.gov/entrez/query.fcgi?cmd=search&db=gene&term=OR1L3) | olfactory receptor, family 1, subfamily L, member 3 | [NM_001005234](http://www.ncbi.nlm.nih.gov/entrez/query.fcgi?db=Nucleotide&term=NM_001005234) | hs\|9q33.2 |
| 0.0086189 | 0.142 | 0.031746 | 3.2 | 0.2 | 0.06 |  |  |  | hs\|15q22.31 |
| 0.0082825 | 0.141 | 0.0396825 | 2.83 | 0.17 | 0.06 | [DTX1](http://www.ncbi.nlm.nih.gov/entrez/query.fcgi?cmd=search&db=gene&term=DTX1) | deltex 1, E3 ubiquitin ligase | [NM_004416](http://www.ncbi.nlm.nih.gov/entrez/query.fcgi?db=Nucleotide&term=NM_004416) | hs\|12q24.13 |
| 0.0042187 | 0.137 | 0.0238095 | 3.44 | 0.21 | 0.06 |  |  | [DA193375](http://www.ncbi.nlm.nih.gov/entrez/query.fcgi?db=Nucleotide&term=DA193375) | hs\|18q11.2 |
| 0.0069422 | 0.138 | 0.0238095 | 3.29 | 0.2 | 0.06 | [LINC00963](http://www.ncbi.nlm.nih.gov/entrez/query.fcgi?cmd=search&db=gene&term=LINC00963) | long intergenic non-protein coding RNA 963 |  | hs\|9q34.11 |
| 0.0063701 | 0.137 | 0.0238095 | 3.05 | 0.18 | 0.06 | [DNAJC27-AS1](http://www.ncbi.nlm.nih.gov/entrez/query.fcgi?cmd=search&db=gene&term=DNAJC27-AS1) | DNAJC27 antisense RNA 1 | [NR_034113](http://www.ncbi.nlm.nih.gov/entrez/query.fcgi?db=Nucleotide&term=NR_034113) | hs\|2p23.3 |
| 0.0014576 | 0.137 | 0.0079365 | 2.1 | 0.12 | 0.06 | [PWRN1](http://www.ncbi.nlm.nih.gov/entrez/query.fcgi?cmd=search&db=gene&term=PWRN1) | Prader-Willi region non-protein coding RNA 1 | [NR_026646](http://www.ncbi.nlm.nih.gov/entrez/query.fcgi?db=Nucleotide&term=NR_026646) | hs\|15q11.2 |
| 0.0034068 | 0.137 | 0.0238095 | 3.64 | 0.22 | 0.06 | [TMEM52](http://www.ncbi.nlm.nih.gov/entrez/query.fcgi?cmd=search&db=gene&term=TMEM52) | transmembrane protein 52 | [NM_178545](http://www.ncbi.nlm.nih.gov/entrez/query.fcgi?db=Nucleotide&term=NM_178545) | hs\|1p36.33 |
| 0.0005782 | 0.137 | 0.0079365 | 6.39 | 0.38 | 0.06 | [MT1H](http://www.ncbi.nlm.nih.gov/entrez/query.fcgi?cmd=search&db=gene&term=MT1H) | metallothionein 1H | [NM_005951](http://www.ncbi.nlm.nih.gov/entrez/query.fcgi?db=Nucleotide&term=NM_005951) | hs\|16q13 |
| 0.0032572 | 0.137 | 0.0238095 | 5.16 | 0.3 | 0.06 | [LINC00856](http://www.ncbi.nlm.nih.gov/entrez/query.fcgi?cmd=search&db=gene&term=LINC00856) | long intergenic non-protein coding RNA 856 | [AF086162](http://www.ncbi.nlm.nih.gov/entrez/query.fcgi?db=Nucleotide&term=AF086162) | hs\|10q22.3 |
| 0.0085601 | 0.142 | 0.0238095 | 1.59 | 0.091 | 0.06 |  |  |  |  |
| 0.0019615 | 0.137 | 0.015873 | 3.33 | 0.19 | 0.06 | [ACSM6](http://www.ncbi.nlm.nih.gov/entrez/query.fcgi?cmd=search&db=gene&term=ACSM6) | acyl-CoA synthetase medium-chain family member 6 | [NM_207321](http://www.ncbi.nlm.nih.gov/entrez/query.fcgi?db=Nucleotide&term=NM_207321) | hs\|10q23.33 |
| 0.0005174 | 0.137 | 0.0079365 | 4.02 | 0.23 | 0.06 | [LOC100506281](http://www.ncbi.nlm.nih.gov/entrez/query.fcgi?cmd=search&db=gene&term=LOC100506281) | uncharacterized LOC100506281 |  | hs\|16q23.1 |
| 0.0042566 | 0.137 | 0.015873 | 3.52 | 0.2 | 0.06 | [SLC30A2](http://www.ncbi.nlm.nih.gov/entrez/query.fcgi?cmd=search&db=gene&term=SLC30A2) | solute carrier family 30 (zinc transporter), member 2 | [NM_001004434](http://www.ncbi.nlm.nih.gov/entrez/query.fcgi?db=Nucleotide&term=NM_001004434) | hs\|1p36.11 |
| 0.0042162 | 0.137 | 0.0238095 | 3.14 | 0.18 | 0.06 |  |  |  | hs\|12p13.32 |
| 0.0091584 | 0.143 | 0.0396825 | 3.39 | 0.19 | 0.06 | [RASA4B](http://www.ncbi.nlm.nih.gov/entrez/query.fcgi?cmd=search&db=gene&term=RASA4B) | RAS p21 protein activator 4B | [NM_001277335](http://www.ncbi.nlm.nih.gov/entrez/query.fcgi?db=Nucleotide&term=NM_001277335) | hs\|7q22.1 |
| 0.0047495 | 0.137 | 0.0238095 | 3.51 | 0.19 | 0.06 | [GSTA7P](http://www.ncbi.nlm.nih.gov/entrez/query.fcgi?cmd=search&db=gene&term=GSTA7P) | glutathione S-transferase alpha 7, pseudogene | [NR_033760](http://www.ncbi.nlm.nih.gov/entrez/query.fcgi?db=Nucleotide&term=NR_033760) | hs\|6p12.2 |
| 0.0044622 | 0.137 | 0.0238095 | 3.37 | 0.18 | 0.05 | [IL36RN](http://www.ncbi.nlm.nih.gov/entrez/query.fcgi?cmd=search&db=gene&term=IL36RN) | interleukin 36 receptor antagonist |  | hs\|2q13 |
| 0.0022033 | 0.137 | 0.015873 | 5.08 | 0.28 | 0.05 | [AQP8](http://www.ncbi.nlm.nih.gov/entrez/query.fcgi?cmd=search&db=gene&term=AQP8) | aquaporin 8 | [NM_001169](http://www.ncbi.nlm.nih.gov/entrez/query.fcgi?db=Nucleotide&term=NM_001169) | hs\|16p12.1 |
| 0.0036918 | 0.137 | 0.015873 | 2.24 | 0.12 | 0.05 | [lnc-C15orf2-9](http://www.ncbi.nlm.nih.gov/entrez/query.fcgi?cmd=search&db=gene&term=lnc-C15orf2-9) | lnc-C15orf2-9:10 |  | hs\|15q11.2 |
| 0.0022001 | 0.137 | 0.0238095 | 6.42 | 0.34 | 0.05 | [MAT1A](http://www.ncbi.nlm.nih.gov/entrez/query.fcgi?cmd=search&db=gene&term=MAT1A) | methionine adenosyltransferase I, alpha | [NM_000429](http://www.ncbi.nlm.nih.gov/entrez/query.fcgi?db=Nucleotide&term=NM_000429) | hs\|10q23.1 |
| 0.0027696 | 0.137 | 0.0238095 | 3.53 | 0.19 | 0.05 | [BRSK2](http://www.ncbi.nlm.nih.gov/entrez/query.fcgi?cmd=search&db=gene&term=BRSK2) | BR serine/threonine kinase 2 | [NM_001256627](http://www.ncbi.nlm.nih.gov/entrez/query.fcgi?db=Nucleotide&term=NM_001256627) | hs\|11p15.5 |
| 0.0026929 | 0.137 | 0.0238095 | 4.38 | 0.23 | 0.05 |  |  |  | hs\|22q11.1 |
| 0.0035732 | 0.137 | 0.0238095 | 4.22 | 0.21 | 0.05 | [BTN2A1](http://www.ncbi.nlm.nih.gov/entrez/query.fcgi?cmd=search&db=gene&term=BTN2A1) | butyrophilin, subfamily 2, member A1 | [NM_001197234](http://www.ncbi.nlm.nih.gov/entrez/query.fcgi?db=Nucleotide&term=NM_001197234) | hs\|6p22.2 |
| 0.0064187 | 0.137 | 0.0238095 | 3.1 | 0.16 | 0.05 | [SYT14](http://www.ncbi.nlm.nih.gov/entrez/query.fcgi?cmd=search&db=gene&term=SYT14) | synaptotagmin XIV | [NM_153262](http://www.ncbi.nlm.nih.gov/entrez/query.fcgi?db=Nucleotide&term=NM_153262) | hs\|1q32.2 |
| 0.0040242 | 0.137 | 0.0238095 | 3.54 | 0.18 | 0.05 |  |  | [XR_424607](http://www.ncbi.nlm.nih.gov/entrez/query.fcgi?db=Nucleotide&term=XR_424607) | hs\|15q22.32 |
| 0.0039769 | 0.137 | 0.0238095 | 3.4 | 0.17 | 0.05 | [XLOC_l2_006973](http://www.ncbi.nlm.nih.gov/entrez/query.fcgi?cmd=search&db=gene&term=XLOC_l2_006973) |  |  | hs\|19p12 |
| 0.0044367 | 0.137 | 0.0238095 | 5.75 | 0.28 | 0.05 | [GPR133](http://www.ncbi.nlm.nih.gov/entrez/query.fcgi?cmd=search&db=gene&term=GPR133) | G protein-coupled receptor 133 | [NM_198827](http://www.ncbi.nlm.nih.gov/entrez/query.fcgi?db=Nucleotide&term=NM_198827) | hs\|12q24.33 |
| 0.0037993 | 0.137 | 0.0238095 | 4.85 | 0.24 | 0.05 | [PM20D1](http://www.ncbi.nlm.nih.gov/entrez/query.fcgi?cmd=search&db=gene&term=PM20D1) | peptidase M20 domain containing 1 | [NM_152491](http://www.ncbi.nlm.nih.gov/entrez/query.fcgi?db=Nucleotide&term=NM_152491) | hs\|1q32.1 |
| 0.0022199 | 0.137 | 0.0079365 | 6.09 | 0.3 | 0.05 | [SPX](http://www.ncbi.nlm.nih.gov/entrez/query.fcgi?cmd=search&db=gene&term=SPX) | spexin hormone | [AK311217](http://www.ncbi.nlm.nih.gov/entrez/query.fcgi?db=Nucleotide&term=AK311217) | hs\|12p12.1 |
| 0.0036916 | 0.137 | 0.015873 | 5.45 | 0.27 | 0.05 | [GNMT](http://www.ncbi.nlm.nih.gov/entrez/query.fcgi?cmd=search&db=gene&term=GNMT) | glycine N-methyltransferase | [NM_018960](http://www.ncbi.nlm.nih.gov/entrez/query.fcgi?db=Nucleotide&term=NM_018960) | hs\|6p21.1 |
| 0.0011548 | 0.137 | 0.015873 | 4.12 | 0.2 | 0.05 | [LOC645434](http://www.ncbi.nlm.nih.gov/entrez/query.fcgi?cmd=search&db=gene&term=LOC645434) | uncharacterized LOC645434 |  | hs\|6q24.1 |
| 0.0005803 | 0.137 | 0.0079365 | 3.09 | 0.14 | 0.05 | [lnc-AFM-1](http://www.ncbi.nlm.nih.gov/entrez/query.fcgi?cmd=search&db=gene&term=lnc-AFM-1) | lnc-AFM-1:4 |  | hs\|4q13.3 |
| 0.0023793 | 0.137 | 0.015873 | 3.91 | 0.18 | 0.05 | [LOC729658](http://www.ncbi.nlm.nih.gov/entrez/query.fcgi?cmd=search&db=gene&term=LOC729658) | uncharacterized LOC729658 | [XR_110196](http://www.ncbi.nlm.nih.gov/entrez/query.fcgi?db=Nucleotide&term=XR_110196) | hs\|6q26 |
| 0.0063623 | 0.137 | 0.031746 | 5.84 | 0.27 | 0.05 | [XLOC_l2_009328](http://www.ncbi.nlm.nih.gov/entrez/query.fcgi?cmd=search&db=gene&term=XLOC_l2_009328) |  |  | hs\|22q11.23 |
| 0.005573 | 0.137 | 0.031746 | 5.64 | 0.25 | 0.05 | [UNC5A](http://www.ncbi.nlm.nih.gov/entrez/query.fcgi?cmd=search&db=gene&term=UNC5A) | unc-5 homolog A (C. elegans) | [NM_133369](http://www.ncbi.nlm.nih.gov/entrez/query.fcgi?db=Nucleotide&term=NM_133369) | hs\|5q35.2 |
| 0.0069967 | 0.138 | 0.0238095 | 1.89 | 0.085 | 0.04 | [AMY1C](http://www.ncbi.nlm.nih.gov/entrez/query.fcgi?cmd=search&db=gene&term=AMY1C) | amylase, alpha 1C (salivary) | [NM_001008219](http://www.ncbi.nlm.nih.gov/entrez/query.fcgi?db=Nucleotide&term=NM_001008219) | hs\|1p21.1 |
| 0.0003337 | 0.137 | 0.0079365 | 3.35 | 0.15 | 0.04 | [FBP2](http://www.ncbi.nlm.nih.gov/entrez/query.fcgi?cmd=search&db=gene&term=FBP2) | fructose-1,6-bisphosphatase 2 | [NM_003837](http://www.ncbi.nlm.nih.gov/entrez/query.fcgi?db=Nucleotide&term=NM_003837) | hs\|9q22.32 |
| 0.0020543 | 0.137 | 0.0238095 | 4.37 | 0.19 | 0.04 | [LOC102724987](http://www.ncbi.nlm.nih.gov/entrez/query.fcgi?cmd=search&db=gene&term=LOC102724987) | uncharacterized LOC102724987 |  | hs\|22q11.23 |
| 0.0086692 | 0.142 | 0.0396825 | 3.02 | 0.13 | 0.04 | [LOC101928223](http://www.ncbi.nlm.nih.gov/entrez/query.fcgi?cmd=search&db=gene&term=LOC101928223) | uncharacterized LOC101928223 | [NR_125889](http://www.ncbi.nlm.nih.gov/entrez/query.fcgi?db=Nucleotide&term=NR_125889) | hs\|4q33 |
| 0.0002627 | 0.137 | 0.0079365 | 4.01 | 0.17 | 0.04 | [PGC](http://www.ncbi.nlm.nih.gov/entrez/query.fcgi?cmd=search&db=gene&term=PGC) | progastricsin (pepsinogen C) | [NM_002630](http://www.ncbi.nlm.nih.gov/entrez/query.fcgi?db=Nucleotide&term=NM_002630) | hs\|6p21.1 |
| 0.0006532 | 0.137 | 0.0079365 | 4.29 | 0.17 | 0.04 | [LOC285191](http://www.ncbi.nlm.nih.gov/entrez/query.fcgi?cmd=search&db=gene&term=LOC285191) | uncharacterized LOC285191 | [XR_249328](http://www.ncbi.nlm.nih.gov/entrez/query.fcgi?db=Nucleotide&term=XR_249328) | hs\|2q37.3 |
| 0.0088307 | 0.142 | 0.0396825 | 2.28 | 0.09 | 0.04 | [MYH7](http://www.ncbi.nlm.nih.gov/entrez/query.fcgi?cmd=search&db=gene&term=MYH7) | myosin, heavy chain 7, cardiac muscle, beta | [NM_000257](http://www.ncbi.nlm.nih.gov/entrez/query.fcgi?db=Nucleotide&term=NM_000257) | hs\|14q11.2 |
| 0.0035516 | 0.137 | 0.015873 | 4.73 | 0.18 | 0.04 | [FLJ38122](http://www.ncbi.nlm.nih.gov/entrez/query.fcgi?cmd=search&db=gene&term=FLJ38122) | uncharacterized LOC401289 | [AK095441](http://www.ncbi.nlm.nih.gov/entrez/query.fcgi?db=Nucleotide&term=AK095441) | hs\|6q27 |
| 0.0044264 | 0.137 | 0.0238095 | 3.43 | 0.13 | 0.04 | [LOC285095](http://www.ncbi.nlm.nih.gov/entrez/query.fcgi?cmd=search&db=gene&term=LOC285095) | uncharacterized LOC285095 | [XR_430644](http://www.ncbi.nlm.nih.gov/entrez/query.fcgi?db=Nucleotide&term=XR_430644) | hs\|2q37.3 |
| 0.0006761 | 0.137 | 0.0079365 | 3.54 | 0.14 | 0.04 | [GRPR](http://www.ncbi.nlm.nih.gov/entrez/query.fcgi?cmd=search&db=gene&term=GRPR) | gastrin-releasing peptide receptor | [NM_005314](http://www.ncbi.nlm.nih.gov/entrez/query.fcgi?db=Nucleotide&term=NM_005314) | hs\|Xp22.2 |
| 0.0035291 | 0.137 | 0.0238095 | 6.29 | 0.24 | 0.04 | [lnc-LPA-1](http://www.ncbi.nlm.nih.gov/entrez/query.fcgi?cmd=search&db=gene&term=lnc-LPA-1) | lnc-LPA-1:1 |  | hs\|6q26 |
| 0.0057252 | 0.137 | 0.0238095 | 3.34 | 0.13 | 0.04 | [TMED6](http://www.ncbi.nlm.nih.gov/entrez/query.fcgi?cmd=search&db=gene&term=TMED6) | transmembrane emp24 protein transport domain containing 6 | [NM_144676](http://www.ncbi.nlm.nih.gov/entrez/query.fcgi?db=Nucleotide&term=NM_144676) | hs\|16q22.1 |
| 0.0002418 | 0.137 | 0.0079365 | 4.72 | 0.18 | 0.04 |  |  | [XR_253006](http://www.ncbi.nlm.nih.gov/entrez/query.fcgi?db=Nucleotide&term=XR_253006) | hs\|12p13.32 |
| 0.0032769 | 0.137 | 0.0238095 | 4.39 | 0.17 | 0.04 | [LINC01167](http://www.ncbi.nlm.nih.gov/entrez/query.fcgi?cmd=search&db=gene&term=LINC01167) | long intergenic non-protein coding RNA 1167 |  | hs\|10q26.3 |
| 0.0024331 | 0.137 | 0.0238095 | 5.51 | 0.21 | 0.04 | [TEX11](http://www.ncbi.nlm.nih.gov/entrez/query.fcgi?cmd=search&db=gene&term=TEX11) | testis expressed 11 | [NM_001003811](http://www.ncbi.nlm.nih.gov/entrez/query.fcgi?db=Nucleotide&term=NM_001003811) | hs\|Xq13.1 |
| 0.009132 | 0.143 | 0.031746 | 5.57 | 0.21 | 0.04 |  |  |  | hs\|8q23.2 |
| 0.004812 | 0.137 | 0.015873 | 2.98 | 0.11 | 0.04 | [CELA3A](http://www.ncbi.nlm.nih.gov/entrez/query.fcgi?cmd=search&db=gene&term=CELA3A) | chymotrypsin-like elastase family, member 3A | [AK308514](http://www.ncbi.nlm.nih.gov/entrez/query.fcgi?db=Nucleotide&term=AK308514) | hs\|1p36.12 |
| 0.0077562 | 0.14 | 0.047619 | 3.39 | 0.12 | 0.04 | [SLC16A12](http://www.ncbi.nlm.nih.gov/entrez/query.fcgi?cmd=search&db=gene&term=SLC16A12) | solute carrier family 16, member 12 | [NM_213606](http://www.ncbi.nlm.nih.gov/entrez/query.fcgi?db=Nucleotide&term=NM_213606) | hs\|10q23.31 |
| 0.0052248 | 0.137 | 0.0238095 | 5.82 | 0.2 | 0.03 | [DPP10](http://www.ncbi.nlm.nih.gov/entrez/query.fcgi?cmd=search&db=gene&term=DPP10) | dipeptidyl-peptidase 10 (non-functional) | [NM_001178037](http://www.ncbi.nlm.nih.gov/entrez/query.fcgi?db=Nucleotide&term=NM_001178037) | hs\|2q14.1 |
| 0.0032135 | 0.137 | 0.015873 | 4.64 | 0.15 | 0.03 | [PNLIPRP1](http://www.ncbi.nlm.nih.gov/entrez/query.fcgi?cmd=search&db=gene&term=PNLIPRP1) | pancreatic lipase-related protein 1 | [NM_006229](http://www.ncbi.nlm.nih.gov/entrez/query.fcgi?db=Nucleotide&term=NM_006229) | hs\|10q25.3 |
| 0.0065117 | 0.137 | 0.031746 | 3.57 | 0.12 | 0.03 | [lnc-CCDC166-1](http://www.ncbi.nlm.nih.gov/entrez/query.fcgi?cmd=search&db=gene&term=lnc-CCDC166-1) | lnc-CCDC166-1:2 | [AA405962](http://www.ncbi.nlm.nih.gov/entrez/query.fcgi?db=Nucleotide&term=AA405962) | hs\|8q24.3 |
| 0.006688 | 0.137 | 0.031746 | 3.72 | 0.12 | 0.03 |  |  |  | hs\|Yq11.221 |
| 0.004119 | 0.137 | 0.0238095 | 5.79 | 0.18 | 0.03 |  |  |  | hs\|2q31.1 |
| 0.0038698 | 0.137 | 0.0238095 | 5.04 | 0.16 | 0.03 |  |  |  | hs\|19p13.3 |
| 3.50E-05 | 0.137 | 0.0079365 | 12.72 | 0.39 | 0.03 | [PGA3](http://www.ncbi.nlm.nih.gov/entrez/query.fcgi?cmd=search&db=gene&term=PGA3) | pepsinogen 3, group I (pepsinogen A) | [NM_001079807](http://www.ncbi.nlm.nih.gov/entrez/query.fcgi?db=Nucleotide&term=NM_001079807) | hs\|11q12.2 |
| 0.0058896 | 0.137 | 0.0238095 | 2.8 | 0.085 | 0.03 | [MYOC](http://www.ncbi.nlm.nih.gov/entrez/query.fcgi?cmd=search&db=gene&term=MYOC) | myocilin, trabecular meshwork inducible glucocorticoid response | [NM_000261](http://www.ncbi.nlm.nih.gov/entrez/query.fcgi?db=Nucleotide&term=NM_000261) | hs\|1q24.3 |
| 0.0088311 | 0.142 | 0.031746 | 7.87 | 0.23 | 0.03 |  |  | [DA740059](http://www.ncbi.nlm.nih.gov/entrez/query.fcgi?db=Nucleotide&term=DA740059) | hs\|16q22.3 |
| 0.00941 | 0.143 | 0.047619 | 2.28 | 0.066 | 0.03 | [PRSS3](http://www.ncbi.nlm.nih.gov/entrez/query.fcgi?cmd=search&db=gene&term=PRSS3) | protease, serine, 3 | [NM_002771](http://www.ncbi.nlm.nih.gov/entrez/query.fcgi?db=Nucleotide&term=NM_002771) | hs\|9p13.3 |
| 0.0090161 | 0.143 | 0.031746 | 6.93 | 0.2 | 0.03 | [ATP4A](http://www.ncbi.nlm.nih.gov/entrez/query.fcgi?cmd=search&db=gene&term=ATP4A) | ATPase, H+/K+ exchanging, alpha polypeptide | [NM_000704](http://www.ncbi.nlm.nih.gov/entrez/query.fcgi?db=Nucleotide&term=NM_000704) | hs\|19q13.12 |
| 0.0038663 | 0.137 | 0.0238095 | 4.19 | 0.11 | 0.03 |  |  |  | hs\|12q21.33 |
| 0.005465 | 0.137 | 0.0238095 | 4.28 | 0.11 | 0.03 | [GPR50](http://www.ncbi.nlm.nih.gov/entrez/query.fcgi?cmd=search&db=gene&term=GPR50) | G protein-coupled receptor 50 | [NM_004224](http://www.ncbi.nlm.nih.gov/entrez/query.fcgi?db=Nucleotide&term=NM_004224) | hs\|Xq28 |
| 0.0058547 | 0.137 | 0.0238095 | 3.13 | 0.079 | 0.03 | [KLK1](http://www.ncbi.nlm.nih.gov/entrez/query.fcgi?cmd=search&db=gene&term=KLK1) | kallikrein 1 | [NM_002257](http://www.ncbi.nlm.nih.gov/entrez/query.fcgi?db=Nucleotide&term=NM_002257) | hs\|19q13.33 |
| 0.0052454 | 0.137 | 0.0238095 | 4.66 | 0.12 | 0.03 | [SNTG2](http://www.ncbi.nlm.nih.gov/entrez/query.fcgi?cmd=search&db=gene&term=SNTG2) | syntrophin, gamma 2 | [NM_018968](http://www.ncbi.nlm.nih.gov/entrez/query.fcgi?db=Nucleotide&term=NM_018968) | hs\|2p25.3 |
| 0.0039341 | 0.137 | 0.0238095 | 6.23 | 0.16 | 0.03 | [EGF](http://www.ncbi.nlm.nih.gov/entrez/query.fcgi?cmd=search&db=gene&term=EGF) | epidermal growth factor | [NM_001963](http://www.ncbi.nlm.nih.gov/entrez/query.fcgi?db=Nucleotide&term=NM_001963) | hs\|4q25 |
| 0.0048879 | 0.137 | 0.0238095 | 4.41 | 0.11 | 0.03 | [RBPJL](http://www.ncbi.nlm.nih.gov/entrez/query.fcgi?cmd=search&db=gene&term=RBPJL) | recombination signal binding protein for immunoglobulin kappa J region-like | [NM_014276](http://www.ncbi.nlm.nih.gov/entrez/query.fcgi?db=Nucleotide&term=NM_014276) | hs\|20q13.12 |
| 0.0066149 | 0.137 | 0.031746 | 4.4 | 0.1 | 0.02 | [BASP1P1](http://www.ncbi.nlm.nih.gov/entrez/query.fcgi?cmd=search&db=gene&term=BASP1P1) | brain abundant, membrane attached signal protein 1 pseudogene 1 | [NR_033774](http://www.ncbi.nlm.nih.gov/entrez/query.fcgi?db=Nucleotide&term=NR_033774) | hs\|13q12.12 |
| 0.0034917 | 0.137 | 0.0238095 | 4.44 | 0.1 | 0.02 | [ERP27](http://www.ncbi.nlm.nih.gov/entrez/query.fcgi?cmd=search&db=gene&term=ERP27) | endoplasmic reticulum protein 27 | [NM_152321](http://www.ncbi.nlm.nih.gov/entrez/query.fcgi?db=Nucleotide&term=NM_152321) | hs\|12p12.3 |
| 0.0057519 | 0.137 | 0.0238095 | 2.58 | 0.057 | 0.02 | [AMY1C](http://www.ncbi.nlm.nih.gov/entrez/query.fcgi?cmd=search&db=gene&term=AMY1C) | amylase, alpha 1C (salivary) | [NM_001008219](http://www.ncbi.nlm.nih.gov/entrez/query.fcgi?db=Nucleotide&term=NM_001008219) | hs\|1p21.1 |
| 0.0059241 | 0.137 | 0.0238095 | 5.16 | 0.11 | 0.02 | [lnc-MAP3K2-1](http://www.ncbi.nlm.nih.gov/entrez/query.fcgi?cmd=search&db=gene&term=lnc-MAP3K2-1) | lnc-MAP3K2-1:1 |  | hs\|2q14.3 |
| 0.0027606 | 0.137 | 0.0238095 | 4.41 | 0.092 | 0.02 |  |  |  | hs\|10p12.2 |
| 0.0096182 | 0.144 | 0.0396825 | 3.43 | 0.071 | 0.02 |  |  | [BX103273](http://www.ncbi.nlm.nih.gov/entrez/query.fcgi?db=Nucleotide&term=BX103273) | hs\|19q13.33 |
| 0.0075905 | 0.139 | 0.031746 | 2.8 | 0.056 | 0.02 | [PTF1A](http://www.ncbi.nlm.nih.gov/entrez/query.fcgi?cmd=search&db=gene&term=PTF1A) | pancreas specific transcription factor, 1a | [NM_178161](http://www.ncbi.nlm.nih.gov/entrez/query.fcgi?db=Nucleotide&term=NM_178161) | hs\|10p12.2 |
| 0.0085788 | 0.142 | 0.0238095 | 2.7 | 0.053 | 0.02 | [CEL](http://www.ncbi.nlm.nih.gov/entrez/query.fcgi?cmd=search&db=gene&term=CEL) | carboxyl ester lipase | [NM_001807](http://www.ncbi.nlm.nih.gov/entrez/query.fcgi?db=Nucleotide&term=NM_001807) | hs\|9q34.2 |
| 0.0074853 | 0.139 | 0.0238095 | 3.26 | 0.063 | 0.02 | [CEL](http://www.ncbi.nlm.nih.gov/entrez/query.fcgi?cmd=search&db=gene&term=CEL) | carboxyl ester lipase | [NM_001807](http://www.ncbi.nlm.nih.gov/entrez/query.fcgi?db=Nucleotide&term=NM_001807) | hs\|9q34.2 |
| 0.005762 | 0.137 | 0.0238095 | 4.4 | 0.082 | 0.02 | [TSGA13](http://www.ncbi.nlm.nih.gov/entrez/query.fcgi?cmd=search&db=gene&term=TSGA13) | testis specific, 13 | [NM_052933](http://www.ncbi.nlm.nih.gov/entrez/query.fcgi?db=Nucleotide&term=NM_052933) | hs\|7q32.2 |
| 0.0075392 | 0.139 | 0.015873 | 4.47 | 0.083 | 0.02 | [PNLIPRP1](http://www.ncbi.nlm.nih.gov/entrez/query.fcgi?cmd=search&db=gene&term=PNLIPRP1) | pancreatic lipase-related protein 1 | [NM_006229](http://www.ncbi.nlm.nih.gov/entrez/query.fcgi?db=Nucleotide&term=NM_006229) | hs\|10q25.3 |
| 0.0047392 | 0.137 | 0.0238095 | 4.05 | 0.072 | 0.02 | [DPP10-AS1](http://www.ncbi.nlm.nih.gov/entrez/query.fcgi?cmd=search&db=gene&term=DPP10-AS1) | DPP10 antisense RNA 1 | [NR_036580](http://www.ncbi.nlm.nih.gov/entrez/query.fcgi?db=Nucleotide&term=NR_036580) | hs\|2q14.1 |
| 0.0058675 | 0.137 | 0.0238095 | 5.66 | 0.093 | 0.02 | [MIR217HG](http://www.ncbi.nlm.nih.gov/entrez/query.fcgi?cmd=search&db=gene&term=MIR217HG) | MIR217 host gene (non-protein coding) | [NR_126406](http://www.ncbi.nlm.nih.gov/entrez/query.fcgi?db=Nucleotide&term=NR_126406) | hs\|2p16.1 |
| 0.0067161 | 0.137 | 0.031746 | 3.76 | 0.059 | 0.02 | [PDIA2](http://www.ncbi.nlm.nih.gov/entrez/query.fcgi?cmd=search&db=gene&term=PDIA2) | protein disulfide isomerase family A, member 2 | [NM_006849](http://www.ncbi.nlm.nih.gov/entrez/query.fcgi?db=Nucleotide&term=NM_006849) | hs\|16p13.3 |
| 0.0031158 | 0.137 | 0.015873 | 5.87 | 0.09 | 0.02 | [CTRL](http://www.ncbi.nlm.nih.gov/entrez/query.fcgi?cmd=search&db=gene&term=CTRL) | chymotrypsin-like | [NM_001907](http://www.ncbi.nlm.nih.gov/entrez/query.fcgi?db=Nucleotide&term=NM_001907) | hs\|16q22.1 |
| 0.0047979 | 0.137 | 0.0238095 | 4.56 | 0.063 | 0.01 | [LOC729159](http://www.ncbi.nlm.nih.gov/entrez/query.fcgi?cmd=search&db=gene&term=LOC729159) | UPF0607 protein ENSP00000381418-like | [NM_001282301](http://www.ncbi.nlm.nih.gov/entrez/query.fcgi?db=Nucleotide&term=NM_001282301) | hs\|16q21 |
| 0.0085806 | 0.142 | 0.0396825 | 4.13 | 0.054 | 0.01 | [SERPINI2](http://www.ncbi.nlm.nih.gov/entrez/query.fcgi?cmd=search&db=gene&term=SERPINI2) | serpin peptidase inhibitor, clade I (pancpin), member 2 | [NM_006217](http://www.ncbi.nlm.nih.gov/entrez/query.fcgi?db=Nucleotide&term=NM_006217) | hs\|3q26.1 |
| 0.0049416 | 0.137 | 0.0238095 | 3.72 | 0.045 | 0.01 | [GP2](http://www.ncbi.nlm.nih.gov/entrez/query.fcgi?cmd=search&db=gene&term=GP2) | glycoprotein 2 (zymogen granule membrane) | [XM_005255261](http://www.ncbi.nlm.nih.gov/entrez/query.fcgi?db=Nucleotide&term=XM_005255261) | hs\|16p12.3 |
| 0.00916 | 0.143 | 0.0238095 | 4.34 | 0.049 | 0.01 | [AQP12A](http://www.ncbi.nlm.nih.gov/entrez/query.fcgi?cmd=search&db=gene&term=AQP12A) | aquaporin 12A | [NM_198998](http://www.ncbi.nlm.nih.gov/entrez/query.fcgi?db=Nucleotide&term=NM_198998) | hs\|2q37.3 |
| 0.0040005 | 0.137 | 0.0238095 | 4.73 | 0.051 | 0.01 | [SYCN](http://www.ncbi.nlm.nih.gov/entrez/query.fcgi?cmd=search&db=gene&term=SYCN) | syncollin | [NM_001080468](http://www.ncbi.nlm.nih.gov/entrez/query.fcgi?db=Nucleotide&term=NM_001080468) | hs\|19q13.2 |
| 0.0038639 | 0.137 | 0.0238095 | 5.12 | 0.051 | 0.01 | [CTRC](http://www.ncbi.nlm.nih.gov/entrez/query.fcgi?cmd=search&db=gene&term=CTRC) | chymotrypsin C (caldecrin) | [NM_007272](http://www.ncbi.nlm.nih.gov/entrez/query.fcgi?db=Nucleotide&term=NM_007272) | hs\|1p36.21 |
| 0.0092186 | 0.143 | 0.031746 | 3.03 | 0.016 | 0.01 | [CELA3B](http://www.ncbi.nlm.nih.gov/entrez/query.fcgi?cmd=search&db=gene&term=CELA3B) | chymotrypsin-like elastase family, member 3B | [NM_007352](http://www.ncbi.nlm.nih.gov/entrez/query.fcgi?db=Nucleotide&term=NM_007352) | hs\|1p36.12 |
| 0.0042395 | 0.137 | 0.0238095 | 4.6 | 0.024 | 0.01 | [TMED11P](http://www.ncbi.nlm.nih.gov/entrez/query.fcgi?cmd=search&db=gene&term=TMED11P) | transmembrane emp24 protein transport domain containing 11, pseudogene |  | hs\|4p16.3 |

**Supporting Table 5: Functional analysis of genes significantly up‐ or down‐regulated in the stroma of PDAC**

| **A) Top 10 enriched Kegg pathways** | | | | | | | | | | | |
| --- | --- | --- | --- | --- | --- | --- | --- | --- | --- | --- | --- |
| Up-regulated genes in PDAC-stroma | | | | | | Down-regulated genes in PDAC-stroma | | | | | |
| Term | P-value | Adjusted P-value | Odds Ratio | Combined Score | Exemple of Genes | Term | P-value | Adjusted P-value | Odds Ratio | Combined Score | Genes |
| Cytokine-cytokine receptor interaction | 0.0001 | 0.0285 | 3.0234 | 28.0810 | CXCL3;TGFBR1;TNFSF9 | Mineral absorption | 0.0000 | 0.0003 | 6.2068 | 85.1291 | SLC6A19;MT1A;MT1M |
| ECM-receptor interaction | 0.0001 | 0.0176 | 5.4201 | 49.2107 | SDC4;LAMC2;ITGB6 | Glycine, serine and threonine metabolism | 0.0000 | 0.0011 | 6.4748 | 76.5844 | DMGDH;GAMT;GATM |
| Transcriptional misregulation in cancer | 0.0001 | 0.0150 | 3.5842 | 31.6648 | HOXA10; UP;BCL2A1 | Protein digestion and absorption | 0.0040 | 0.4151 | 2.8777 | 15.8585 | CELA3A;SLC6A19;CELA3B |
| TNF signaling pathway | 0.0002 | 0.0130 | 4.5455 | 39.4869 | CXCL10;MMP14;LIF;CXCL3 | Vitamin B6 metabolism | 0.0165 | 1.0000 | 9.5923 | 39.3820 | PSAT1;AOX1 |
| Small cell lung cancer | 0.0003 | 0.0169 | 4.7790 | 39.1901 | LAMB3;ITGA2;LAMA3;LAMC2; | Propanoate metabolism | 0.0240 | 1.0000 | 3.5971 | 13.4118 | ALDH6A1;ABAT;ACACB |
| Arrhythmogenic right ventricular cardiomyopathy | 0.0003 | 0.0160 | 5.4012 | 43.6071 | SGCD;JUP;ITGB6;DSC2 | Gastric acid secretion | 0.0461 | 1.0000 | 2.3022 | 7.0859 | MYLK2;ATP4A;CALML5;CALML6 |
| Pathways in cancer | 0.0007 | 0.0292 | 2.2013 | 16.1073 | PDGFRB;MMP1;LAMC2;GFBR1 | Fat digestion and absorption | 0.0531 | 1.0000 | 2.8075 | 8.2403 | PNLIPRP1;MOGAT3 |
| Focal adhesion | 0.0010 | 0.0385 | 3.0709 | 21.2157 | VASP;LAMC2;ITGB6;MET | Aldosterone synthesis and secretion | 0.0545 | 1.0000 | 2.0555 | 5.9803 | KCNJ5;CALML5;CALML6 |
| Central carbon metabolism in cancer | 0.0011 | 0.0377 | 5.1282 | 34.9250 | PDGFRB;SLC16A3;MET | Pancreatic secretion | 0.0545 | 1.0000 | 2.0555 | 5.9803 | PNLIPRP1;CELA3A;CELA3B |

| **B) Top 10 enriched Reactome pathways** | | | | | | | | | | | |
| --- | --- | --- | --- | --- | --- | --- | --- | --- | --- | --- | --- |
| Up-regulated genes in PDAC-stroma | | | | | | Down-regulated genes in PDAC-stroma | | | | | |
| Term | P-value | Adjusted P-value | Odds Ratio | Combined Score | Genes | Term | P-value | Adjusted P-value | Odds Ratio | Combined Score | Genes |
| Laminin interactions | 0.0000 | 0.0039 | 14.4928 | 186.7035 | LAMB3;ITGB4;ITGA3;ITGA2; | Response to metal ions Homo sapiens R-HSA-5660526 | 0.0000 | 0.0000 | 20.9287 | 458.4006 | MT1A;MT1M;MT1F |
| Type I hemidesmosome assembly | 0.0000 | 0.0093 | 24.6914 | 279.5403 | LAMB3;ITGB4;LAMA3;LAMC2 | Metallothioneins bind metals Homo sapiens R-HSA-5661231 | 0.0000 | 0.0000 | 20.9287 | 458.4006 | MT1A;MT1M;MT1F; |
| Cell junction organization | 0.0000 | 0.0124 | 5.8140 | 61.7546 | VASP;CLDN4;LAMB3;JUP;ITGB4; | Metabolism of ingested SeMet, Sec, MeSec into H2Se Homo sapiens R-HSA-2408508 | 0.0021 | 1.0000 | 10.7914 | 66.7833 | CBS;MAT1A;GNMT |
| Extracellular matrix organization | 0.0001 | 0.0226 | 3.1410 | 30.5805 | MMP7;CEACAM6;ADAM9;ITGB6 | ERBB2 Activates PTK6 Signaling Homo sapiens R-HSA-8847993 | 0.0092 | 1.0000 | 6.6408 | 31.1307 | EGF;ERBB4;NRG4 |
| Cell surface interactions at the vascular wall | 0.0001 | 0.0268 | 4.9505 | 46.2612 | CEACAM1;CEACAM6;CD58;SLC16A3 | ERBB2 Regulates Cell Motility Homo sapiens R-HSA-6785631 | 0.0139 | 1.0000 | 5.7554 | 24.6046 | EGF;ERBB4;NRG4 |
| Non-integrin membrane-ECM interactions | 0.0001 | 0.0252 | 7.9365 | 73.1767 | SDC4;LAMB3;TGA2;LAMA3;LAMC2 | Choline catabolism Homo sapiens R-HSA-6798163 | 0.0165 | 1.0000 | 9.5923 | 39.3820 | DMGDH;SARDH |
| Degradation of the extracellular matrix | 0.0001 | 0.0278 | 4.7170 | 42.3146 | CAPN8;MMP14;ADAM10;ADAM9 | PI3K events in ERBB2 signaling Homo sapiens R-HSA-1963642 | 0.0167 | 1.0000 | 5.3957 | 22.0853 | EGF;ERBB4;NRG4 |
| O-linked glycosylation Homo sapiens | 0.0002 | 0.0323 | 4.5455 | 39.4869 | SPON2;GALNT5;ADAMTS12 | GRB2 events in ERBB2 signaling Homo sapiens R-HSA-1963640 | 0.0167 | 1.0000 | 5.3957 | 22.0853 | EGF;ERBB4;NRG4 |
| O-linked glycosylation of mucins | 0.0002 | 0.0307 | 5.8923 | 50.7844 | GALNT5;MUC13;GCNT3 | Zinc transporters Homo sapiens R-HSA-435354 | 0.0197 | 1.0000 | 5.0783 | 19.9305 | SLC30A2;SLC39A5; |
| Anchoring fibril formation Homo sapiens R-HSA-2214320 | 0.0002 | 0.0294 | 23.8095 | 203.7831 | LAMB3;LAMA3;LAMC2 | Digestion of dietary lipid Homo sapiens R-HSA-192456 | 0.0197 | 1.0000 | 5.0783 | 19.9305 | PNLIPRP1;CEL;LMF2 |

| **C) Top 10 enriched Wikipathway 2019** | | | | | | | | | | | |
| --- | --- | --- | --- | --- | --- | --- | --- | --- | --- | --- | --- |
| Up-regulated genes in PDAC-stroma | | | | | | Down-regulated genes in PDAC-stroma | | | | | |
| Term | P-value | Adjusted P-value | Odds Ratio | Combined Score | Genes | Term | P-value | Adjusted P-value | Odds Ratio | Combined Score | Genes |
| Focal Adhesion | 0.0001 | 0.0307 | 3.6476 | 35.1680 | VASP;PDGFRB;LAMC2;ITGB6;MET | Zinc homeostasis WP3529 | 0.0000 | 0.0000 | 8.7930 | 154.8741 | MT1A;SLC30A2;MT1M |
| Arrhythmogenic Right Ventricular Cardiomyopathy | 0.0004 | 0.0871 | 5.2553 | 41.5377 | SGCD;JUP;ITGB4;ITGA3;ITGA2;ITGB6 | Copper homeostasis WP3286 | 0.0001 | 0.0160 | 4.9806 | 47.8182 | BACE1;STEAP3;MT1A |
| Photodynamic therapy-induced HIF-1 survival signaling | 0.0005 | 0.0785 | 7.5075 | 57.0810 | PKM;BCL2A1;SLC2A1;PMAIP1;BAK1 | Folate-Alcohol and Cancer Pathway Hypotheses WP1589 | 0.0030 | 0.4720 | 9.5923 | 55.7235 | CBS;ALDH1A1;CYP2E1 |
| TYROBP Causal Network | 0.0008 | 0.0929 | 5.4645 | 39.0570 | SLC7A7;KCNE3;TGFBR1;CXCL16 | Methionine metabolism leading to Sulphur Amino Acids and related disorders WP4292 | 0.0056 | 0.6601 | 7.8483 | 40.7013 | CBS;MAT1A;GNMT |
| Apoptosis | 0.0008 | 0.0753 | 4.6296 | 33.0282 | HELLS;PMAIP1;NFKBIE; | Secretion of Hydrochloric Acid in Parietal Cells WP2597 | 0.0112 | 1.0000 | 11.5108 | 51.6590 | ATP4A;HRH2 |
| Prostaglandin Synthesis and Regulation | 0.0012 | 0.0978 | 6.1728 | 41.2965 | EDNRA;ANXA2;AKR1C3;S100A6 | Notch Signaling WP268 | 0.0194 | 1.0000 | 3.1974 | 12.6076 | RBPJL;JAG2;KCNJ5; |
| Hippo-Merlin Signaling Dysregulation | 0.0015 | 0.1005 | 3.7037 | 24.1053 | CXCL10;ITGB4;ITGB6;MET | Trans-sulfuration and one carbon metabolism WP2525 | 0.0216 | 1.0000 | 3.7132 | 14.2397 | CBS;PSAT1;PHGDH;MAT1A |
| Alpha 6 Beta 4 signaling pathway | 0.0028 | 0.1650 | 6.7340 | 39.5907 | LAMB3;ITGB4;LAMA3;LAMC2 | EV release from cardiac cells and their functional effects WP3297 | 0.0225 | 1.0000 | 8.2220 | 31.1794 | CXCL12;ERBB4 |
| Focal Adhesion-PI3K-Akt-mTOR-signaling pathway | 0.0035 | 0.1846 | 2.3836 | 13.4656 | PDGFRB;ITGB4;TGB6;MET | PTF1A related regulatory pathway WP4147 | 0.0539 | 1.0000 | 5.2322 | 15.2835 | RBPJL;PTF1A |
| Apoptosis Modulation and Signaling WP1772 | 0.0060 | 0.2847 | 3.6630 | 18.7209 | BCL2A1;BIK;PMAIP1 | Vitamin A and Carotenoid Metabolism WP716 | 0.0614 | 1.0000 | 2.6769 | 7.4705 | DGAT1;CRABP1;ALDH1A1 |

| **D) Top 10 enriched Biocarta 2016 pathways** | | | | | | | | | | | |
| --- | --- | --- | --- | --- | --- | --- | --- | --- | --- | --- | --- |
| Up-regulated genes in PDAC-stroma | | | | | | Down-regulated genes in PDAC-stroma | | | | | |
| Term | P-value | Adjusted P-value | Odds Ratio | Combined Score | Genes | Term | P-value | Old Adjusted P-value | Odds Ratio | Combined Score | Genes |
| Role of Mitochondria in Apoptotic Signaling Homo sapiens h mitochondriaPathway | 0.0014 | 0.3428 | 12.8205 | 83.8288 | BIK;BAK1;BIRC3 | g-Secretase mediated ErbB4 Signaling Pathway Homo sapiens h erbB4pathway | 0.0294 | 0.0000 | 7.1942 | 25.3780 | ERBB4;NRG4 |
| Prion Pathway Homo sapiens h prionPathway | 0.0039 | 0.4575 | 9.2593 | 51.4528 | LAMB3;LAMA3;LAMC2 | Corticosteroids and cardioprotection Homo sapiens h gcrPathway | 0.2152 | 0.0000 | 2.3022 | 3.5369 | CXCL12;NPPA |
| Beta-arrestins in GPCR Desensitization Homo sapiens h bArrestinPathway | 0.0136 | 1.0000 | 5.9524 | 25.5915 | EDNRA;P2RY2;CXCR4 | Mechanism of Acetaminophen Activity and Toxicity Homo sapiens h AcetaminophenPathway | 0.2193 | 0.0000 | 4.1110 | 6.2369 | CYP2E1 |
| Activation of cAMP-dependent protein kinase, PKA Homo sapiens h gsPathway | 0.0149 | 0.8855 | 5.7471 | 24.1572 | EDNRA;P2RY2;CXCR4 | CBL mediated ligand-induced downregulation of EGF receptors Homo sapiens h cblPathway | 0.2465 | 0.0000 | 3.5971 | 5.0377 | EGF |
| Role of Beta-arrestins in the activation and targeting of MAP kinases Homo sapiens h barr-mapkPathway | 0.0164 | 0.7768 | 5.5556 | 22.8397 | EDNRA;P2RY2;CXCR4 | Pertussis toxin-insensitive CCR5 Signaling in Macrophage Homo sapiens h Ccr5Pathway | 0.2727 | 0.0000 | 3.1974 | 4.1550 | CXCL12 |
| TNFR2 Signaling Pathway Homo sapiens h tnfr2Pathway | 0.0189 | 0.7477 | 9.2593 | 36.7325 | TNFAIP3;TRAF1 | Alternative Complement Pathway Homo sapiens h alternativePathway | 0.2980 | 0.0000 | 2.8777 | 3.4843 | C5 |
| Roles of Beta-arrestin-dependent Recruitment of Src Kinases in GPCR Signaling Homo sapiens h bArrestin-srcPathway | 0.0229 | 0.7760 | 4.9020 | 18.5086 | EDNRA;P2RY2;CXCR4 | G-Protein Signaling Through Tubby Proteins Homo sapiens h tubbyPathway | 0.2980 | 0.0000 | 2.8777 | 3.4843 | CXCL12 |
| IL 4 signaling pathway Homo sapiens h il4Pathway | 0.0291 | 0.8610 | 7.4074 | 26.2091 | IL4R;HMGA1 | CXCR4 Signaling Pathway Homo sapiens h cxcr4Pathway | 0.3224 | 0.0000 | 2.6161 | 2.9616 | CXCL12 |
| ChREBP regulation by carbohydrates and cAMP Homo sapiens h chrebpPathway | 0.0350 | 0.9214 | 4.1667 | 13.9697 | EDNRA;P2RY2;CXCR4 | EPO Signaling Pathway Homo sapiens h epoPathway | 0.3224 | 0.0000 | 2.6161 | 2.9616 | EPO |
| Activation of Csk by cAMP-dependent Protein Kinase Inhibits Signaling through the T Cell Receptor Homo sapiens h CSKPathway | 0.0420 | 0.9961 | 3.8760 | 12.2842 | EDNRA;P2RY2;CXCR4 | Activation of PKC through G-protein coupled receptors Homo sapiens h pkcPathway | 0.3224 | 1.0000 | 2.6161 | 2.9616 | CXCL12 |

| **E) Top 10 enriched GO Biological process** | | | | | | | | | | | |
| --- | --- | --- | --- | --- | --- | --- | --- | --- | --- | --- | --- |
| Up-regulated genes in PDAC-stroma | | | | | | Down-regulated genes in PDAC-stroma | | | | | |
| Term | P-value | Adjusted P-value | Odds Ratio | Combined Score | Genes | Term | P-value | Adjusted P-value | Odds Ratio | Combined Score | Genes |
| proteolysis (GO:0006508) | 0.0000 | 0.1177 | 3.2455 | 34.6543 | MMP7;MMP1;ADAMTS12; | cellular zinc ion homeostasis (GO:0006882) | 0.0000 | 0.0000 | 10.2112 | 198.2425 | MT1A;MT1M;MT1F |
| cell-substrate junction assembly (GO:0007044) | 0.0000 | 0.0787 | 20.2020 | 209.8416 | LAMB3;ITGB4;LAMA3;LAMC2 | zinc ion homeostasis (GO:0055069) | 0.0000 | 0.0000 | 10.2112 | 198.2425 | MT1A;MT1M;MT1F |
| hemidesmosome assembly (GO:0031581) | 0.0000 | 0.0524 | 20.2020 | 209.8416 | LAMB3;ITGB4;LAMA3;LAMC2 | cellular response to zinc ion (GO:0071294) | 0.0000 | 0.0000 | 13.6312 | 261.2587 | MT1A;MT1M |
| skin development (GO:0043588) | 0.0000 | 0.0634 | 5.3191 | 52.7088 | SCEL;SFN;MET;EPHA2 | cellular response to copper ion (GO:0071280) | 0.0000 | 0.0000 | 12.3330 | 222.8733 | MT1A;MT1M;MT1F |
| O-glycan processing (GO:0016266) | 0.0002 | 0.1674 | 5.9829 | 52.1446 | GALNT5;MUC13;GCNT3 | response to zinc ion (GO:0010043) | 0.0000 | 0.0001 | 8.9928 | 147.2199 | MT1A;SLC30A2 |
| response to tumor necrosis factor (GO:0034612) | 0.0004 | 0.3114 | 4.5819 | 36.2549 | CCL20;ADAMTS12;TNFRSF21 | response to copper ion (GO:0046688) | 0.0000 | 0.0001 | 10.3597 | 168.4225 | MT1A;MT1M |
| endoderm formation (GO:0001706) | 0.0004 | 0.3192 | 7.7160 | 59.6733 | DUSP5;MMP14;NHBA | cellular response to cadmium ion (GO:0071276) | 0.0000 | 0.0003 | 8.9308 | 132.1095 | MT1A;MT1M |
| mesodermal cell differentiation (GO:0048333) | 0.0004 | 0.2859 | 18.5185 | 142.7828 | ITGB4;ITGA3;ITGA2 | response to cadmium ion (GO:0046686) | 0.0000 | 0.0005 | 8.3546 | 118.2621 | MT1A;MT1M |
| extracellular matrix disassembly (GO:0022617) | 0.0005 | 0.2891 | 4.9858 | 37.7989 | MMP14;LAMA3;ADAM10;LAMC2 | cellular divalent inorganic cation homeostasis (GO:0072503) | 0.0002 | 0.1127 | 3.4190 | 29.1404 | MT1A;CXCL12 |
| regulation of chemotaxis (GO:0050920) | 0.0008 | 0.4209 | 9.2593 | 65.7431 | PDGFRB;EFNB2;CXCR4;ZSWIM4 | cellular transition metal ion homeostasis (GO:0046916) | 0.0004 | 0.1927 | 3.4037 | 26.8268 | MT1A;MT1M |

| **F) Top 10 enriched GO Molecular fonction** | | | | | | | | | | | |
| --- | --- | --- | --- | --- | --- | --- | --- | --- | --- | --- | --- |
| Up-regulated genes in PDAC-stroma | | | | | | Down-regulated genes in PDAC-stroma | | | | | |
| Term | P-value | Adjusted P-value | Odds Ratio | Combined Score | Genes | Term | P-value | Adjusted P-value | Odds Ratio | Combined Score | Genes |
| metalloendopeptidase activity (GO:0004222) | 0.0002 | 0.1888 | 5.9829 | 52.1446 | MMP14;ADAM9;ADAMTS12 | aspartic-type peptidase activity (GO:0070001) | 0.0197 | 1.0000 | 5.0783 | 19.9305 | BACE1;PGA3;PGC |
| chemokine activity (GO:0008009) | 0.0002 | 0.0958 | 7.2464 | 63.0507 | CXCL10; CXCL3;CKLF;CXCL16 | metal ion binding (GO:0046872) | 0.0213 | 1.0000 | 1.5626 | 6.0120 | PNLIPRP1;BRSK2;MT1M |
| cadherin binding (GO:0045296) | 0.0002 | 0.0729 | 2.8399 | 24.3320 | VASP;LAD1;JUP;ANXA2;CAPG | aspartic-type endopeptidase activity (GO:0004190) | 0.0349 | 1.0000 | 4.1110 | 13.7965 | BACE1;PGA3;PGC |
| chemokine receptor binding (GO:0042379) | 0.0002 | 0.0683 | 6.8027 | 56.7726 | CXCL10;CCL20;CXCL1;CXCL3; | zinc ion transmembrane transporter activity (GO:0005385) | 0.0394 | 1.0000 | 3.9241 | 12.6939 | SLC30A2;SLC39A5;SLC39A8 |
| actin binding (GO:0003779) | 0.0007 | 0.1699 | 2.8434 | 20.5057 | TPM4;CXCR4;ABRACL;CEACAM1; | neurexin family protein binding (GO:0042043) | 0.0451 | 1.0000 | 5.7554 | 17.8355 | SYTL1;CEL |
| low-density lipoprotein receptor activity (GO:0005041) | 0.0014 | 0.2775 | 12.8205 | 83.8288 | TNFAIP6;LRP8;CXCL16 | sodium:potassium-exchanging ATPase activity (GO:0005391) | 0.0539 | 1.0000 | 5.2322 | 15.2835 | ATP4A;ATP1A2 |
| fucosyltransferase activity (GO:0008417) | 0.0014 | 0.2378 | 12.8205 | 83.8288 | FUT6;FUT2;FUT3 | potassium-transporting ATPase activity (GO:0008556) | 0.0539 | 1.0000 | 5.2322 | 15.2835 | ATP4A;ATP1A2 |
| beta-1,3-galactosyltransferase activity (GO:0048531) | 0.0018 | 0.2613 | 11.9048 | 75.1291 | B3GNT5;C1GALT1;B3GNT3 | peptide hormone receptor binding (GO:0051428) | 0.0539 | 1.0000 | 5.2322 | 15.2835 | GPHA2;GPHB5 |
| endopeptidase activity (GO:0004175) | 0.0020 | 0.2569 | 2.3540 | 14.6193 | MMP7;MMP1;ADAMTS12;KLK6; | microtubule-severing ATPase activity (GO:0008568) | 0.0539 | 1.0000 | 5.2322 | 15.2835 | FIGNL2;KATNB1 |
| cytokine activity (GO:0005125) | 0.0020 | 0.2324 | 3.2258 | 20.0160 | CXCL10;IL1RN;CCL20 | C-acyltransferase activity (GO:0016408) | 0.0632 | 1.0000 | 4.7962 | 13.2445 | ACSM6;ACAT1 |

**Supporting Figure 1: Flowchart of study design**

**
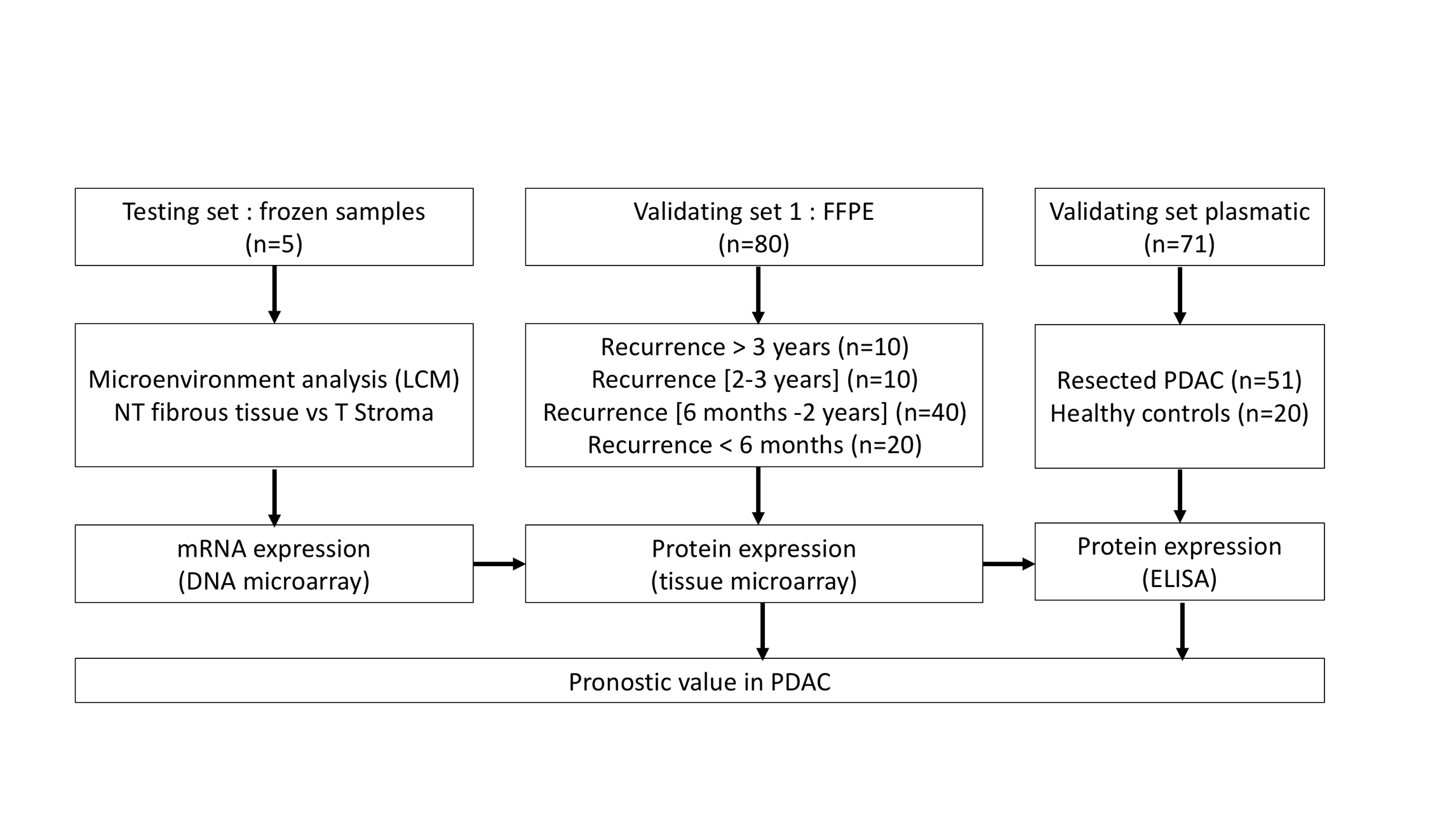
**

**Supporting Figure 2: Functional analysis of the PDAC stroma signature**

Gene set enrichment analysis (GSEA) using the gene expression profiles of PDAC tumoral microenvironment (left side) and adjacent NT fibrous tissue (right side).


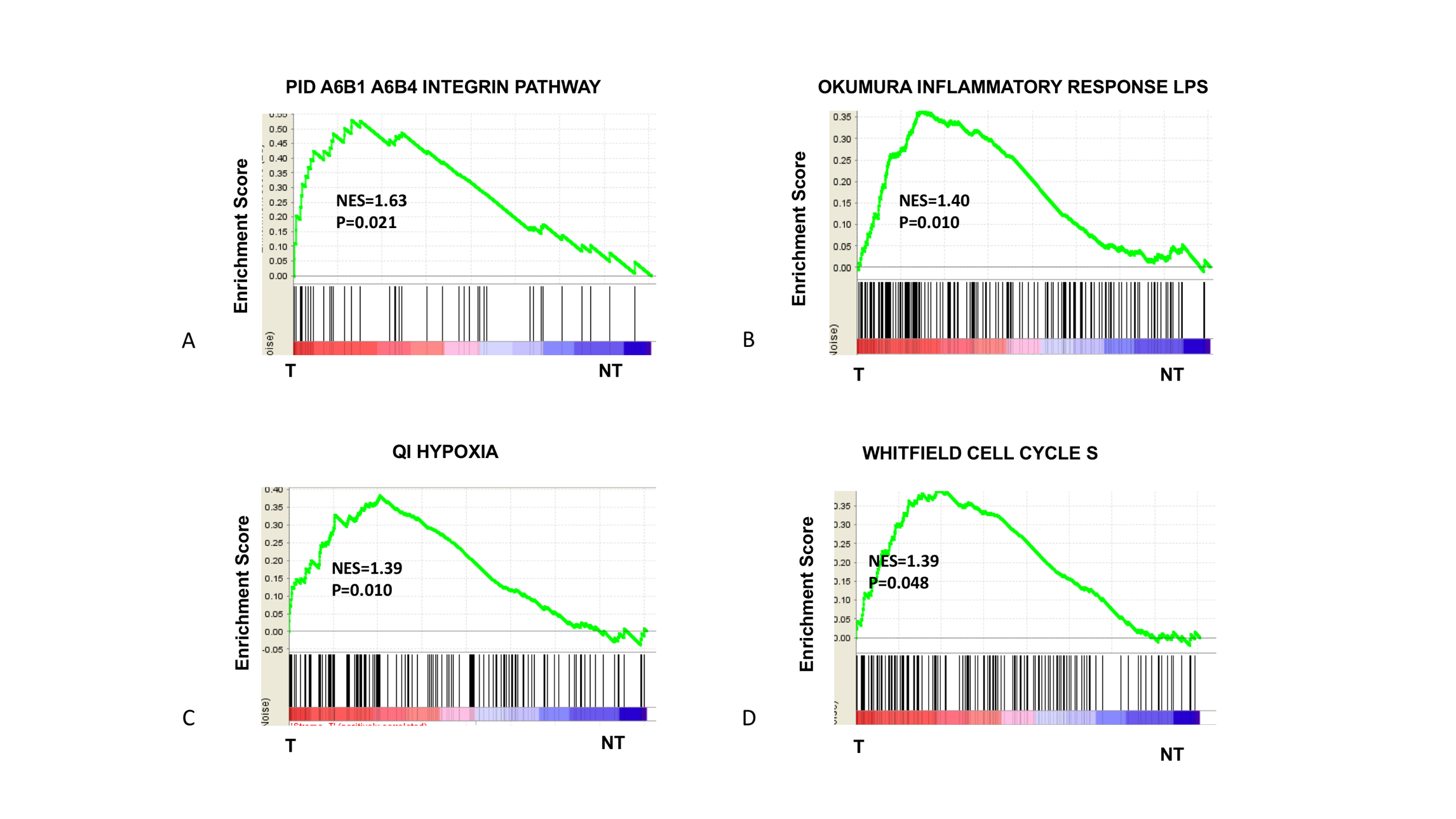


**Supporting Figure 3 : No association between epithelial expression of SFN and survival**

Analysis based solely on tumour epithelial cell expression revealed no significant association of SFN expression with OS (p=0.128) and DFS (p=0.160).


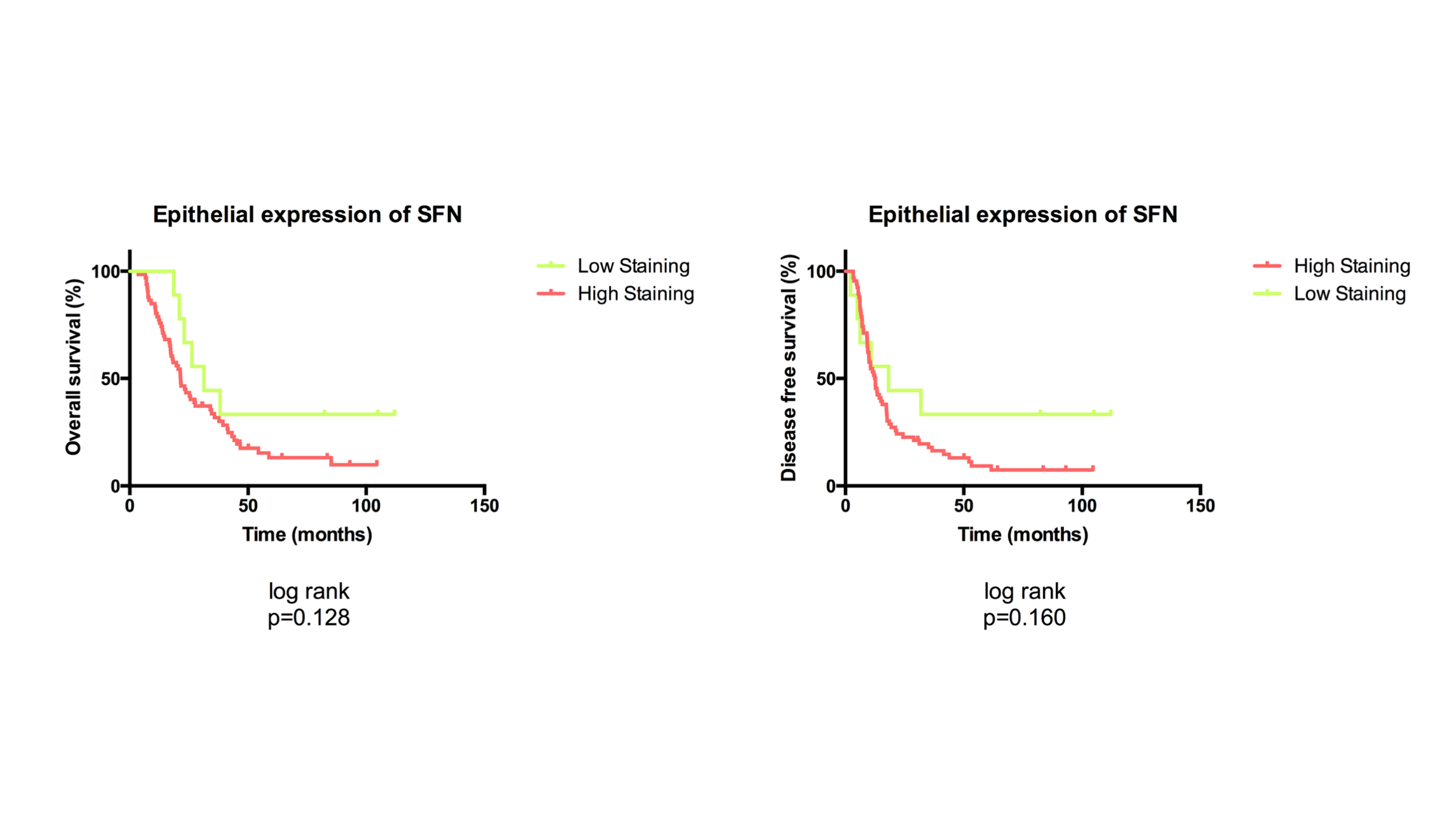


**Supporting Figure 4 : The prognostic value of SFN in PDAC is confirmed in the TCGA database**

High expression of SFN was associated with a statistically significant reduction of both OS and DFS (P<0.05) in and independant set of 178 bulk tumors.

**
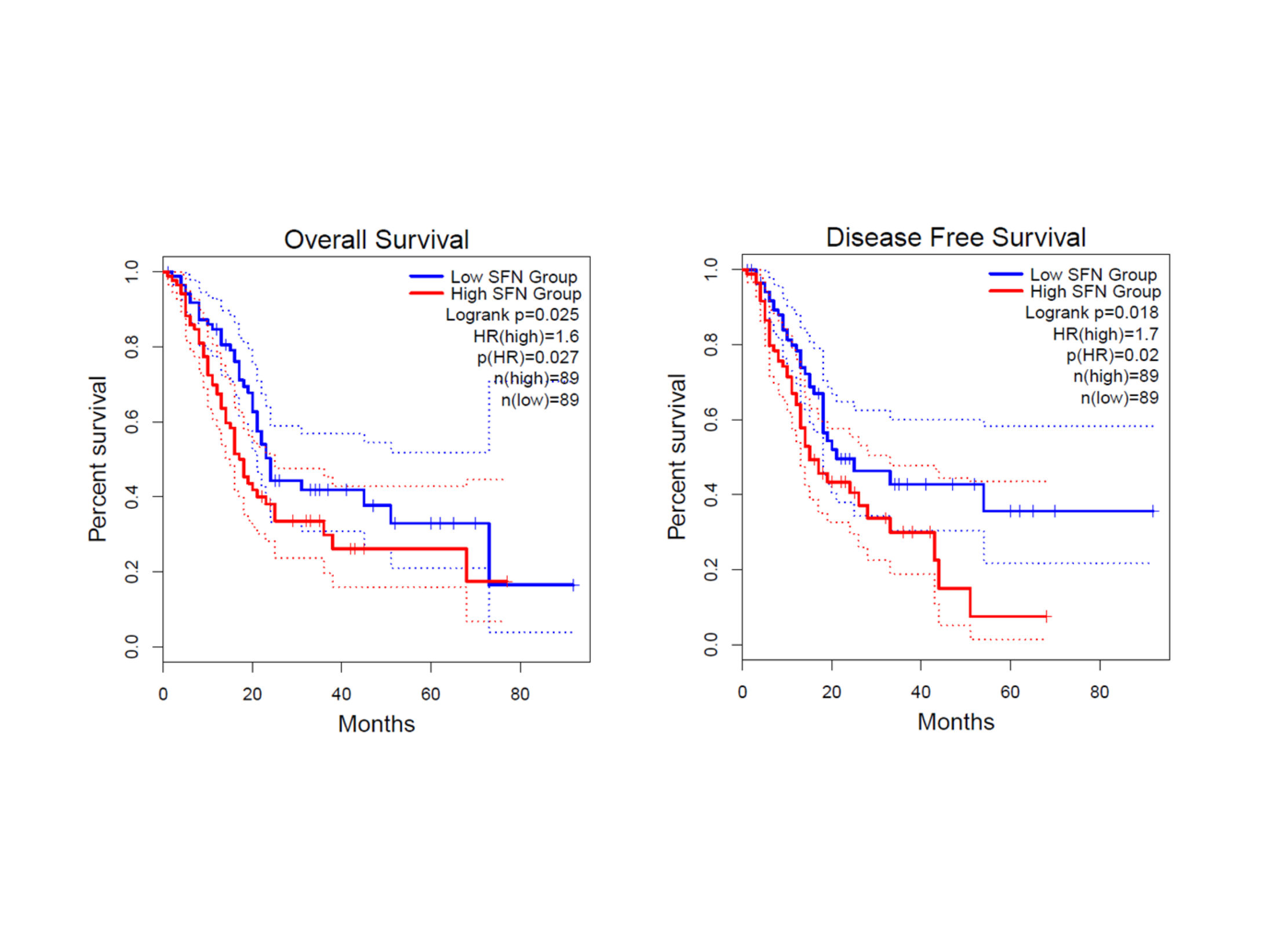
**

**Supporting Figure 5: The SFN protein is secreted plasma of PDAC**

The expression of SFN in 51 plasmatic samples of resected PDAC exhibits a trend toward overexpression compared to healthy controls without statistically significant difference (p=0.223). Non parametric Wilcoxon- Mann-Whitney test.


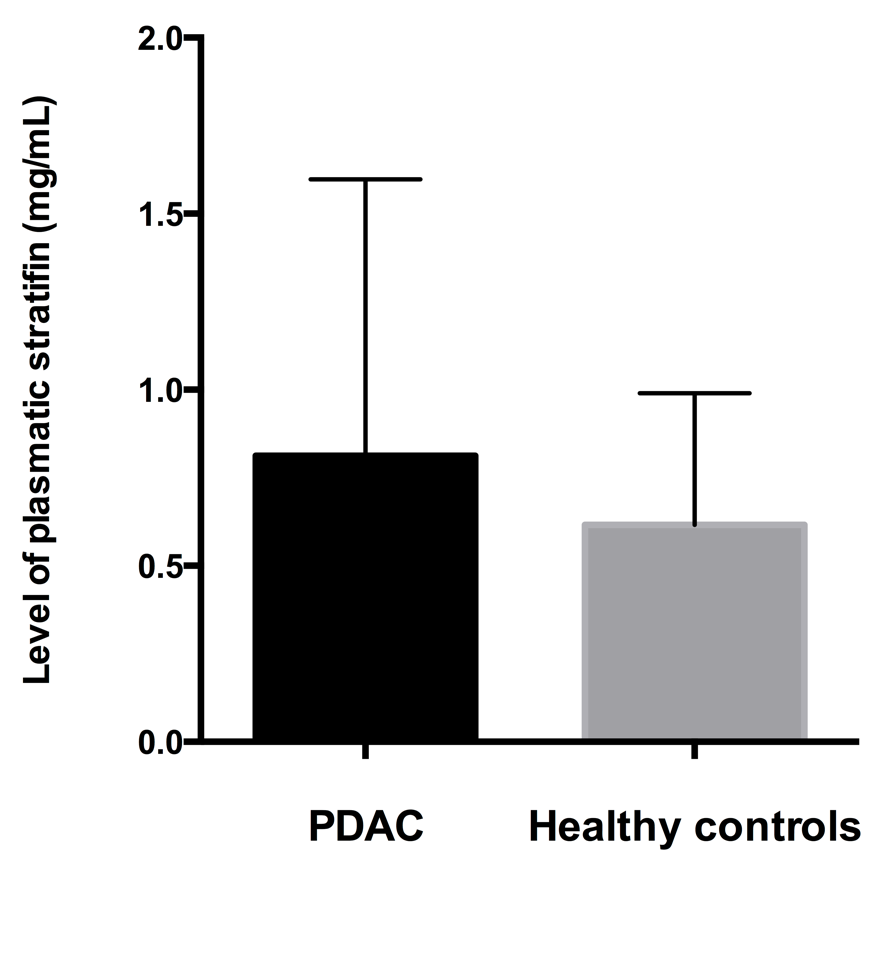


**Supporting Figure 6 : High level of SFN plasmatic expression is associated with reccurence**

Kaplan-Meier curves and log-rank analysis of overall survival (OS) (left panels) and disease-free survival (DFS) (right panels) according to the plasmatic expression of SFN. The threshold determing high (n=9) and low (n=42) expression is 1.043ng/mL.


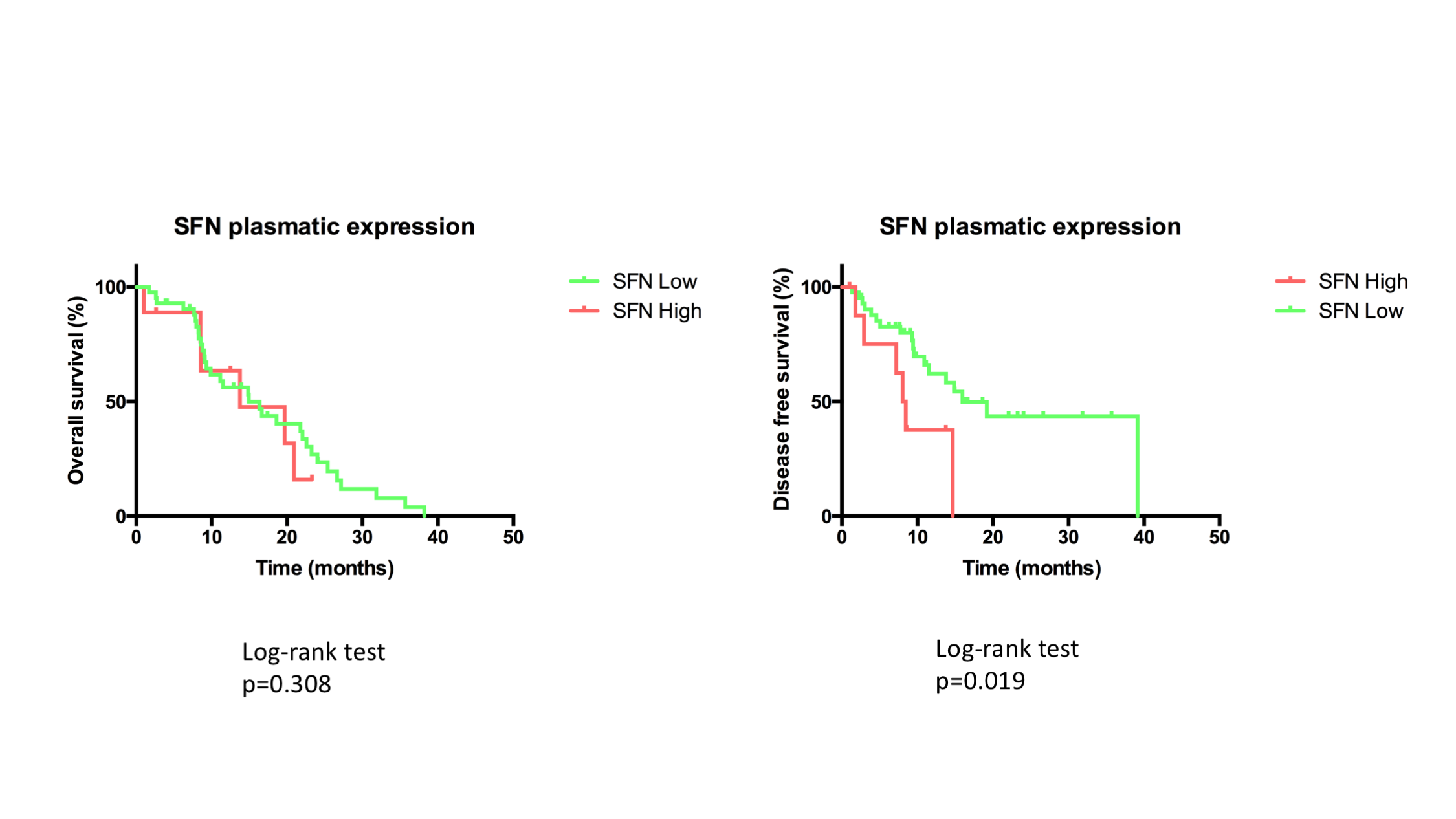

Supplement: Supplementary file 1 — Supplementary Figures and Tables [file 41416_2020_863_MOESM1_ESM.docx]
